# *De novo* synthesis, structural assignment and biological evaluation of pseudopaline, a metallophore produced by *Pseudomonas aeruginosa*†
†Electronic supplementary information (ESI) available. CCDC 1850196 and 1863986. For ESI and crystallographic data in CIF or other electronic format see DOI: 10.1039/c9sc01405e


**DOI:** 10.1039/c9sc01405e

**Published:** 2019-05-30

**Authors:** Jian Zhang, Tianhu Zhao, Rongwen Yang, Ittipon Siridechakorn, Sanshan Wang, Qianqian Guo, Yingjie Bai, Hong C. Shen, Xiaoguang Lei

**Affiliations:** a Beijing National Laboratory for Molecular Sciences , Key Laboratory of Bioorganic Chemistry and Molecular Engineering of Ministry of Education , Department of Chemical Biology , College of Chemistry and Molecular Engineering , Synthetic and Functional Biomolecules Center , Peking-Tsinghua Center for Life Sciences , Peking University , Beijing 100871 , China . Email: xglei@pku.edu.cn; b Roche Innovation Center Shanghai , Roche Pharma Research & Early Development , Shanghai 201203 , China

## Abstract

Pseudopaline is an opine carboxylate metallophore produced by *Pseudomonas aeruginosa* for harvesting divalent metals. However, the structure of pseudopaline is not fully elucidated. Herein, we report the first *de novo* total synthesis and isolation of pseudopaline, which allows unambiguous determination and confirmation of both the absolute and the relative configuration of the natural product. The synthesis highlights an efficient and stereocontrolled route using the asymmetric Tsuji–Trost reaction as the key step. The preliminary structure–activity relationship study indicated that one pseudopaline derivative shows comparable activity to pseudopaline. Moreover, a pseudopaline-fluorescein conjugate was prepared and evaluated, which confirmed that pseudopaline could be transported in the bacteria. Since the metal acquisition by *P. aeruginosa* is crucial for its ability to cause diseases, our extensive structural and functional studies of pseudopaline may pave the way for developing new therapeutic strategies such as the “Trojan horse” antibiotic conjugate against *P. aeruginosa*.

## Abstract 2

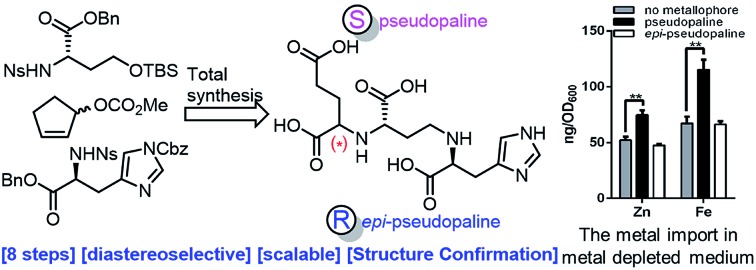
The first total synthesis and isolation of pseudopaline was reported, which allows determination and confirmation of the absolute configuration of the natural product.

## Introduction

Bacterial resistance, especially from Gram-negative bacteria against most antibiotics, poses a huge threat to public health.^1^*Pseudomonas aeruginosa* infection has become a severe problem for nosocomial immuno-compromised patients, which put the bacteria as one of the three “critical priority pathogens” on the WHO priority list.^2^,^3^ Despite an increased awareness of combating *P. aeruginosa* infection, little progress has been achieved. Therefore, novel and effective treatment options are in great demand.

**Fig. 1:**
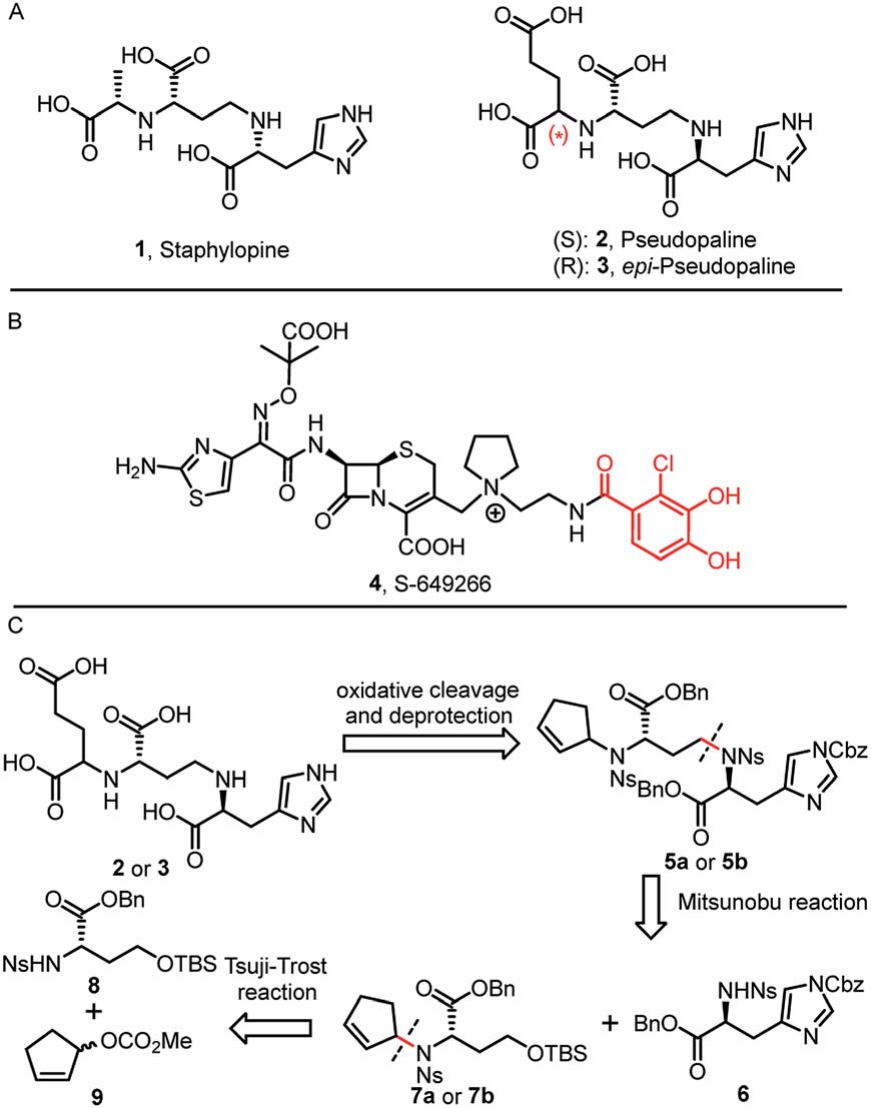
(A) Chemical structures of staphylopine (**1**), pseudopaline (**2**) and *epi*-pseudopaline (**3**). (B) “Trojan horse” antibiotic-conjugate S-649266 or cefiderocol (**4**) currently in phase III clinical trials. (C) Retrosynthetic analysis for pseudopaline.

The permeability barrier of outer-membranes is one of the significant factors which contributes to the antibiotic resistance of Gram-negative bacteria.^4^ Therefore, deciphering the multiple strategies that bacteria employ for proliferation and invasion is crucial for developing new therapeutics.^5^ For example, transition metals (Mn, Fe, Co, Ni, Cu and Zn) are essential micronutrients that bacterial pathogens must acquire during the host–pathogen interactions. Bacteria have developed a metal acquisition strategy to survive under metal depleted conditions. One common strategy involves the biosynthesis of high-affinity ferric siderophores.^6^ Exploitation of this essential bacterial reliance on competing for iron can potentially help the development of new antibiotic therapies, such as “Trojan horse” siderophore-antibiotic conjugates.^7^ In fact, the most advanced drug candidate S-649266 (**4**) has been extensively evaluated in phase III clinical trials (Fig. 1B).7*h*

In 2016, Arnoux and co-workers described a new type of metallophore, called staphylopine (**1**) (Fig. 1A), that contributes to the broad-spectrum metal uptake capabilities in *Staphylococcus aureus*.^8^ Importantly, homologous biosynthetic enzymes are also encoded by other microbial pathogens, such as the Gram-negative bacteria *P. aeruginosa*. Indeed, this staphylopine-like metallophore, named pseudopaline (**2**), is biosynthesized from two enzymes, PaCntL and PaCntM.^9^–^11^ Recently, a series of studies indicated that the pseudopaline operon contributes to *P. aeruginosa* virulence. The pseudopaline receptor ZrmA exhibits great relevance to lung infections in cystic fibrosis patients, and the pseudopaline operon was dramatically up-regulated in the infected host sites, suggesting that the pseudopaline production is closely correlated with the *P. aeruginosa* pathogenicity.^12^–^14^ However, pseudopaline has never been isolated from nature and the stereochemistry at the glutaric acid moiety remains unclear (Fig. 1A). Therefore, total synthesis represents the practical means by which the correct structure of pseudopaline (**2**) can be elucidated unambiguously.^15^

Herein, we report the first total synthesis of pseudopaline (**2**) and *epi*-pseudopaline (**3**) using the asymmetric Tsuji–Trost reaction and Mitsunobu reaction as the key steps. Compared with the natural pseudopaline sample isolated from *P. aeruginosa*, we have unambiguously determined and confirmed both the absolute and the relative configuration of the natural product. Moreover, a pseudopaline-fluorescein conjugate (P-FL) **16** was prepared and evaluated. The results showed that **16** could be transported in *P. aeruginosa*. This work offers new chemical and biological insights into this crucial metallophore from Gram-negative bacteria, and a foundation for future drug discovery.

## Results and discussion

Inspired by our previous synthetic studies towards carboxylate type metallophores,^16^ our retrosynthetic analysis is outlined in Fig. 1C. Due to the steric hindrance of the C–N bond at the glutaric acid moiety, no reaction was observed under the previously established Mitsunobu conditions. Therefore, we decided to invent a new approach for the synthesis of pseudopaline. The glutaric acid moiety of pseudopaline may be derived from oxidative cleavage and deprotection from **5**. Cyclopentene **5** could be derived from the key intermediate **7** and l-histidine derivative **6** through the Mitsunobu reaction. We envisioned that the asymmetric Tsuji–Trost reaction might serve as a key step for the diastereoselective construction of **7** from cyclopentenyl carbonate **9** and l-homoserine derivative **8**.

The asymmetric Tsuji–Trost reaction has been widely used in the synthesis of biologically active natural products.17*a* But to our knowledge, *N*-cyclopentenyl asymmetric allylation using unsubstituted cyclopentene is rare.17b,c Our synthesis of pseudopaline commenced with deprotection of **10**16*b* with TFA to yield free amine followed by reprotection with an *o*-nosyl group to afford the precursor **10a** in moderate yield (Scheme S1†). We then screened the asymmetric Tsuji–Trost reaction conditions with **10a** and **9** using Pd_2_(dba)_3_·CHCl_3_ and ligand **L1** in THF at 0 °C for 2 h, which mainly gave the allylation product with debenzylation of carboxylic acid (Table 1, entry 1). We hypothesized that the free hydroxyl group in **10a** coordinates with Pd(0), which induces the hydrogenolysis of the benzyl group. l-Homoserine **10a** was then protected with TBSCl to give compound **8** (Scheme S1†). Extensive ligand examination revealed that naphtho-derived Trost ligand **L2** provided an excellent diastereoselectivity with 27 : 1 d.r. (entry 3). To our delight, we could also obtain another diastereoisomer with high stereoselectivity using ***ent*-L2** (entry 8), suggesting that there was complete reagent control. The scope of this transformation using various *o*-nosyl protected amino acid derivatives was explored (Table 2). Good to excellent yields were obtained in all cases (absolute structure of **7c** was unambiguously confirmed by X-ray crystallography and **7d** was confirmed by chemical synthesis, see the ESI† for details). These results indicate that the Tsuji–Trost conditions can be applied to synthesize various isomers of pseudopaline as well as other pseudopaline analogs for further structure–activity relationship study.

**Table 1:** Optimization of the asymmetric Tsuji–Trost reaction^*a*^

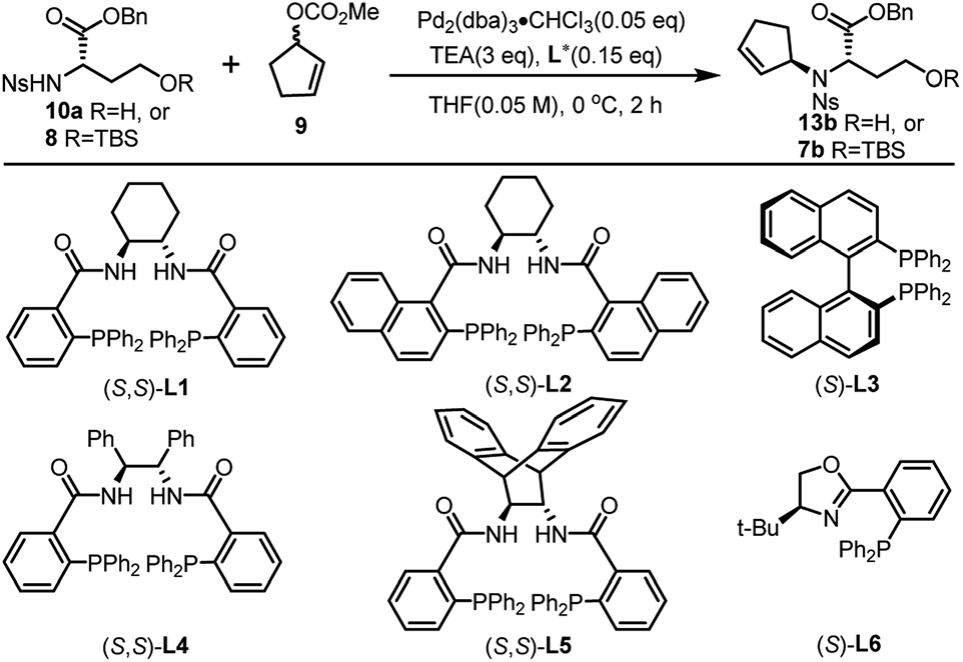				
Entry	R	Lig.	Conv.^*b*^	d.r.^*c*^
1^*d*^	H	**L1**	Full	2 : 1
2	TBS	**L1**	Full	2 : 1
3	TBS	**L2**	Full	27 : 1
4	TBS	**L3**	Trace	—
5	TBS	**L4**	92%	3.8 : 1
6	TBS	**L5**	Trace	—
7	TBS	**L6**	48%	1.9 : 1
8^*e*^	TBS	***ent*-L2**	Full	1 : 27

^*a*^Reaction conditions: 0.1 mmol of **10a** or **8**, 0.005 mmol of Pd_2_(dba)_3_·CHCl_3_, 0.015 mmol of **L**, 0.3 mmol of TEA and **9**, in THF at 0 °C for 2 h.

^*b*^Determined by HPLC.

^*c*^Determined by ^1^H NMR analysis.

^*d*^Allylation product with debenzylation of carboxylic acid.

^*e*^ ***ent*-L2** provided another diastereoisomer.

**Table 2:** Scope of the asymmetric Tsuji–Trost reaction^*a*^

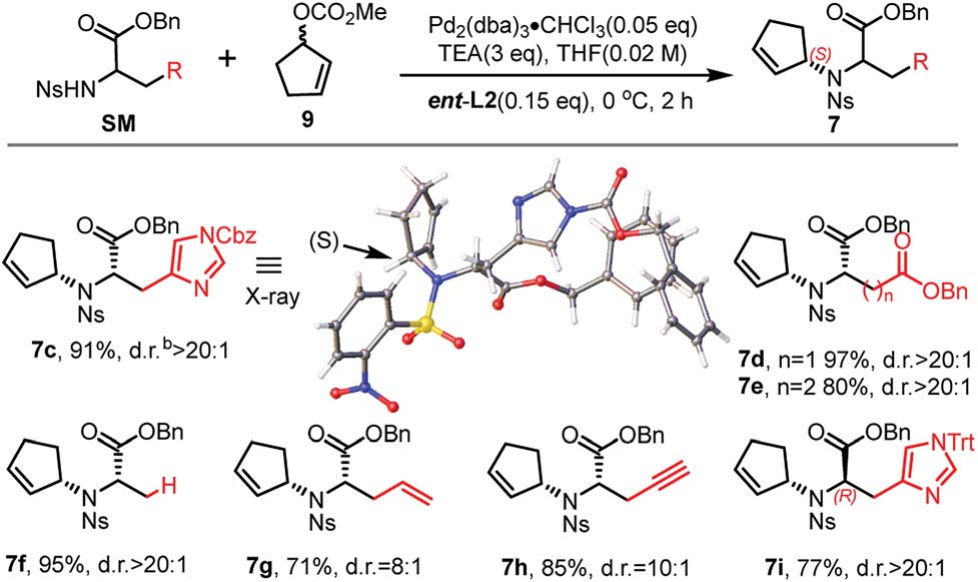

^*a*^Reaction conditions: 0.1 mmol of **SM**, 0.005 mmol of Pd_2_(dba)_3_·CHCl_3_, 0.015 mmol of **L**, 0.3 mmol of TEA and **9**, in THF at 0 °C for 2 h.

^*b*^Determined by ^1^H NMR analysis.

**Scheme 1:**
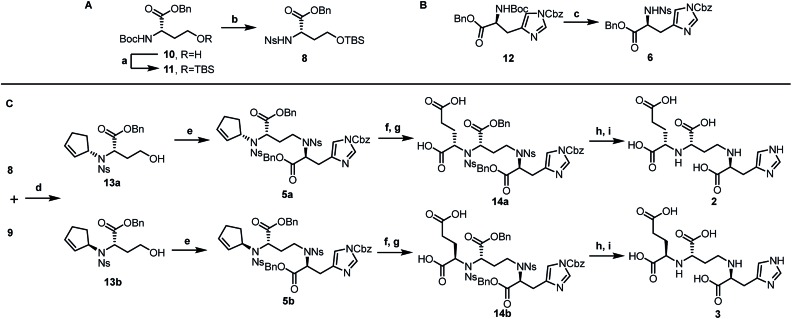
(A) Synthesis of l-homoserine derivative (**8**). (B) Synthesis of l-histidine derivative (**6**). (C) Total syntheses of pseudopaline (**2**) and *epi*-pseudopaline (**3**). (a) TBSCl (1.25 eq.), imidazole (1.5 eq.), DCM (0.2 M), 0 °C, 1 h, 93%; (b) TMSOTf (8 eq.), 2,6-lutidine (10 eq.), DCM (0.2 M), 0 °C-r.t., 2 h then *o*-NsCl (1.2 eq.), NaHCO_3_ (5 eq.), EtOAc/H_2_O (1 : 1, 0.2 M), r.t., 12 h, 72%; (c) TFA/DCM (1 : 9, 0.3 M), 0 °C-r.t., 1 h then *o*-NsCl (1.1 eq.), NaHCO_3_ (4 eq.), EtOAc/H_2_O (1 : 1, 0.2 M), r.t., 12 h, 87%; (d) **9** (3eq.), Pd_2_(dba)_3_-CHCl_3_ (0.05 eq.), ***ent*-L2** (0.15 eq.), TEA (3 eq.), THF (0.1 M), 0 °C, 2 h; then HF·Py (4 eq.), 0 °C-r.t., 10 h, 94% for **13a**; **L2** afford **13b**, 87%; (e) **6** (0.7 eq.), Ph_2_P–Py (1.5 eq.), DEAD (1.5 eq.), THF (0.1 M), 0 °C-r.t., 2 h, 82% for **5a**, 80% for **5b**; (f) OsO_4_ (0.2 eq.), NaIO_4_ (10 eq.), dioxane/H_2_O (2 : 1, 0.01 M) 2,6-lutidine (4 eq.), r.t., 3 h; (g) Jones reagent (2.2 eq.), acetone (0.02 M), 0 °C-r.t., 16 h; 58% for **14a**, 2 steps, 55% for **14b**, 2 steps; (h) TfOH (10 eq.), anisole (11 eq.), DCM (0.01 M), 0 °C, 0.5 h; r.t., 1.5 h; (i) PhSH (20 eq.), K_2_CO_3_ (8 eq.), DMF (0.02 M), r.t., 14 h; 74% for **2**, 2 steps, 80% for **3**, 2 steps. DEAD = Diethyl azodicarboxylate.

After establishing the key asymmetric Tsuji–Trost reaction conditions, we continued the total synthesis of pseudopaline and its isomer (Scheme 1). The yield of the initial route to the Tsuji–Trost precursor **8** was only moderate due to the formation of a homoserine γ-lactone byproduct under TFA or other acidic conditions (Scheme S1†). Therefore, l-homoserine **10** was first protected with TBSCl, and then the Boc group was selectively deprotected with TMSOTf followed by *o*-nosyl protection to afford compound **8** in 72% yield (Scheme 1A). In addition, we have optimized the route to generate the L-His fragment **6**. The Cbz protected L-His **12**^18^ was produced efficiently on a large scale and purified by recrystallization. The Boc group was deprotected smoothly with TFA to yield free amine followed by protection with an *o*-nosyl group to afford the key precursor **6** in excellent yield (Scheme 1B). Applying the optimized asymmetric Tsuji–Trost reaction conditions on compounds **8** and **9**, followed by deprotection of the TBS group, enabled the smooth generation of product **13a** (Scheme 1C). The second C–N bond formation between **13a** and the protected L-His **13** was realized through the Mitsunobu reaction to afford the desired product **5a** with the pseudopaline skeleton. However, the initial attempts for the one-step conversion to di-acid **14a** with ruthenium trichloride19*a* or ozonolysis19*b* only gave complex mixtures. Gratifyingly, Lemieux–Johnson oxidation of **5a** afforded dialdehyde,19*c* which could be further oxidized to generate di-acid **14a** without purification using the Jones reagent.16*a*

At this stage, deprotection of **14a** would complete the synthesis of pseudopaline (**2**). From our previous syntheses of aspergillomarasmine A16*a* and staphylopine,16*b* we had utilized a global deprotection protocol.^20^ As expected, the benzyl and Cbz groups of di-acid **14a** were deprotected smoothly to give di-Ns pseudopaline. However, the *o*-nosyl group deprotection with ethyldiisopropylamine did not go to completion even after 2 days. To our delight, the replacement of ethyldiisopropylamine with potassium carbonate could smoothly afford pure pseudopaline (**2**) without any racemization in 8 steps with an overall 22% yield starting from the protected l-homoserine **10**. As the stereochemistry of the glutaric acid moiety in natural pseudopaline (**2**) is unclear, it is essential to synthesize the *R*-glutaric acid moiety of pseudopaline for comparison. Accordingly, the chemical synthesis of *epi*-pseudopaline (**3**) was also accomplished using a previously described synthetic route (Scheme 1C). Direct comparison with natural pseudopaline is the most convincing way to elucidate the correct structure.^21^ However, due to the extremely high polarity of pseudopaline (**5**), the direct isolation turned out to be very challenging. Then we decided to *in situ* generate Fmoc-pseudopaline using *N*-(9-fluorenylmethoxycarbonyloxy)succinimide (Fmoc-NHS) in order to improve the detection sensitivity and decrease the polarity of pseudopaline.^22^ Starting from 4.2 liters of VBMM culture medium cultivated with the wild type *P. aeruginosa* PAO1, 1.1 mg of natural Fmoc-pseudopaline was obtained (Scheme S3†).^23^ In addition, we have prepared the Fmoc derivative of synthetic pseudopaline for data comparison (Scheme S2 and S4†). Through extensive data comparison, Fmoc-pseudopaline from synthetic and natural sources exhibited indistinguishable NMR spectra and optical rotation values. Furthermore, after mixing our synthetic Fmoc-pseudopaline with natural Fmoc-pseudopaline (in a 1 : 1 ratio), the ^1^H NMR spectrum showed only one set of peaks (see the ESI† for details). Collectively, these results unambiguously confirmed the chemical structure of pseudopaline (**2**), and the experimentally determined structure matched the structures proposed in the literature.^9^

**Fig. 2:**
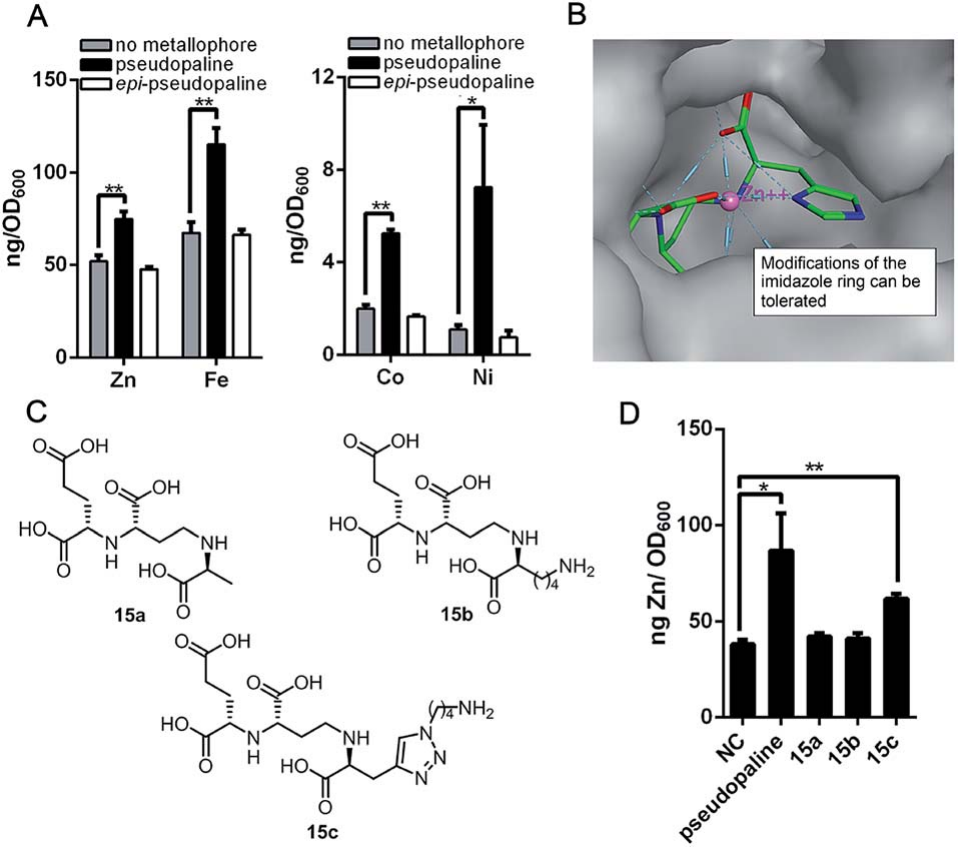
The metal import by pseudopaline and *epi*-pseudopaline in *P. aeruginosa* in the mineral scarce medium and the design of three pseudopaline analogs **15a–c**. (A) Intracellular zinc, nickel, cobalt and iron contents of wild type *P. aeruginosa* grown in VBMM containing the corresponding metal and 50 μM EDTA and treated with and without 100 μM pseudopaline or *epi*-pseudopaline measured by ICP-MS from three independent trials. (B) The co-crystal structure of the CntA/staphylopine/Zn^2+^ complex.^24^ (C) The chemical structures of the three pseudopaline analogs **15a–c**. (D) Intracellular zinc contents of wild type *P. aeruginosa* grown in VBMM containing 10 μM and 50 μM EDTA and treated with and without 100 μM pseudopaline, **15a**, **15b** or **15c**, measured by ICP-MS from three independent trials. Error bars, mean ± SD. *P < 0.05 and **P < 0.01.

As the pseudopaline operon was discovered in the zinc-deficient environment and subsequently was found involved in zinc uptake,9a,^10^,^12^ the role of pseudopaline (**2**) for zinc import in *P. aeruginosa* was evaluated. The intracellular zinc concentration with and without pseudopaline or *epi*-pseudopaline (**3**) was measured in a metal chelated VBMM medium (supplied with EDTA).^10^ The intracellular concentration of zinc significantly increased when supplied with pseudopaline (**2**) (Fig. 2A). The effect of pseudopaline in transporting other transition metals was also investigated. The intracellular contents of nickel, cobalt and iron significantly increased when supplied with pseudopaline (**2**) (Fig. 2A). These results were consistent with previous studies that this operon (PA4834–PA4837, *zrmABCD*) was involved in zinc, nickel, cobalt and iron uptake.9a,^10^,14a No significant alterations in the intracellular contents of copper, manganese, magnesium or calcium were observed (Figure S1†). However, *epi*-pseudopaline showed no effect on metal import (Fig. 2A). Further experiments revealed that in the metal-rich VBMM medium (without EDTA) or nutrient-rich (LB) medium, pseudopaline (**2**) could not promote metal uptake (Figure S2†). The results suggest that pseudopaline (**2**) mediates metal uptake especially in the nutrient-depleted environment, and the *S*-configuration of pseudopaline (**2**) is necessary for this activity.

According to the recently reported co-crystal structure of staphylopine (staphylopine and pseudopaline share the aminobutyrate and histidine moieties while pseudopaline differs from staphylopine with the stereochemistry of its histidine moiety associated with an alpha ketoglutarate moiety instead of pyruvate) and its transporting protein CntA,^24^ we postulated that the imidazole ring is essential for metal chelation but at the same time could be modified without interfering with the metal-chelating and protein-binding activities. As shown in Fig. 2B, the co-crystal structure indicates that the imidazole ring forms a coordinate bond with the metal and meanwhile it is the only moiety of the natural product that points toward the outside of the protein-binding pocket. Based on this information, we rationally designed and applied our established synthetic route to prepare three pseudopaline analogs **15a–c** (Fig. 2C) to examine whether the imidazole ring on pseudopaline is indispensable for metal binding and metal uptake capability (Scheme S5,† the structure of **20b** was confirmed by X-ray crystallography). The ability of zinc transport was used to evaluate the activity of the pseudopaline analogs (Fig. 2D). Although the average intracellular zinc content of the bacteria treated with **15c** was 28% lower than that treated with pseudopaline (**2**), the difference was not statistically significant (*P* > 0.05), indicating that the change of the original imidazole ring to the substituted triazole ring could preserve the activity of zinc transport. However, the other two analogs **15a** and **15b** without the chelation moiety showed much reduced activities. To measure the metal binding affinity of pseudopaline (**2**), *epi*-pseudopaline (**3**) and the positive analog **15c**, the stability constants for pseudopaline–metal complexes were estimated by metal competition experiments using the ESI-MS method.8a,16b,^25^ Interestingly, all the tested compounds showed strong coordination properties toward bivalent metal ions as shown by the complex dissociation constants (Table 3). Both pseudopaline (**2**) and *epi*-pseudopaline (**3**) show higher or comparable metal binding affinity to staphylopine (**1**), which suggests that the carboxyl group of glutamate side chain does not participate in the metal coordination and therefore the configuration of the glutaric acid moiety does not affect metal binding. Accordingly, we propose that the configuration of the glutaric acid moiety is important for forming the desired interactions in the protein-binding pocket, as *epi*-pseudopaline cannot promote metal transport into the bacteria (Fig. 2A). To our delight, the pseudopaline analog (**15c**) showed comparable binding affinity to the parent natural product pseudopaline (**2**), together with its equivalent biological activity to pseudopaline (**2**), which suggests that functionalized **15c** will be a promising candidate for the antibiotic conjugate.

**Table 3:** The putative metal-chelation structures and the estimated p*K*_d_ values in comparison with those of staphylopine^*a*^

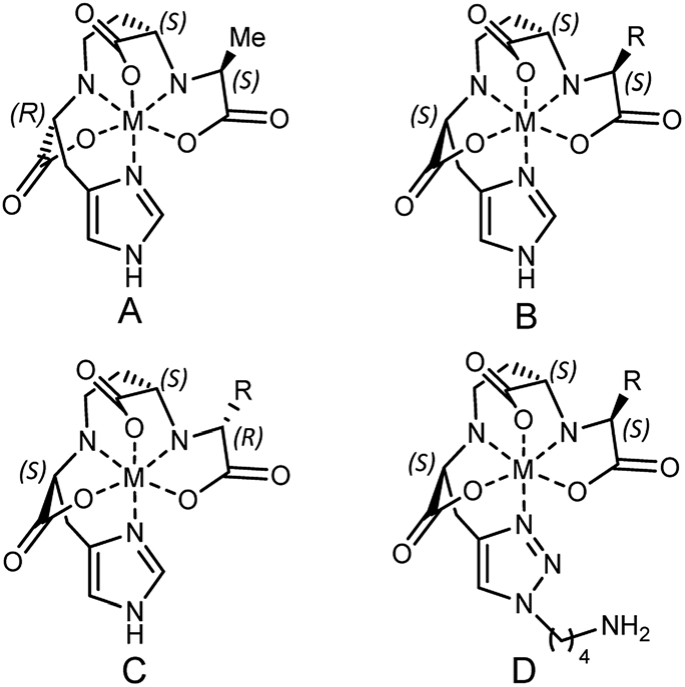				
Metal ions	p*K*_d_			
	Staphylopine8*a*	Pseudopaline	*epi*-Pseudopaline	**15c**
Cu^2+^	19.0	19.4	19.6	19.3
Ni^2+^	16.4	16.6	16.6	17.0
Co^2+^	15.1	16.1	14.9	15.7
Zn^2+^	15.0	15.8	14.7	15.5
Fe^2+^	12.3	13.1	12.9	13.3
Mn^2+^	9.1	10.4	9.3	9.7

^*a*^The putative chemical structure of the staphylopine–metal complex (A), pseudopaline–metal complex (B), *epi*-pseudopaline–metal complex (C) and **15c**–metal complex (D). R = (CH_2_)_2_COOH. The estimated p*K*_d_ values for staphylopine (**1**), pseudopaline (**2**), *epi*-pseudopaline (**3**) and the pseudopaline analog (**15c**) using the competition experiments with six different metal species by ESI-MS.8*a* p*K*_d_ represents the dissociation equilibrium constant of coordination complexes.

**Fig. 3:**
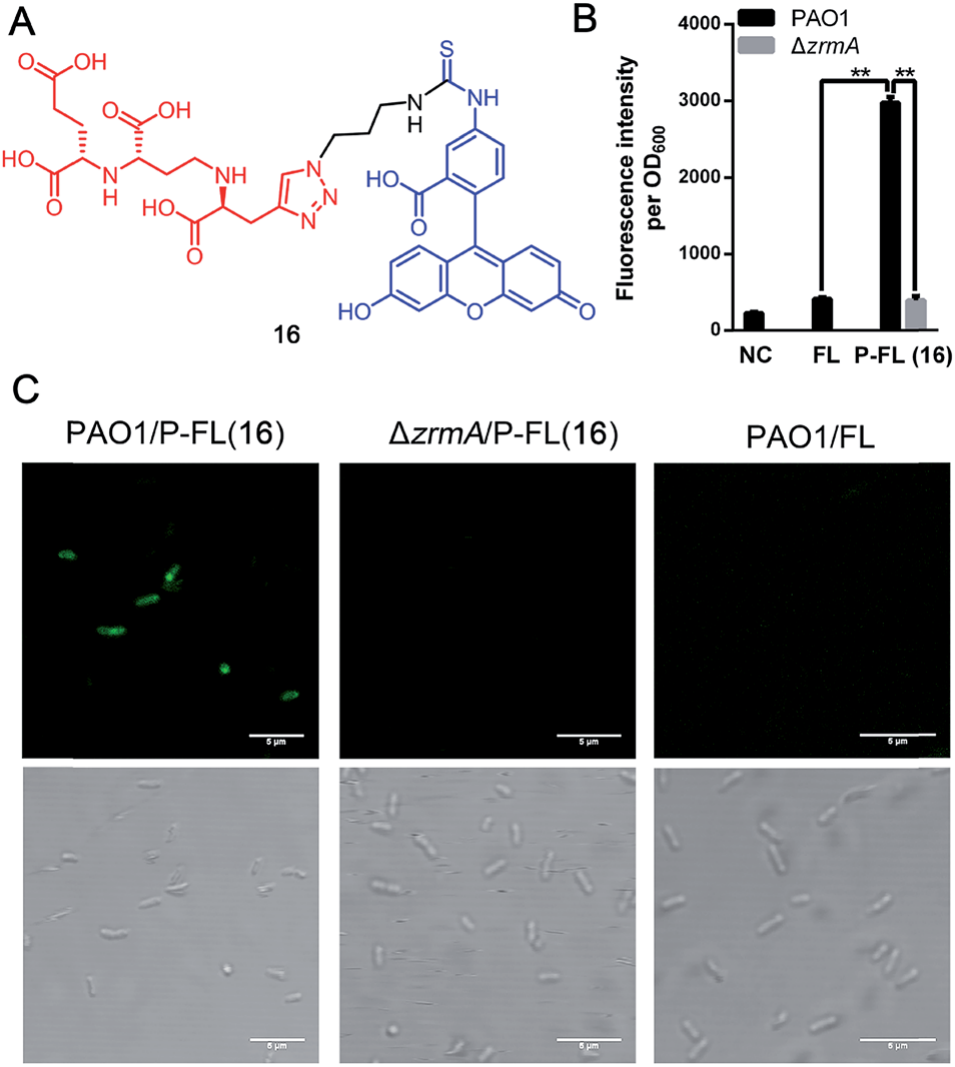
Pseudopaline-fluorescein conjugate (P-FL **16**) could be transported in *P. aeruginosa*. (A) The chemical structure of pseudopaline-fluorescein (P-FL **16**). (B) The fluorescence intensity of wild type *P. aeruginosa* PAO1 treated with fluorescein (5-carboxyfluorescein, FL) or pseudopaline-fluorescein (P-FL **16**), and pseudopaline receptor knock-out strain Δ*zrmA* treated with pseudopaline-fluorescein (P-FL **16**) and detected by using a microplate reader. NC, negative control, PAO1 treated without FL. (C) Fluorescence microscopy images of PAO1 and Δ*zrmA* treated with pseudopaline-fluorescein (P-FL **16**), and PAO1 treated with fluorescein (FL). The top pictures were taken at: ex. 493 ± 20 nm/em. 516 ± 20 nm. The bottom pictures were taken by light microscopy (bright phase). Scale bar: 5 μm. Error bars, mean ± SD. **P < 0.01.

Fluorescent siderophores have been widely used as invaluable tools to investigate the molecular mechanisms and to facilitate the diagnosis of bacterial infections.^26^ To confirm the potential uptake of pseudopaline itself or pseudopaline analog **15c** by *P. aeruginosa*, a pseudopaline-fluorescein probe **16** (Fig. 3A, P-FL **16**) was designed and synthesized (Scheme S6†). The fluorescence intensity of *P. aeruginosa* treated with P-FL **16** in the metal-limited medium was further investigated. When the wild type *P. aeruginosa* PAO1 was incubated with P-FL **16**, the fluorescence signal was clearly observed and the bacterial cell was stained (Fig. 3B, C and S3†). In contrast, when the pseudopaline outer membrane receptor knock-out strain Δ*zrmA*^11^ was treated with P-FL **16**, the bacteria did not exhibit a detectable fluorescence signal (Fig. 3B, C and S3†). Meanwhile, when PAO1 was incubated with the fluorescein (5-carboxyfluorescein, FL) alone under the same conditions, no significant fluorescence signal was detected either. Collectively, these data have revealed that the pseudopaline component of the probe is indispensable for the fluorescence enhancement, and illustrated that P-FL can be transported in the bacteria, and further confirmed that ZrmA is the pseudopaline receptor.

## Conclusions

In summary, we have accomplished the first *de novo* total synthesis of pseudopaline (**2**), a metallophore produced by *P. aeruginosa*, in 8 steps from the known l-homoserine **10**. The key synthetic strategy relies on the asymmetric Tsuji–Trost reaction and Mitsunobu reaction to form the challenging C–N bonds. The total synthesis along with the natural product isolation allowed data comparison, which led to the unambiguous structural assignment of pseudopaline. The synthetic pseudopaline (**2**) has been shown to transport metals such as zinc, cobalt, iron, and nickel, but not copper, manganese, calcium or magnesium. The total synthesis also enabled the preparation of the inactive *epi*-pseudopaline (**3**), which demonstrated the importance of the (*S*)-configuration for metallophore function. The preliminary structure–activity relationship study indicated that pseudopaline derivative **15c** shows comparable activity to pseudopaline. Moreover, a pseudopaline-fluorescein conjugate P-FL **16** was prepared and evaluated. The results showed that P-FL **16** could be transported in *P. aeruginosa*. Our study demonstrates the proof-of-concept that pseudopaline could be used to develop a new “Trojan horse” antibiotic conjugate. Collectively, this work provides a solid foundation for further chemical and biological investigations to uncover the new mechanisms about how this novel metallophore transports metal ions for bacteria as well as a guide to develop new effective antibiotics against Gram-negative bacterial infections.

## Conflicts of interest

There are no conflicts to declare.

## Supplementary Material

Supplementary information

Crystal structure data
