# Supplementary material for: De novo synthesis, structural assignment and biological evaluation of pseudopaline, a metallophore produced by Pseudomonas aeruginosa
Source: Chem Sci. 2019 May 30;10(27):6635–41. doi: 10.1039/c9sc01405e (PMC6625496; doi:10.1039/c9sc01405e)
Supplement: Supplementary file 1 [file SC-010-C9SC01405E-s001.pdf]

Electronic Supplementary Material (ESI) for Chemical Science.
This journal is © The Royal Society of Chemistry 2019

Supporting Information

De Novo Synthesis, Structural Assignment and Biological Evaluation of Pseudopaline, a Metallophore Produced by *Pseudomonas aeruginosa*

Jian Zhang,[1,3] Tianhu Zhao,[1,3] Rongwen Yang, [1,3] Ittipon Siridechakorn,[1] Sanshan Wang,[1] Qianqian Guo,[1] Yingjie Bai,[1] Hong C. Shen,[2] Xiaoguang Lei[1]*

[1]Department of Chemical Biology, College of Chemistry and Molecular Engineering, Beijing National Laboratory for Molecular Sciences, Key Laboratory of Bioorganic Chemistry and Molecular Engineering of Ministry of Education, Synthetic and Functional Biomolecules Center and Peking-Tsinghua Center for Life Sciences, Peking University, Beijing 100871, China, [2] Roche Innovation Center Shanghai, Roche Pharma Research & Early Development, Shanghai 201203, China, [3]These authors contributed equally, *Correspondence: xglei@pku.edu.cn

Table of Contents

**I) General Information**	S3
**II) Detailed synthetic procedures**	S4-S18
**III) Comparison of the synthetic and natural Fmoc-pseudopaline**	S19-S22
**IV) Biological evaluations**	S23-S24
**V) Metal chelation experiments**	S25-S29
**VI) NMR spectra**	S30-S73
**VII) HRMS data for the metal chelation experiments**	S74-S91
**VIII) X-ray crystal structures**	S92-S95
**IX) References**	S96

I) General Information

^{1}H NMR spectra were recorded on a Bruker 400 MHz spectrometer at ambient temperature with $CDCl_3$ as the solvent unless otherwise stated. ^{13}C NMR spectra were recorded on a Bruker 101 MHz or 126 MHz spectrometer (with complete proton decoupling) at ambient temperature. ^{13}C NMR spectra of synthetic Fmoc-pseudopaline and natural Fmoc-pseudopaline were recorded on a Bruker AVANCE 201 MHz spectrometer (with complete proton decoupling) at ambient temperature. ^{13}C NMR spectrum of compound **16** was recorded on a Bruker AVANCE 239 MHz spectrometer (with complete proton decoupling) at ambient temperature. Chemical shifts are reported in parts per million relative to chloroform (^{1}H, δ 7.26 ppm; ^{13}C, δ 77.00 ppm) or deuterium oxide (^{1}H, δ 4.79 ppm). Data for ^{1}H NMR is reported as follow: chemical shift, integration, multiplicity (s = singlet, d = doublet, t = triplet, q = quartet, m = multiplet) and coupling constants. IR spectra were recorded on a Thermo Fisher FT–IR200 spectrophotometer. High–resolution mass spectra were obtained at Peking University Mass Spectrometry Laboratory using a Bruker Fourier Transform Ion Cyclotron Resonance Mass Spectrometer Solarix XR. Optical rotations were recorded on an AUTOPOL III digital polarimeter at 589 nm as $[\alpha]_D^{20}$ (concentration in grams/100 mL solvent). The samples were analyzed by HPLC/MS on a Waters Auto Purification LC/MS system (3100 Mass Detector, 2545 Binary Gradient Module, 2767 Sample Manager, and 2998 Photodiode Array (PDA) Detector). The system was equipped with a Waters C18 5 *μ*m SunFire separation column (150*4.6 mm), equilibrated with HPLC grade water (solvent A) and HPLC grade methanol (solvent B) with a flow rate of 0.3 mL/min at rt. Preparative HPLC–MS on a Waters Auto Purification LC/MS system (3100 Mass Detector, 2545 Binary Gradient Module, 515 HPLC pump, 2767 Sample Manager, and 2998 Photodiode Array (PDA) Detector). The system was equipped with a Waters C18 5*μ*m X–bridge separation column (150*19 mm). Analytical thin layer chromatography was performed using 0.25 mm silica gel 60–F plates. Flash chromatography was performed using 200–400 mesh silica gel. Yields refer to chromatographically and spectroscopically pure materials, unless otherwise stated. All reagents were used as supplied by Sigma–Aldrich, J&K and Alfa Aesar Chemicals. All reactions were carried out in oven–dried glassware under an argon atmosphere unless otherwise noted.

II) Detailed Experimental Section

Syntheses of the building blocks

Compound **12**

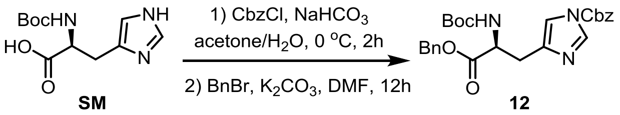

To a stirred solution of **SM** (15 g, 59 mmol, 1 eq) in H_2O (100 mL) and acetone (125 mL) at 0 °C was added $NaHCO_3$ (12.9 g, 153 mmol, 2.6 eq) and CbzCl (10 mL, 71 mmol, 1.2 eq). After stirring for 2 h at 0 °C, acetone was removed *in vacuo,* then added sat. $KHSO_4$ to pH=1-2. The water phase was extracted by EA (100 mL x 2). The combined organic phases were dried over Na_2SO_4 and concentrated *in vacuo*. To the residue was added DMF (100 mL), K_2CO_3 (12.2 g, 88 mmol, 1.5 eq) and BnBr (7.4 mL, 62 mmol, 1.05 eq), and the resulting mixture was stirred for 12 h. then added EA (300 mL) and H_2O (100 mL), the organic phase was washed with brine (50 mL x 3) and dried over Na_2SO_4. The solvent was evaporated *in vacuo,* and the crude product was purified by recrystallization from hexane: DCM to afford the product **12** as a white solid (21.2 g, 44.3 mmol, 75%).

Compound **6**

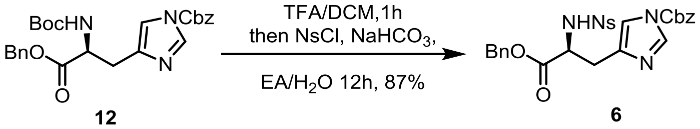

To a stirred solution of compound **12** (21.2 g, 44.3 mmol, 1 eq) in DCM (132 mL) at 0 °C was added TFA (14.7 mL). After stirring for 1 h at room temperature, the solvent was removed *in vacuo.* To the residue was added EA (110 mL), NsCl (10.8 g, 48.7 mmol, 1.1 eq) and a solution of $NaHCO_3$ (14.9 g, 177.2 mmol, 4 eq) in H_2O (110 mL), and the resulting mixture was stirred for 12 h. The organic phase was washed with aq. $NaHCO_3$ (100 mL x 2), brine (100 mL x 1) and dried over Na_2SO_4. The solvent was evaporated *in vacuo,* and the crude product was purified by recrystallization from hexane: DCM to afford the product as a white solid (21.7 g, 38.5 mmol, 87%). ^{1}H NMR (400 MHz, $CDCl_3$) δ 8.00 – 7.96 (m, 2H), 7.75 – 7.66 (m, 1H), 7.60 – 7.52 (m, 2H), 7.46 – 7.38 (m, 5H), 7.25 – 7.19 (m, 3H), 7.12 – 7.09 (m, 3H), 7.05 (s, 1H), 5.38 (s, 2H), 4.93 (d, *J* = 12.0 Hz, 1H), 4.82 (d, *J* = 12.0 Hz, 1H), 4.65 – 4.61 (m, 1H), 3.12 – 3.09 (m, 2H); ^{13}C NMR (101 MHz, $CDCl_3$) δ 170.1, 148.2, 147.4, 138.1, 137.0, 134.9, 133.9, 133.1, 132.6, 130.3, 129.3, 128.9, 128.9, 128.4, 128.3, 125.3, 114.9, 69.9, 67.2, 56.2, 31.0; IR (neat) ν_{max} 1683, 1515, 1387, 1362, 1319, 1148, 928 cm^{-1}; HRMS (ESI): $[M+H]^+$ calculated for $C_{27}H_{25}N_4O_8S$: 565.1388, found: 565.1381; $[\alpha]_D^{23}$ –88.6 (*c* 0.5, $CHCl_3$); m.p. = 125-130℃; R_f = 0.52 (DCM/MeOH = 40/1 with 1/1000 ammonium hydroxide).

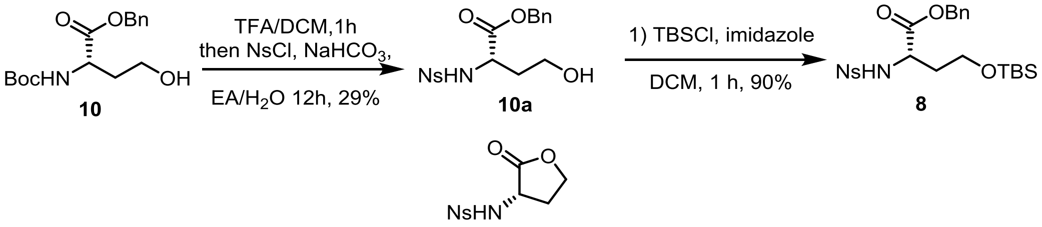

homoserine Υ-lactone byproduct

Scheme S1: Initial Synthesis of the Homoserine Building Block **8**

Compound **10a**

To a stirred solution of **10** (3.26 g, 10.5 mmol, 1 eq) in DCM (24 mL) at 0 °C was added TFA (6 mL). After stirred for 1 h at r.t., the solvent was removed *in vacuo.* To the residue was added EA (20 mL), NsCl (2.35 g, 10.6 mmol, 1.01 eq) and saturated $NaHCO_3$ (30 mL), and the resulting mixture was stirred for 12 h. The organic phase was washed with aq. $NaHCO_3$ (20 mL x 2), brine (10 mL x 1) and dried over Na_2SO_4. The solvent was evaporated *in vacuo,* and the crude product was purified by silica gel column chromatography (PE/EA=10:1 to 8:1) to afford product **10a** (1.19 g, 3.05 mmol, 29%) as white oil. ^{1}H NMR (400 MHz, $CDCl_3$) δ 8.02 – 7.99 (m, 1H), 7.82 – 7.80 (m, 1H), 7.64 – 7.59 (m, 2H), 7.32 – 7.29 (m, 3H), 7.17 – 7.15 (m, 2H), 6.54 (d, *J* = 8.8 Hz, 1H), 4.96 (d, *J* = 12.1 Hz, 1H), 4.91 (d, *J* = 12.1 Hz, 1H), 4.43 (td, *J* = 8.7, 4.3 Hz, 1H), 3.85 – 3.79 (m, 2H), 2.20 – 2.13 (m, 1H), 1.99 – 1.88 (m, 2H); ^{13}C NMR (101 MHz, $CDCl_3$) δ 171.0, 147.4, 134.7, 133.8, 133.5, 132.8, 130.3, 128.5, 128.5, 128.3, 125.6, 67.4, 58.2, 54.2, 35.0; IR (neat) ν_{max} 3329, 2925, 1741, 1540, 1356, 1167, 1119 cm^{-1}; HRMS (ESI): $[M+H]^+$ calculated for $C_{17}H_{19}N_2O_7S$: 395.0906, found: 395.0907; $[\alpha]_D^{24}$ –173 (*c* 1, $CHCl_3$); R_f = 0.22 (PE/EA = 2/1).

Compound **8**

Compound **10a** (1.19 g, 3.02 mmol, 1.0 eq) was dissolved in DCM (10 mL) and to this solution was added imidazole (308 mg, 4.53 mmol, 1.5 eq) and TBSCl (569 mg, 3.78 mmol, 1.25 eq) at 0 °C under argon. The mixture was warmed to r.t. and stirred for 1 h. The reaction was quenched by water (10 mL) at 0 °C, and the water phase was extracted by DCM (10 mL x 3). The combined organic

phases were dried over Na_2SO_4 and concentrated *in vacuo*. The residue was purified by silica gel column chromatography (PE/EA = 20/1) to afford the desired product **8** (1.38 g, 2.72 mmol, 90%) as a colorless oil. ^{1}H NMR (400 MHz, $CDCl_3$) δ 7.98 (d, *J* = 7.6 Hz, 1H), 7.78 (d, *J* = 7.6 Hz, 1H), 7.62 – 7.57 (m, 2H), 7.32 – 7.30 (m, 3H), 7.22 – 7.13 (m, 2H), 6.48 (d, *J* = 8.8 Hz, 1H), 4.95 (d, *J* = 12.0 Hz, 1H), 4.86 (d, *J* = 12.0 Hz, 1H), 4.47 – 4.42 (m, 1H), 3.77 – 3.70 (m, 2H), 2.14 – 1.99 (m, 2H), 0.90 (s, 9H), 0.06 (s, 6H);
^{13}C NMR (101 MHz, $CDCl_3$) δ 170.9, 147.5, 134. 9, 134.4, 133.3, 132.6, 130.3, 128.5, 128.4, 128.2, 125.4, 67.1, 59.0, 54.7, 35.2, 25.9, 18.3, –5.6; IR (neat) v_{max} 3329, 2957, 2857, 1745, 1542, 1368, 1172, 1122, 837 cm^{-1}; HRMS (ESI): $[M+H]^+$ calculated for $C_{23}H_{32}N_2O_7SSi$: 509.1772, found: 509.1768; $[\alpha]_D^{24}$ –110 (*c* 1, $CHCl_3$); R_f = 0.67 (PE/EA = 2/1).

Compound **11**

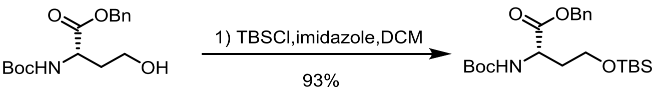

10 **11**

Compound **10** (7.8 g, 25.2 mmol, 1.0 eq) was dissolved in DCM (126 mL) and to this solution was added imidazole (2.58 g, 37.8 mmol, 1.5 eq) and TBSCl (4.76 g, 31.6 mmol, 1.25 eq) at 0 ℃ under argon. The mixture was stirred for 1 h at 0 ℃. Then the reaction was quenched by water (50 mL) at 0 ℃, the water phase was extracted by DCM (100 mL x 3). The combined organic phases were dried and concentrated *in vacuo*. The residue was further purified by silica gel column chromatography (PE/EA = 20/1) to afford the desired product **11** (9.1 g, 23.4 mmol, 93%) as a colorless oil. ^{1}H NMR (400 MHz, $CDCl_3$) δ 7.35 – 7.29 (m, 5H), 5.76 (d, *J* = 7.5 Hz, 1H), 5.21 (d, *J* = 12.4 Hz, 1H), 5.11 (d, *J* = 12.4 Hz, 1H), 4.42 (dd, *J* = 11.6, 7.0 Hz, 1H), 3.71 – 3.61 (m, 2H), 2.11 – 1.98 (m, 1H), 1.94 (dd, *J* = 12.9, 7.4 Hz, 1H), 1.42 (s, 9H), 0.88 (s, 9H), 0.02 (s, 6H); ^{13}C NMR (101 MHz, $CDCl_3$) δ 172.4, 155.6, 135.7, 128.5, 128.3, 128.2, 79.5, 66.8, 60.2, 52.6, 33.7, 28.3, 25.9, 25.7, 18.1, -5.6, -5.7; IR (neat) v_{max} 2916, 1723, 1496, 1460, 1378, 1159, 836, 730 cm^{-1}; HRMS (ESI): $[M+H]^+$ calculated for $C_{22}H_{38}NO_5Si$: 424.2514 found: 424.2528; $[\alpha]_D^{21}$ -25.4 (*c* 1, $CHCl_3$); R_f = 0.80 (PE/EA = 3/1).

Compound **8**

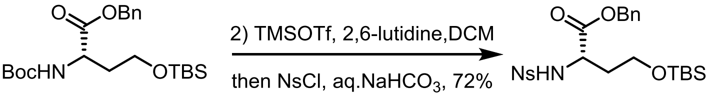

11 **8**

TMSOTf (18.8 mL, 98.7 mmol, 8.0 eq) was added to a stirring solution of compound **11** (5.23 g, 12.3 mmol, 1.0 eq) in DCM (61.5 mL) in the presence of 2,6-lutidine (14.3 mL, 123.0 mmol, 10.0 eq) at r.t.. After 1.5 h, MeOH (20 mL) was added to the reaction mixture at 0 °C, then warmed to r.t. and stirred for 2 h. The reaction mixture was concentrated *in vacuo* to afford colorless oil, which was immediately used for the next reaction without further purification. NsCl (3.27 g, 14.8 mmol, 1.2 eq) was added in one portion to a stirring EA (30 mL) solution of the crude amine at r.t.. A solution of $NaHCO_3$ (5.46 g, 65 mmol, 5.0 eq,) in 31.5 mL H_2O was added to the mixture. After stirring for 12 h, the mixture was diluted with EA (100 mL), and the water layer was separated. The organic layer was concentrated *in vacuo* to afford a residue, which was purified by silica gel column chromatography (PE/EA 30/1) to afford compound **8** (4.50 g, 8.9 mmol, 72%) as a colorless oil.

The Asymmetric Tsuji-Trost reaction
General procedure:

Preparation of these substrates: The amino group was firstly protected by NsCl using EA and aq. $NaHCO_3$ as co-solvent. The carboxylic acid was protected by BnBr using $NaHCO_3$ as base and DMF as solvent.

For the asymmetric Tsuji-Trost reaction: to a solution of $Pd_2(dba)_3$•$CHCl_3$(5.2 mg, 0.005 mmol, 0.05 eq) and Trost-ligand (11.9 mg, 0.015 mmol, 0.15 eq) in THF (1 mL) was added compound **9** (42.6 mg, 0.3 mmol, 3 eq) and TEA (30.3 mg, 0.3 mmol, 3 eq) at 0 °C. Then a solution of **SM** (0.1 mmol, 1 eq) in THF (1 mL) was added into the mixture. The reaction was monitored by TLC. The reaction mixture was concentrated *in vacuo* and the residue was purified by silica gel column chromatography (PE/EA=10/1 to 8/1) to afford the desired product.

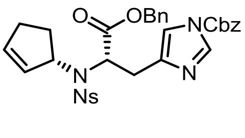

Compound **7c,** 91% yield, d.r.>20:1, white solid

Single crystals with X-ray diffraction quality were grown by dissolving the material in DCM and n-hexane. The mixture was lightly capped and the solvents were allowed to slowly evaporate.

^{1}H NMR (400 MHz, $CDCl_3$) δ 8.06 (s, 1H), 8.03 (dd, *J* = 8.0, 1.3 Hz, 1H), 7.58 – 7.45 (m, 3H), 7.44 – 7.41 (m, 5H), 7.31 – 7.28 (m, 3H), 7.26 – 7.23 (m, 2H), 7.18 (s, 1H), 5.96 (dd, *J* = 5.6, 2.4 Hz, 1H), 5.60 (dd, *J* = 5.6, 2.4 Hz, 1H), 5.40 (d, *J* = 11.6 Hz, 1H), 5.37 (d, *J* = 11.6 Hz, 1H), 5.11 (d, *J* = 12 Hz, 1H), 5.04 (d, *J* = 12 Hz, 1H), 4.90 – 4.85 (m, 1H), 4.66 (t, *J* = 6.8 Hz,1H), 3.59 (dd, *J* =14.8 Hz, 7.0 Hz, 1H), 3.16 (d, *J* = 14.8 Hz, 7.0 Hz, 1H), 2.61 – 2.55 (m, 1H), 2.30 – 2.07 (m, 2H), 1.72 - 1.67 (m, 1H); ^{13}C NMR (101 MHz, $CDCl_3$) δ 170.5, 148.6, 148.4, 139.8, 136.9, 136.6, 135.1, 134.6, 134.0, 133.2, 131.3, 130.8, 129.3, 129.2, 128.9, 128.8, 128.5, 128.4, 123.9,

115.1, 69.8, 67.6, 66.4, 58.4, 31.4, 31.2, 28.8; IR (neat) v_{max} 2958, 1757, 1544, 1405, 1243, 1213, 1012 cm^{-1}; HRMS (ESI): $[M+H]^+$ calculated for $C_{32}H_{31}N_4O_8S$: 631.1857, found: 631.1855; $[\alpha]_D^{22}$ –30.4 (*c* 1, $CHCl_3$); R_f =0.55(DCM/MeOH = 40/1 with 1/1000 ammonium hydroxide); m.p. =146-149°C.

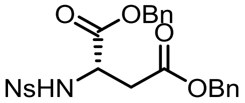

Substrate for Compound **7d**, white oil
^{1}H NMR (400 MHz, $CDCl_3$) δ 7.95 (dd, *J* = 7.7, 1.5 Hz, 1H), 7.79 (dd, *J* = 7.9, 1.5 Hz, 1H), 7.64 – 7.54 (m, 2H), 7.36 – 7.30 (m, 8H), 7.15 – 7.13 (m, 2H), 6.24 (d, *J* = 9.1 Hz, 1H), 5.13 (s, 2H), 4.94 – 4.83 (m, 2H), 4.31 (td, *J* = 9.1, 4.7 Hz, 1H), 2.64 – 2.50 (m, 2H), 2.32 – 2.24 (m, 1H), 2.08 – 1.92 (m, 1H); ^{13}C NMR (101 MHz, $CDCl_3$) δ 172.2, 170.4, 147.5, 135.7, 134.6, 133.8, 133.6, 132.8, 130.3, 128.6, 128.6, 128.6, 128.3, 128.3, 128.3, 125.6, 67.5, 66.6, 56.0, 29.7, 28.1; IR (neat) v_{max} 3358, 2954, 1740, 1539, 1355, 1169 cm^{-1}; HRMS (ESI): $[M+NH_4]^+$ calculated for $C_{24}H_{26}N_3O_8S$: 516.1435, found: 516.1449; $[\alpha]_D^{22}$ -132.0 (*c* 2, $CHCl_3$); R_f = 0.32 (PE/EA = 2/1).

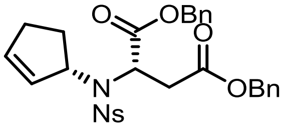

Compound **7d**, 97% yield, d.r.>20:1, colorless oil
^{1}H NMR (400 MHz, $CDCl_3$) δ 8.07 – 7.97 (m, 1H), 7.77 – 7.67 (m, 1H), 7.64 – 7.56 (m, 2H), 7.41 – 7.26 (m, 8H), 7.10 – 7.04 (m, 2H), 6.75 (d, *J* = 9.0 Hz, 1H), 5.10 (s, 2H), 4.87 (dd, *J* = 31.3, 12.1 Hz, 2H), 4.53 (dt, *J* = 9.0, 4.5 Hz, 1H), 3.18 (dd, *J* = 17.3, 4.4 Hz, 1H), 2.97 (dd, *J* = 17.3, 4.6 Hz, 1H); ^{13}C NMR (101 MHz, $CDCl_3$) δ 170.2, 169.6, 147.4, 135.0, 134.4, 134.3, 133.4, 132.7, 130.2, 128.6, 128.6, 128.5, 128.5, 128.5, 128.3,125.6, 67.9, 67.2, 53.2, 38.0; IR (neat) v_{max} 3033, 2917, 1734, 1543, 1341, 1160 cm^{-1}; HRMS (ESI): $[M+NH_4]^+$ calculated for $C_{29}H_{32}N_3O_8S$: 582.1904, found: 582.1907; $[\alpha]_D^{22}$ -75.9 (*c* 1, $CHCl_3$); R_f = 0.42 (PE/EA = 2/1).

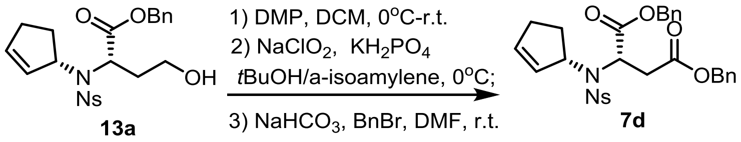

To a solution of the alcohol **13a** (28 mg, 0.06 mmol) in DCM (1mL) was added Dess-Martin reagent (38 mg, 0.09 mmol 1.5 eq) at 0 °C, then the reaction mixture was warmed to room temperature and stirred for 2.5 h, then cooled to 0 °C, and quenched with saturated $Na_2S_2O_3$ (1 mL) and saturated $NaHCO_3$ (1 mL). The resulting mixture was warmed up to room temperature again and stirred for an additional 1 h. The resulting solution was extracted with DCM (10 mL × 3), then dried over anhydrous Na_2SO_4 and the filtrate was concentrated *in vacuo* to afford the crude aldehyde which was directly used for the next step without further purification.
To a cooled (0 °C) stirred solution of the intermediate aldehyde in 5:1.2 *t*-BuOH/2-methyl-2-butene (1.4 mL) was added an ice-cold buffered 1.1 M aqueous solution of $NaClO_2$ (0.27 mL, 0.3 mmol 5 eq) dropwise. The reaction mixture was stirred for 3 h and then quenched with pH=2 KH_2PO_4-HCl buffer (1 mL). The mixture was extracted with EA (10 mL × 3) and dried ($MgSO_4$), filtered and evaporated under reduced pressure to give colorless oil. The product was used without further purification.
To the residue in DMF (1 mL) was added $NaHCO_3$ (15.1 mg, 0.18 mmol, 3 eq) and BnBr (7.8 *μ*L, 0.066 mmol, 1.1 eq), the mixture was stirred for 12 h at room temperature. The mixture was quenched with water (2 mL), and extracted with EA (10 mL x 2). The organic phase was washed with brine (2 mL x 2), dried with Na_2SO_4, and concentrated *in vacuo*. The residue was purified by silica gel column chromatography (PE/EA = 10/1) to afford the desired product **7d** (22 mg, 0.039 mmol, 65%) as colorless oil. ^{1}H NMR was provided.

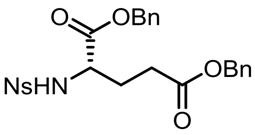

Substrate for Compound **7e,** white oil
^{1}H NMR (400 MHz, $CDCl_3$) δ 7.95 (dd, *J* = 7.7, 1.5 Hz, 1H), 7.79 (dd, *J* = 7.9, 1.5 Hz, 1H), 7.64 – 7.54 (m, 2H), 7.36 – 7.30 (m, 8H), 7.15 – 7.13 (m, 2H), 6.24 (d, *J* = 9.1 Hz, 1H), 5.13 (s, 2H), 4.94 – 4.83 (m, 2H), 4.31 (td, *J* = 9.1, 4.7 Hz, 1H), 2.64 – 2.50 (m, 2H), 2.32 – 2.24 (m, 1H), 2.08 – 1.92 (m, 1H); ^{13}C NMR (101 MHz, $CDCl_3$) δ 172.2, 170.4, 147.5, 135.7, 134.6, 133.8, 133.6, 132.8, 130.3, 128.6, 128.6, 128.6, 128.3, 128.3, 128.3, 125.6, 67.5, 66.6, 56.0, 29.7, 28.1; IR (neat) v_{max} 3319, 2955, 1735, 1540, 1355, 1169, 970 cm^{-1}; HRMS (ESI): $[M+NH_4]^+$ calculated for $C_{25}H_{28}N_3O_8S$: 530.1591, found: 530.1592; $[\alpha]_D^{22}$ –141.2 (*c* 1, $CHCl_3$); R_f = 0.45 (PE/EA = 2/1).

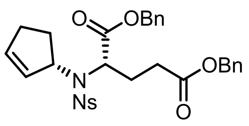

Compound **7e,** 80% yield, d.r.>20:1, colorless oil
^{1}H NMR (400 MHz, $CDCl_3$) δ 8.10 – 8.04 (m, 1H), 7.63 – 7.51 (m, 2H), 7.49 – 7.43 (m, 1H), 7.38 – 7.24 (m, 10H), 6.10 – 5.96 (m, 1H), 5.66 – 5.51 (m, 1H),, 5.16 – 4.96 (m, 4H), 4.91 – 4.83 (m, 1H), 4.78 (dd, *J* = 9.4, 3.6 Hz, 1H), δ 3.54 (dd, *J* = 16.7, 9.4 Hz, 1H), 2.79 (dd, *J* = 16.7, 3.8 Hz, 1H), 2.55 – 2.39 (m, 1H), 2.32 – 2.17 (m, 2H), 1.85 – 1.72 (m, 1H); ^{13}C NMR (101 MHz, $CDCl_3$) δ 170.5, 137.4, 134.8, δ 133.6, 133.5, 131.5, 130.7, 129.0, 128.4, 128.3, 128.3, 128.2, 128.2, 124.0, 67.9, 66.9, 66.0, 54.7, 38.1, 31.2, 28.7. IR (neat) v_{max} 2925, 1734, 1543, 1372, 1348, 1259, 1160 cm^{-1}; HRMS (ESI): $[M+NH_4]^+$ calculated for $C_{30}H_{30}N_2O_8S$: 596.2061, found: 596.2059; $[\alpha]_D^{22}$ +7.0 (*c* 1, $CHCl_3$); R_f = 0.55 (PE/EA = 2/1).

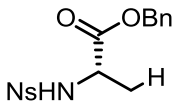

Compound **21a**. colorless oil
^{1}H NMR (400 MHz, $CDCl_3$) δ 8.00 (dd, *J* = 7.7, 1.6 Hz, 1H), 7.81 (dd, *J* = 7.7, 1.6 Hz, 1H), 7.68 – 7.58 (m, 2H), 7.35 – 7.31 (m, 3H), 7.22 – 7.09 (m, 2H), 6.15 (d, *J* = 8.6 Hz, 1H), 4.97 – 4.90 (m, 2H), 4.34 – 4.26 (m, 1H), 1.50 (d, *J* = 7.2 Hz, 3H); ^{13}C NMR (101 MHz, $CDCl_3$) δ 171.3, 147.6, 142.4, 134.7, 133.5, 132.8, 130.3, 128.6, 128.6, 128.2, 125.6, 67.4, 52.6, 19.8; IR (neat) v_{max} 3325, 2916, 1741, 1540, 1353, 1172, 1123 cm^{-1}; HRMS (ESI): $[M+NH_4]^+$ calculated for $C_{16}H_{20}N_3O_6S$: 382.1067, found: 382.1066; $[\alpha]_D^{22}$ -176.1 (*c* 1, $CHCl_3$); R_f = 0.31 (PE/EA = 2/1).

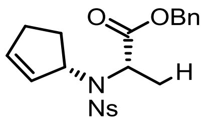

Compound **7f,** 95% yield, d.r.>20:1, colorless oil
^{1}H NMR (400 MHz, $CDCl_3$) δ 8.10 (dd, *J* = 7.9, 1.4 Hz, 1H), 7.62 – 7.59(m, 1H), 7.55 – 7.50 (m, 2H), 7.36 – 7.28 (m, 5H), 5.98 – 5.94 (m, 1H), 5.60 – 5.56 (m, 1H), 5.15 (d, *J* = 12.2 Hz, 1H), 5.09 (d, *J* = 12.2 Hz, 1H), 4.74 – 4.71 (m, 1H), 4.56 (q, *J* = 7.2 Hz, 1H), 2.57 – 2.50 (m, 1H), 2.32 – 2.21 (m, 2H), 1.97 – 1.90 (m, 1H), 1.66 (d, *J* = 7.2 Hz, 3H); ^{13}C NMR (101 MHz, $CDCl_3$) δ 171.6, 148.5, 136.1, 135.2, 134.8, 133.3, 133.1, 131.3, 131.0, 129.6, 128.5, 128.4, 128.3, 123.9, 67.4, 65.7, 55.3, 31.5, 29.7, 18.3; IR (neat) v_{max} 2947, 1741, 1543, 1372, 1342, 1160, 1126 cm^{-1}; HRMS (ESI): $[M+NH_4]^+$ calculated for $C_{21}H_{26}N_3O_6S$: 448.1537, found: 448.1535; $[\alpha]_D^{22}$ -29.5 (*c* 1, $CHCl_3$); R_f = 0.43 (PE/EA = 2/1).

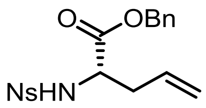

Substrate for compound **7g,** colorless oil
^{1}H NMR (400 MHz, $CDCl_3$) δ 8.00 (dd, *J* = 7.6, 1.4 Hz, 1H), 7.82 (dd, *J* = 7.6, 1.4 Hz, 1H), 7.67 – 7.59 (m, 2H), 7.33 – 7.32 (m, 3H), 7.22 – 7.18 (m, 2H), 6.12 (d, *J* = 8.9 Hz, 1H), 5.69 – 5.59 (m, 1H), 5.13 – 5.08 (m, 2H), 5.00 – 4.86 (m, 2H), 4.36 – 4.31 (m, 1H), 2.58 (t, *J* = 6.5 Hz, 2H); ^{13}C NMR (101 MHz, $CDCl_3$) δ 170.2, 147.6, 134.7, 134.3, 133.5, 132.8, 130.9, 130.3, 128.6, 128.6, 128.4, 125.5, 120.3, 67.4, 56.2, 37.4; IR (neat) v_{max} 3328, 1747, 1540, 1355, 1171, 1124 cm^{-1}; HRMS (ESI): $[M+NH_4]^+$ calculated for $C_{18}H_{22}N_3O_6S$: 408.1224, found: 408.1223; $[\alpha]_D^{22}$ –130.9 (*c* 1, $CHCl_3$); R_f = 0.54 (PE/EA = 2/1).

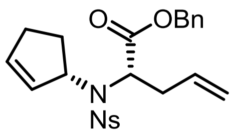

Compound **7g,** 71% yield, d.r.=8:1, colorless oil
^{1}H NMR (400 MHz, $CDCl_3$) δ 8.06 (dd, *J* = 8.8 , 1.6 Hz , 1H), 7.59 – 7.55 (m, 1H), 7.49 – 7.46 (m, 2H), 7.36 – 7.31 (m, 3H), 7.25 – 7.22 (m, 2H), 6.02 – 5.99 (m, 0.11H), 5.98 – 5.94 (m, 0.88H), 5.89 – 5.82 (m, 1H), 5.61 – 5.58 (m, 0.11H), 5.56 – 5.52 (m, 0.88H), 5.18 – 5.04 (m, 4H), 4.70 – 4.67 (m, 1H), 4.62 – 4.58 (m, 1H), 3.05 – 2.98 (m, 1H), 2.65 – 2.55 (m, 2H), 2.32 – 2.17 (m, 2 H), 2.11 – 2.05 (m, 1H); ^{13}C NMR (101 MHz, $CDCl_3$) δ 170.8, 148.3, 136.4, 135.0, 135.0, 134.0, 133.1, 131.5, 131.2, 129.4, 128.5, 128.4, 128.4, 123.8, 118.4, 67.4, 66.1, 60.6, 36.1, 31.6, 30.0; IR (neat) v_{max} 2924, 1739, 1542, 1371, 1345, 1161, 1061 cm^{-1}; HRMS (ESI): $[M+NH_4]^+$ calculated for $C_{23}H_{28}N_3O_6S$: 474.1693, found: 474.1687; $[\alpha]_D^{22}$ +8.2 (*c* 1, $CHCl_3$); R_f = 0.63 (PE/EA = 2/1).

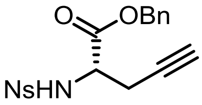

Substrate for compound **7h,** colorless oil

^{1}H NMR (400 MHz, $CDCl_3$) δ 8.03 – 8.01 (m, 1H), 7.84 – 7.82 (m, 1H), 7.63 – 7.61 (m, 2H), 7.35 – 7.30 (m, 3H), 7.23 – 7.18 (m, 2H), 6.44 (d, *J* = 9.1 Hz, 1H), 5.04 – 4.94 (m, 2H), 4.43 (dt, *J* = 9.5, 4.9 Hz, 1H), 2.88 – 2.74 (m, 2H), 2.05 (t, *J* = 2.6 Hz, 1H); ^{13}C NMR (101 MHz, $CDCl_3$) δ 169.1, 155.0, 147.5, 134.6, 133.6, 132.9, 130.2, 128.7, 128.6, 128.4, 125.7, 72.8, 67.9, 55.2, 24.0; IR (neat) v_{max} 3292, 2917, 1742, 1538, 1354, 1168, 1114, 739 cm^{-1}; HRMS (ESI): $[M+NH_4]^+$ calculated for $C_{18}H_{20}N_3O_6S$: 382.1067, found: 382.1066; $[\alpha]_D^{22}$ -153.6 (*c* 1, $CHCl_3$); R_f = 0.33 (PE/EA = 2/1).

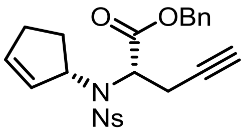

Compound **7h,** 85% yield, d.r.=10:1, colorless oil

^{1}H NMR (400 MHz, $CDCl_3$) δ 8.14 – 8.12 (m, 1H), 7.64 – 7.61 (m, 1H), 7.58 – 7.52 (m, 2H), 7.35 – 7.30 (m, 5H), 5.96 (dd, *J* = 6.0, 2.1 Hz ,0.96H), 5.62 (dd, *J* = 5.6, 2.3 Hz, 0.91H), 5.20 – 5.06 (m, 2H), 4.87 - 4.83 (m, 1H), 4.50 (dd, *J* =8.8, 4.2 Hz, 0.94H), 4.32 (dd, *J* =8.8, 4.2 Hz, 0.09H), 3.18 - 3.12 (m, 1H), 3.00 - 2.92 (m, 1H), 2.59 - 2.51 (m, 1H), 2.31 - 2.14 (m, 3H), 2.09 (t, *J* = 2.7 Hz, 1H); ^{13}C NMR (101 MHz, $CDCl_3$) δ 169.6, 148.5, 136.6, 134.8, 134.6, 133.3, 131.3, 130.9, 129.4, 128.5, 128.4, 128.4, 124.0, 80.2, 71.8, 67.8, 66.0, 58.5, 31.4, 29.2, 22.1, 14.1; IR (neat) v_{max} 3287, 2917, 1740, 1543, 1346, 1167, 742 cm^{-1}; HRMS (ESI): $[M+NH_4]^+$ calculated for $C_{23}H_{26}N_3O_6S$: 472.1537, found: 472.1535; $[\alpha]_D^{22}$ -20.2 (*c* 1, $CHCl_3$); R_f = 0.45 (PE/EA = 2/1)

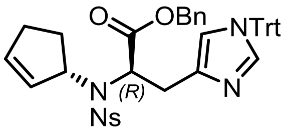

Compound **7i,** 77% yield, d.r.>20:1, colorless oil

^{1}H NMR (400 MHz, $CDCl_3$) δ 8.15 – 8.08 (m, 1H), 7.58 – 7.27 (m, 18H), 7.12 - 7.06 (m, 6H), 6.63 (s, 1H), 5.92 (dd, *J* = 5.5, 2.0 Hz, 1H), 5.21 (dd, *J* = 5.5, 2.0 Hz, 1H), 5.17 – 5.02 (m, 2H), 4.98 - 4.96 (m, 1H), 4.60 (t, *J* = 7.0 Hz, 1H), 3.54 (dd, *J* = 14.9, 7.4 Hz, 1H), 3.09 (dd, *J* = 14.9, 7.4 Hz, 1H), 2.42 – 2.36 (m, 1H), 2.20 – 2.07 (m, 2H), 2.00 – 1.99 (m, 1H); ^{13}C NMR (101 MHz, $CDCl_3$) δ 170.8, 148.7, 142.3, 137.9, 137.2, 135.2, 134.0, 133.2, 131.2, 131.0, 129.7, 128.8, 128.5, 128.2, 128.0, 128.0, 123.7, 120.1, 75.3, 67.4, 65.7, 58.6, 31.1, 31.0, 28.6; IR (neat) v_{max} 2926, 1741, 1544, 1372, 1161, 1128 cm^{-1}; HRMS (ESI): $[M+H]^+$ calculated for $C_{43}H_{39}N_4O_6S$: 739.2585, found: 739.2584; $[\alpha]_D^{22}$ +23.0 (*c* 1, $CHCl_3$); R_f = 0.35 (DCM/MeOH = 40/1 with 1/1000 ammonium hydroxide); m.p. = 83-86 ^{o}C.

Total synthesis of pseudopaline

Compound **13a**

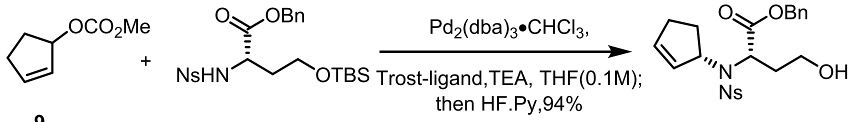

9 **8** **13a**

To a solution of $Pd_2(dba)_3$•$CHCl_3$ (63 mg, 0.061 mmol, 0.05 eq) and Trost-ligand *ent*-**L2** (144 mg, 0.183 mmol, 0.15 eq) in THF (10.2 mL) was added compound **9** (520 mg, 3.66 mmol, 3 eq) and TEA (370 mg, 3.66 mmol, 3 eq) at 0 ^{o}C, then a solution of compound **8** (620 mg, 1.22 mmol, 1 eq) in THF (2 mL) was added into the mixture which was stirred at 0 °C for 2 h. TLC showed **8** was consumed completely. To the mixutre at 0 °C was added HF-pyridine (70% solution) (0.63 mL, 4.88 mmol, 4 eq). After stirring for 10h at r.t., the mixture was cooled to 0 ^{o}C, diluted with EA (20 mL), quenched with saturated $NaHCO_3$ (20 mL), and the aqueous phase was extracted with EA (20 mL x 2). The combined organic phases were washed with brine, dried over Na_2SO_4 and concentrated *in vacuo* to afford crude product, which was purified by silica gel column chromatography (PE/EA=6/1 to 4/1) to afford compound **13a** (553 mg, 1.15 mmol, 94%) as an oil (d.r.=27:1).

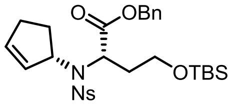

TBS-protection of **13a.**

^{1}H NMR (400 MHz, $CDCl_3$) δ 8.05 (d, *J* = 1.6 Hz, 1H), 7.61 – 7.57 (m, 1H), 7.52 – 7.48 (m, 2H), 7.34 – 7.28 (m, 5H), 5.95 – 5.93 (m, 1H), 5.63 – 5.61 (m, 1H), 5.13 (d, *J* = 12.1 Hz, 1H), 5.05 (d, *J* = 12.1 Hz, 1H), 4.85 – 4.81 (m, 1H), 4.51 (t, *J* = 6.7 Hz, 1H), 3.77 – 3.73 (m, 2H), 2.57 – 2.50 (m, 2H), 2.27 – 2.21 (m, 2H), 2.04 – 1.96 (m, 2H), 0.85 (s, 9H), 0.01 (s, 6H); ^{13}C NMR (101 MHz, $CDCl_3$) δ 171.2, 148.6, 136.2, 135.2, 134.5, 133.2, 131.2, 130.9, 129.8, 128.5, 128.3, 123.8, 67.3, 66.3, 59.9, 55.4, 35.6, 31.3, 28.8, 25.9, 18.2, –5.5, –5.5; IR(neat) v_{max} 2928, 2855, 1739, 1544, 1358, 1257, 1161, 1095 cm^{-1}; HRMS (ESI): $[M+NH_4]^+$ calculated for $C_{28}H_{42}N_3O_7SSi$: 592.2507, found: 592.2511; $[\alpha]_D^{19}$ –34.5(*c* 1, $CHCl_3$); R_f = 0.22 (PE/EA = 15/1).

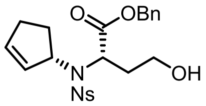

Compound **13a**

^{1}H NMR (400 MHz, $CDCl_3$) δ 8.04 (dd, *J* = 8.0, 4.0 Hz, 1H), 7.59–7.55 (m, 1H), 7.51 – 7.49 (m, 2H), 7.32 – 7.30 (m, 3H), 7.24 – 7.18 (m, 2H), 5.99 – 5.97 (m, 1H), 5.55 – 5.52 (m, 1H), 5.11 (d, *J* = 12 Hz, 1H), 5.04 (d, *J* = 12 Hz, 1H), 4.87 (dd, *J* = 9.2, 5.2 Hz,1H), 4.62 – 4.58 (m, 1H), 3.85 – 3.76 (m, 2H), 2.68 – 2.62 (m, 1H), 2.54 – 2.44 (m, 1H), 2.33 – 2.13 (m, 4H), 1.98 – 1.92 (m, 1H); ^{13}C NMR (101 MHz, $CDCl_3$) δ 171.3, 148.1, 136. 5, 134.8, 134.4, 133.3, 131.2, 129.0, 128.5, 128.4, 123.8, 67.5, 66.37, 58.3, 57.6, 34.0, 31.6, 29.7; IR (neat) v_{max} 3557, 2949, 1737, 1543, 1372, 1160, 1061 cm^{-1}; HRMS (ESI): $[M+NH_4]^+$ calculated for $C_{22}H_{28}N_3O_7S$: 478.1642, found: 478.1636; $[\alpha]_D^{24}$ 1.5 (*c* 1, $CHCl_3$); R_f = 0.22 (PE/EA = 2/1).

Compound **5a**

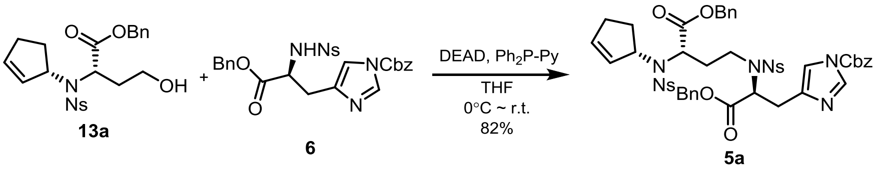

The Ph_2P-Py (264 mg, 1.00 mmol, 1.5 eq) was dissolved in THF (3 mL) and to this solution was added DEAD (158 μL, 1.00 mmol, 1.5 eq) at 0 ℃ under argon. The mixture was stirred for 10 minutes, then a solution of compound **6** (265 mg, 0.47 mmol, 0.7 eq) in THF (1 mL) was added. A solution of compound **13a** (310 mg, 0.67 mmol, 1 eq) in THF (2.7 mL) was dropwise into the solution. After stirring at r.t. for 2 h, the mixture was evaporated under *in vacuo*, and the residue was dissolved in EA and washed by 2N HCl (5 x 10 mL) and brine. The organic phase was concentrated *in vacuo.* The residue was purified by silica gel column chromatography (PE/EA = 7/1) to afford the desired product (389 mg, 0.39 mmol, 82%) as a colorless oil.

^{1}H NMR (400 MHz, $CDCl_3$) δ 8.06 – 7.99 (m, 1H), 7.97 (d, *J* = 0.9 Hz, 1H), 7.87 (dd, *J* = 7.9, 1.1 Hz, 1H), 7.62 – 7.54 (m, 1H), 7.53 – 7.26 (m, 18H), 7.22 – 7.12 (m, 2H), 5.96 (dd, *J* = 5.5, 2.1 Hz, 1H), 5.55 (dd, *J* = 5.5, 2.1 Hz, 1H), 5.38 (s, 2H), 5.16 – 4.86 (m, 5H), 4.70 – 4.66 (m, 1H), 4.36 (t, *J* = 6.5 Hz, 1H), 3.79 – 3.58 (m, 1H), 3.53 – 3.39 (m, 1H), 3.34 (dd, *J* = 15.6, 5.1 Hz, 1H), 3.12 (dd, *J* = 15.6, 9.6 Hz, 1H), 2.60 – 2.56 (m, 2H), 2.31 – 2.16 (m, 3H), 2.00 – 1.90 (m, 1H); ^{13}C NMR (101 MHz, $CDCl_3$) δ 170.7, 169.9, 148. 5, 148.4, 148.0, 138.9, 136.8, 136. 7, 135.0, 134.8, 134.4, 134.1, 133.4, 133.3, 132.2, 131.4, 131.4, 131.2, 130.0, 129.4, 129.1, 128.8, 128.8, 128.6, 128.5, 128.5, 128.5, 128.4, 123.9, 123.7, 115.0, 77.4, 77.1, 76.7, 69.8, 67.7, 67.5, 66.3, 60.3, 57.8, 45.3, 33.6, 31.6, 29.6, 29.2; IR (neat) v_{max} 2955, 1739, 1542, 1213, 1162, 970, 730, 580 cm^{-1}; HRMS (ESI): $[M+H]^+$ calculated for $C_{49}H_{47}N_6O_{14}S_2$: 1007.2586, found: 1007.2585; $[\alpha]_D^{21}$ -17.2 (*c* 1, $CHCl_3$); R_f = 0.29 (PE/EA = 1/1).

Compound **14a**

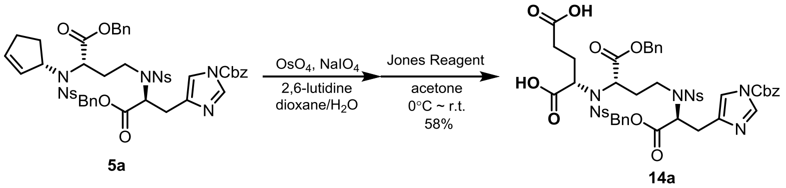

Compound **5a** (380 mg, 0.377 mmol, 1 eq) was dissolved in dioxane (25.1 mL) and H_2O (12.6 mL), and to this solution was added 2,6–lutidine (174 *μ*L, 1.51 mmol, 4 eq), OsO_4 (0.01 M in *t*-BuOH) (7.6 mL, 0.076 mmol, 0.2 eq) and $NaIO_4$ (808 mg, 3.77 mmol, 10 eq) at r.t.. After stirring for 3 h at r.t., the resulting solution was quenched by saturated $Na_2S_2O_3$ solution (12 ml) and extracted by EA (16 mL x 3) and the combined organic extracts were washed with saturated $Na_2S_2O_3$ solution (20 mL x 2), 5% NaH_2PO_4 solution (20 mL x 2) and brine. The organic phases were dried over anhydrous Na_2SO_4 and the filtrate was concentrated *in vacuo*. The residue was directly used in next step without further purification. The residue was dissolved in acetone (18.9 mL), then Jones reagent (2 M, 414 μL, 0.829 mmol, 2.2 eq) was added to the solution at 0 ℃. After stirring for 16 h at r.t., the reaction mixture was quenched by *i*–PrOH (1 mL), diluted with EA (20 mL) and washed with saturated $Na_2S_2O_3$ solution (10 mL x 2) and brine (10 mL). The water phase was extracted by EA (5 mL x 2). The combined organic phases were dried over anhydrous Na_2SO_4 and the filtrate was concentrated *in vacuo*. The residue was purified by silica gel column chromatography (DCM/MeOH = 100:1 with 1/10000 AcOH) to afford pure product **14a** (235 mg, 0.219 mmol, 58%) as white solid.

^{1}H NMR (400 MHz, $CDCl_3$) δ 10.35 (br, 2H), 8.42 (s, 1H), 7.97 (d, *J* = 7.2 Hz, 1H), 7.86 (d, *J* = 7.2 Hz, 1H), 7.65 – 7.01 (m, 23H), 5.47 – 5.28 (m, 2H), 5.13 – 4.63 (m, 6H), 4.47 (m, 1H), 3.89 – 3.65 (m, 1H), 3.49 – 3.38 (m, 2H), 3.14 (dd, *J* = 16.0, 9.6 Hz, 1H), 2.67 – 2.25 (m, 4H), 2.16 – 2.08 (m, 1H), 1.98 – 1.86 (m, 1H); ^{13}C NMR (101 MHz, $CDCl_3$) δ 177.0, 173.0, 171.9, 169.1, 147.9, 147.8, 147.3, 136.7, 135.4, 134.5, 134.4, 133.8, 133.4, 132.7, 131.9, 131.8, 131.7, 131.6, 131.1, 129.2, 128.9, 128.9, 128.8, 128.6, 128.5, 123.9, 123.8, 116.2, 77.4, 77.3, 77.1, 76.8, 70.9, 68.1, 67.9, 60.1, 58.2, 56.8, 45.2, 31.3, 28.1, 16.3; IR (neat) ν_{max} 3148, 2961, 1739, 1544, 1372, 1165, 753, 591 cm^{-1}; HRMS (ESI): $[M+H]^+$ calculated for $C_{49}H_{47}N_6O_{18}S_2$: 1071.2384, found: 1071.2585; $[\alpha]^{19}_D$ +76.3 (*c* 1, $CHCl_3$); R_f = 0.59 (DCM/MeOH = 10/1 with 1/10000 AcOH); m.p. = 102-105 ℃.

Pseudopaline **2**

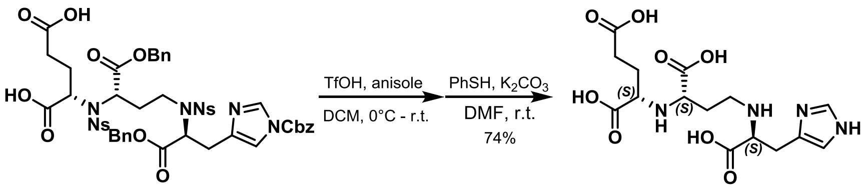

14a **2**

To a solution of diacid **14a** (184 mg, 0.172 mmol, 1 eq) in DCM (17.2 mL) was added anisole (206 μL, 1.89 mmol, 11 eq) and trifluoromethanesulfonic acid (151 μL, 1.72 mmol, 10 eq) at 0 ℃. The reaction mixture was stirred at 0 ℃ for 0.5 h, then warmed to room temperature, and stirred for 1.5 h. The reaction was quenched with a solution of $NaHCO_3$ (218 mg, 2.58 mmol, 15 eq) in water (20 mL) at 0 ℃ and stirred for an additional 0.5 h. The mixture was washed with DCM (20 mL x 3) and the water phase was concentrated *in vacuo* and directly used for the next step without further purification. The crude mixture and K_2CO_3 (190 mg, 1.37 mmol, 8 eq) were dissolved in dry DMF (8.6 mL) and to this solution was added thiophenol (352 μL, 3.44 mmol, 20 eq). After stirring for 14 h at r.t. water (20 mL) was added and the aqueous phase was washed with DCM (20 mL x 3). Then the aqueous phase was concentrated *in vacuo* to the resulting solid which was purified by gel column to afford **2** (49 mg, 0.127 mmol, 74 %) as a white solid. ^{1}H NMR (400 MHz, D_2O) δ 7.72 (d, *J* = 0.9 Hz, 1H), 6.92 (s, 1H), 3.61 (t, *J* = 6.3 Hz, 1H), 3.32 – 3.19 (m, 2H), 3.10 (dt, *J* = 11.2, 5.4 Hz, 1H), 3.03 (d, *J* = 6.4 Hz, 2H), 2.90 (ddd, *J* = 12.4, 8.9, 5.8 Hz, 1H), 2.20 (t, *J* = 7.9 Hz, 2H), 2.03 – 1.78 (m, 4H); ^{13}C NMR (101 MHz, D_2O) δ 181.8, 176.9, 176.1, 175.5, 135.6, 131.3, 117.4, 62. 9, 62.8, 62.2, 45.6, 33.8, 28.6, 28.4, 28.1; IR (neat) ν_{max} 3359, 1570, 1395, 1103, 995, 831cm^{-1}; HRMS (ESI): $[M-H]^-$ calculated for $C_{15}H_{21}N_4O_8$: 385.1365, found: 385.1372; $[\alpha]^{19}_D$ + 5.9 (*c* 1, H_2O); m.p.>330°C.

Total synthesis of *epi*-pseudopaline

Same procedure as the synthesis of pseudopaline.

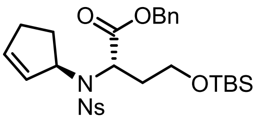

Compound **7b,** colorless oil, 27:1 d.r.

^{1}H NMR (400 MHz, $CDCl_3$) δ 8.05 (d, *J* = 8.0 Hz, 1H), 7.60 – 7.57 (m, 1H), 7.54 – 7.45 (m, 2H), 7.35 – 7.29 (m, 5H), 5.97 (dd, *J* = 5.2, 2.0 Hz, 1H), 5.60 (dd, *J* = 5.2, 2.0 Hz, 1H), 5.14 (d, *J* = 12.2 Hz, 1H), 5.08 (d, *J* = 12.2 Hz, 1H), 5.04 – 5.00 (m, 1H), 4.36 (dd, *J* = 8.0, 4.2 Hz, 1H), 3.71 (dd, *J* = 8.0, 4.0 Hz, 2H), 2.56 – 2.31 (m, 2H), 2.32 – 2.14 (m, 2H), 2.10 – 1.84 (m, 2H), 0.85 (s, 9H), –0.01 (d, *J* = 4.0 Hz , 6H); ^{13}C NMR (101 MHz, $CDCl_3$) δ 171.0, 148.6, 137.2, 135.1, 134.1, 133.3, 131.3, 130.7, 128.8, 128.5, 128.4, 128.3, 123.9, 67.4, 65.8, 60.0, 54.5, 35.2, 31.1, 28.8, 25.9, 18.2, –5.4, –5.6; IR (neat) ν_{max} 2958, 2928, 2856, 1740, 1544, 1360, 1259, 1091 cm^{-1}; HRMS (ESI): $[M+NH_4]+$ calculated for $C_{28}H_{42}N_3O_7SSi$: 592.2507, found: 592.2515; $[\alpha]^{24}_D$ –35.8 (*c* 1, $CHCl_3$); R_f = 0.22 (PE/EA = 15/1).

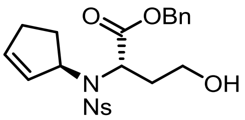

Compound **13b**, 87% yield, colorless oil, d.r.=27:1

^{1}H NMR (400 MHz, $CDCl_3$) δ 8.05 (d, *J* = 8.0 Hz, 1H), 7.58 – 7.53 (m, 1H), 7.48 (d, *J* = 8.2 Hz, 2H), 7.33 (m, 3H), 7.25–7.23 (m, 2H), 5.99 (dd, *J* = 5.6, 2.4 Hz, 1H), 5.56 (dd, *J* = 5.6, 2.4 Hz, 1H), 5.13 – 4.97 (m, 3H), 4.50 (t, *J* = 6.8 Hz, 1H), 3.80 – 3.72 (m, 2H), 2.54 – 2.48 (m, 2H), 2.32 – 2.27 (m, 2H), 2.00 – 1.97 (m, 3H); ^{13}C NMR (101 MHz, $CDCl_3$) δ 171.3, 148.4, 137.5, 134.7, 133.7, 133.4, 131.4, 130.7, 128.6, 128.6, 128.5, 128.45, 123.9, 67.7, 65.5, 59.2, 55.6, 34.4, 31.1, 29.5; IR (neat) ν_{max} 3559, 2961, 2924, 1738, 1542, 1373, 1256, 1158 cm^{-1}; HRMS (ESI): $[M+NH_4]^+$ calculated for $C_{22}H_{28}N_3O_7S$: 478.1642 , found: 478.1648; $[\alpha]^{22}_D$ –31.7 (*c* 1, $CHCl_3$); R_f = 0.22 (PE/EA = 2/1).

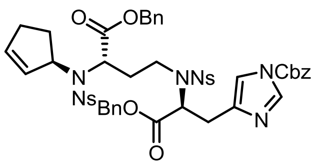

Compound **5b,** 80% yield, colorless oil

^{1}H NMR (400 MHz, $CDCl_3$) δ 8.06 – 7.98 (m, 2H), 7.91 – 7.87 (m, 1H), 7.60 – 7.26 (m, 20H), 7.22 – 7.12 (m, 2H), 5.96 (dd, *J* = 5.5, 2.1 Hz, 1H), 5.55 (dd, *J* = 5.5, 2.1 Hz, 1H), 5.38 (s, 2H), 5.16 – 5.03 (m, 3H), 4.97 – 4.90 (m, 3H), 4.10 (d, *J* = 8.0 Hz, 1H), 3.79 – 3.58 (m, 1H), 3.44 – 3.37 (m, 1H), 3.34 (dd, *J* = 15.6, 5.1 Hz, 1H), 3.12 (dd, *J* = 15.6, 9.6 Hz, 1H), 2.59 – 2.49 (m, 1H), 2.45 – 2.35 (m, 1H), 2.26 – 2.10 (m, 3H), 2.00 – 1.90 (m, 1H); ^{13}C NMR (101 MHz, $CDCl_3$) δ 170.7, 169.8, 148.4, 148.3, 147.8, 138. 9, 138.1, 136.7, 134.8, 134. 8, 134.0, 133.8, 133.4, 133.4, 132.1, 131.6, 131.3, 131.0, 130.7, 129.0, 128.8, 128.7, 128.5, 128.5, 128.4, 128.4, 128.4, 128.3, 128.2, 124.0, 123.6, 114.9, 69.7, 67.7, 67.3, 65.3, 60.2, 55.8, 45.1, 33.7, 31.2, 29.4, 28.6; IR (neat) v_{max} 2958, 1742, 1543, 1405, 1372, 1244, 1163 cm^{-1}; HRMS (ESI): $[M+H]^+$ calculated for $C_{49}H_{47}N_6O_{14}S_2$: 1007.2586, found: 1007.2587; $[\alpha]_D^{19}$–23.7 (*c* 1, $CHCl_3$); R_f = 0.29 (PE/EA = 1/1).

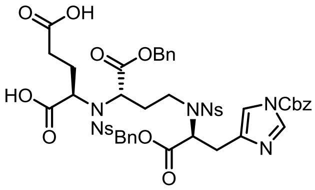

Compound **14b**, 55% yield for 2 steps, white solid

^{1}H NMR (400 MHz, $CDCl_3$) δ 8.42 (s, 2H contain COOH), 7.97 (d, *J* = 7.2 Hz, 1H), 7.86 (d, *J* = 7.2 Hz, 1H), 7.55 – 7.29 (m, 18H), 7.25 – 7.22 (m, 3H), 7.17 – 7.13 (m, 2H), 5.38 (s, 2H), 5.18 – 5.08 (m, 2H), 4.94 (s, 2H), 4.89 (t, *J* = 6 Hz, 1H), 4.50 – 4.46 (m, 1H), 4.29 – 4.25 (m, 1H), 3.65 – 3.59 (m, 1H), 3.56 – 3.49 (m, 1H), 3.44 (dd, *J* = 8.4, 4.2 Hz, 1H), 3.11 (dd, *J* = 8.4, 4.2 Hz, 1H), 2.67 – 2.62 (m, 1H), 2.51 – 2.40 (m, 2H), 2.35 – 2.28 (m, 2H), 2.11 – 2.04 (m, 1H); ^{13}C NMR (126 MHz, $CDCl_3$) δ 176.7, 172.6, 170.0, 169.6, 148.7, 147.8, 147.5, 137.0, 136.5, 134.8, 134.7, 134.0, 133.6, 133.5, 132.7, 132.2, 131.7, 131.2, 130.9, 129.4, 129.0, 128.9, 128.8, 128.7, 128.6, 128.6, 128.6, 128.6, 128.5, 128.5, 128.4, 128.4, 128.3, 127.7, 127.0, 123.8, 123.8, 115.8, 70.6, 69.7, 68.1, 67.6, 60.5, 59.4, 57.8, 32.3, 30.4, 28.3, 25.6; IR (neat) v_{max} 2923, 1737, 1543, 1372, 1259, 1017 cm^{-1}; HRMS (ESI): $[M+H]^+$ calculated for $C_{49}H_{47}N_6O_{18}S_2$: 1071.2383, found: 1071.2396; $[\alpha]_D^{21}$ –2.5 (*c* 0.8, $CHCl_3$); Rf =0.47 (DCM/AcOH = 20/1); m.p. = 89-91℃.

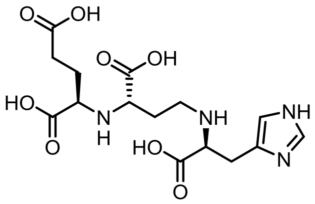

epi-pseudopaline **3**, 80% yield for 2 steps, white solid.

^{1}H NMR (400 MHz, D_2O) δ 7.70 (s, 1H), 6.96 (s, 1H), 3.68 (t, *J* = 7.2 Hz, 1H), 3.43 (t, *J* = 7.2 Hz, 1H), 3.32 (t, *J* = 7.2 Hz, 1H), 3.07 (m, 2H), 2.99 (m, 2H), 2.25 (t, *J* = 7.2 Hz, 2H), 2.00 (m, , 2H), 1.89 (q, *J* = 7.2 Hz, 2H); ^{13}C NMR (101 MHz, D_2O) δ 181.8, 175.7, 174.9, 174.8, 135.9, 131.7, 117.1, 62.3, 61.5, 59.9, 44.3, 33.7, 27.7, 27.0, 26.3; IR (neat) v_{max} 3395, 1628, 1574, 1399, 1318, 1118, 835, 758cm^{-1}; HRMS (ESI): $[M+H]^+$ calculated for $C_{15}H_{23}N_4O_8$: 387.1510 , found: 387.1511; $[\alpha]_D^{21}$+9.5 (*c* 0.9, H_2O); m.p. >330 ℃.

Synthesis of Fmoc-pseudopaline 17

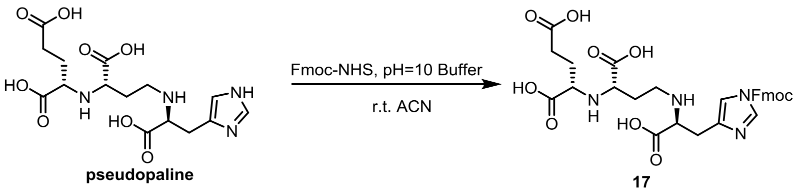

Scheme S2: Synthesis of the Fmoc-pseudopaline

To a solution of pseudopaline (4 mg, 0.0104 mmol, 1 eq) in water (0.6 mL) and pH=10 buffer (0.4 mL) was added a solution of Fmoc–NHS (17 mg, 0.052 mmol, 5 eq) in ACN (0.6 mL) at room temperature. After stirring for 3h at r.t., the reaction mixture was subjected directly to C–18 reverse phase column (a gradient of 0% – 50% acetonitrile in water) to afford the crude product which was purified

further by reverse phase prep-TLC with 30% acetonitrile in water to give pure Fmoc–pseudopaline (2.1 mg, 3.4 μmol, 33%). This compound is unstable in water for a long period of time, so the NMR spectrum was recorded immediately.
^{1}H NMR (400 MHz, D_2O) δ 7.92 (t, *J* = 7.2, 3H), 7.72 (d, *J* = 7.2, 2H), 7.48 (t, *J* = 7.2, 3H), 7.42 (t, *J* = 7.2, 3H), 7.20 (s,1H), 4.92 (d, *J* = 5.2, 2H), 4.49 (t, *J* = 5.2, 1H), 3.89 (t, *J* = 5.6, 1H), 3.65 – 3.59 (m, 2H), 3.24 (t, *J* = 7.2, 2H), 3.09 (d, *J* = 5.2, 2H), 2.38 (t, *J* = 7.2, 2H), 2.30 – 2.21(m, 2H), 2.10 – 2.08(m, 2H); ^{13}C NMR (201 MHz, D_2O) δ 180.3, 173.2, 172.2, 172.0, 149.0, 143.1, 141.0, 138.0, 136.2, 128.2, 127.5, 125.0, 120.2, 115.6, 69.4, 62.8, 61.6, 59.8, 46.4, 43.5, 32.8, 27.4, 26.8, 26.5; IR (neat) ν_{max} 3397, 1767, 1700, 1616, 1532, 1491, 1449, 1410, 1281, 1248, 1215,1169, 1107, 1104, 763, 740 cm^{-1}; HRMS (ESI): $[M+H]^+$ calculated for $C_{30}H_{33}N_4O_{10}$: 609.2191, found: 609.2193; $[M-H]^-$ calculated for $C_{30}H_{31}N_4O_{10}$: 607.2046, found: 607.2046;
$[\alpha]_D^{20}$ +18 (*c* 0.1, H_2O); R_f = 0.3 (ACN/H_2O = 3:7).

Natural product isolation

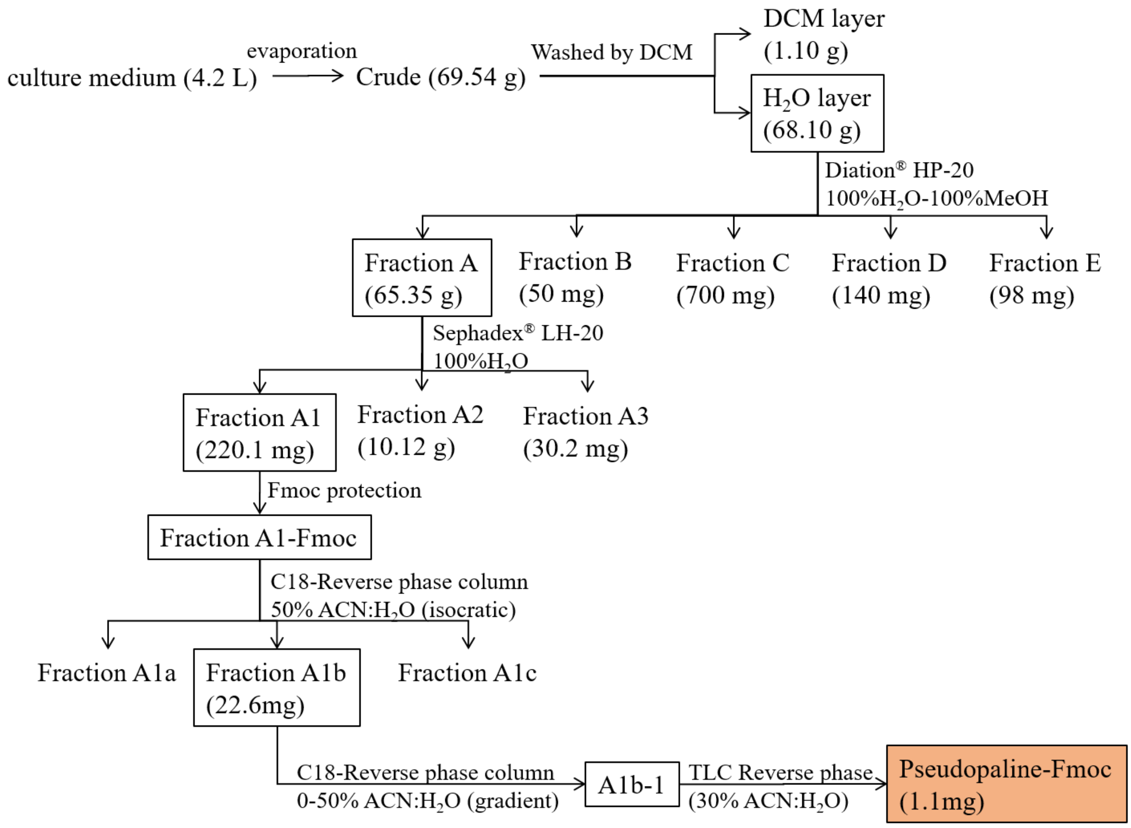

Scheme S3: Isolation of the Fmoc-pseudopaline

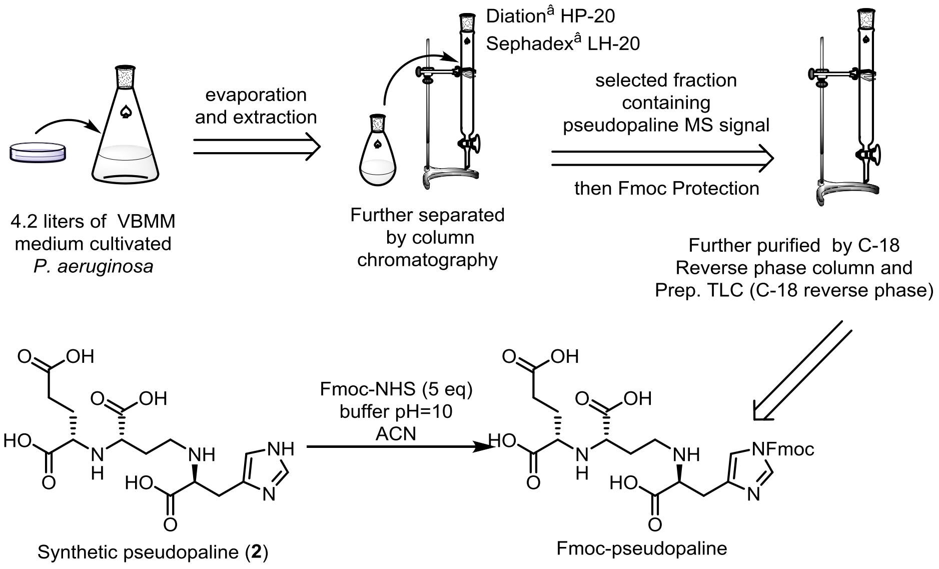

Scheme S4. Comparison of the natural Fmoc-pseudopaline and synthetic Fmoc-pseudopaline.

The bacteria cells were separated from the broth by centrifugation and filtration. The filtrate (4.2 L) was evaporated under reduced pressure to afford a crude mixture (69.54 g) then washed by CH_2Cl_2. The residue (68.10 g) was applied on a Diation® HP-20 column eluting with H_2O (1 L), 10% MeOH (1 L), 25% MeOH (1 L), 60% MeOH (1 L), and 100% MeOH (1 L) to yield five fractions (A-E). 50 μL

of each fraction was added to 50 μL of borate buffer (0.2 M, pH 10.0) and 100 μL of Fmoc-NHS (100 mM in acetonitrile), and stirred at room temperature overnight, followed by addition of 200 μL MeOH then subjected to LC–MS analysis.
Fraction A (65.35 g), where Fmoc-pseudopaline derivative was previously detected by LC-MS after modification of Fmoc, was eluted through Sephadex® LH-20 column with 100% H_2O and yielded three sub-fractions, A1-A3. 50 μL of each sub-fraction was added to 50 μL of borate buffer (0.2 M, pH 10.0) and 100 μL of Fmoc-NHS (100 mM in acetonitrile), and stirred at room temperature overnight, followed by addition of 200 μL MeOH then subjected to LC–MS analysis by which Fmoc-pseudopaline was found from sub-fraction A1.

Sub-fraction A1 (220.1 mg) was added to Fmoc-NHS in acetonitrile. After stirring for overnight, the mixture was subjected to C-18 reverse phase column with an isocratic conditions of 50% acetonitrile in water giving three sub-fractions (A1a-A1c). Sub-fraction A1b (22.6 mg) was further purified by C-18 reverse phase column with a gradient of 0%-50% acetonitrile in water followed by reverse phase prep. TLC with 30% acetonitrile in water to afford Fmoc-pseudopaline (1.1 mg).
^{1}H NMR (400 MHz, D_2O) δ 7.92 (t, *J* = 7.2, 3H), 7.73 (d, *J* = 7.2, 2H), 7.49 (t, *J* = 7.2, 3H), 7.41 (t, *J* = 7.2, 3H), 7.20 (s,1H), 4.94 (d, *J* = 5.2, 2H), 4.50 (t, *J* = 5.2, 1H), 3.90 (t, *J* = 5.6, 1H), 3.66–3.60 (m, 2H),3.25 (t, *J* = 7.2, 2H), 3.10 (d, *J* = 5.2, 2H), 2.46 (t, *J* = 7.2, 2H), 2.32 – 2.24 (m, 2H), 2.15 – 2.09 (m,2H); ^{13}C NMR (201 MHz, D_2O) δ 180.2, 173.2, 172.2, 172.0, 149.0, 143.1, 141.0, 138.0, 136.2, 128.2, 127.5, 125.0, 120.2, 115.6, 69.4, 62.8, 61.6, 59.9, 46.4, 43.5, 32.7, 27.4, 26.8, 26.4; IR (neat) ν_{max} 3397, 1767, 1700, 1616, 1532, 1491, 1449, 1410, 1281, 1248, 1215, 1169, 1107, 1104, 763, 740 cm^{-1}; HRMS (ESI): $[M+H]^+$ calculated for $C_{30}H_{33}N_4O_{10}$: 609.2191, found: 609.2204; $[M-H]^-$ calculated for $C_{30}H_{31}N_4O_{10}$: 607.2046, found: 607.2047; $[\alpha]^{20}_D$ +15 (*c* 0.1, H_2O); R_f = 0.3 (ACN/H_2O = 3:7).

Syntheses of the analogs 15a, 15b and 15c

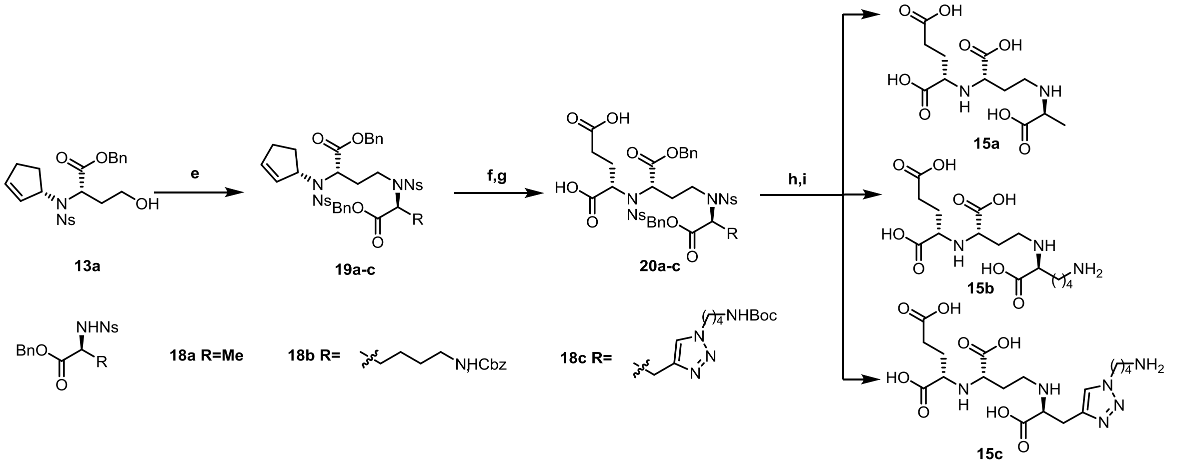

Scheme S5: (e) **18a-c** (1.2 eq), Ph_3P (1.5 eq), DEAD (1.5 eq), THF (0.1 M), 0ºC-r.t., 2 h; 76% for **19a**, 78% for **19b**, 96% for **19c**; (f) OsO_4 (0.2 eq), $NaIO_4$ (10 eq), dioxane/H_2O (2:1, 0.01 M) 2,6-lutidine (4 eq), r.t., 3 h; (g) Jones reagent (2.2 eq), acetone (0.02 M), 0ºC-r.t., 16 h; 65% for **20a**, 2 steps, 67% for **20b**, 2 steps, 54% for **20c**, 2 steps; (h) TfOH (5 eq), anisole (15 eq), DCM (0.01 M), 0ºC, 0.5 h; r.t., 2 h for **20a**; TfOH (10 eq), anisole (15 eq), DCM (0.01 M), 0ºC, 0.5 h; r.t., 2 h for **20b**; TfOH (10 eq), anisole (11 eq), DCM (0.01 M), 0ºC, 0.5 h; r.t., 1.5 h for **20c**; (i) PhSH (20 eq), K_2CO_3 (8 eq), DMF (0.02 M), r.t., 14 h; 75% for **15a**, 2 steps, 72% for **15b**, 2 steps, 78% for **15c**, 2 steps.

Compound **19a**

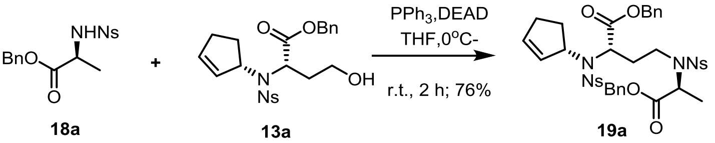

The PPh_3 (86 mg, 0.33 mmol, 1.5 eq) was dissolved in THF (1 mL) and to this solution was added DEAD (52 *μ*L, 0.33 mmol, 1.5 eq) at 0 ℃ under argon. The mixture was stirred for 10 minutes, then a solution of compound **18a** (95 mg, 0.26 mmol, 1.2 eq) in THF (0.7 mL) was added. A solution of compound **13a** (100 mg, 0.22 mmol, 1.0 eq) in THF (0.5 mL) was added dropwise into the solution. After stirring at r.t. for 2 h, the mixture was evaporated *in vacuo*, and the residue was purified by silica gel column chromatography (DCM/EA = 10/1) to afford the desired product (134 mg, 0.167 mmol, 76%) as a colorless oil.

^1H NMR (400 MHz, $CDCl_3$) δ 8.05 (dd, *J* = 8.3, 1.4 Hz, 1H), 7.92 (dd, *J* = 7.9, 1.5 Hz, 1H), 7.65 – 7.35 (m, 6H), 7.35 – 7.30 (m, 6H), 7.28 – 7.24 (m, 2H), 7.23 – 7.17 (m, 2H), 6.00 (dd, *J* = 5.6, 2.3 Hz, 1H), 5.57 (dd, *J* = 5.6, 2.3 Hz, 1H), 5.13 (d, *J* = 12.1 Hz, 1H), 5.04 (d, *J* = 12.1 Hz, 1H), 5.00 (d, *J* = 12.2 Hz, 1H), 4.91 (d, *J* = 12.2 Hz, 1H), 4.84 (q, *J* = 7.3 Hz, 1H), 4.68 (d, *J* = 8.5 Hz, 1H), 4.43 (t, *J* = 6.7 Hz, 1H), 3.70 (ddd, *J* = 16.1, 10.9, 5.6 Hz, 1H), 3.37 (ddd, *J* = 15.6, 11.2, 4.5 Hz, 1H), 2.67 – 2.53 (m, 2H), 2.36 – 2.20 (m, 3H), 2.02 (ddd, *J* = 14.4, 7.2, 3.6 Hz, 1H), 1.57 (d, *J* = 7.4 Hz, 3H); ^{13}C NMR (101 MHz, $CDCl_3$) δ 171.0, 170.7, 148.6, 148.0, 136.9, 135.1, 135.0, 134.6, 133.6, 133.5, 132.6, 131.7, 131.5, 131.4, 131.0, 129.5, 128.8, 128.7, 128.7, 128.5, 124.1, 124.0, 67.9, 67.5, 66.5, 58.0, 56.6, 45.0, 34.3, 31.7, 29.5, 17.3; IR (neat) v_{max} 1741, 1544, 1556, 1373, 1159, 1061 cm^{-1}; HRMS (ESI): $[M+NH_4]^+$ calculated for $C_{38}H_{42}N_5O_{12}S_2$: 824.2266, found: 824.2263; $[\alpha]^{21}_{D}$ – 21.2 (*c* 1, $CHCl_3$); R_f = 0.30 (PE/EA = 2/1).

Compound **20a**

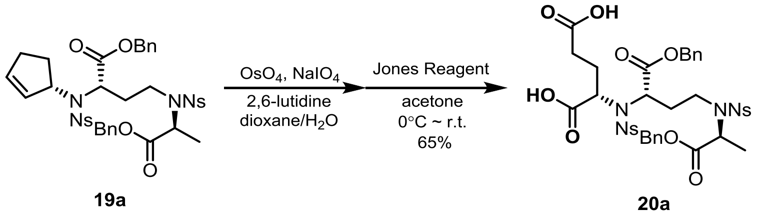

Compound **19a** (120 mg, 0.15 mmol, 1 eq) was dissolved in dioxane (10 mL) and H_2O (5 mL), and to this solution was added 2,6–lutidine (69 *μ*L, 0.59 mmol, 4 eq), OsO_4 (0.01M in *t*-BuOH) (3.0 mL, 0.03 mmol, 0.2 eq) and $NaIO_4$ (317 mg, 1.48 mmol, 10 eq) at r.t.. After stirring for 3 h at r.t., the resulting solution was quenched by saturated $Na_2S_2O_3$ solution (12 mL) and extracted by EA (10 mL x 3) and the combined organic extracts were washed with saturated $Na_2S_2O_3$ solution (15 mL x 2), 5% NaH_2PO_4 solution (15 mL x 2) and brine, dried over anhydrous Na_2SO_4 and the filtrate was concentrated *in vacuo*. The residue was directly used in next step without further purification. The residue was dissolved in acetone (7.5 mL) and Jones reagent (2 M, 163 μL, 0.33 mmol, 2.2 eq) was added to the solution at 0 ℃ and the mixture was allowed to stir for 16 h at r.t. before quenched by *i*–PrOH (1 mL). The mixture was diluted by EA (20 mL) and washed by saturated $Na_2S_2O_3$ solution (10 mL x 2) and brine (10 mL), the water phase was extracted by EA (5 mL). The organic phase was dried over anhydrous Na_2SO_4 the filtrate was concentrated *in vacuo*. The residue was purified by silica gel column chromatography to afford pure product **20a** (85 mg, 0.097 mmol, 65%) as white solid.

^1H NMR (400 MHz, $CDCl_3$) δ 8.10 (d, *J* = 7.6 Hz, 1H), 7.93 (d, *J* = 7.6, 1H), 7.60 – 7.46 (m, 5H), 7.42 – 7.39 (m, 1H), 7.32 – 7.28 (m, 6H), 7.25 – 7.22 (m, 2H), 7.20 – 7.16 (m, 2H), 5.11 (d, *J* = 12.1 Hz, 1H), 5.03 (d, *J* = 12.1 Hz, 1H), 4.96 (d, *J* = 12.2 Hz, 1H), 4.88 (d, *J* = 12.2 Hz, 1H), 4.81 (q, *J* = 7.5 Hz, 1H), 4.74 (t, *J* = 6.5 Hz, 1H), 4.41 (dd, *J* = 8.9, 4.5 Hz, 1H), 3.77 – 3.65 (m, 1H), 3.39 – 3.25 (m, 1H), 2.64 – 2.35 (m, 5H), 2.11 – 1.98 (m, 1H), 1.51 (d, *J* = 7.4 Hz, 3H); ^{13}C NMR (101 MHz, $CDCl_3$) δ 177.6, 172.5, 171.8, 171.1, 148.2, 147.9, 135.1, 134.4, 134.4, 133.8, 132.5, 132.3, 132.2, 132.0, 131.8, 131.1, 129.1, 128.9, 128.9, 128.8, 128.7, 128.7, 128.6, 128.5, 128.5, 128.4, 128.4, 124.3, 124.0, 68.7, 67.5, 58.8, 58.1, 56.7, 44.7, 33.0, 31.0, 26.3, 17.2; IR (neat) v_{max} 1740, 1544, 1372, 1153, 750, 587 cm^{-1}; HRMS (ESI): $[M+NH_4]^+$ calculated for $C_{38}H_{42}N_5O_{16}S_2$: 888.2062, found: 888.2048; $[\alpha]_D^{22}$ – 26.2 (*c* 1.9, $CHCl_3$); R_f =0.45 (DCM/MeOH = 10/1); m.p. = 125-128 ℃.

Compound **15a**

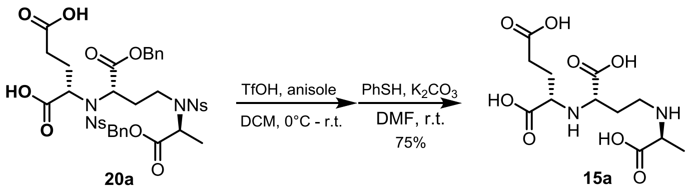

To a solution of diacid **20a** (40 mg, 0.046 mmol, 1 eq) in DCM (4.6 mL) was added anisole (75 *μ*L, 0.690 mmol, 15 eq) and trifluoromethanesulfonic acid (20 *μ*L, 0.23 mmol, 5 eq) at 0 ℃. The reaction mixture was stirred at 0 ℃ for 0.5 h, then warmed to room temperature, and stirred for 2 h. The reaction was quenched with a solution of $NaHCO_3$ (31 mg, 0.370 mmol, 8 eq) in water (5 mL) at 0 ℃ and stirred for an additional 0.5 h. The mixture was washed with DCM (10 mL x 3) and the water phase was concentrated *in vacuo* and directly used for the next step without further purification. The crude mixture and K_2CO_3 (51 mg, 0.370 mmol, 8 eq) were dissolved in dry DMF (2.3 mL) and to this solution was added thiophenol (94 *μ*L, 0.920 mmol, 20 eq). The reaction mixture was stirred for 14 h at room temperature before water (10 mL) was added and washed with DCM (10 mL x 3). Then the aqueous phase was concentrated *in vacuo*. The resulting solid was purified by gel column to afford **15a** (11 mg, 0.034 mmol, 75 %) as a white solid.
^1H NMR (400 MHz, D_2O) δ 3.68 (m, 2H), 3.60 (t, *J* = 5.9 Hz, 1H), 3.24 (t, *J* = 7.7 Hz, 2H), 2.41 (t, *J* = 7.3 Hz, 2H), 2.29 (m, 2H), 2.11 (m, 2H), 1.49 (d, *J* = 7.2 Hz, 3H);
^{13}C NMR (201 MHz, D_2O) δ 181.2, 174.7, 173.5, 172.2, 63.0, 59.9, 57.9, 42.6, 33.6, 27.0, 26.8, 14.9; IR (neat) v_{max} 3383, 3038, 1514, 1399, 1117 cm^{-1}; HRMS (ESI): $[M+H]^+$ calculated for $C_{12}H_{21}N_2O_8$: 321.1292, found: 321.1293; $[\alpha]_D^{22}$ + 10.7 (*c* 1, H_2O); m.p.>330°C.

Compound **18b**

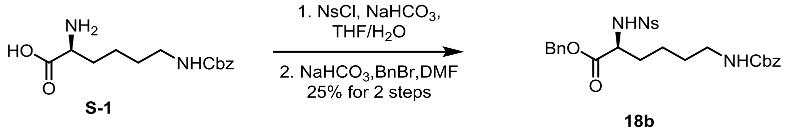

To a mixture of compound **S-1** (2 g, 7.14 mmol, 1 eq) in THF (20 mL) and H_2O (20 mL) was added $NaHCO_3$ (2.4 g, 28.60 mmol, 4 eq) and NsCl (1.6 g, 7.14 mmol, 1 eq) at r.t. After stirring for overnight, the mixture was diluted with EA (100 mL), and adjusted to pH~3 with 1N HCl. The organic phase was washed with 1N HCl (20 mL x 2), brine (20 mL x 2), dried with Na_2SO_4, and evaporated *in vacuo*. The residue was used in next step without purification. To the residue in DMF (20 mL) was added $NaHCO_3$ (0.90 g, 10.71 mmol, 1.5 eq) and BnBr (0.85 mL, 7.14 mmol, 1 eq), the mixture was stirred for 12 h at room temperature. The mixture was quenched with water (20 mL), and extracted with Et_2O (100 mL x 1). The organic phase was washed with H_2O (20 mL x 4), brine (20 mL x 1), dried with Na_2SO_4, and evaporated under *in vacuo*. The residue was further purified by silica gel column chromatography (PE/EA = 10/1) to afford the desired product (990 mg, 1.79 mmol, 25%) as yellow oil.
^1H NMR (400 MHz, $CDCl_3$) δ 7.98 (dd, *J* = 7.6, 1.7 Hz, 1H), 7.79 (dd, *J* = 7.8, 1.5 Hz, 1H), 7.65 – 7.55 (m, 2H), 7.39 – 7.28 (m, 8H), 7.16 (dd, *J* = 6.5, 3.0 Hz, 2H), 6.18 (d, *J* = 9.0 Hz, 1H), 5.10 (s, 2H), 4.91 (d, *J* = 12.4 Hz, 1H), 4.87 (d, *J* = 12.4 Hz, 1H), 4.77 (d, *J* = 6.2 Hz, 1H), 4.20 (td, *J* = 8.6, 4.9 Hz, 1H), 3.14 (q, *J* = 6.6 Hz, 2H), 1.91 – 1.82 (m, 1H), 1.81 – 1.69 (m, 1H), 1.5 – 1.34 (m, 4H); ^{13}C NMR (101 MHz, $CDCl_3$) δ 170.9, 156.6, 147.7, 136.7, 134.8, 134.1, 133.7, 132.9, 130.4, 128.76, 128. 73, 128.6, 128.5, 128.23, 128.19, 125.7, 67.5, 66.8, 56.7, 40.6, 32.8, 29.3, 22.2; IR (neat) v_{max} 1719, 1541, 1455, 1355, 1260, 1170, 1124 cm^{-1}; HRMS (ESI): $[M+H]^+$ calculated for $C_{27}H_{30}N_3O_8S$: 556.1748, found: 556.1744; $[\alpha]_D^{21}$ – 90.3 (*c* 2.7, $CHCl_3$); R_f = 0.30 (PE/EA = 5/3).

Compound **19b**

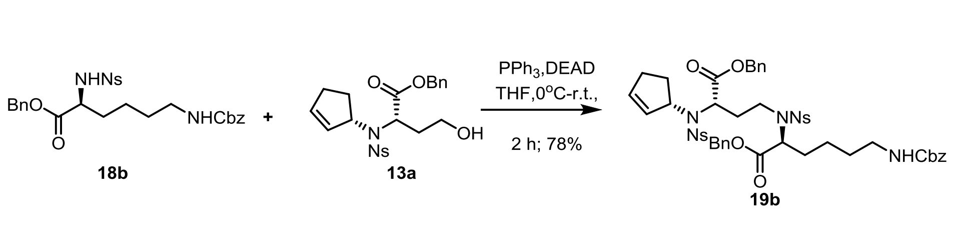

PPh_3 (170 mg, 0.65 mmol, 1.5 eq) was dissolved in THF (2 mL) and to this solution was added DEAD (100 μL, 0.65 mmol, 1.5 eq) at 0 ℃ under argon. The mixture was stirred for 10 minutes, then a solution of **18b** (290 mg, 0.52 mmol, 1.2 eq) in THF (2 mL) was added. A solution of **13a** (200 mg, 0.43 mmol, 1.0 eq) in THF (1 mL) was added into the solution. After stirring for 2 h, the mixture was evaporated *in vacuo*, and the residue was purified by silica gel column chromatography (DCM/EA = 15/1) to afford the desired product (404 mg, 0.41 mmol, 78%) as colorless oil.

^1H NMR (400 MHz, $CDCl_3$) δ 8.04 (dd, *J* = 8.2, 1.5 Hz, 1H), 7.89 (dd, *J* = 7.9, 1.4 Hz, 1H), 7.61 – 7.57 (m, 1H), 7.55 – 7.46 (m, 3H), 7.45 – 7.28 (m, 13H), 7.17 (dd, *J* = 6.6, 3.0 Hz, 2H), 6.01 (dd, *J* = 5.8, 2.3 Hz, 1H), 5.58 (dd, *J* = 5.8, 2.3 Hz, 1H), 5.15 – 4.94 (m, 5H), 4.87 – 4.83 (m, 2H), 4.74 – 4.68 (m, 1H), 4.62 (dd, *J* = 10.4, 4.5 Hz, 1H), 4.38 (t, *J* = 6.8 Hz, 1H), 3.75 – 3.62 (m, 1H), 3.43 – 3.27 (m, 1H), 3.18 – 3.10 (m, 1H), 2.66 – 2.60 (m, 1H), 2.52 – 2.46 (m, 1H), 2.39 – 2.32 (m, 3H), 2.10 – 2.00 (m, 2H), 1.80 – 1.70 (m, 1H), 1.53 – 1.41 (m, 4H); ^{13}C NMR (126 MHz, $CDCl_3$) δ 168.0, 167.8, 153.9, 145.9, 145.4, 134.2, 132.3, 132.3, 132.0, 130.9, 130.8, 129.6, 128.9, 128.8, 128.7, 128.3, 126.9, 126.09, 126.07, 126.0, 125.96, 125.94, 125.9, 125.5, 125.4, 121.4, 121.2, 65.3, 64.8, 64.0, 63.9, 58.5, 42.7, 38.2, 31.3, 29.1, 27.3, 27.2, 26.9, 26.5, 20.8; IR (neat) v_{max} 2925, 1739, 1545, 1456, 1374, 1166 cm^{-1}; HRMS (ESI): $[M+H]^+$ calculated for $C_{49}H_{52}N_5O_{14}S_2$: 998.2947, found: 998.2934; $[\alpha]_D^{21}$ – 6.2 (*c* 0.3, $CHCl_3$); R_f = 0.35 (PE/EA = 5/3).

Compound **20b**

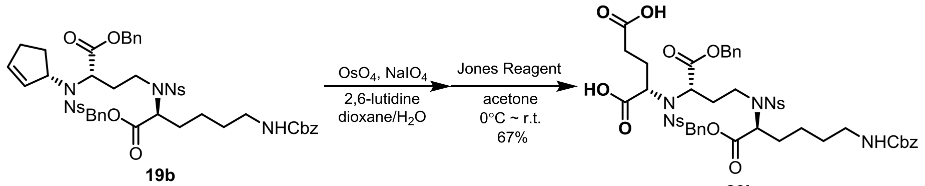

Compound **19b** (281 mg, 0.28 mmol, 1 eq) was dissolved in dioxane (18.6 mL) and H_2O (9.3 mL). To this solution was added 2,6–lutidine (130 µL, 1.12 mmol, 4 eq), OsO_4 (0.01M in *t*-BuOH) (5.6 mL, 0.056 mmol, 0.2 eq) and $NaIO_4$ (600 mg, 2.81 mmol, 10 eq) at r.t.. After stirring for 2 h at r.t., the resulting solution was quenched by saturated $Na_2S_2O_3$ solution (20 mL) and extracted by EA (20 mL x 3) and the combined organic extracts were washed with saturated $Na_2S_2O_3$ solution (15 mL x 2), 5% NaH_2PO_4 solution (15 mL x 2) and brine, dried over anhydrous Na_2SO_4 and the filtrate was concentrated *in vacuo*. The residue was directly used in next step without further purification. The residue was dissolved in acetone (14 mL) and Jones reagent (2 M, 309 µL, 0.62 mmol, 2.2 eq) was added to the solution at 0 ℃ and the mixture was allowed to stir for 2 h at r.t. before quenched by *i*–PrOH (1 mL). The mixture was diluted by EA (20 mL) and washed by saturated $Na_2S_2O_3$ solution (10 mL x 2) and brine (10 mL). The aqueous phase was extracted by EA (5 mL). The organic phase was dried over anhydrous Na_2SO_4 the filtrate was concentrated *in vacuo*. The residue was purified by silica gel column chromatography to afford pure product **20b** (199 mg, 0.19 mmol, 67%) as white solid. Single crystals with X-ray diffraction quality were grown by dissolving the material in $CDCl_3$. The mixture was lightly capped and the solvents were allowed to slowly evaporate.
^1H NMR (400 MHz, $CDCl_3$) δ 8.20 – 8.12 (m, 1H), 8.10 – 8.05 (m, 1H), 7.75 – 7.48 (m, 5H), 7.42 – 7.21 (m, 16H), 7.20 – 7.12 (m, 2H), 6.89 – 6.84 (m, 1H), 5.34 – 4.75 (m, 8H), 4.70 – 4.60 (m, 1H), 4.49 (dd, *J* = 10.0, 3.8 Hz, 1H), 3.90 – 3.64 (m, 1H), 3.28 – 3.10 (m, 3H), 2.95 (t, *J* = 12.9 Hz, 1H), 2.70 – 2.50 (m, 4H), 2.32 (td, *J* = 8.1, 4.2 Hz, 1H), 2.23 – 2.12 (m, 1H), 1.89 – 1.78 (m, 1H), 1.68 – 1.60 (m, 1H), 1.57 – 1.30 (m, 4H); ^{13}C NMR (126 MHz, $CDCl_3$) δ 177.5, 175.5, 173.6, 170.4, 158.9, 148.1, 147.8, 136.0, 134.9, 134.4, 134.0, 133.5, 133.2, 132.4, 132.3, 131.6, 131.5, 131.4, 129.0, 128.9, 128.8, 128.6, 128.54, 128.51, 128.47, 128.2, 128.1, 124.4, 123.8, 72.8, 69.0, 67.6, 61.4, 59.0, 45.2, 41.6, 32.1, 30.0, 29.7, 28.1, 26.9, 23.8; IR (neat) v_{max} 2931, 1737, 1544, 1372, 1155, 755 cm^{-1}; HRMS (ESI): $[M-H]^-$ calculated for $C_{49}H_{50}N_5O_{18}S_2$: 1060.2598, found: 1060.2641; $[\alpha]_D^{22}$ – 14.2 (*c* 1.5, $CHCl_3$); R_f =0.35 (DCM/MeOH = 10/1); m.p. = 102-105 ℃.

Compound **15b**

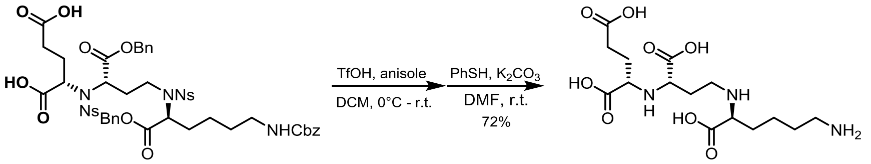

To a solution of diacid **20b** (40 mg, 0.038 mmol, 1 eq) in DCM (3.8 mL) was added anisole (61 *µ*L, 0.564 mmol, 15 eq) and trifluoromethanesulfonic acid (33 *µ*L, 0.380 mmol, 10 eq) at 0 ℃. The reaction mixture was stirred at 0 ℃ for 0.5 h, then warmed to room temperature, and stirred for 2 h. The reaction was quenched with a solution of $NaHCO_3$ (32 mg, 0.380 mmol, 10 eq) in water (5 mL) at 0 ℃ and stirred for an additional 0.5 h. The mixture was washed with DCM (10 mL x 3) and the water phase was concentrated *in vacuo* and directly used for the next step without further purification. The crude mixture and K_2CO_3 (42 mg, 0.301 mmol, 8 eq) were dissolved in dry DMF (2 mL) and to this solution was added thiophenol (77 *µ*L, 0.752 mmol, 20 eq), and the reaction was stirred for 14 h at room temperature before water (10 mL) was added and washed with DCM (10 mL x 3). Then the water phase was concentrated *in vacuo*. The resulting solid was purified by gel column to afford **15b** (10 mg, 0.027 mmol, 72 %) as a white solid.
^1H NMR (400 MHz, D_2O) δ 3.55 (t, *J* = 6.0 Hz, 1H), 3.36 – 3.21 (m, 3H), 3.11 – 2.88 (m, 3H), 2.40 – 2.17 (m, 2H), 2.12 – 1.81 (m, 6H), 1.76 – 1.63 (m, 2H), 1.53 – 1.34 (m, 2H); ^{13}C NMR (201 MHz, D_2O) δ 182.1, 174.9, 62.6, 62.6, 62.3, 46.7, 39.0, 33.9, 30.0, 29.2, 28.6, 26.4, 21.4; IR (neat) v_{max} 3381, 2945, 1577, 1399, 1033 cm^{-1}; HRMS (ESI): $[M+H]^+$ calculated for $C_{15}H_{28}N_3O_8$: 378.1871, found: 378.1858;
$[\alpha]_D^{22}$ + 7.6 (*c* 0.65, H_2O); m.p.>330°C.

Compound **18c**

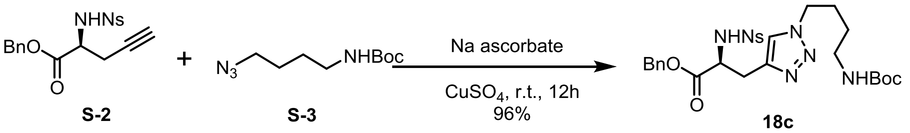

To a mixture of **S-2** (753 mg, 1.94 mmol, 1 eq) in THF (20 mL) and H_2O (20 mL) was added **S-3** (622 mg, 2.91 mmol, 1.5 eq), $CuSO_4{\cdot}5H_2O$ (1.46 g, 5.82 mmol, 3 eq) and Na ascorbate (1.15 g, 5.82 mmol, 3 eq) at r.t.. After stirring for 2 h, THF was removed, and the residue was diluted with EA (100 mL). The combined organic phases were washed with brine (20 mL x 2), dried with Na_2SO_4, and evaporated under *in vacuo*. The residue was further purified by silica gel column chromatography (DCM/EA = 10/1) to afford the desired product (1.12 g, 1.86 mmol, 96%) as colorless oil.

^{1}H NMR (400 MHz, $CDCl_3$) δ 8.04 – 7.93 (m, 1H), 7.76 – 7.71 (m, 1H), 7.64 – 7.55 (m, 2H), 7.31 – 7.25(m, 3H), 7.17 – 7.13(m, 2H), 6.66 (d, *J* = 8.8 Hz, 1H), 4.96 – 4.88 (m, 2H), 4.70 – 4.62 (m, 1H), 4.57 (dt, *J* = 8.8, 5.6 Hz, 1H), 4.27 (t, *J* = 7.0 Hz, 2H), 3.34 – 3.26 (m, 2H), 3.13 – 3.08 (m, 2H), 1.93 – 1.75 (m, 2H), 1.45 – 1.35 (m, 11H);

^{13}C NMR (101 MHz, $CDCl_3$) δ 169.9, 156.0, 147.4, 134.7, 134.1, 133.4, 132.7, 130.2, 128.5, 128.4, 125.4, 122.4, 79.1, 67.5, 56.1, 49.8, 39.6, 29.4, 28.3, 27.3, 26.9; IR (neat) v_{max} 3332, 2927, 1743, 1699, 1540, 1364, 1165, 742 cm^{-1}; HRMS (ESI): $[M+H]^+$ calculated for $C_{27}H_{35}N_6O_8S$: 603.2232, found: 603.2225; $[\alpha]_D^{21}$ – 99.1 (*c* 1, $CHCl_3$); R_f = 0.15 (PE/EA = 1/1).

Compound **19c**

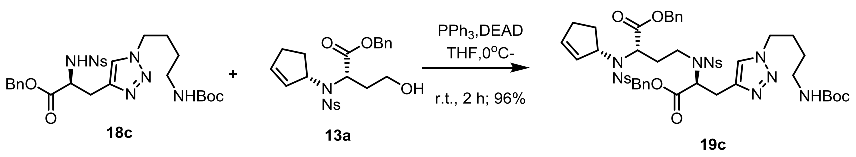

Same procedure as the synthesis of **5a** to afford **19c** as white solid, yield = 96%.

^{1}H NMR (400 MHz, $CDCl_3$) δ 8.06 (d, *J* = 7.7 Hz, 1H), 7.90 (d, *J* = 7.8 Hz, 1H), 7.62 – 7.27 (m, 15H), 7.21 – 7.16 (m, 2H), 5.97 (d, *J* = 3.3 Hz, 1H), 5.53 (d, *J* = 3.4 Hz, 1H), 5.16 – 4.92 (m, 5H), 4.72 – 4.58 (m, 2H), 4.32 (t, *J* = 6.8 Hz, 3H), 3.76 – 3.58 (m, 1H), 3.54 – 3.40 (m, 2H), 3.30 (dd, *J* = 15.8, 10.9 Hz, 1H), 3.11 – 3.07 (m, 2H), 2.62 – 2.50 (m, 2H), 2.30 – 2.14 (m, 3H), 1.95 – 1.81 (m, 3H), 1.39 (s, 11H); ^{13}C NMR (101 MHz, $CDCl_3$) δ 170.6, 169.6, 156.0, 148.4, 147.8, 142.8, 136.7, 134.9, 134.7, 134.2, 133.6, 133.3, 132.1, 131.8, 131.6, 131.5, 131.14, 131.05, 129.2, 128.59, 128.57, 128.53, 128.50, 128.5, 128.4, 123.8, 123.7, 122.4, 79.0, 67.8, 67.6, 66.2, 60.8, 57.6, 49.8, 45.2, 39.7, 33.6, 31.5, 29.1, 28.3, 27.4, 27.2, 26.9; IR (neat) v_{max} 3417, 2928, 1739, 1706, 1543, 1370, 1163, 750 cm^{-1}; HRMS (ESI): $[M+H]^+$ calculated for $C_{49}H_{57}N_8O_{14}S_2$: 1045.3430, found: 1045.3425; $[\alpha]_D^{21}$ – 13.8 (*c* 1, $CHCl_3$); R_f = 0.30 (PE/EA = 1/2); m.p. = 65-68 ℃.

Compound **20c**

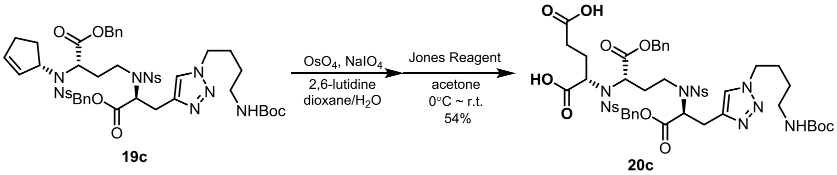

Same procedures as the synthesis of **14a** to afford **20c** as a white solid, yield = 54% for 2 steps.

^{1}H NMR (400 MHz, CD_3CN) δ 8.13 (d, *J* = 7.9 Hz, 1H), 7.84 (d, *J* = 7.6 Hz, 1H), 7.76 – 7.68 (m, 1H), 7.68 –7.60 (m, 4H), 7.58 – 7.52 (m, 2H), 7.38 – 7.26 (m, 8H), 7.19 – 7.13 (m, 2H), 5.38 – 5.28 (m, 1H), 5.17 – 4.79 (m, 5H), 4.62 (dd, *J* = 9.8, 3.9 Hz, 1H), 4.52 – 4.42 (m, 1H), 4.31 (t, *J* = 6.8 Hz, 2H), 3.66 – 3.58 (m, 1H), 3.43 – 3.35 (m, 1H), 3.35 – 3.25 (m, 1H), 3.18 – 3.09 (m, 1H), 3.05 – 2.95 (m, 2H), 2.47 – 2.21 (m, 5H), 1.84 – 1.74 (m, 3H), 1.38 – 1.33 (m, 11H); ^{13}C NMR (101 MHz, CD_3CN) δ 174.5, 172.0, 171.5, 170.4, 157.3, 149.2, 148.7, 143.5, 136.1, 136.0, 135.7, 135.4, 133.2, 132.8, 132.1, 132.0, 131.6, 129.7, 129.63, 129.56, 129.5, 129.4, 129.3, 125.0, 124.9, 124.3, 79.4, 68.9, 68.3, 62.4, 59.4, 58.3, 50.7, 46.1, 40.3, 33.2, 31.4, 28.6, 28.0, 27.6, 27.4, 26.9; IR (neat) v_{max} 2925, 2852, 1731, 1544, 1370, 1261, 1165, 754 cm^{-1}; HRMS (ESI): $[M+H]^+$ calculated for $C_{49}H_{57}N_8O_{18}S_2$: 1109.3227, found: 1109.3195; $[\alpha]_D^{21}$ + 7.4 (*c* 1, $CHCl_3$); m.p. = 112 - 115 ℃.

Compound **15c**

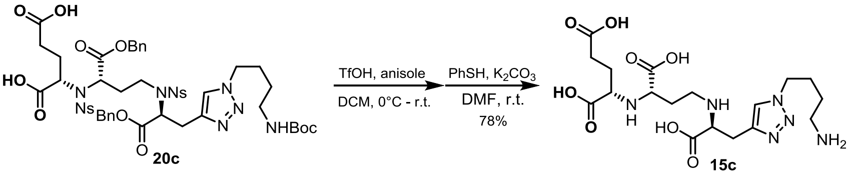

Same procedures as the synthesis of **2** to afford **15c** as a white solid, yield = 78% for 2 steps.

^{1}H NMR (400 MHz, D_2O) δ 7.85 (s, 1H), 4.45 (t, *J* = 6.5 Hz, 2H), 3.66 (t, *J* = 5.8 Hz, 1H), 3.38 (dd, *J* = 9.0, 4.1 Hz, 1H), 3.33 (t, *J* = 5.9 Hz, 1H), 3.27 – 3.11 (m, 3H), 3.02 – 2.91 (m, 3H), 2.37 – 2.23 (m, 2H), 1.97 (dt, *J* = 15.5, 7.8 Hz, 6H), 1.59 (d, *J* = 7.6 Hz, 2H); ^{13}C NMR (126 MHz, D_2O) δ 181.9, 177.4, 176.8, 176.3, 143.0, 124.3, 62.9, 62.7, 62.6, 49.6, 46.2, 38.8, 33.9, 29.0, 28.5, 27.5, 26.4, 23.7;

IR (neat) ν_{max} 3411, 1554, 1397, 1102, 996, 829 cm^{-1}; HRMS (ESI): $[M+H]^+$ calculated for $C_{18}H_{31}N_6O_8$: 459.2198, found: 459.2198; $[\alpha]_D^{21}$ + 9.8 (*c* 0.5, H_2O); m.p. > 330℃.

Compound **16**

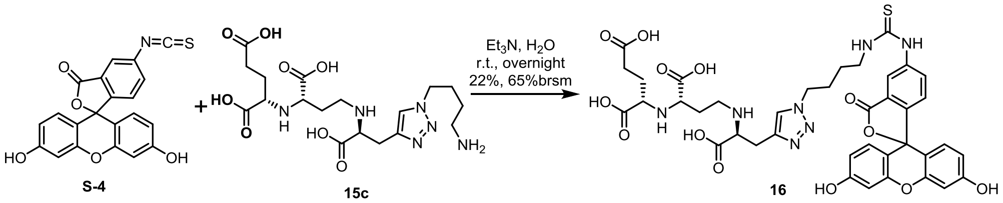

Scheme S6: Syntheses of the pseudopaline-fluorescein probe.

To a solution of **15c** (5.6 mg, 12.3 μmol, 1 eq) in water (1 mL) was added Et_3N (8.5 *μ*L, 61.5 μmol, 5 eq) and **S-4** (4.8 mg, 12.3 μmol, 1 eq) at r.t.. After stirring for overnight at r.t., The reaction mixture was subjected directly to prep-HPLC (20% acetonitrile in 0.02% HCl in H_2O for 3 min then a gradient of 20% – 90% acetonitrile in 0.02% HCl in H_2O for 10 min) to afford the pure product **16** (2.3 mg, 2.71 *μ*mol, 22%) and the recovered **15c** (2 mg).

^1H NMR (400 MHz, D_2O) δ 8.27 (s, 1H), 7.97 (s, 1H), 7.78 (d, *J* = 8.4 Hz, 1H), 7.62 (d, *J* = 9.8 Hz, 2H), 7.41 (d, *J* = 8.1 Hz, 1H), 7.35 (d, *J* = 2.0 Hz, 2H), 7.17 (dd, *J* = 9.2, 2.0 Hz, 2H), 4.48 (t, *J* = 5.6 Hz, 2H), 4.36 (d, *J* = 5.7 Hz, 1H), 4.12 (t, *J* = 6.4 Hz, 1H), 4.08 (t, *J* = 6.4 Hz, 1H), 3.64 – 3.56 (m, 2H), 3.50 – 3.38 (m, 4H), 2.72 – 2.57 (m, 2H), 2.48 – 2.33 (m, 2H), 2.29 – 2.16 (m, 2H), 2.03 – 1.93 (m, 2H), 1.65 – 1.56 (m, 2H); ^{13}C NMR (239 MHz, D_2O/ CD_3CN) δ 180.8, 176.2, 171.3, 170.8, 170.2, 168.7, 166.0, 156.7, 141.0, 140.7, 131.8, 129.8, 129.6, 128.5, 124.9, 117.3, 114.4, 102.9, 100.0, 62.8, 60.3, 60.0, 59.0, 50.3, 44.0, 29.9, 27.1, 26.6, 25.5, 25.2, 25.1; IR (neat) ν_{max} 3209, 2292, 2851, 1608, 1393, 1115 cm^{-1}; HRMS (ESI): $[M+H]^+$ calculated for $C_{39}H_{40}N_7O_{13}S$: 846.2410, found: 846.2440; $[\alpha]_D^{21}$ + 18.0 (*c* 0.05, H_2O/ACN); m.p. > 330℃.

III) Comparison of the synthetic and natural Fmoc-pseudopaline

Table S1:
^{1}H NMR Comparison of the synthetic and natural Fmoc-pseudopaline

^{1}H NMR of Fmoc–pseudopaline	
Natural Fmoc–pseudopaline	Synthetic Fmoc–pseudopaline
7.92 (t, *J* = 7.2, 3H)	7.92 (t, *J* = 7.2, 3H)
7.73 (d, *J* = 7.2, 2H)	7.72 (d, *J* = 7.2, 2H)
7.49 (t, *J* = 7.2, 3H)	7.48 (t, *J* = 7.2, 3H)
7.41 (t, *J* = 7.2, 3H)	7.42 (t, *J* = 7.2, 3H)
7.20 (s, 1H)	7.20 (s, 1H)
4.94 (d, *J* = 5.2, 2H)	4.92 (d, *J* = 5.2, 2H)
4.50 (t, *J* = 5.2, 1H)	4.49 (t, *J* = 5.2, 1H)
3.90 (t, *J* = 5.6, 1H)	3.90 (t, *J* = 5.6, 1H)
3.66–3.60 (m, 2H))	3.65–3.59 (m, 2H))
3.25 (t, *J* = 7.2, 2H)	3.24 (t, *J* = 7.2, 2H)
3.10 (d, *J* = 5.2, 2H)	3.09 (d, *J* = 5.2, 2H)
2.46 (t, *J* = 7.2, 2H)	2.38 (t, *J* = 7.2, 2H)
2.32–2.24 (m, 2H)	2.30–2.21 (m, 2H)
2.15 –2.09 (m, 2H)	2.10 –2.08 (m, 2H)

Table S2:
^{13}C NMR Comparison of the synthetic and natural Fmoc-pseudopaline

^{13}C NMR of Fmoc–pseudopaline	
Natural Fmoc–pseudopaline	Synthetic Fmoc–pseudopaline
180.2	180.3
173.2	173.2
172.2	172.2
172.0	172.0
149.0	149.0
143.1	143.1
141.0	141.0
138.0	138.0
136.2	136.2
128.2	128.2
127.5	127.5
125.0	125.0
120.2	120.2
115.6	115.6
69.4	69.4
62.8	62.8
61.6	61.6
59.9	59.8
46.4	46.4
43.5	43.5
32.7	32.8
27.4	27.4
26.8	26.8
26.4	26.5

Table S3:
$[\alpha]_D^{20}$ Comparison of the synthetic and natural Fmoc-pseudopaline

	Natural Fmoc–pseudopaline	Synthetic Fmoc–pseudopaline
$[\alpha]_D^{20}$	+15 (*c* 0.1, H_2O)	+18 (*c* 0.1, H_2O)

^{1}H NMR Comparison of synthetic and natural Fmoc-pseudopaline

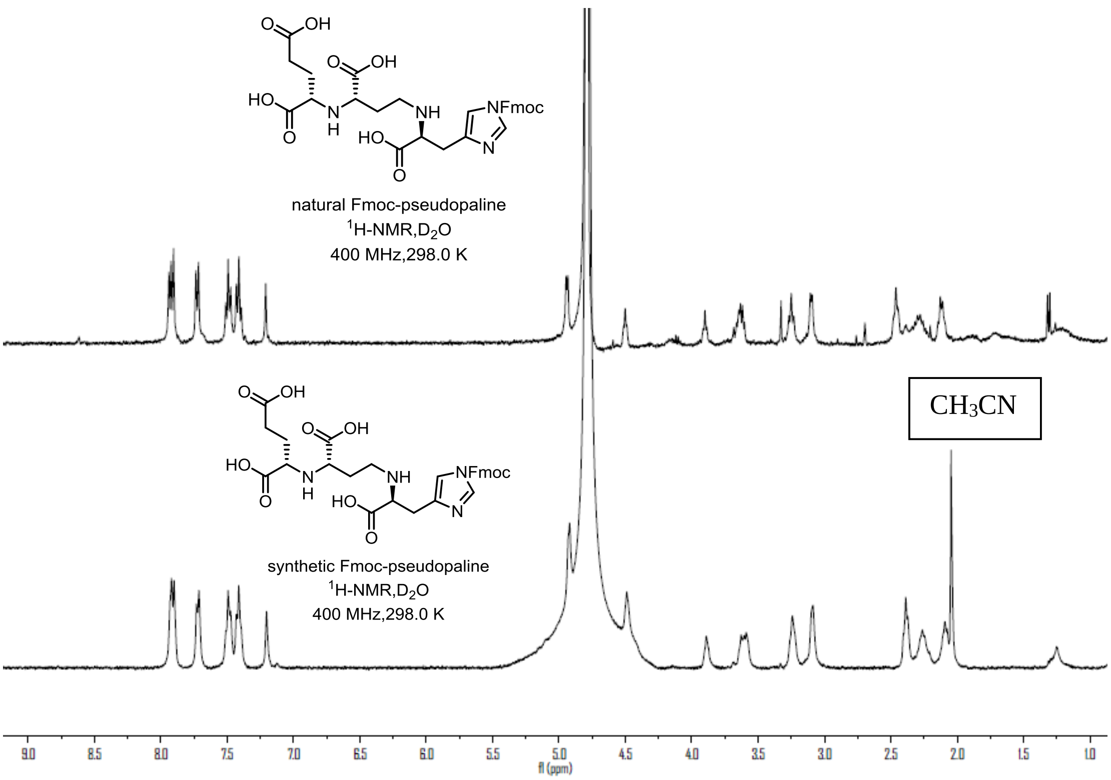

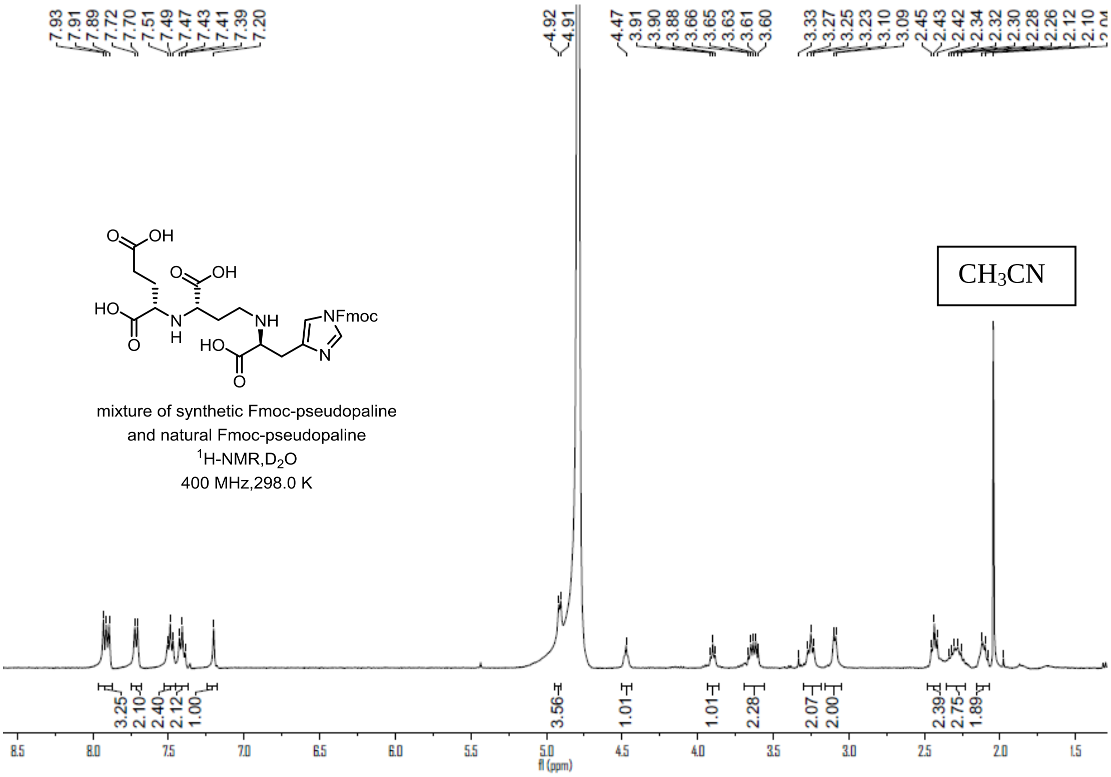

^{13}C NMR Comparison of synthetic and natural Fmoc-pseudopaline

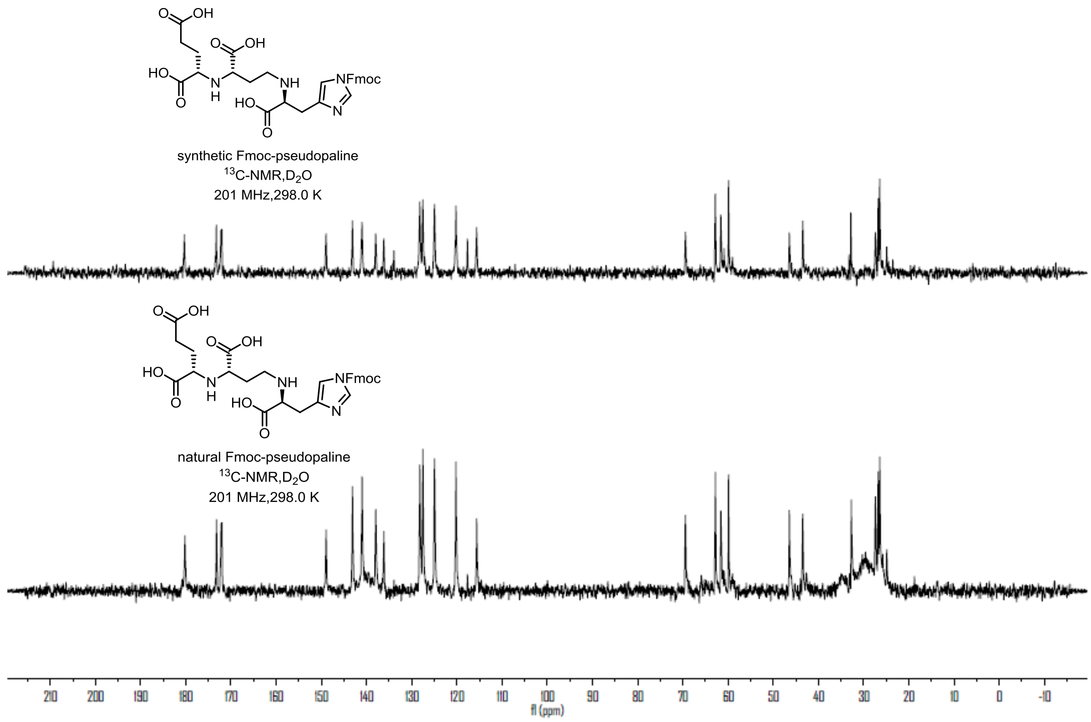

HRMS Comparison of synthetic and natural Fmoc-pseudopaline

$[M+H]^+$

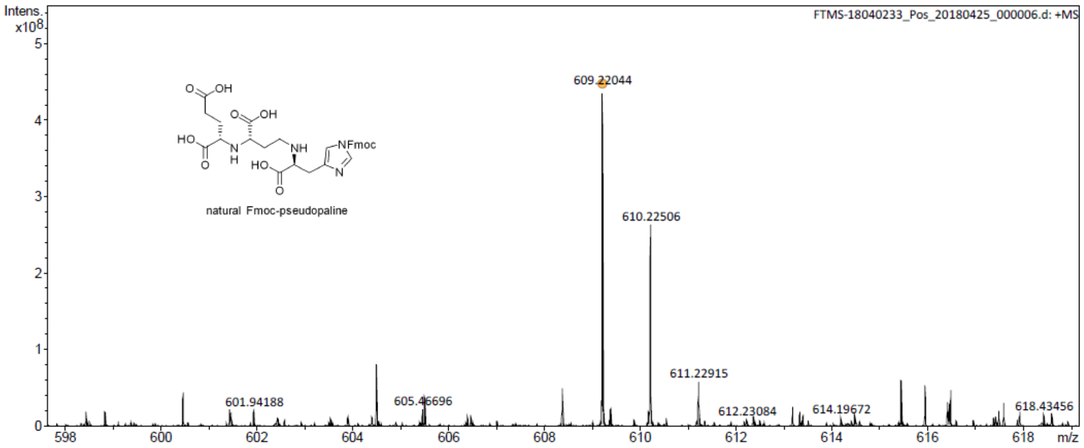

Meas. m/z	#	Ion Formula	Score	m/z	err [ppm]	Mean err [ppm]	mSigma	rdb	e⁻ Conf	N-Rule
609.220445	1	C30H33N4O10	100.00	609.219120	-2.2	-4.1	128.7	17.0	even	ok

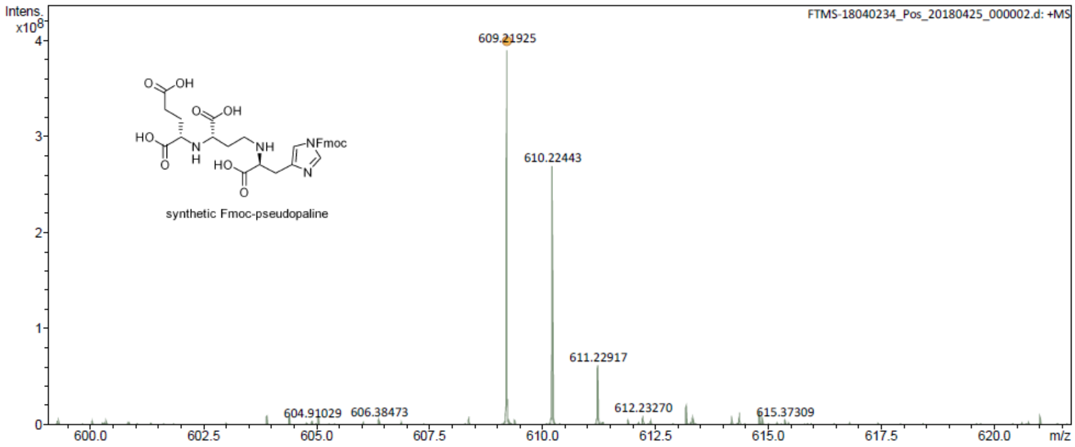

Meas. m/z	#	Ion Formula	Score	m/z	err [ppm]	Mean err [ppm]	mSigma	rdb	e⁻ Conf	N-Rule
609.219247	1	C30H33N4O10	100.00	609.219120	-0.2	-3.5	172.9	17.0	even	ok

$[M-H]^{-}$

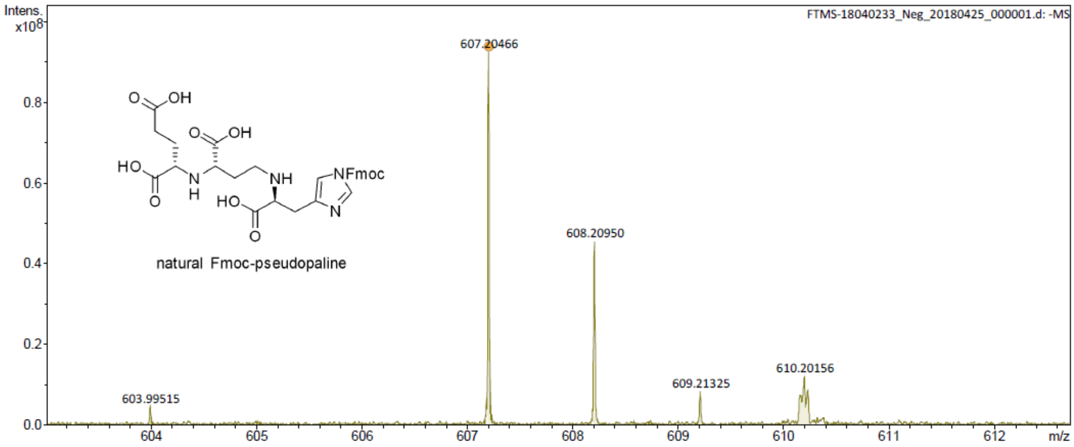

Meas. m/z	#	Ion Formula	Score	m/z	err [ppm]	Mean err [ppm]	mSigma	rdb	e⁻ Conf	N-Rule
607.204664	1	C30H31N4O10	100.00	607.204567	-0.2	-1.6	74.0	17.0	even	ok

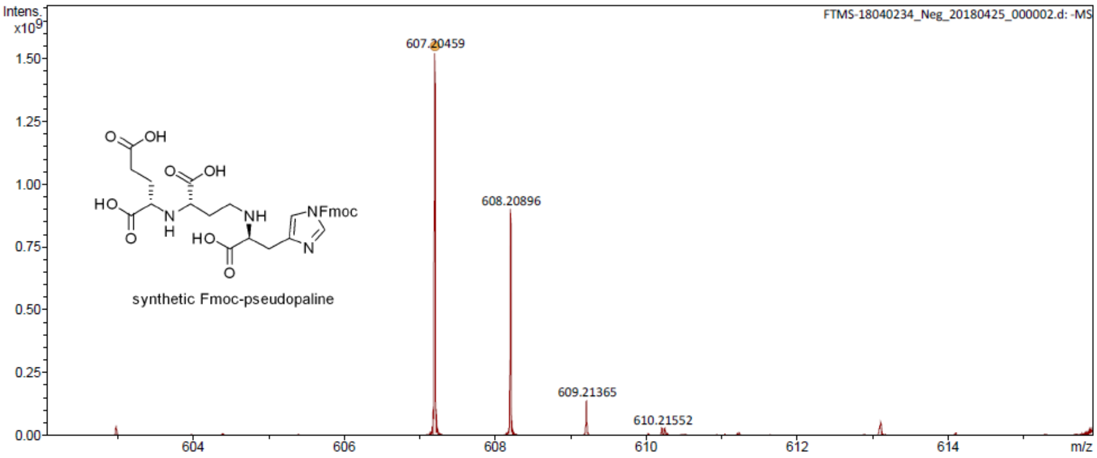

Meas. m/z	#	Ion Formula	Score	m/z	err [ppm]	Mean err [ppm]	mSigma	rdb	e⁻ Conf	N-Rule
607.204588	1	C30H31N4O10	100.00	607.204567	-0.0	-2.2	122.0	17.0	even	ok

IV) Biological Evaluations

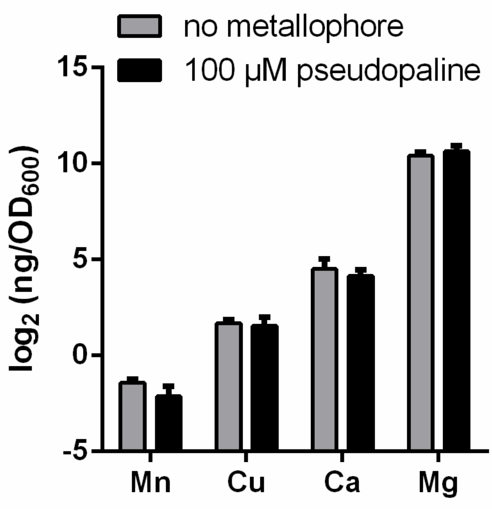

Fig. S1 Pseudopaline had no influence on the uptake of Mn^{2+}, Cu^{2+}, Ca^{2+} and Mg^{2+} in *P. aeruginosa*. Intracellular manganese, copper, magnesium or calcium contents of wild type *P. aeruginosa* grown in VBMM containing corresponding metal and 50 μM EDTA and treated with or without 100 μM pseudopaline was measured by ICP-MS from three independent trials. Error bars, mean ± SD.

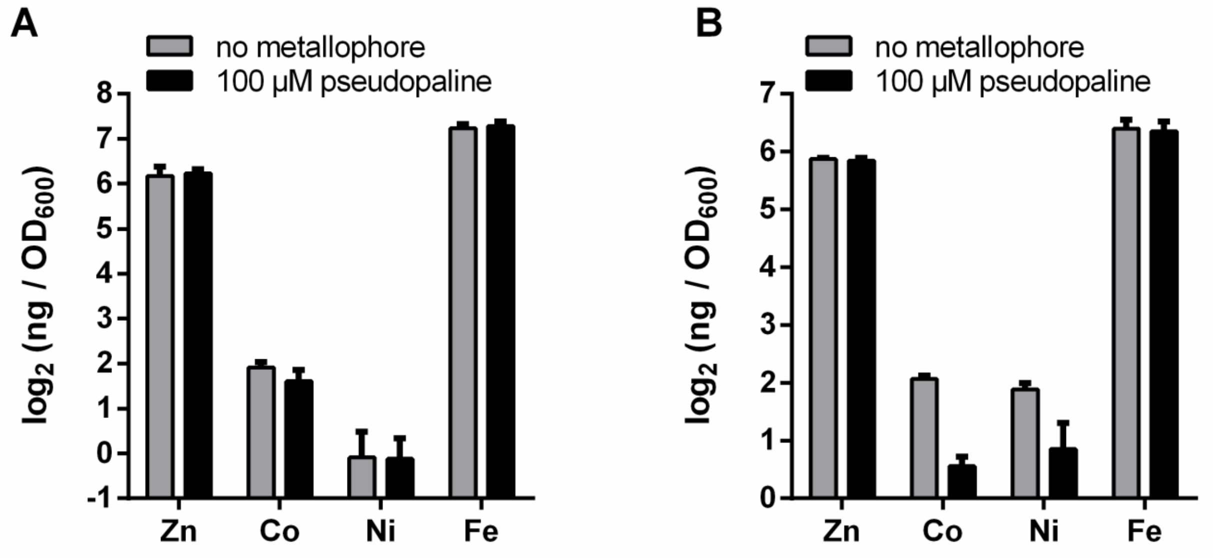

Fig. S2 Pseudopaline could not promote metal uptake in nutrient-rich LB medium (A) or in the VBMM medium containing the corresponding metal but without EDTA (B). Intracellular zinc, cobalt, nickel or iron contents of wild type *P. aeruginosa* treated with or without 100 μM pseudopaline was measured by ICP-MS from three independent trials. Error bars, mean ± SD.

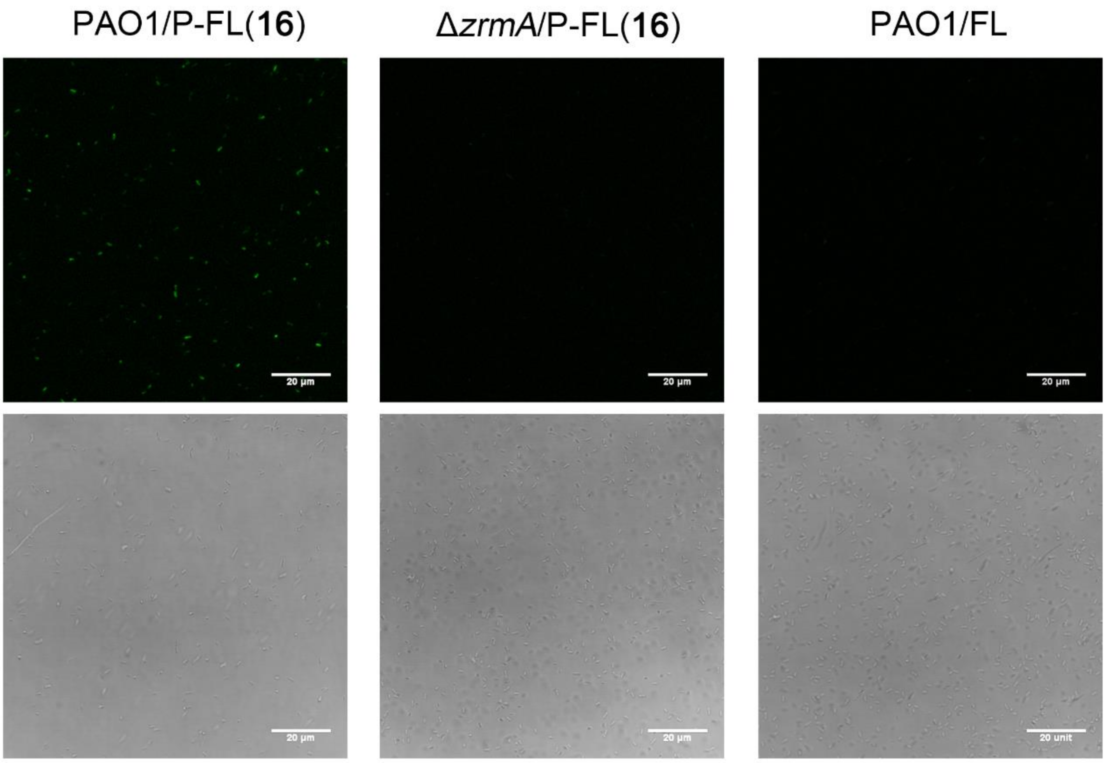

Fig. S3 Pseudopaline-fluorescein conjugate (P-FL 16) could be transported into *P. aeruginosa*. Fluorescence microscopy images of PAO1 and Δ*zrmA* treated with pseudopaline-fluorescein (P-FL **16**), and PAO1 treated with fluorescein (FL) for 15 min. The top pictures were taken at: ex. 493±20 nm/em. 516±20 nm. The bottom pictures were taken by light microscopy (bright phase). Scale bar: 20 μm.

Bacterial strain, growth condition and strain construction

Unless indicated, *P. aeruginosa* and *E. coli* were grown at 37 °C in LB medium. Gentamycin was used at 10 μg/ml for *E. coli*, while gentamycin was used at 30 μg/ml, and irgasan at 25 μg/ml for *P. aeruginosa*.

The pseudopaline receptor mutated strain Δ*zrmA* was constructed according to Battistoni's paper.[2] An unmarked, nonpolar deletion strategy was used. Simply, the upstream and downstream regions of *zrmA* were amplified by primers Up-F (GCTCGGTACCCGGGGATCCGAAATGCAGCGGATCGAGC), Up-R (TAGTTCAGGGGGGAAATCGCACCAGAAAAG), and Dw-F (GCGATTTCCCCCCTGAACTACTGAGCCCTC), Dw-R (GACGGCCAGTGCCAAGCTTGAAGCGATCAGGAAATGCGT), overlapped, and ligated in plasmid pEX18Gm.[3] The recombinant plasmid was transferred to PAO1 by mating, and a double-crossover recombinant was isolated as previously described.[3] This generated strain Δ*zrmA*, in which 1-2113 bp were deleted.

Inductively coupled plasma mass spectrometry (ICP-MS)

The intracellular metal concentration was detected according to Battistoni's method with slight modification.[2] Overnight *P. aeruginosa* culture was collected, washed with PBS twice, diluted in the VBMM medium containing 10 μM $ZnSO_4$, a cocktail of transition metals in trace amounts (0.1 μM $FeSO_4$, $NiSO_4$, $CoSO_4$, $CuSO_4$ and $MnSO_4$) or 1 μM $MgSO_4$ and $CaCl_2$ and supplied with 50 μM EDTA. The intracellular metal contents were detected with or without 100 μM pseudopaline or *epi*-pseudopaline. The intracellular metal contents were also detected in LB medium with 50 μM EDTA or VBMM medium without EDTA. The effect of pseudopaline analog **15a**, **15b**, or **15c** on metal uptake was detected in VBMM medium containing 10 μM $ZnSO_4$ and 50 μM EDTA. After grown for 18 h at 37 °C, 10 mL of bacterial cultures were collected, centrifuged at 8000 rpm for 5 min and then the pellet was washed with 10 mL of PBS with 1 mM EDTA twice and 10 mL PBS once to remove excess metals. After the OD_{600} was measured, the cell pellet was dried overnight at 95 °C. The metal contents were determined by re-suspending the dried pellet with 5 mL absolute HNO_3, heated at 140 °C for 3 h and then diluted with 15 mL water. The metal contents were analyzed by ICP-MS (PerkinElmer NexION^{TM} 350×) operating in argon collision gas mode to remove possible interferences. The intracellular metal concentration was normalized by OD_{600}.

Fluorescence Microscopy

The uptake of pseudopaline-fluorescein **16** was detected according to Mislin's method with slight modification.[4] Overnight culture of wild type *P. aeruginosa* PAO1 and pseudopaline receptor mutated Δ*zrmA* were collected, washed with PBS twice, re-suspended in PBS, 100 fold diluted into VBMM medium, and grown at 37 °C for 6 h with shaking. The pseudopaline-fluorescein **16** and fluorescein (5-Carboxyfluorescein) were first incubated with zinc at a ratio of 1: 1 for 15 min at room temperature before addition to the cells. The zinc complexes of the pseudopaline-fluorescence **16** or fluorescence molecule were then added to 1 mL aliquots of bacterial culture to obtain a 10 μM final concentration. The mixtures were then incubated at 37 °C for 15 min. Cells were pelleted, washed with PBS for five times, re-suspended in PBS and then the fluorescence intensity of the bacteria was detected using Infinite F200 PRO Reader

(TECAN) (ex. 485 nm; em. 535 nm). And the images of fluorescence microscopy were acquired on a Leica TCS SP8× with 100x oil immersion lens using the following filter set: ex. 493±20 nm and em. 516±20 nm.

V) Chelation Experiments

The Metal-Chelation experiments were established referring to P. Arnoux and co-workers' work[1] and our previous work[5]. Fe(III) has a poor affinity for staphylopine at neutral pH reported in the same work[1], so we just tested the affinity of Cu(II), Co(II), Ni(II), Zn(II), Mn(II) and Fe(II). And the same mass-response pattern was observed in ESI-MS tests which suggested the metal(II)-chelators were maintained in the whole processes, and no oxidation reaction was observed by ESI-MS, especially for Fe(II).

We also tested the ion intensity of apo-ligands and holo-ligands to establish maximize ion intensities prior to doing the competition and found they shared the similar ion intensity (always the level from 10^8 to 10^9) by ESI-MS under the conditions we had screened. Only the $[M+H]^+$ ions were collected to estimate the pK_d values.

1) Stability constant test

The Ion intensities (measured by MS) of different metal-ligand complexes have strongly positive correlation to the concentrations of chelation complexes, which allows us to using ESI-MS to estimate the pK_d value of Pseudopaline-metal complexes by pK_d values of staphylopine complexes.[1] Stability constants for Pseudopaline-metal complexes were estimated by metal competition experiments with staphylopine by using ESI-MS. A mixture of Pseudopaline (Pseudopaline, *epi*-Pseudopaline, Pseudopaline analog **15c**) (100 *μ*M, presented as $C_{(PSEU)}$=100 μM), staphylopine (100 *μ*M, presented as $C_{(STA)}$=100 *μ*M) and limited level of metal (80 *μ*M, each metal was tested in a different experiment, presented as $C_{(M)}$=80 *μ*M) to create competition and the different Ion intensity ratios (presented as R_X) were observed by ESI-MS between metal complexes. The equations between equilibrium concentration of different species (presented as [X]) and dissociation constants of ligand – metal complex (presented as $K_{d\ (M\text{-}X)}$) were established to estimate the K_d of Pseu. The equations were listed here:

Dissociation reactions in the mixture of different metal complexes:

$$\text{M-PSEU} = \text{M} + \text{PSEU}$$

$$\text{M-STA} = \text{M} + \text{STA}$$

Equations between C_x and [X]:

$$C_{(M)} = [\text{M-STA}] + [\text{M- PSEU}] + [\text{M}]$$

$$\approx [\text{M-STA}] + [\text{M- PSEU}]$$

$$C_{(PSEU)} = [\text{M- PSEU}] + [\text{PSEU}]$$

$$C_{(STA)} = [\text{M- STA}] + [\text{STA}]$$

Equations between [X] and $K_{d\ (M\text{-}X)}$:

$$R_X = \frac{[\text{M}-\text{STA}]}{[\text{M}-\text{ PSEU}]+[\text{M}-\text{STA}]}$$

$$[\text{M-STA}]=C(M)\cdot R_x,\ [\text{M-PSEU}]=C(M)-[\text{M-STA}]$$

$$[\text{STA}]=C_{(STA)}-[\text{M-STA}],\ [\text{PSEU}]=C_{(PSEU)}-[\text{M-PSEU}]$$

$$K_{d\ (M\text{-}PSEU)} = \frac{[\text{PSEU}][\text{M}]}{[\text{M}-\text{PSEU}]},\ \ K_{d\ (M\text{-}STA)} = \frac{[\text{STA}][\text{M}]}{[\text{M}-\text{STA}]}$$

$$K_{d\ (M\text{ - }PSEU)} = \frac{[\text{PSEU}][\text{M}-\text{STA}]}{[\text{PSEU}-\text{M}][\text{STA}]}\cdot K_{d\ (M\text{ - }STA)}$$

When pseudopaline was replaced by other analogues, the equations still exist.

General Procedures:

A water solution of Metal salts (1mmol/L, 80 μL) was added to a mixture of Pseu solution (100 μL, 1mmol/L), Sta solution (100 μL, 1mmol/L) and then diluted with 720 μL water. The obtained solution was allowed to shake for half an hour at room temperature to get a full chelation to get Pseudopaline-Metal complex and Sta-Metal complex. Then 200 μL of the solution was diluted by 200 μL methanol and 4 *μ*L formic acid, and the mixture was directly injected to the mass spectrum to observe the different Ion intensity ratios of Pseu-Metal complex and Sta-Metal complexes. Each sample was measured for two times.

Results:

Table S4: Ion intensity Data for Pseu-M(II) complexes and Sta-M(II) complexes.

**Metal**	$I_{1(Sta\text{-}M)}$	$I_{1(PSEU\ \text{-}M)}$	$I_{2(Sta\text{-}M)}$	$I_{2(PSEU\text{-}M)}$
Cu^{2+}	1.11×10^{10}	1.91×10^{10}	5.56×10^{9}	1.07×10^{10}
Ni^{2+}	3.88×10^{9}	4.82×10^{9}	3.63×10^{9}	4.93×10^{9}
Co^{2+}	1.80×10^{9}	7.21×10^{9}	1.39×10^{9}	5.24×10^{9}
Zn^{2+}	9.9×10^{8}	2.76×10^{9}	5.85×10^{8}	1.75×10^{9}
Fe^{2+}	2.64×10^{8}	1.15×10^{9}	2.62×10^{8}	1.13×10^{9}
Mn^{2+}	8.82×10^{8}	5.90×10^{9}	5.10×10^{8}	3.52×10^{9}

I: Ion intensity

Table S5: pK_d values of Pseudopaline-metal complexes.

**Metal**	R_{aver}	$pK_{d\ (Sta)}$	$K_{d\ (PSEU)}$	$pK_{d\ (PSEU)}$
Cu^{2+}	0.342	19.0	3.72×10^{-20}	19.42
Ni^{2+}	0.435	16.4	2.58×10^{-17}	16.59
Co^{2+}	0.204	15.1	8.89×10^{-17}	15.1
Zn^{2+}	0.257	15.0	1.77×10^{-16}	15.75
Fe^{2+}	0.197	12.3	7.24×10^{-14}	13.14
Mn^{2+}	0.128	9.1	3.94×10^{-11}	10.40

Table S6: Ion intensity Data for *epi*-Pseudopaline-M(II) and Sta-M(II) complexes.

**Metal**	$I_{1(Sta-M)}$	$I_{1(epi-Pseu\ -M)}$	$I_{2(Sta-M)}$	$I_{2(epi-Pseu-M)}$
Cu^{2+}	1.36×10^{9}	3.26×10^{9}	1.91×10^{9}	4.36×10^{9}
Ni^{2+}	3.41×10^{9}	4.64×10^{9}	2.66×10^{9}	3.66×10^{9}
Co^{2+}	8.24×10^{8}	6.45×10^{8}	8.06×10^{8}	6.04×10^{8}
Zn^{2+}	3.91×10^{8}	2.78×10^{9}	6.15×10^{8}	4.20×10^{9}
Fe^{2+}	4.84×10^{8}	1.09×10^{9}	3.75×10^{8}	8.60×10^{8}
Mn^{2+}	1.37×10^{9}	1.85×10^{9}	6.91×10^{8}	9.87×10^{8}

I: Ion intensity

Table S7: pK_d values of *epi*-Pseudopaline-metal complexes.

**Metal**	R_{aver}	p$K_{d\ (Sta)}$	$K_{d\ (epi-Pseu)}$	p$K_{d\ (epi-Pseu)}$
Cu^{2+}	0.299	19.0	2.47×10^{-20}	19.60
Ni^{2+}	0.422	16.4	2.36×10^{-17}	16.62
Co^{2+}	0.566	15.1	1.24×10^{-15}	14.90
Zn^{2+}	0.589	15.0	1.82×10^{-15}	14.73
Fe^{2+}	0.306	12.3	1.30×10^{-13}	12.89
Mn^{2+}	0.419	9.1	4.60×10^{-10}	9.34

Table S8: Ion intensity Data for **15c**-M(II) complexes and Sta-M(II) complexes.

**Metal**	$I_{1(Sta\text{-}M)}$	$I_{1(\mathbf{I8c}\text{-}M)}$	$I_{2(Sta\text{-}M)}$	$I_{2(\mathbf{18c}\text{-}M)}$
Cu^{2+}	1.20×10^{10}	1.58×10^{10}	4.62×10^{9}	7.28×10^{9}
Ni^{2+}	3.43×10^{9}	7.77×10^{9}	4.77×10^{9}	1.15×10^{10}
Co^{2+}	1.31×10^{9}	3.14×10^{9}	5.28×10^{8}	1.23×10^{9}
Zn^{2+}	9.08×10^{8}	1.80×10^{9}	5.85×10^{8}	1.75×10^{9}
Fe^{2+}	7.73×10^{8}	3.22×10^{9}	4.58×10^{8}	1.93×10^{9}
Mn^{2+}	7.02×10^{8}	2.15×10^{9}	2.58×10^{8}	8.50×10^{8}

I: Ion intensity

Table S9: pK_d values of Pseudopaline-**15c**-metal complexes.

**Metal**	R_{aver}	p$K_{d\ (Sta)}$	$K_{d\ (PSEU)}$	p$K_{d\ (PSEU)}$
Cu^{2+}	0.410	19.0	5.46×10^{-20}	19.26
Ni^{2+}	0.300	16.4	9.9×10^{-18}	17.01
Co^{2+}	0.297	15.1	1.93×10^{-16}	15.72
Zn^{2+}	0.410	15.0	3.34×10^{-16}	15.47
Fe^{2+}	0.193	12.3	5.01×10^{-14}	13.30
Mn^{2+}	0.243	9.1	1.98×10^{-10}	9.70

VI) ^{1}H and ^{13}C NMR spectra

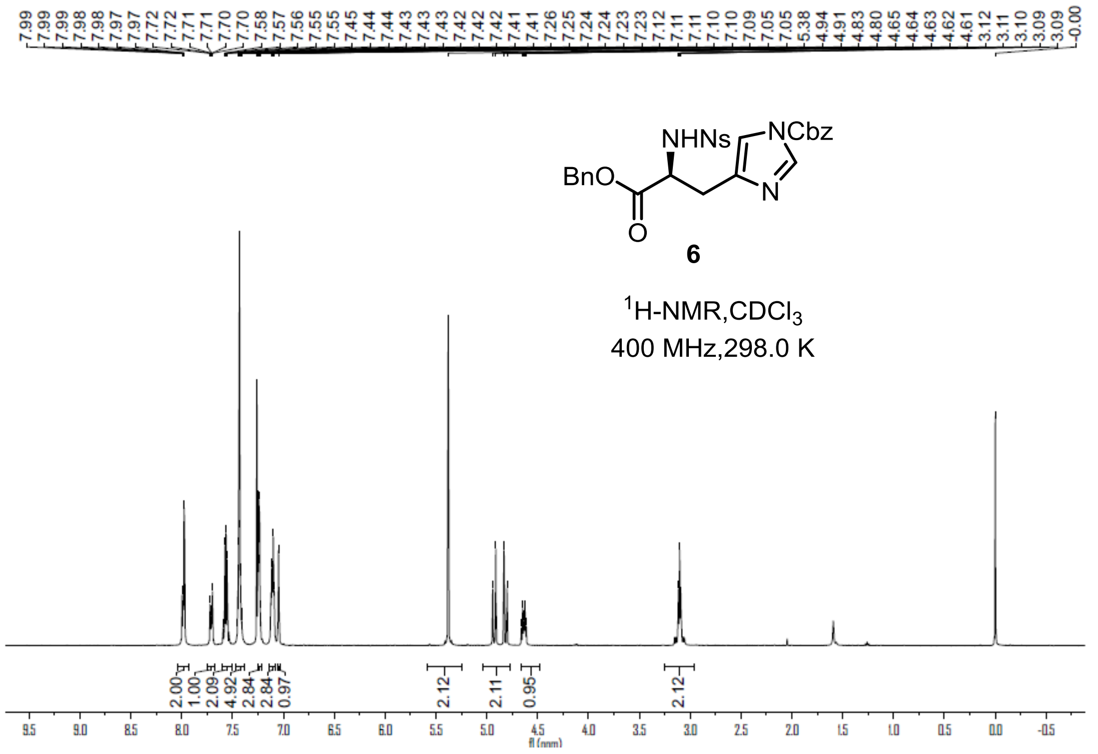

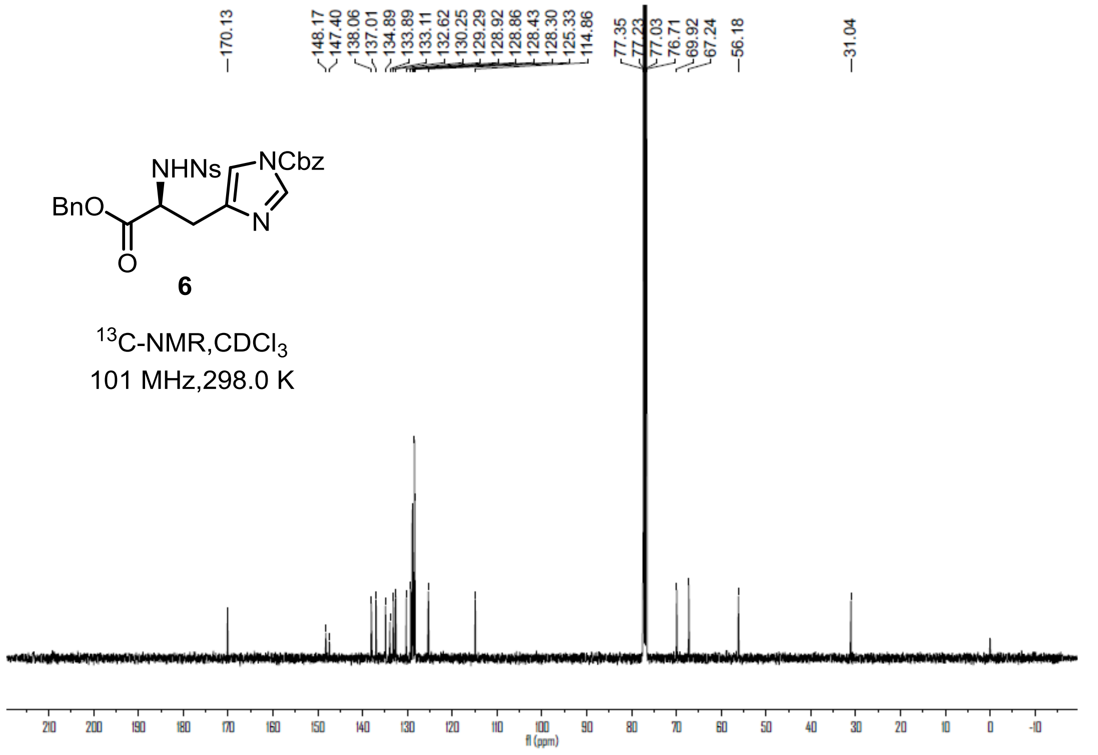

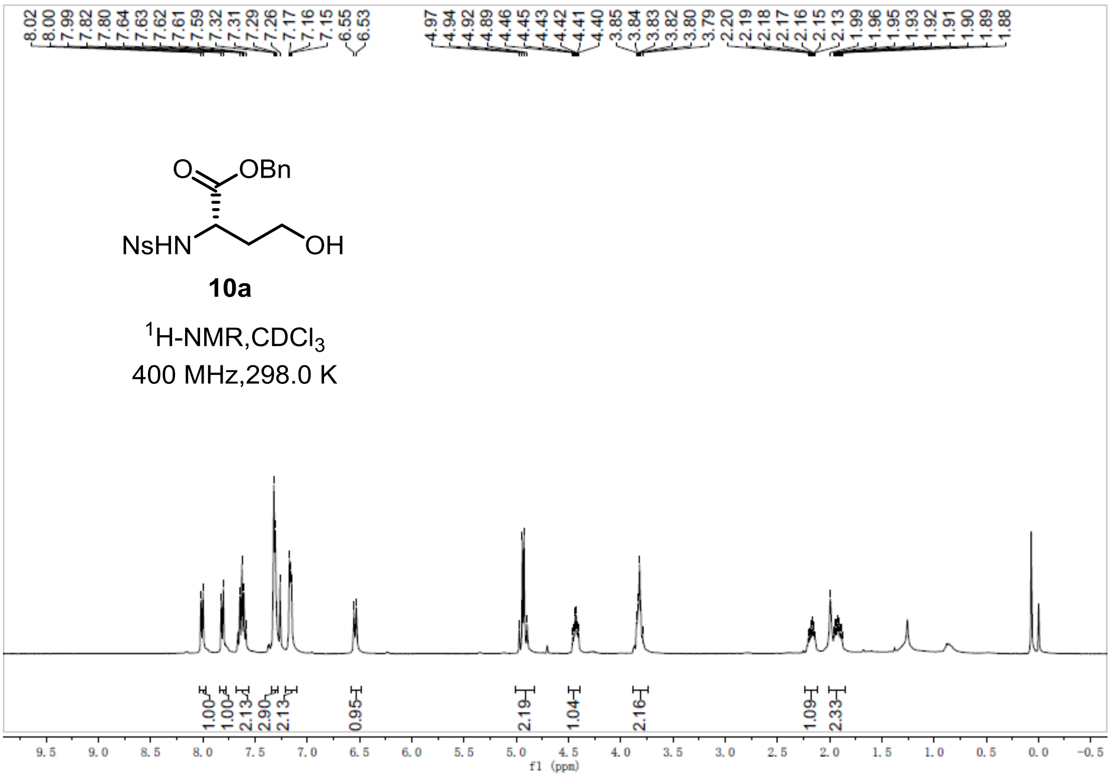

8.02
8.00
7.99
7.82
7.80
7.64
7.63
7.62
7.61
7.59
7.32
7.31
7.29
7.26
7.17
7.16
7.15
6.55
6.53
4.97
4.94
4.92
4.89
4.46
4.45
4.43
4.42
4.41
4.40
3.85
3.84
3.83
3.82
3.80
3.79
2.20
2.19
2.18
2.17
2.16
2.15
2.13
1.99
1.96
1.95
1.93
1.92
1.91
1.90
1.89
1.88
O
OBn
NsHN
OH
10a
1H-NMR,CDCl3
400 MHz,298.0 K
1.00
1.00
2.13
2.90
2.13
0.95
2.19
1.04
2.16
1.09
2.33
f1 (ppm)

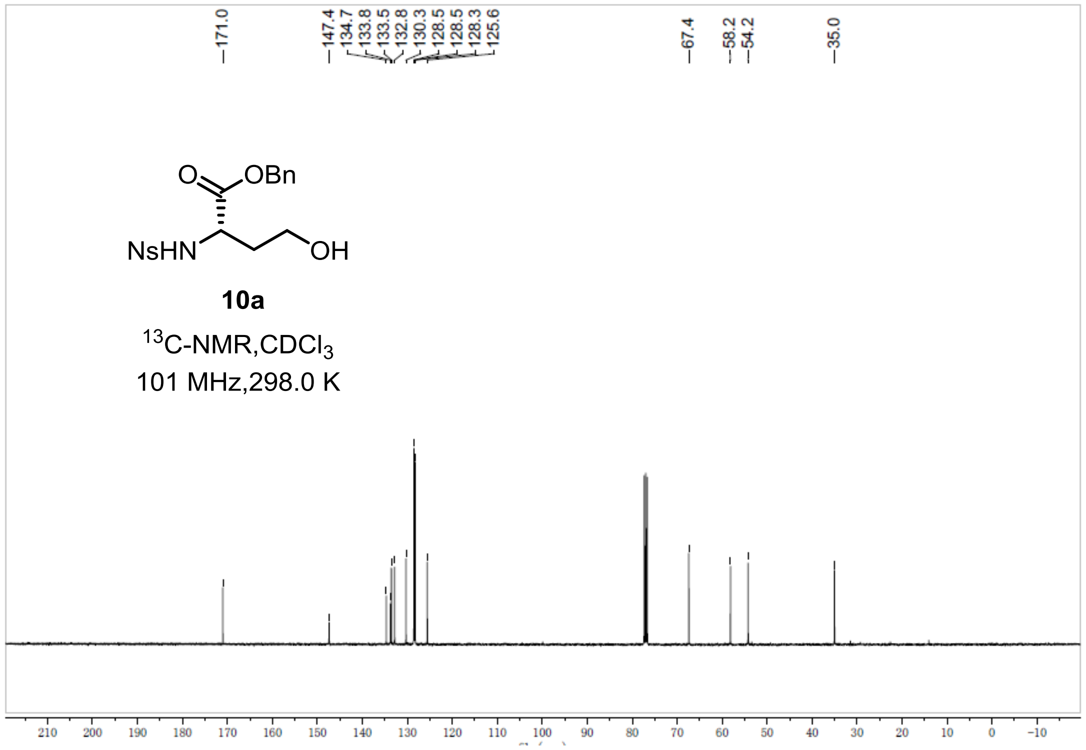

171.0
147.4
134.7
133.8
133.5
132.8
130.3
128.5
128.5
128.3
125.6
67.4
58.2
54.2
35.0
O
OBn
NsHN
OH
10a
13C-NMR,CDCl3
101 MHz,298.0 K

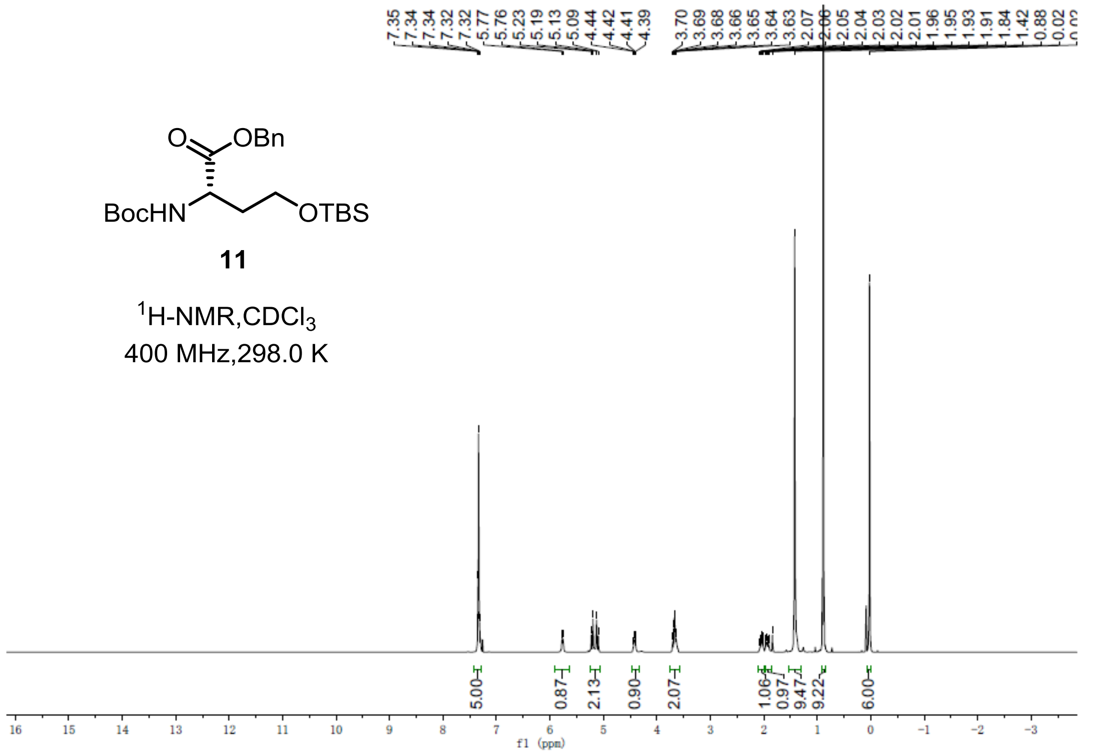

11

^{1}H-NMR, $CDCl_3$
400 MHz, 298.0 K

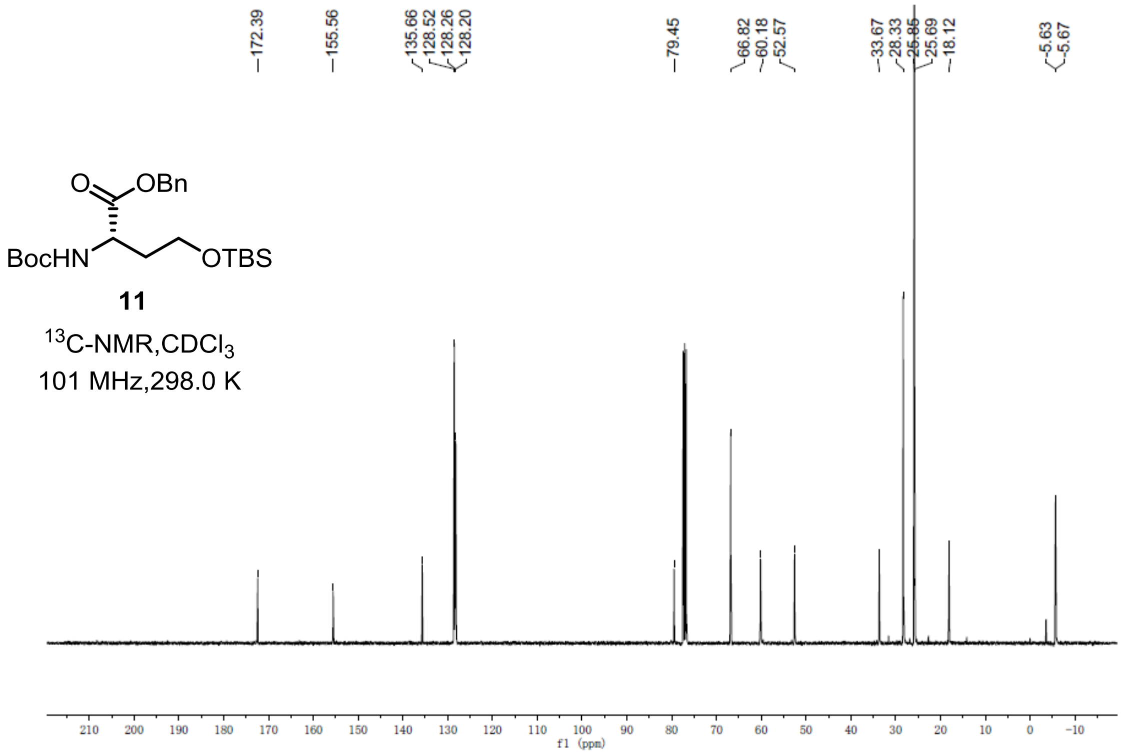

11

^{13}C-NMR, $CDCl_3$
101 MHz, 298.0 K

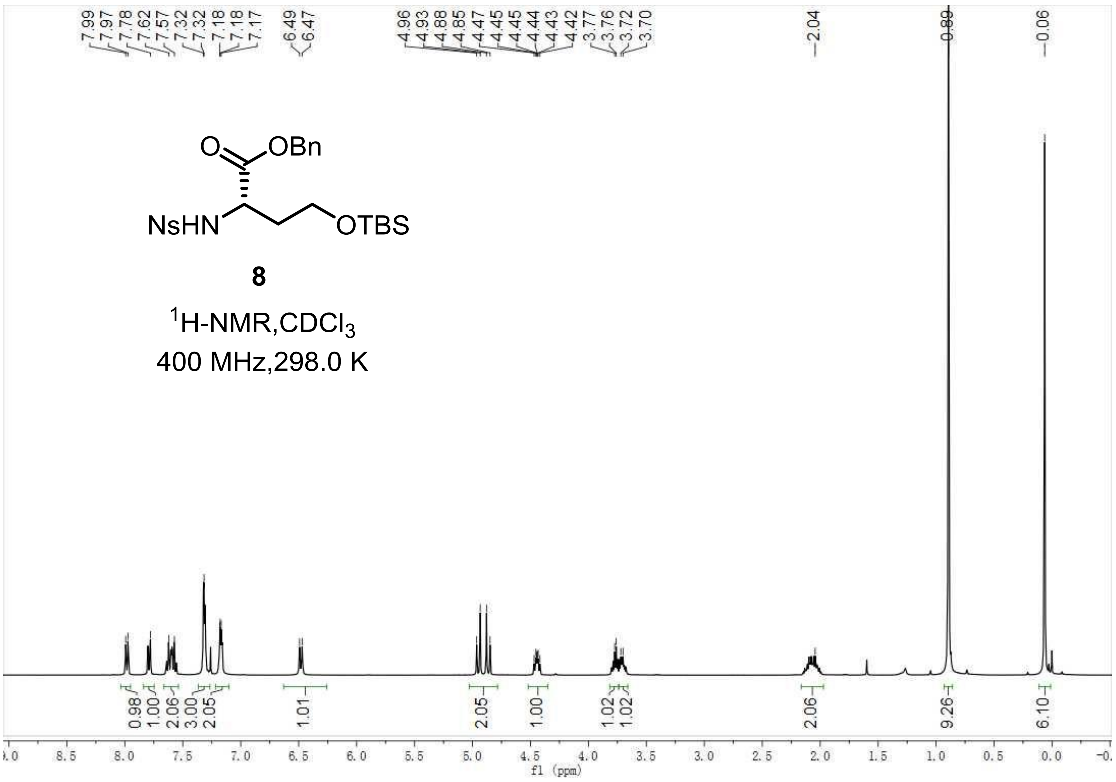
7.99
7.97
7.78
7.62
7.57
7.32
7.32
7.18
7.18
7.17
6.49
6.47
4.96
4.93
4.88
4.85
4.47
4.45
4.45
4.44
4.43
4.42
3.77
3.76
3.72
3.70
2.04
0.89
0.06
OBn
O
NsHN
OTBS
8
1H-NMR,CDCl3
400 MHz,298.0 K
0.98
1.00
2.06
3.00
2.05
1.01
2.05
1.00
1.02
1.02
2.06
9.26
6.10
f1 (ppm)

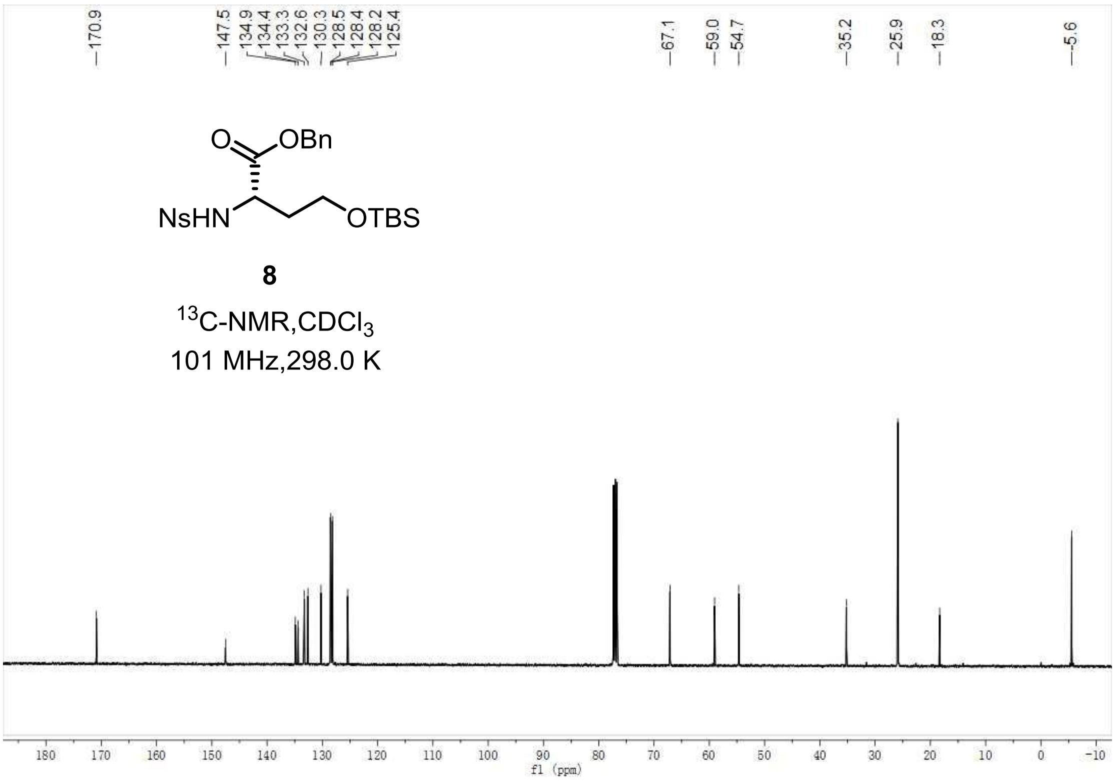
170.9
147.5
134.9
134.4
133.3
132.6
130.3
128.5
128.4
128.2
125.4
67.1
59.0
54.7
35.2
25.9
18.3
-5.6
OBn
O
NsHN
OTBS
8
13C-NMR,CDCl3
101 MHz,298.0 K
f1 (ppm)

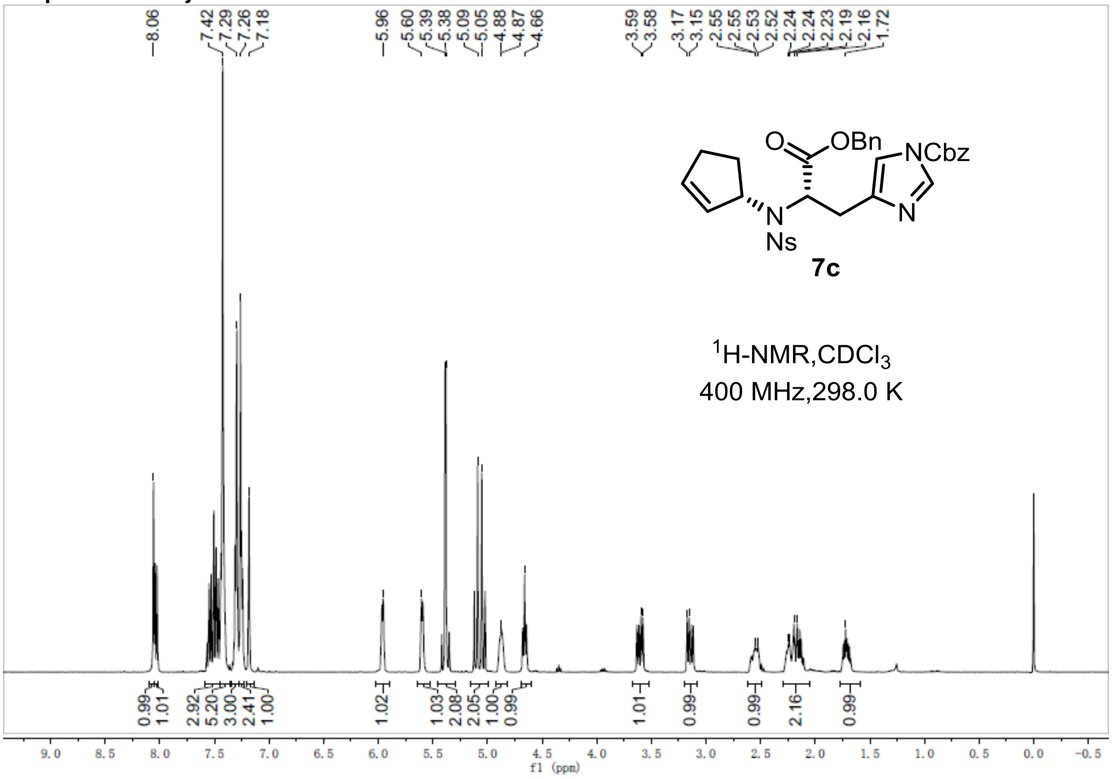
8.06
7.42
7.29
7.26
7.18
5.96
5.60
5.39
5.38
5.09
5.05
4.88
4.87
4.66
3.59
3.58
3.17
3.15
2.55
2.55
2.53
2.52
2.24
2.24
2.23
2.19
2.16
1.72
O
OBn
NCbz
N
N
Ns
7c
^{1}H-NMR,CDCl$_3$
400 MHz,298.0 K
0.99
1.01
2.92
5.20
3.00
2.41
1.00
1.02
1.03
2.08
2.05
1.00
0.99
1.01
0.99
0.99
2.16
0.99
f1 (ppm)

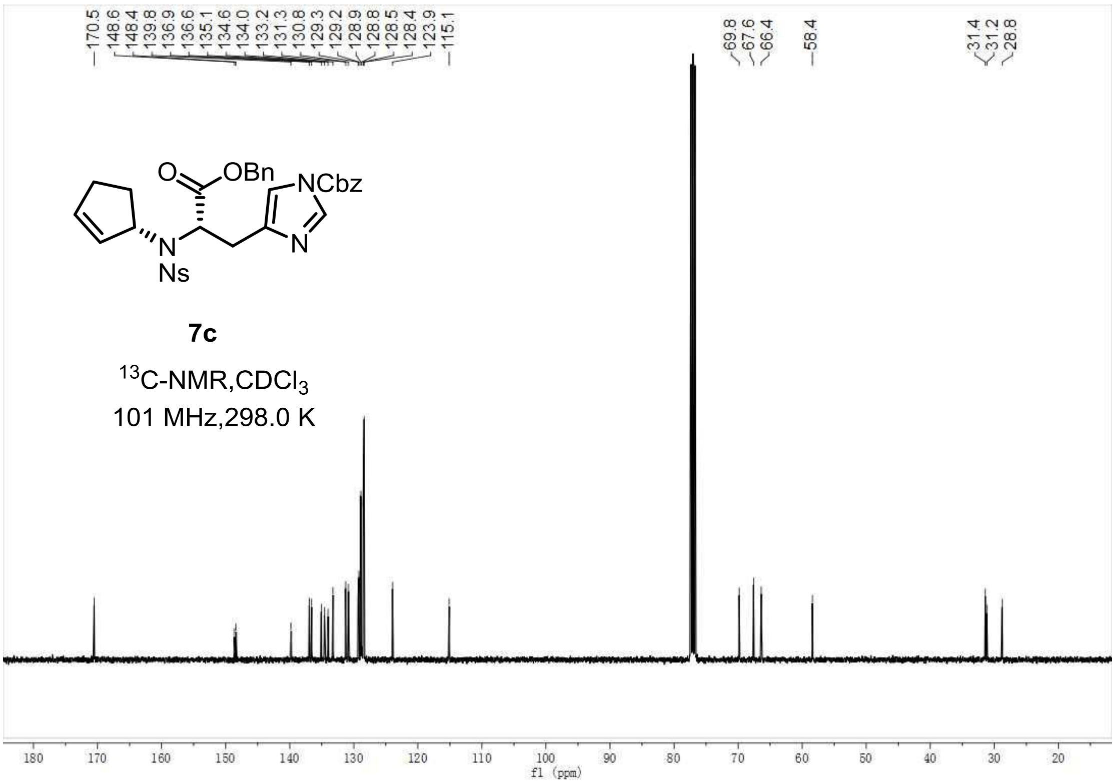
170.5
148.6
148.4
139.8
136.9
136.6
135.1
134.6
134.0
133.2
131.3
130.8
129.3
129.2
128.9
128.8
128.5
128.4
123.9
115.1
69.8
67.6
66.4
58.4
31.4
31.2
28.8
O
OBn
NCbz
N
N
Ns
7c
^{13}C-NMR,CDCl$_3$
101 MHz,298.0 K
f1 (ppm)

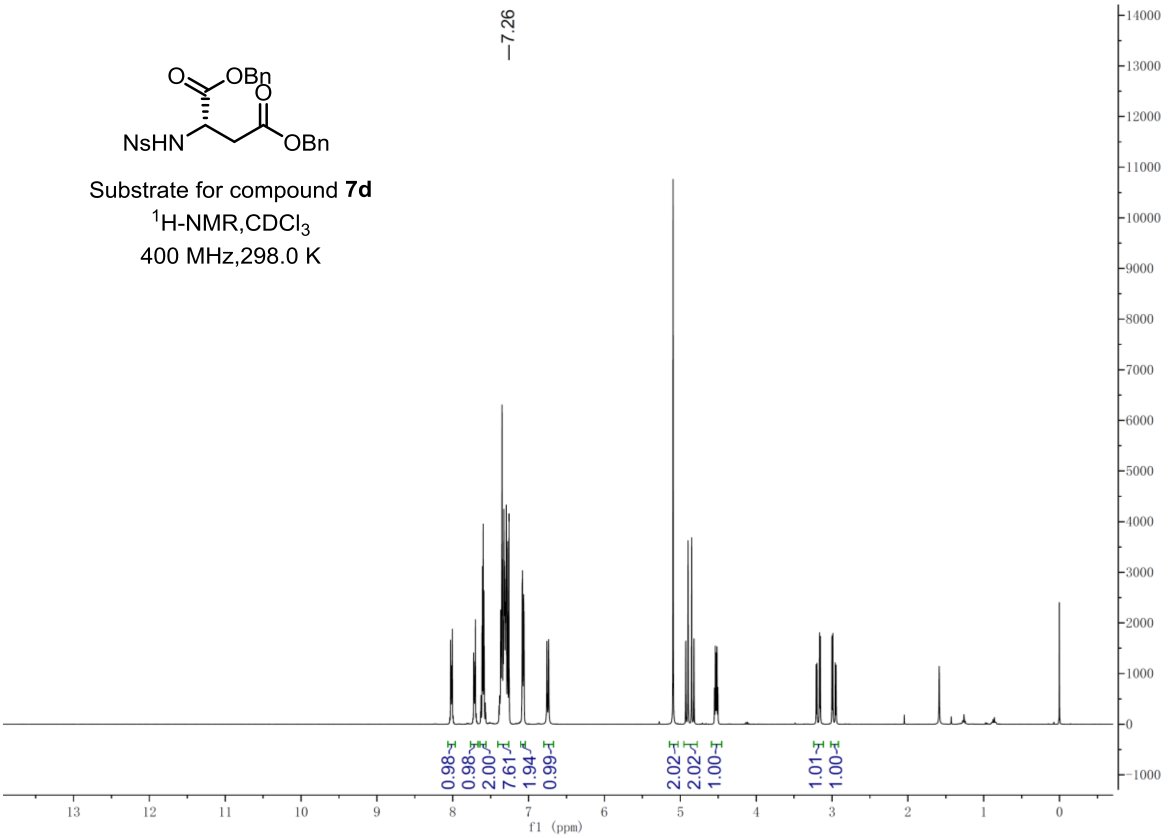
OBn
NsHN
OBn
Substrate for compound **7d**
^{1}H-NMR,CDCl$_3$
400 MHz,298.0 K
7.26
0.98
0.98
2.00
7.61
1.94
0.99
2.02
2.02
1.00
1.01
1.00
f1 (ppm)

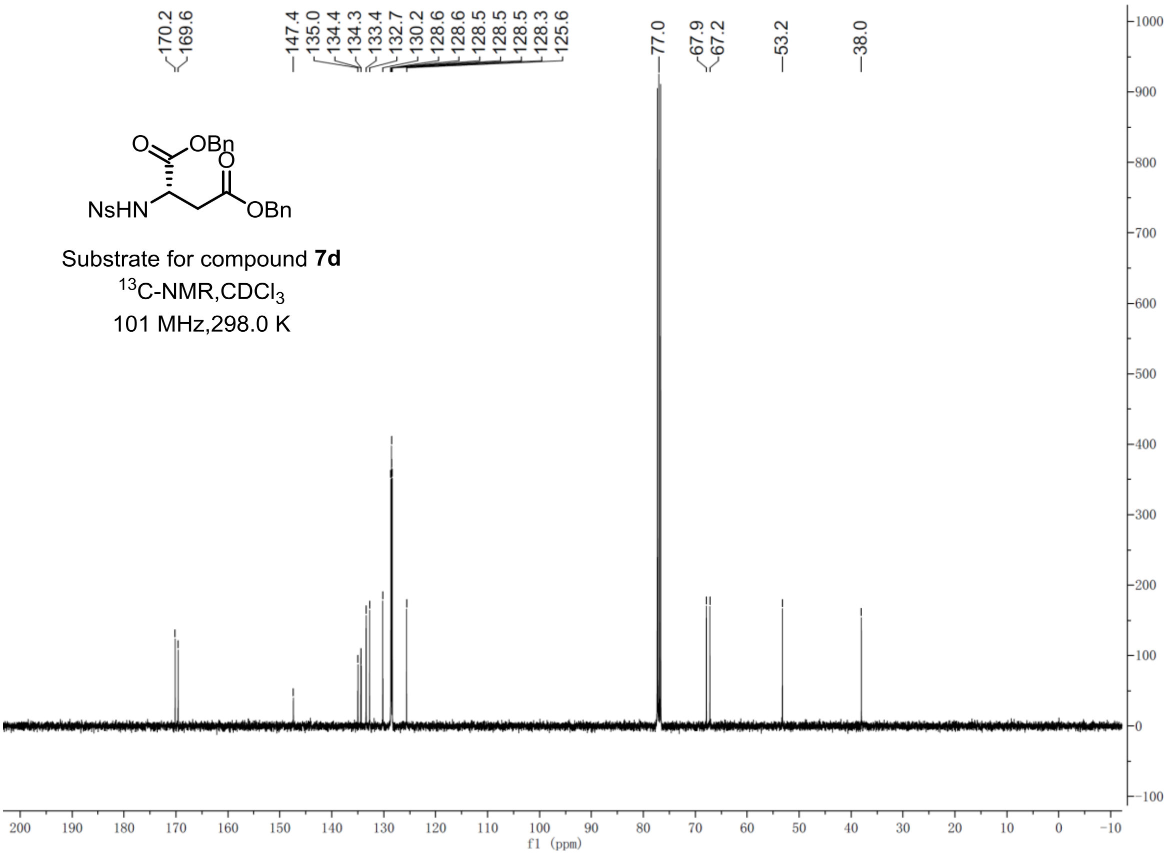
170.2
169.6
147.4
135.0
134.4
134.3
133.4
132.7
130.2
128.6
128.6
128.5
128.5
128.5
128.3
125.6
77.0
67.9
67.2
53.2
38.0
OBn
NsHN
OBn
Substrate for compound **7d**
^{13}C-NMR,CDCl$_3$
101 MHz,298.0 K
f1 (ppm)

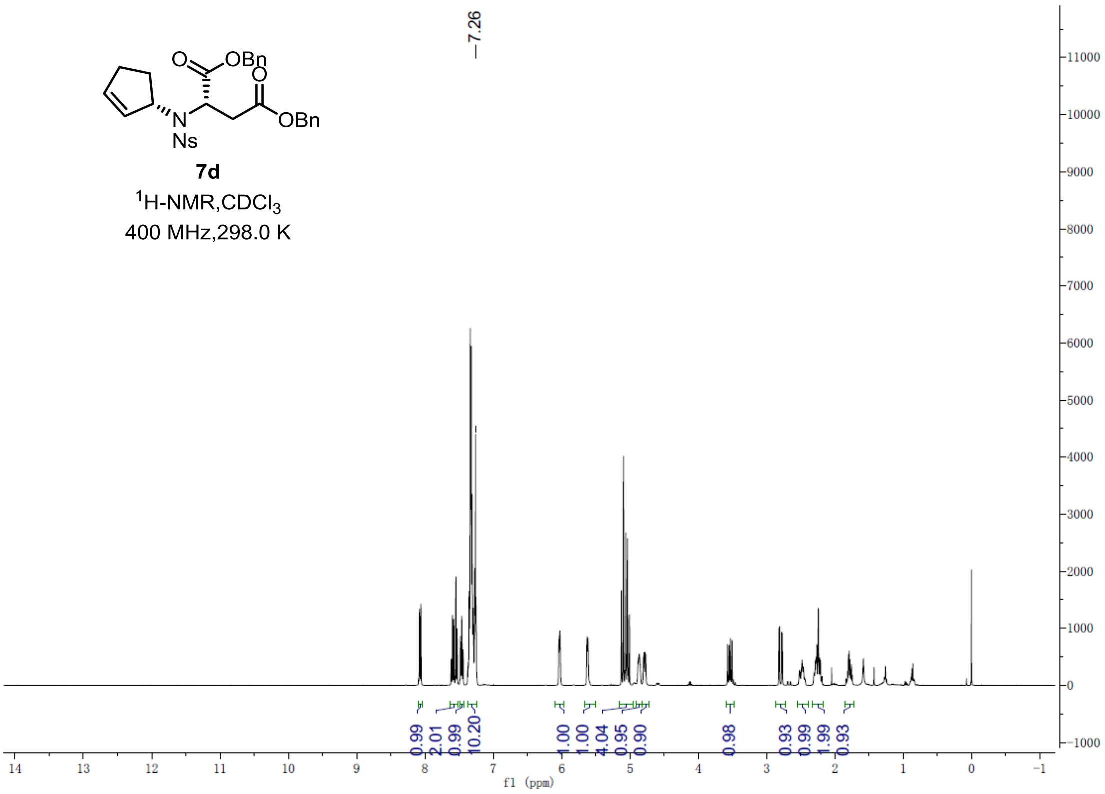
7d
^{1}H-NMR,CDCl$_3$
400 MHz,298.0 K
7.26
0.99 2.01 0.99 10.20 1.00 1.00 4.04 0.95 0.90 0.98 0.93 0.99 1.99 0.93
f1 (ppm)

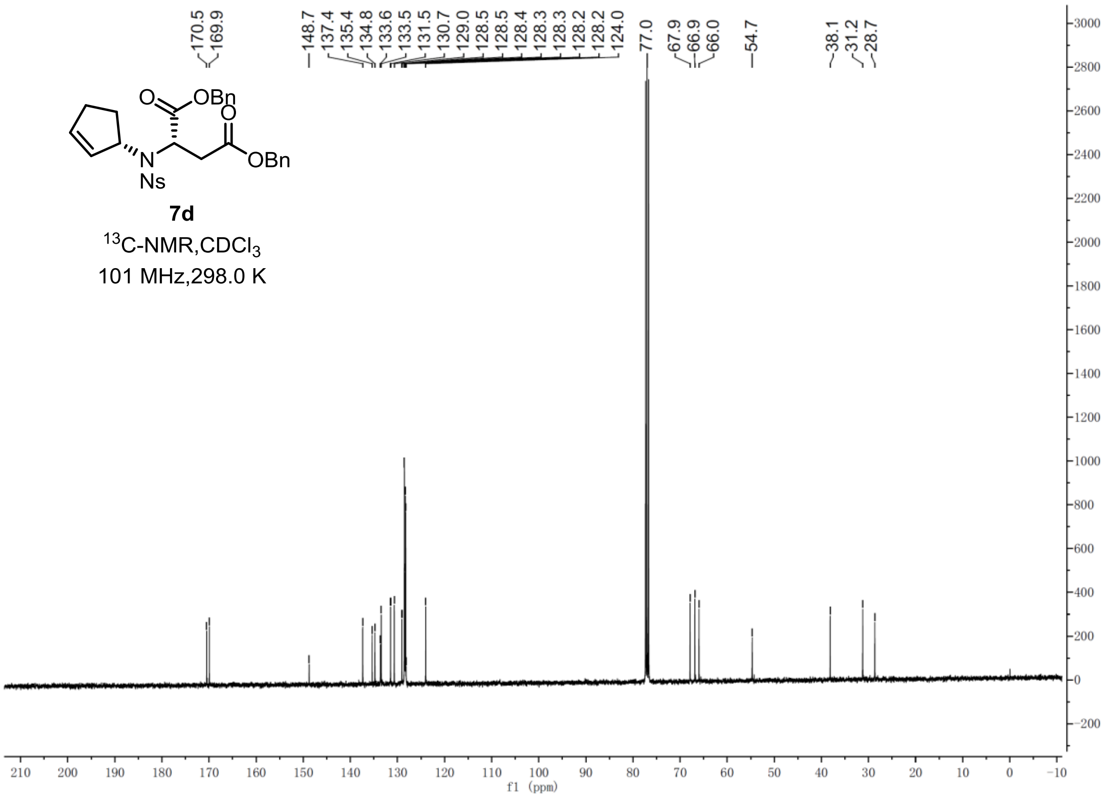
7d
^{13}C-NMR,CDCl$_3$
101 MHz,298.0 K
170.5 169.9 148.7 137.4 135.4 134.8 133.6 133.5 131.5 130.7 129.0 128.5 128.5 128.4 128.3 128.3 128.2 128.2 124.0 77.0 67.9 66.9 66.0 54.7 38.1 31.2 28.7
f1 (ppm)

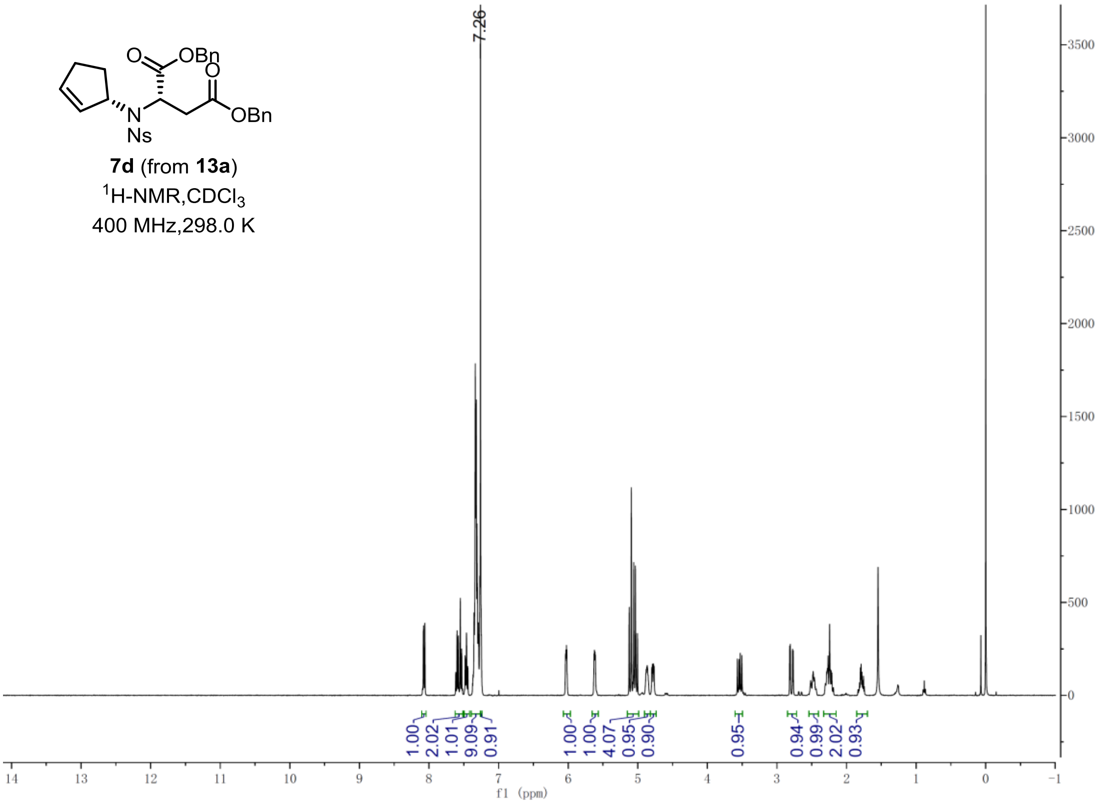

7d (from **13a**)
^{1}H-NMR,CDCl$_3$
400 MHz,298.0 K

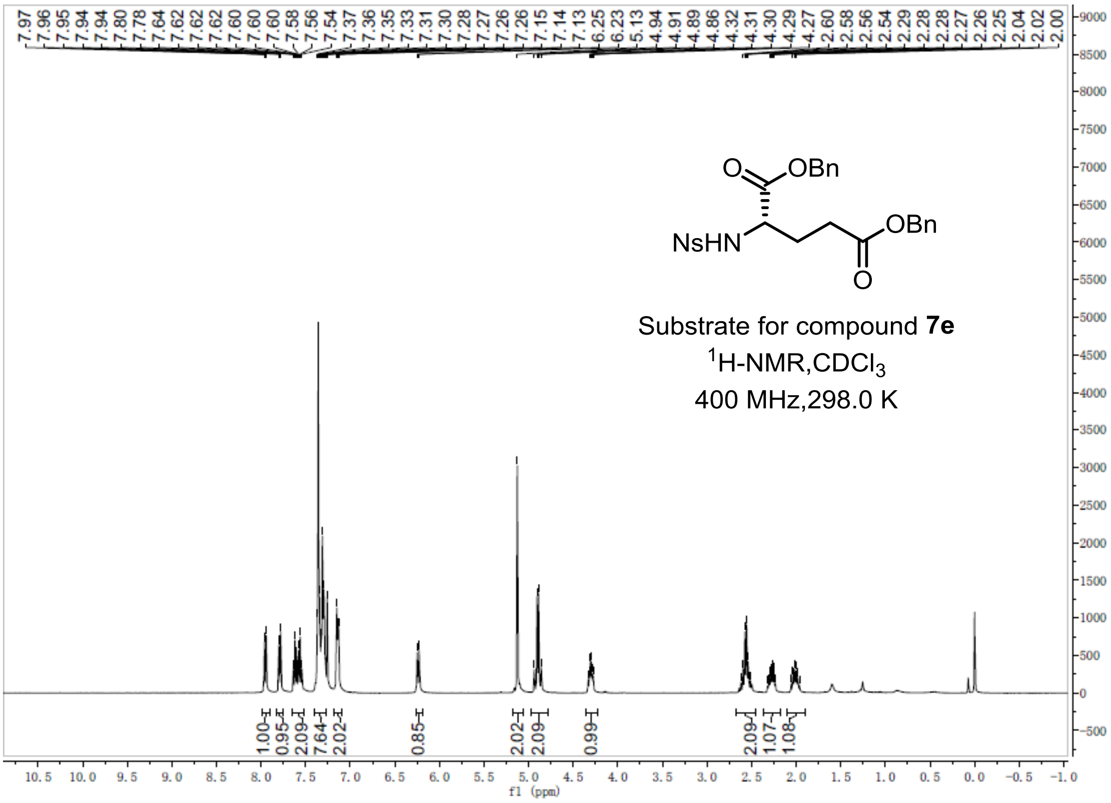

Substrate for compound **7e**
^{1}H-NMR,$CDCl_3$
400 MHz,298.0 K

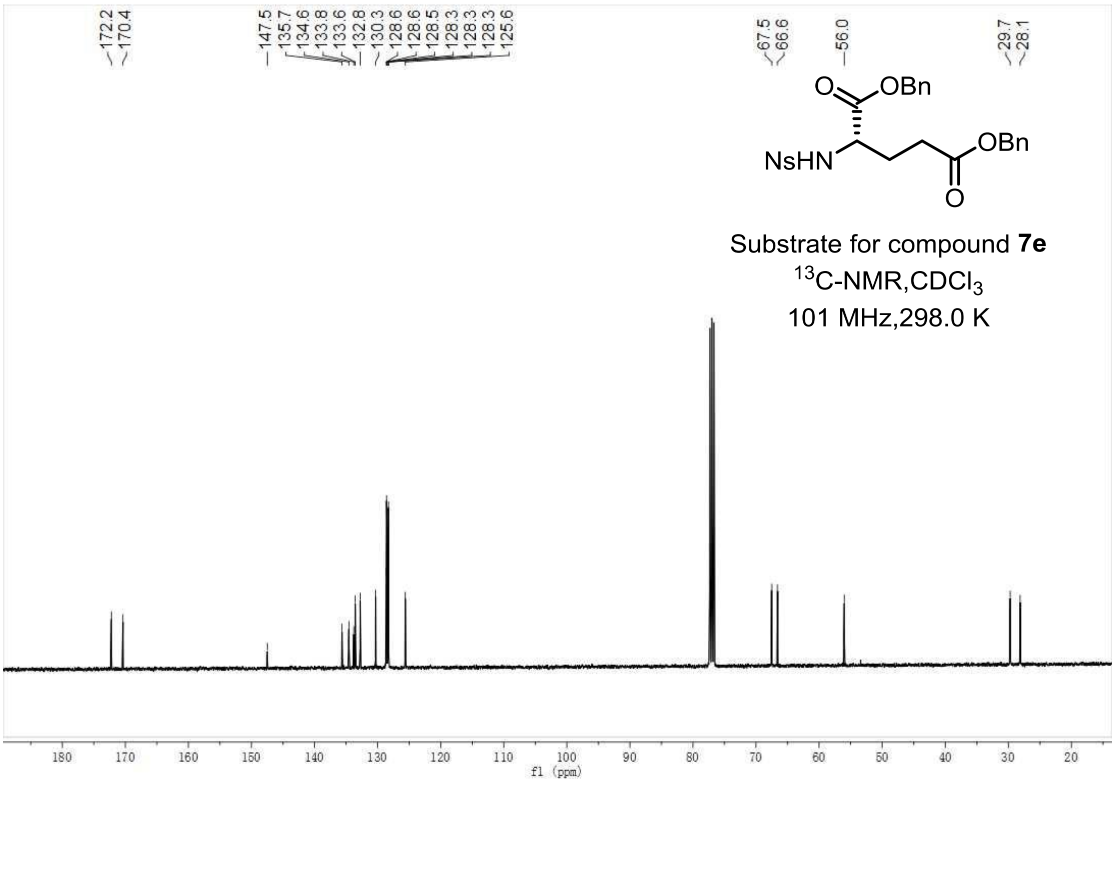

Substrate for compound **7e**
^{13}C-NMR,$CDCl_3$
101 MHz,298.0 K

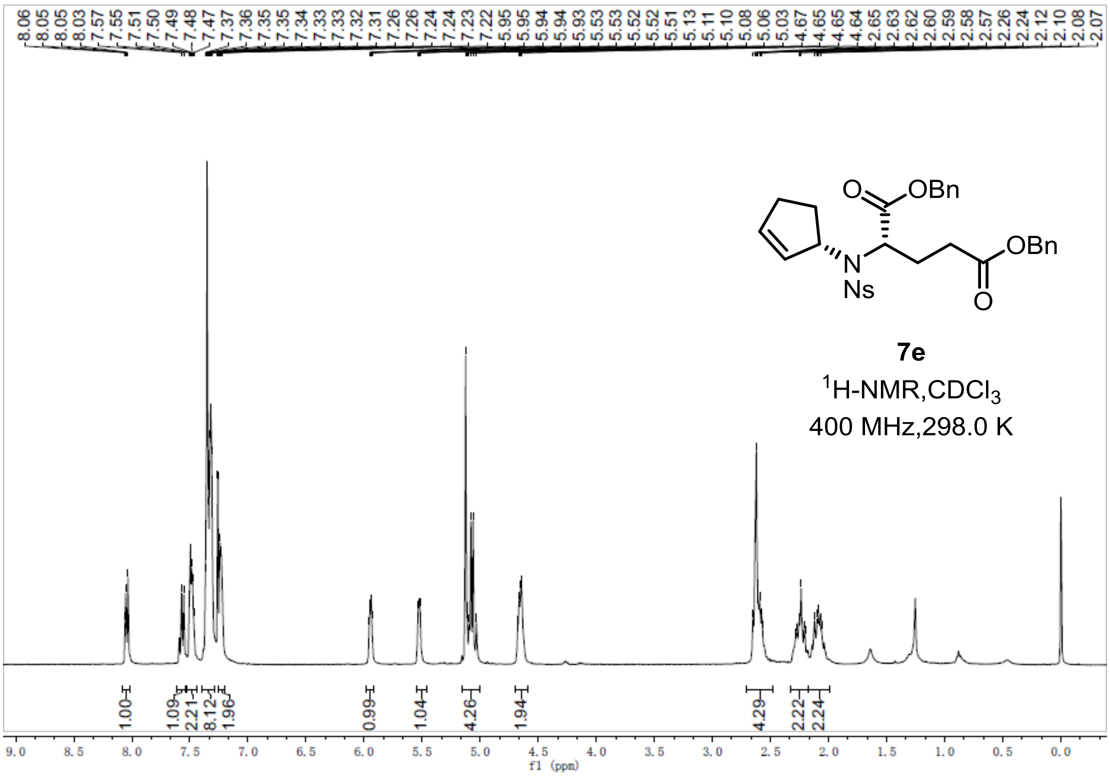
OBn
OBn
N
Ns
O
O
7e
1H-NMR,CDCl3
400 MHz,298.0 K
f1 (ppm)

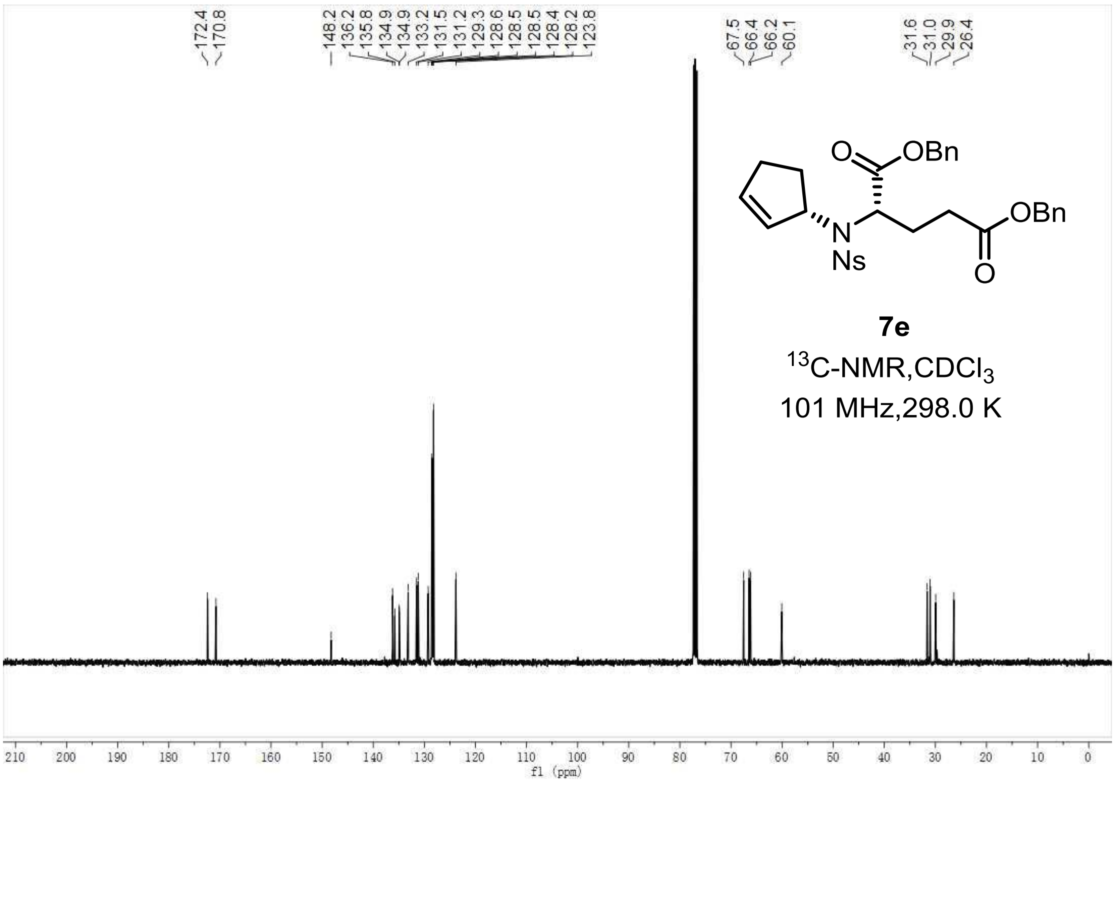
OBn
OBn
N
Ns
O
O
7e
13C-NMR,CDCl3
101 MHz,298.0 K
f1 (ppm)

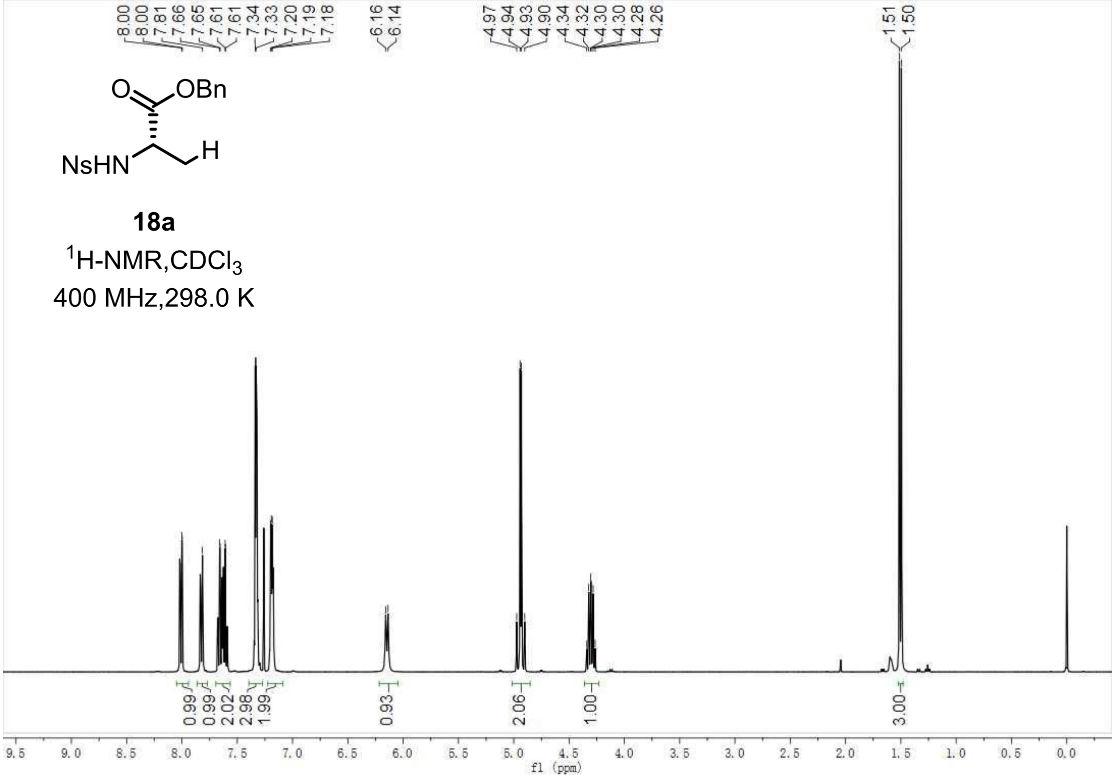
8.00
8.00
7.81
7.66
7.65
7.61
7.61
7.34
7.33
7.20
7.19
7.18
6.16
6.14
4.97
4.94
4.93
4.90
4.34
4.32
4.30
4.30
4.28
4.26
1.51
1.50
O
OBn
NsHN
H
18a
1H-NMR,CDCl3
400 MHz,298.0 K
0.99
0.99
2.02
2.98
1.99
0.93
2.06
1.00
3.00
f1 (ppm)

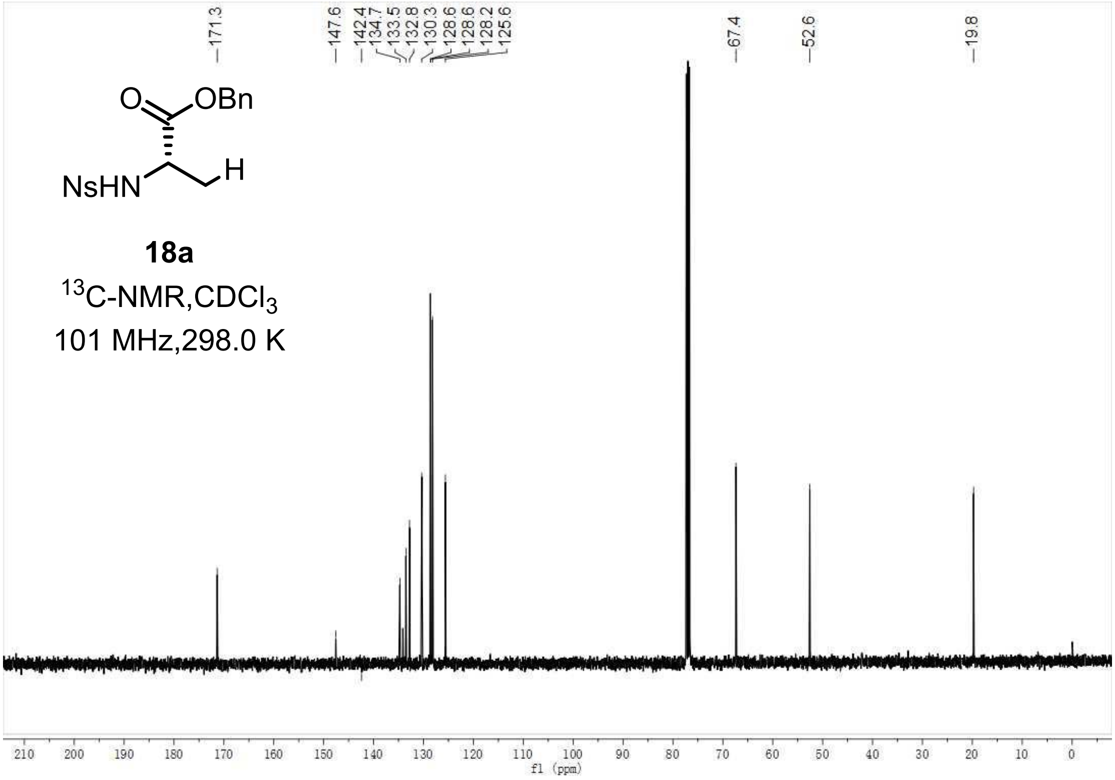
171.3
147.6
142.4
134.7
133.5
132.8
130.3
128.6
128.6
128.2
125.6
67.4
52.6
19.8
O
OBn
NsHN
H
18a
13C-NMR,CDCl3
101 MHz,298.0 K
f1 (ppm)

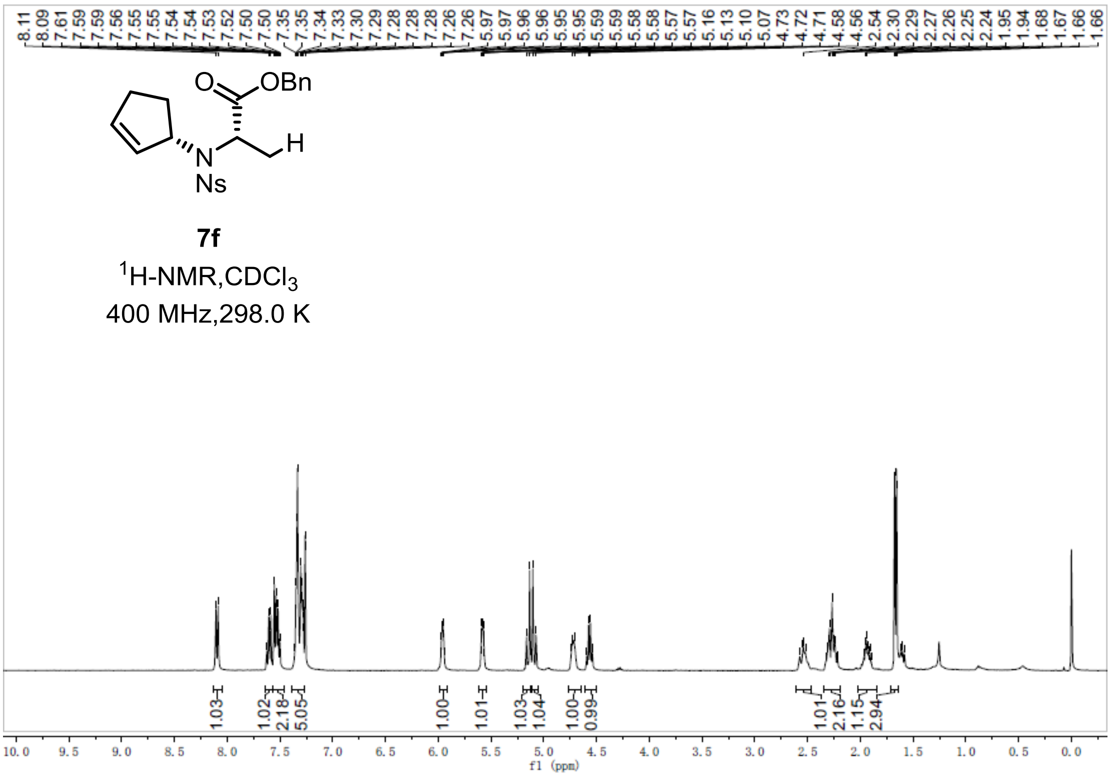

8.11
8.09
7.61
7.59
7.59
7.56
7.55
7.55
7.54
7.54
7.53
7.52
7.50
7.50
7.35
7.35
7.34
7.33
7.30
7.29
7.28
7.28
7.28
7.26
7.26
5.97
5.97
5.96
5.96
5.95
5.95
5.59
5.59
5.58
5.58
5.57
5.57
5.16
5.13
5.10
5.07
4.73
4.72
4.71
4.58
4.56
2.54
2.30
2.29
2.27
2.26
2.25
2.24
1.95
1.94
1.68
1.67
1.66
1.66
O
OBn
H
N
Ns
7f
1H-NMR,CDCl3
400 MHz,298.0 K
1.03
1.02
2.18
5.05
1.00
1.01
1.03
1.04
1.00
0.99
1.01
2.16
1.15
2.94
f1 (ppm)

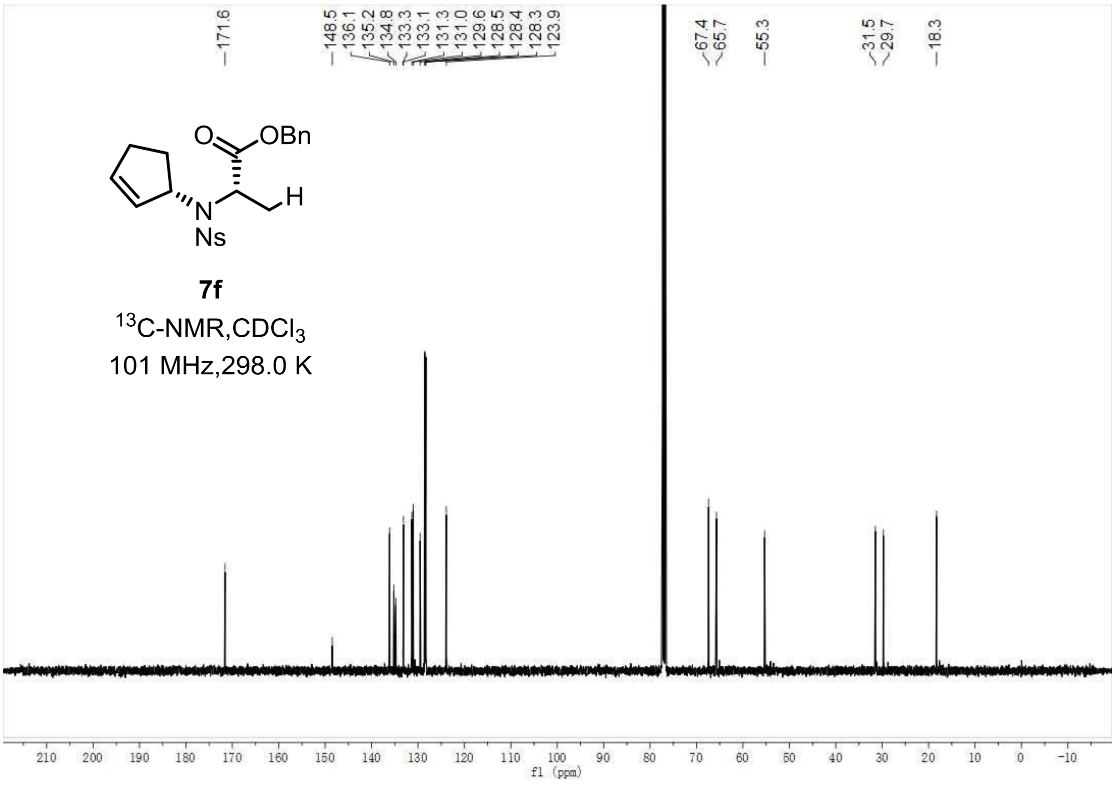

171.6
148.5
136.1
135.2
134.8
133.3
133.1
131.3
131.0
129.6
128.5
128.4
128.3
123.9
67.4
65.7
55.3
31.5
29.7
18.3
O
OBn
H
N
Ns
7f
13C-NMR,CDCl3
101 MHz,298.0 K
f1 (ppm)

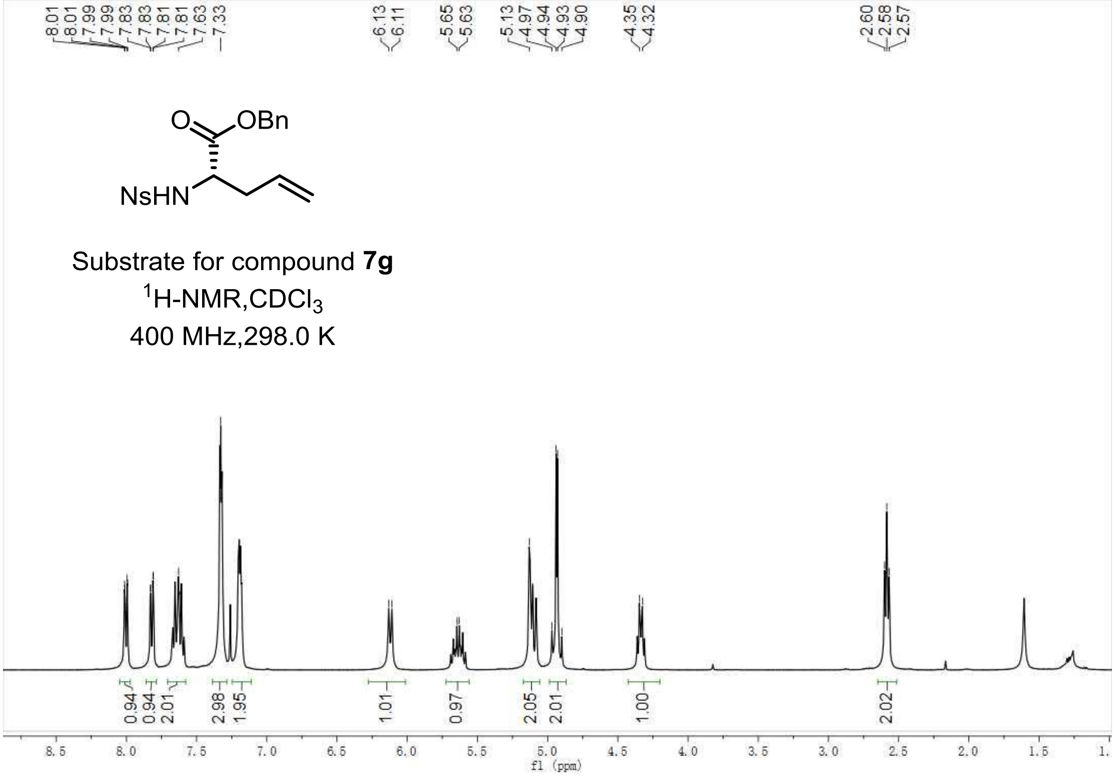
8.01 8.01 7.99 7.99 7.83 7.83 7.81 7.81 7.63 7.33
6.13 6.11
5.65 5.63
5.13 4.97 4.94 4.93 4.90
4.35 4.32
2.60 2.58 2.57
O
OBn
NsHN
Substrate for compound **7g**
^{1}H-NMR,$CDCl_3$
400 MHz,298.0 K
0.94 0.94 2.01 2.98 1.95 1.01 0.97 2.05 2.01 1.00 2.02
f1 (ppm)

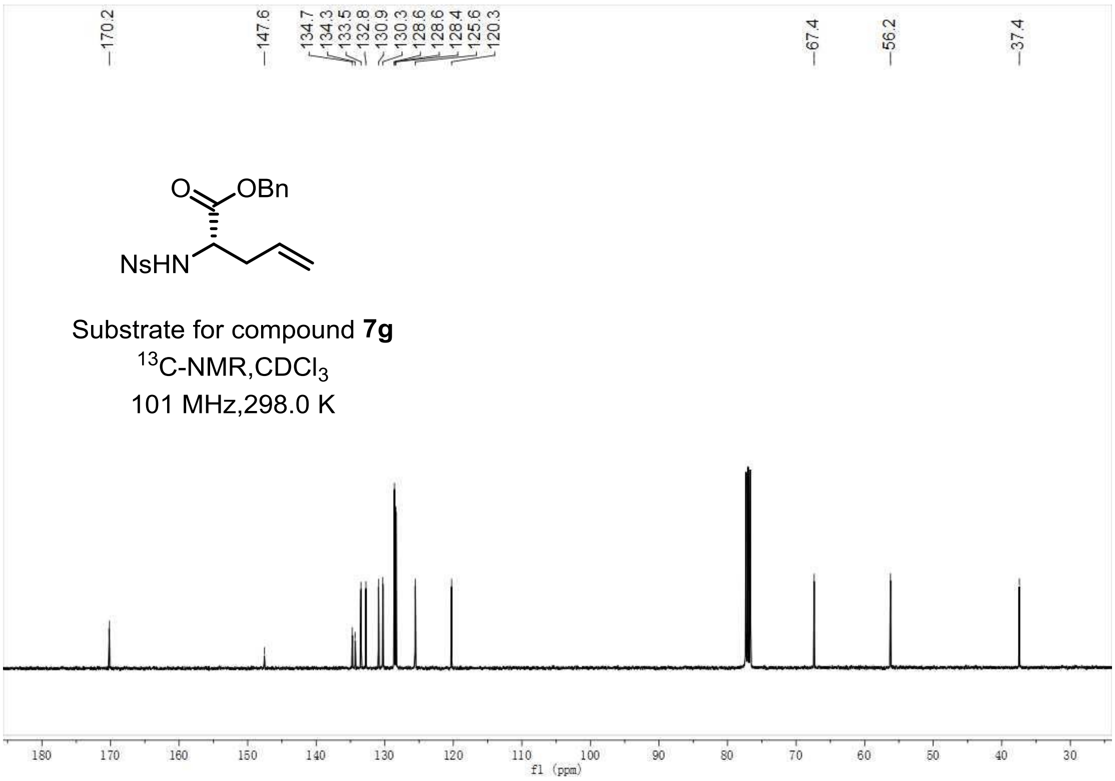
170.2
147.6
134.7 134.3 133.5 132.8 130.9 130.3 128.6 128.6 128.4 125.6 120.3
67.4
56.2
37.4
O
OBn
NsHN
Substrate for compound **7g**
^{13}C-NMR,$CDCl_3$
101 MHz,298.0 K
f1 (ppm)

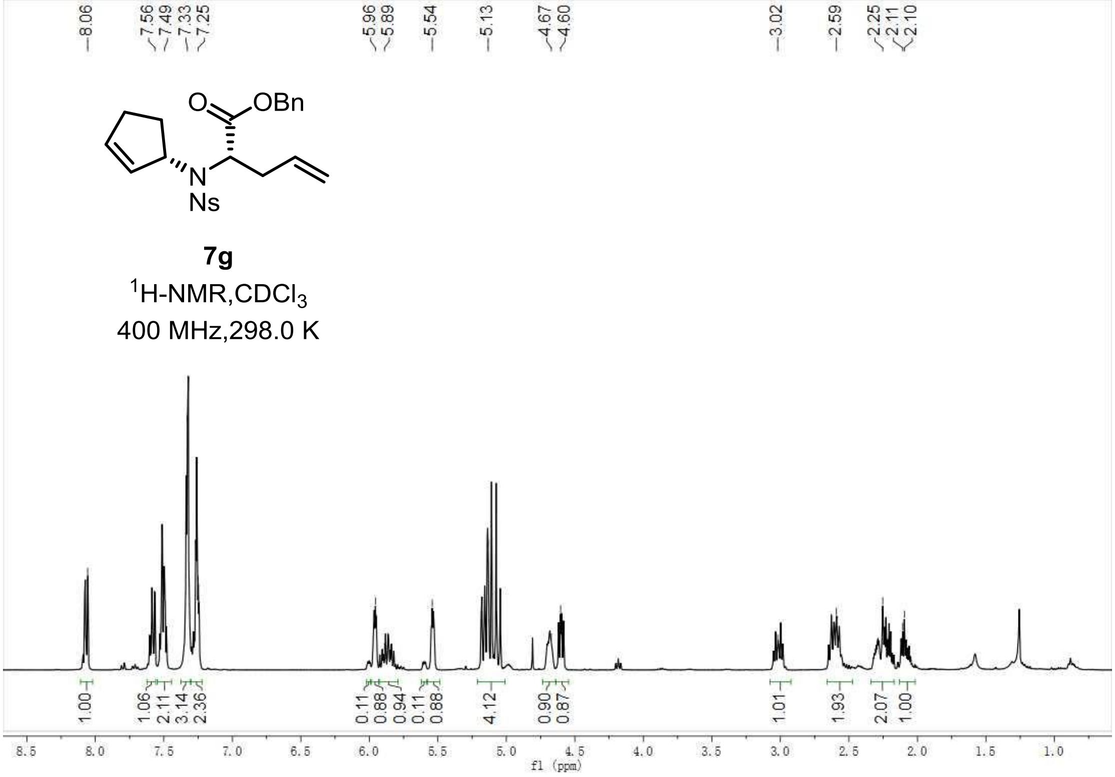
8.06
7.56
7.49
7.33
7.25
5.96
5.89
5.54
5.13
4.67
4.60
3.02
2.59
2.25
2.11
2.10
OBn
O
N
Ns
7g
1H-NMR,CDCl3
400 MHz,298.0 K
1.00
1.06
2.11
3.14
2.36
0.11
0.88
0.94
0.11
0.88
4.12
0.90
0.87
1.01
1.93
2.07
1.00
f1 (ppm)

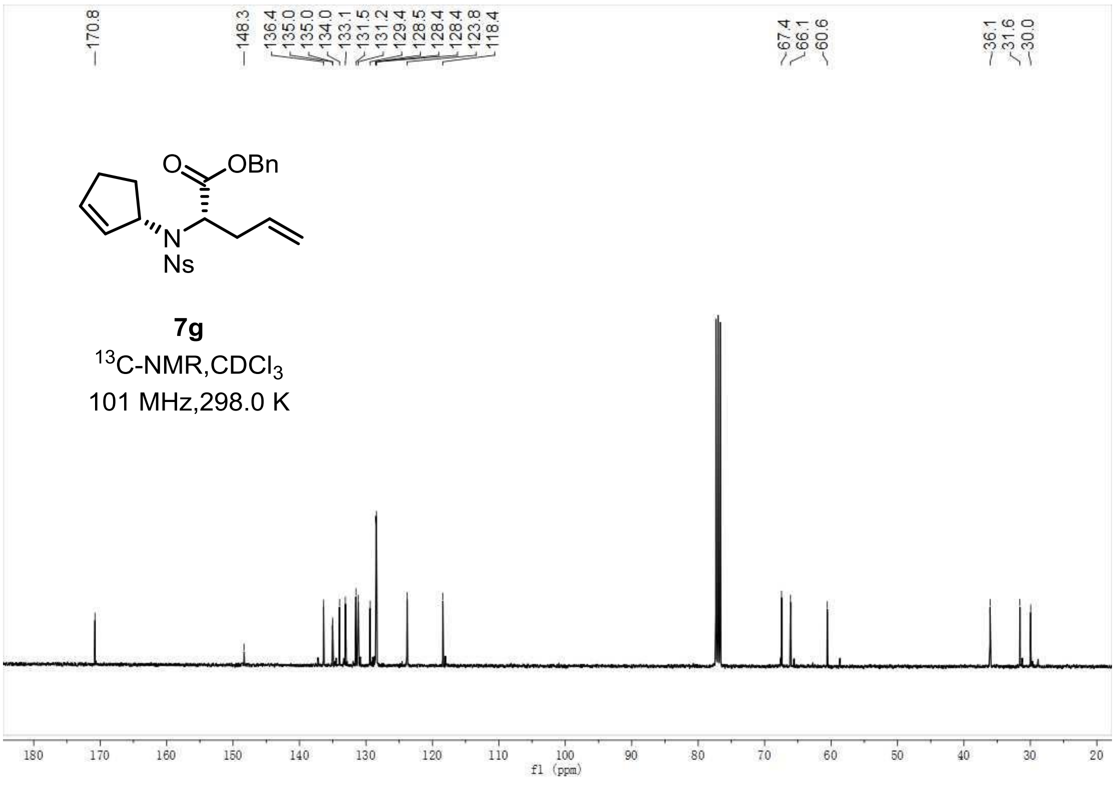
170.8
148.3
136.4
135.0
135.0
134.0
133.1
131.5
131.2
129.4
128.5
128.4
128.4
123.8
118.4
67.4
66.1
60.6
36.1
31.6
30.0
OBn
O
N
Ns
7g
13C-NMR,CDCl3
101 MHz,298.0 K
f1 (ppm)

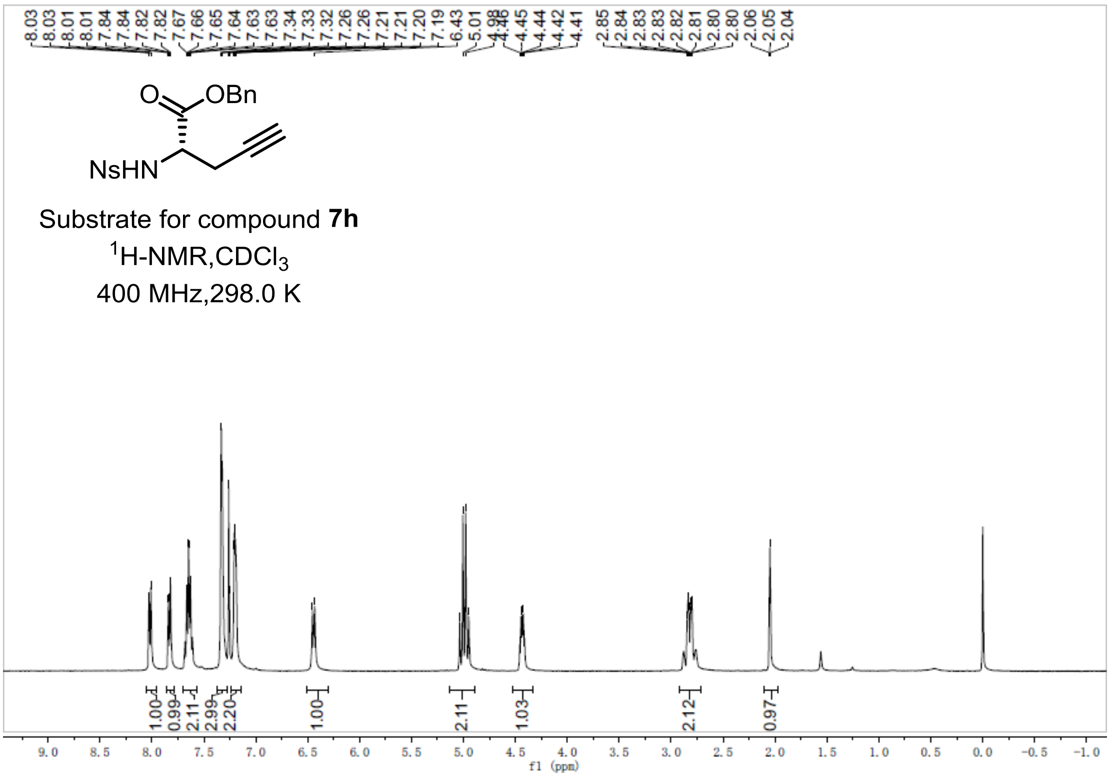
8.03 8.03 8.01 8.01 7.84 7.84 7.82 7.82 7.67 7.66 7.65 7.64 7.63 7.63 7.34 7.33 7.32 7.26 7.26 7.21 7.21 7.20 7.19 6.43 5.01 4.98 4.45 4.44 4.42 4.41 2.85 2.84 2.83 2.83 2.82 2.81 2.80 2.06 2.05 2.04
O OBn
NsHN
Substrate for compound **7h**
^{1}H-NMR,CDCl$_3$
400 MHz,298.0 K
1.00 0.99 2.11 2.99 2.20 1.00 2.11 1.03 2.12 0.97
9.0 8.5 8.0 7.5 7.0 6.5 6.0 5.5 5.0 4.5 4.0 3.5 3.0 2.5 2.0 1.5 1.0 0.5 0.0 -0.5 -1.0
f1 (ppm)

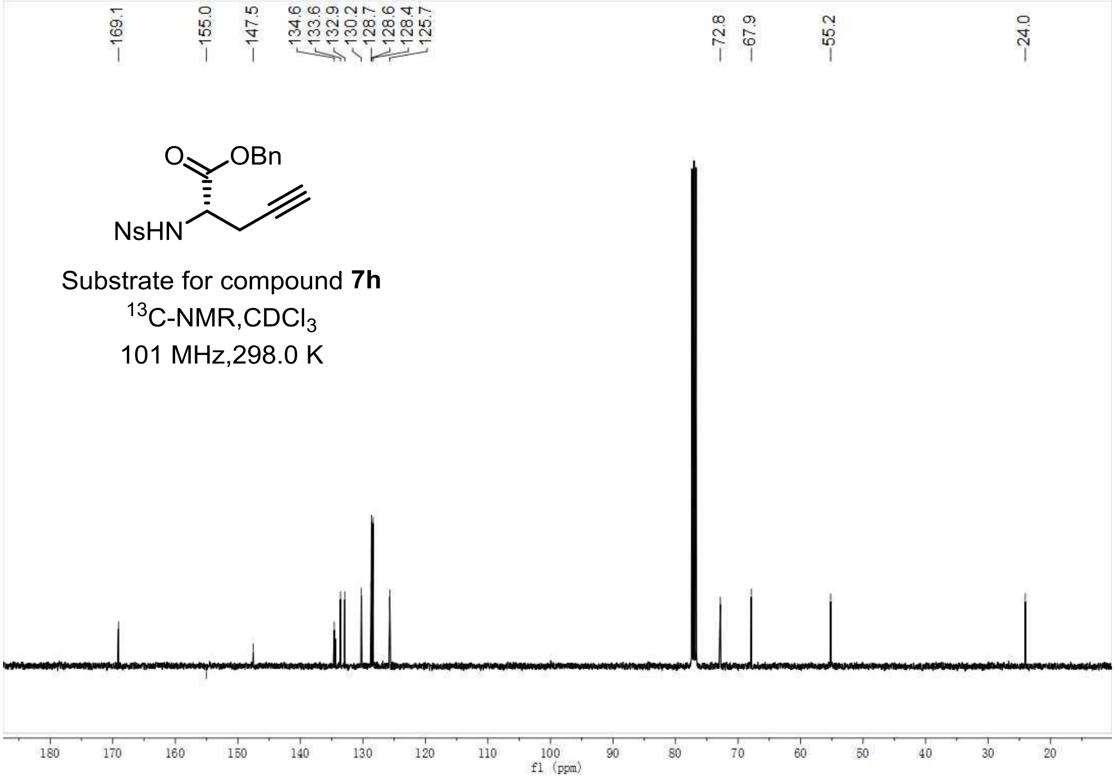
169.1 155.0 147.5 134.6 133.6 132.9 130.2 128.7 128.6 128.4 125.7 72.8 67.9 55.2 24.0
O OBn
NsHN
Substrate for compound **7h**
^{13}C-NMR,CDCl$_3$
101 MHz,298.0 K
180 170 160 150 140 130 120 110 100 90 80 70 60 50 40 30 20
f1 (ppm)

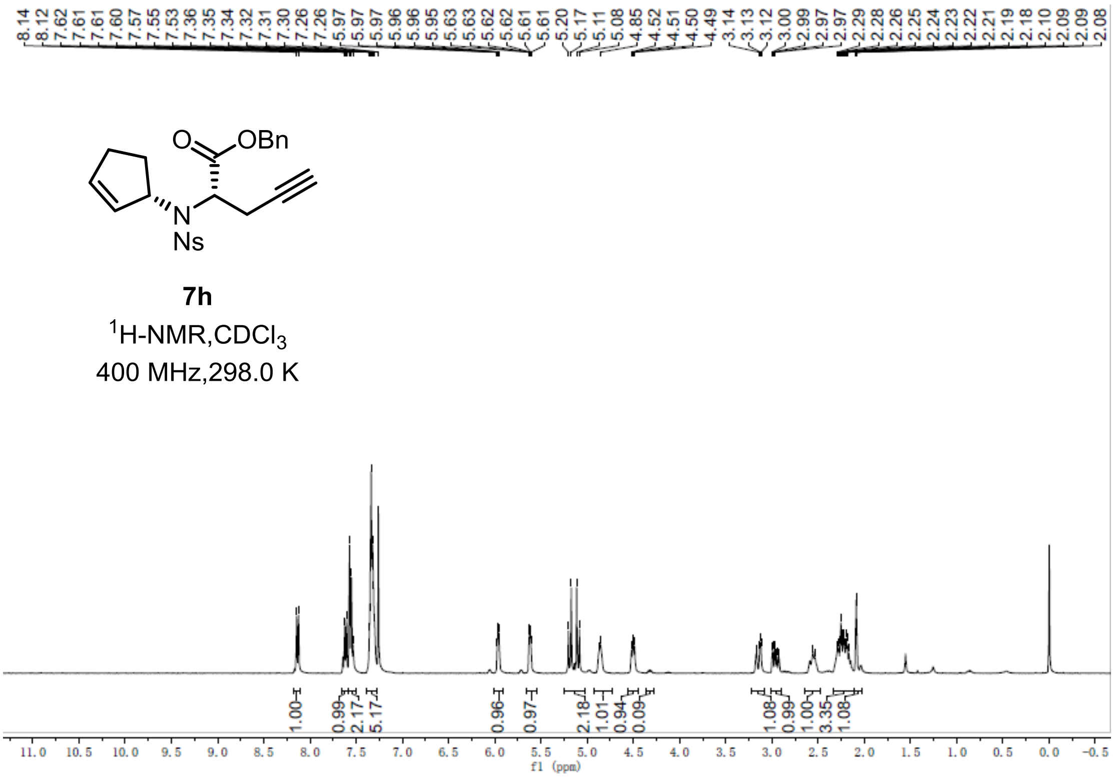
OBn
N
Ns
7h
1H-NMR,CDCl3
400 MHz,298.0 K
f1 (ppm)

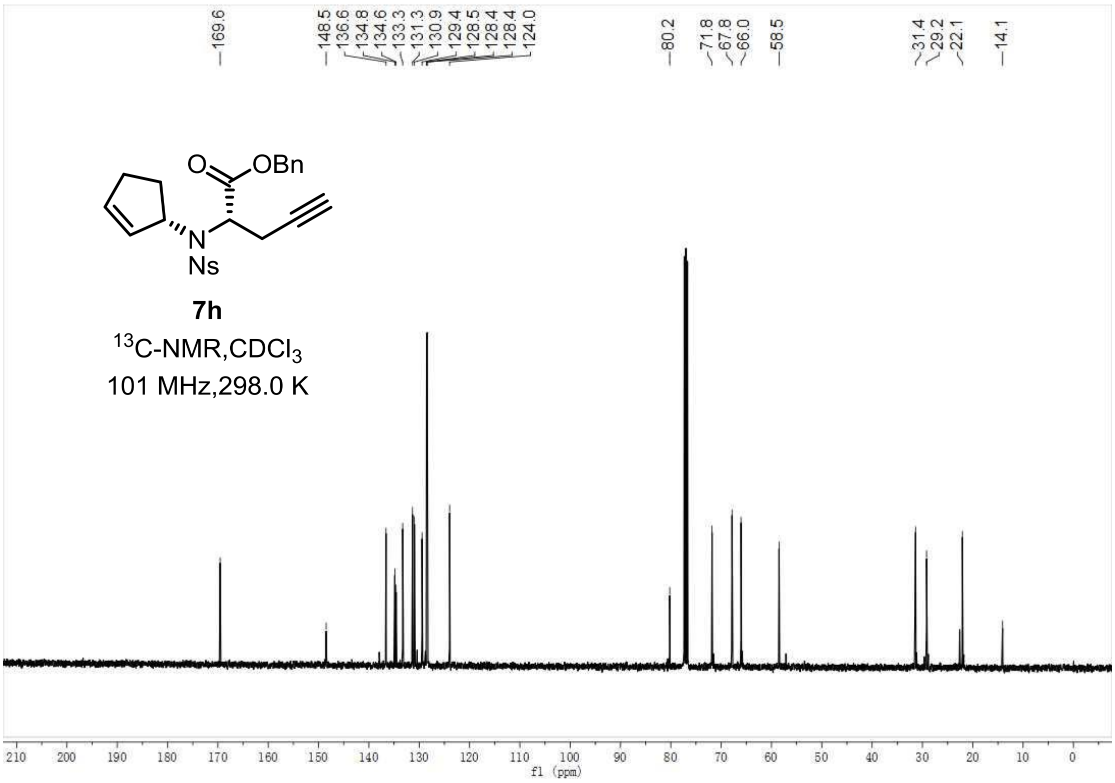
OBn
N
Ns
7h
13C-NMR,CDCl3
101 MHz,298.0 K
f1 (ppm)

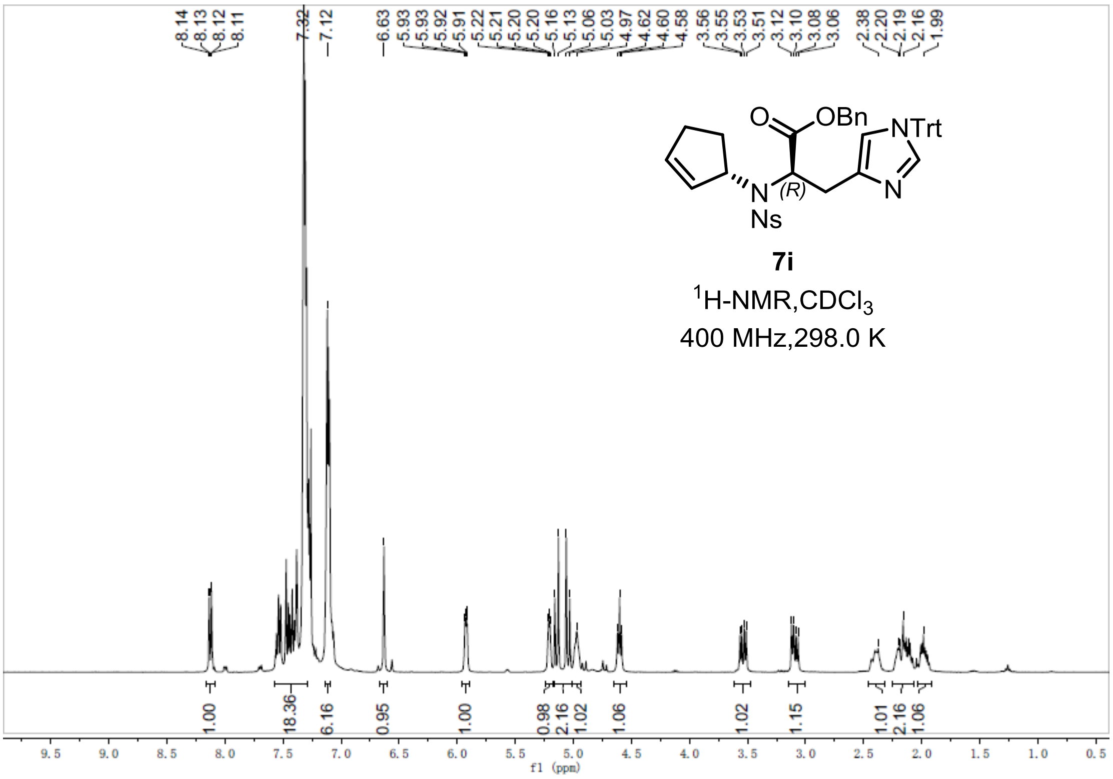
8.14 8.13 8.12 8.11 7.32 7.12 6.63 5.93 5.93 5.92 5.91 5.22 5.21 5.20 5.20 5.16 5.13 5.06 5.03 4.97 4.62 4.60 4.58 3.56 3.55 3.53 3.51 3.12 3.10 3.08 3.06 2.38 2.20 2.19 2.16 1.99
O
OBn
NTrt
N
(R)
N
Ns
7i
1H-NMR,CDCl3
400 MHz,298.0 K
1.00 18.36 6.16 0.95 1.00 0.98 2.16 1.02 1.06 1.02 1.15 1.01 2.16 1.06
f1 (ppm)

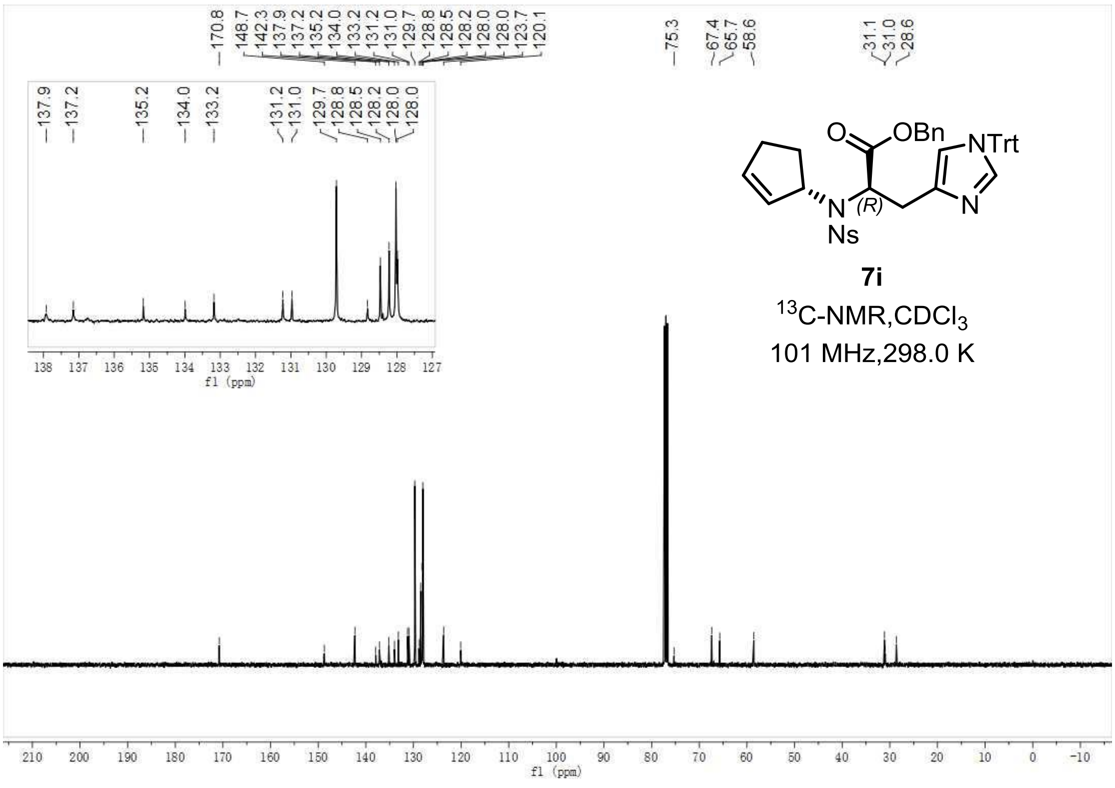
170.8 148.7 142.3 137.9 137.2 135.2 134.0 133.2 131.2 131.0 129.7 128.8 128.5 128.2 128.0 128.0 123.7 120.1 75.3 67.4 65.7 58.6 31.1 31.0 28.6
137.9 137.2 135.2 134.0 133.2 131.2 131.0 129.7 128.8 128.5 128.2 128.0 128.0
O
OBn
NTrt
N
(R)
N
Ns
7i
13C-NMR,CDCl3
101 MHz,298.0 K
f1 (ppm)

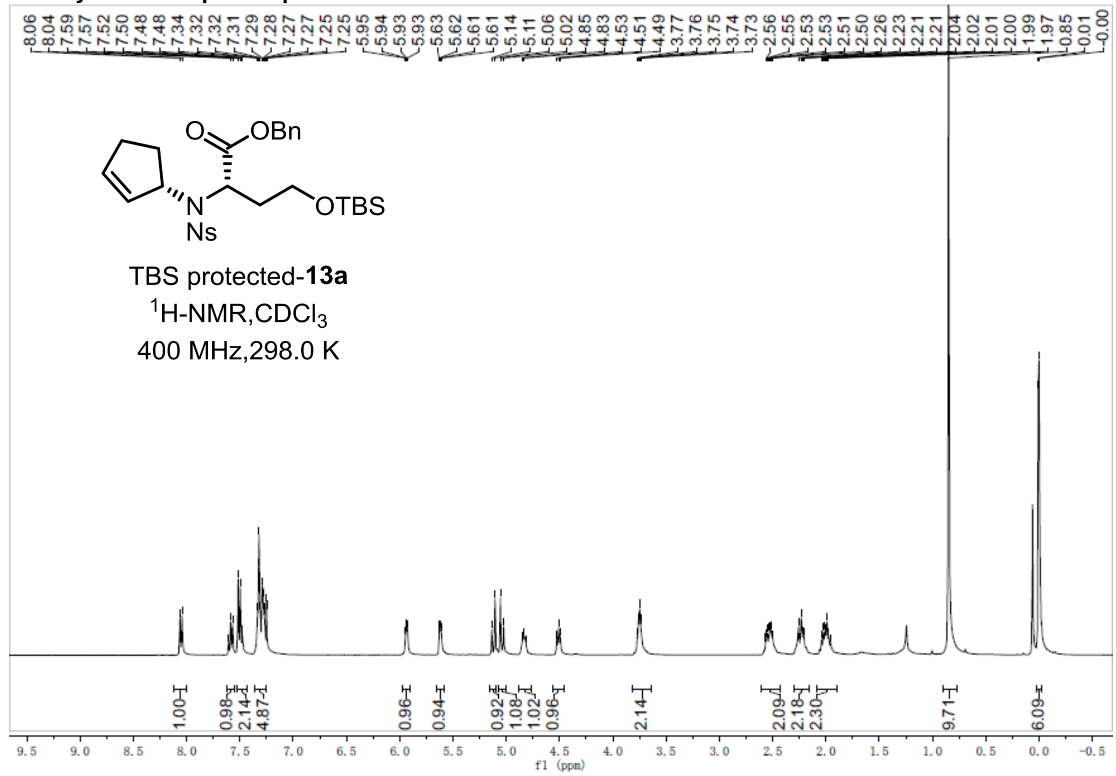
8.06 8.04 7.59 7.57 7.52 7.50 7.48 7.48 7.34 7.32 7.32 7.31 7.29 7.28 7.27 7.27 7.25 7.25 5.95 5.94 5.93 5.93 5.63 5.62 5.61 5.61 5.14 5.11 5.06 5.02 4.85 4.83 4.53 4.51 4.49 3.77 3.76 3.75 3.74 3.73 2.56 2.55 2.53 2.53 2.51 2.50 2.26 2.23 2.21 2.21 2.04 2.02 2.01 2.00 1.99 1.97 0.85 0.01 -0.00
O
OBn
N
Ns
OTBS
TBS protected-13a
1H-NMR,CDCl3
400 MHz,298.0 K
1.00 0.98 2.14 4.87 0.96 0.94 0.92 1.08 1.02 0.96 2.14 2.09 2.18 2.30 9.71 6.09
f1 (ppm)

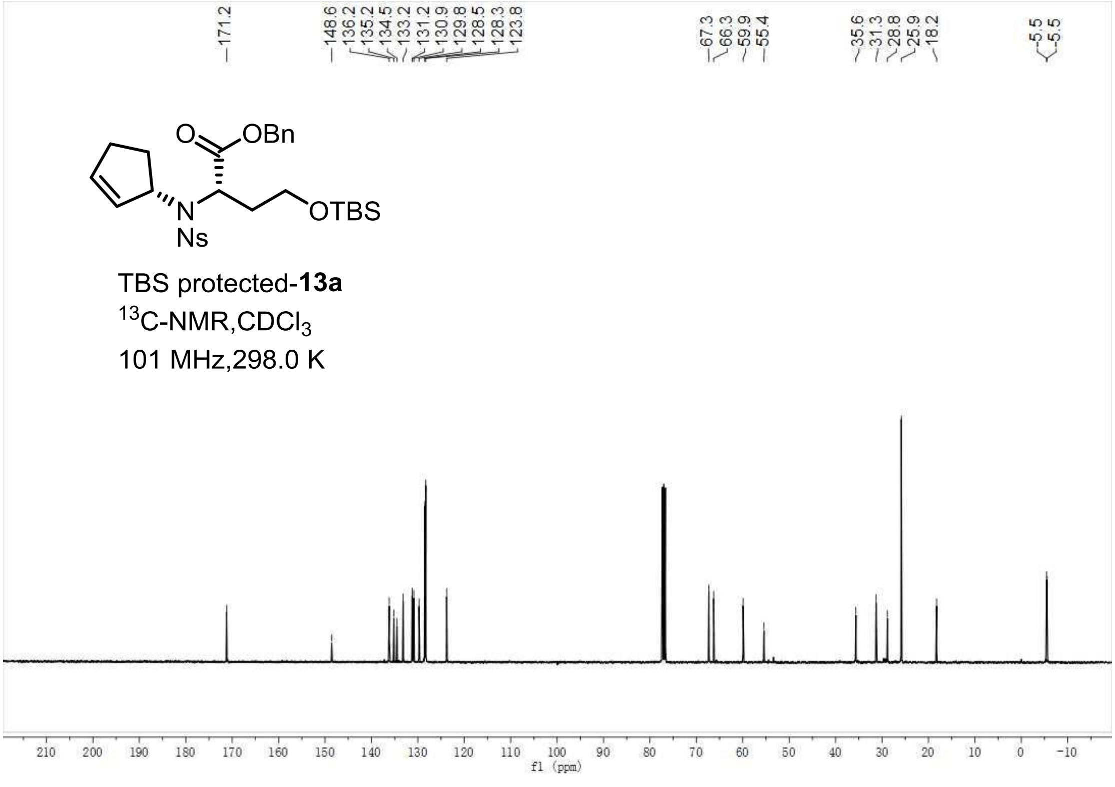
171.2
148.6 136.2 135.2 134.5 133.2 131.2 130.9 129.8 128.5 128.3 123.8
67.3 66.3 59.9 55.4
35.6 31.3 28.8 25.9 18.2
-5.5 -5.5
O
OBn
N
Ns
OTBS
TBS protected-13a
13C-NMR,CDCl3
101 MHz,298.0 K
f1 (ppm)

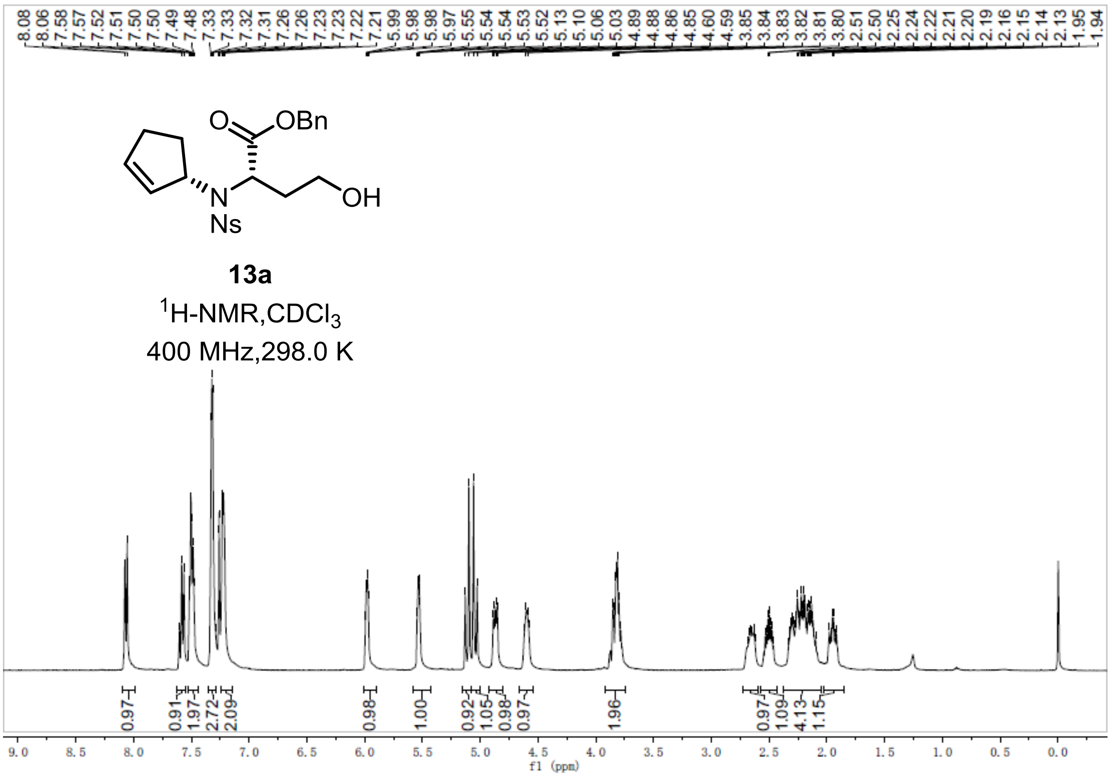

8.08 8.06 7.58 7.57 7.52 7.51 7.50 7.50 7.49 7.48 7.33 7.33 7.32 7.31 7.26 7.26 7.23 7.23 7.22 7.21 5.99 5.98 5.98 5.97 5.55 5.54 5.54 5.53 5.52 5.13 5.10 5.06 5.03 4.89 4.88 4.86 4.85 4.60 4.59 3.85 3.84 3.83 3.82 3.81 3.80 2.51 2.50 2.25 2.24 2.22 2.21 2.20 2.19 2.16 2.15 2.14 2.13 1.95 1.94
O
OBn
N
Ns
OH
13a
1H-NMR,CDCl3
400 MHz,298.0 K
0.97 0.91 1.97 2.72 2.09 0.98 1.00 0.92 1.05 0.98 0.97 1.96 0.97 1.09 4.13 1.15
f1 (ppm)

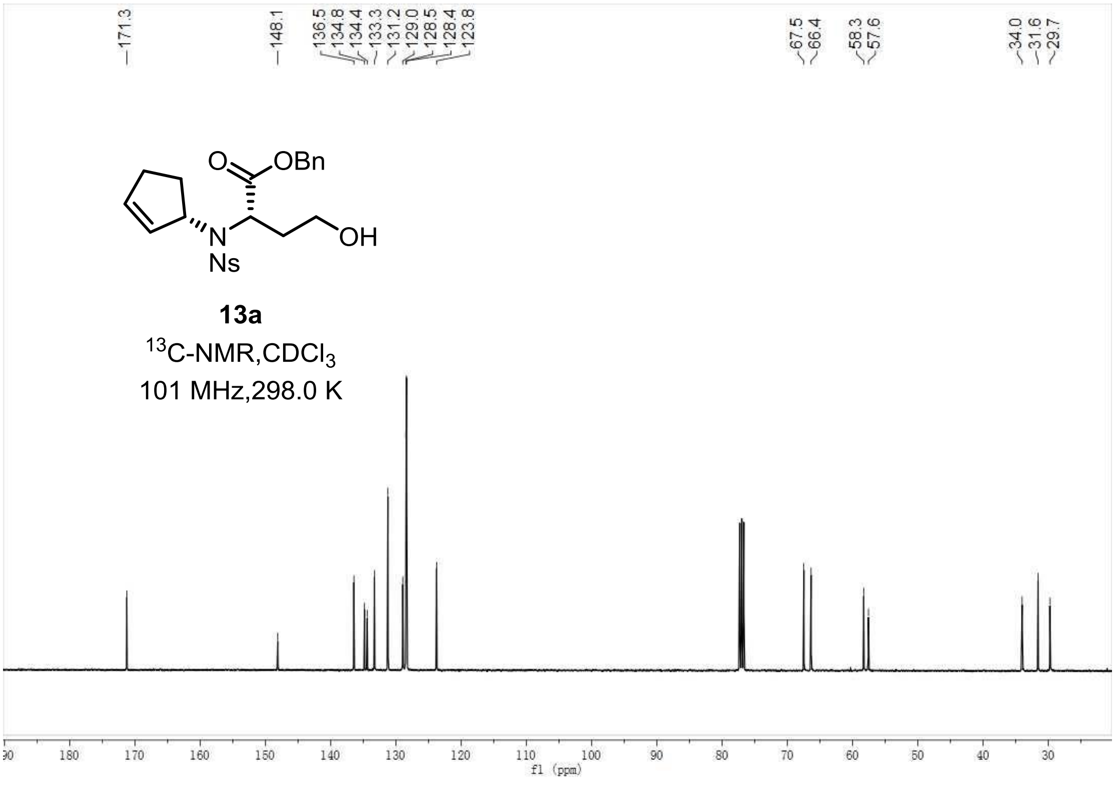

171.3 148.1 136.5 134.8 134.4 133.3 131.2 129.0 128.5 128.4 123.8 67.5 66.4 58.3 57.6 34.0 31.6 29.7
O
OBn
N
Ns
OH
13a
13C-NMR,CDCl3
101 MHz,298.0 K
f1 (ppm)

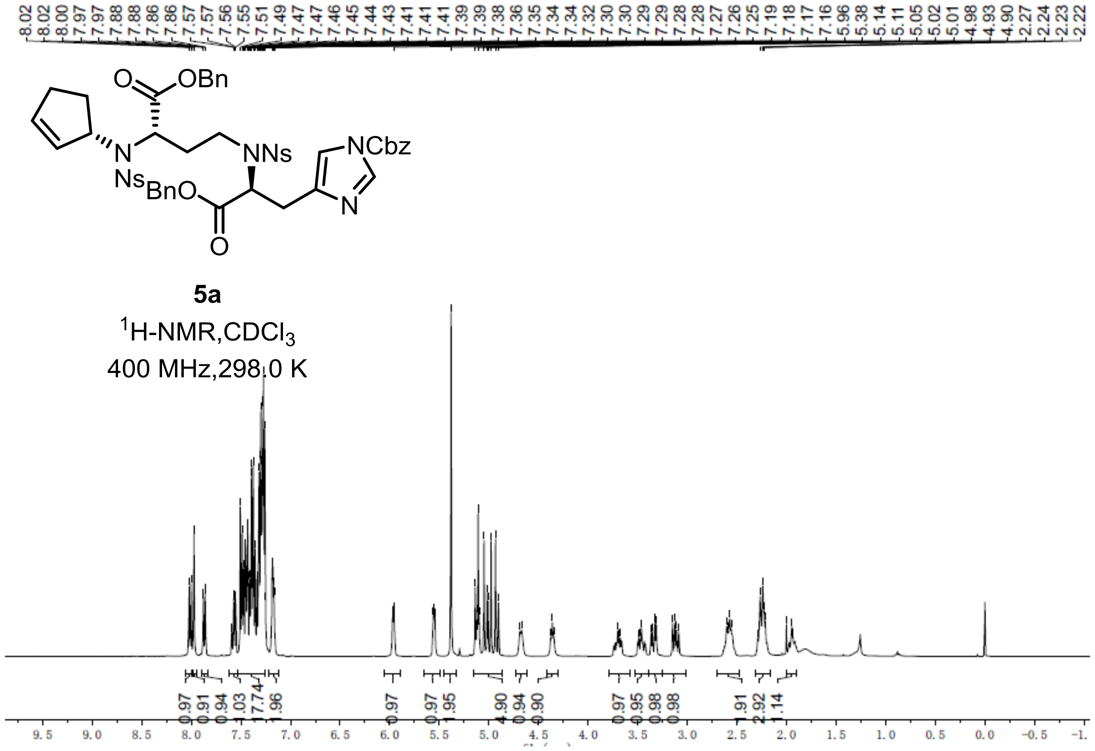
8.02
8.02
8.00
7.97
7.88
7.88
7.86
7.86
7.57
7.57
7.56
7.55
7.51
7.49
7.47
7.46
7.45
7.44
7.43
7.41
7.41
7.39
7.39
7.38
7.36
7.35
7.34
7.34
7.32
7.30
7.30
7.29
7.29
7.28
7.28
7.27
7.26
7.25
7.19
7.18
7.17
7.16
5.96
5.38
5.14
5.11
5.05
5.02
5.01
4.98
4.93
4.90
2.27
2.24
2.23
2.22
OBn
NNs
NCbz
N
Ns
BnO
N
O
5a
1H-NMR,CDCl3
400 MHz,298.0 K
0.97
0.91
0.94
1.03
17.74
1.96
0.97
0.97
1.95
4.90
0.94
0.90
0.97
0.95
0.98
0.98
1.91
2.92
1.14
9.5 9.0 8.5 8.0 7.5 7.0 6.5 6.0 5.5 5.0 4.5 4.0 3.5 3.0 2.5 2.0 1.5 1.0 0.5 0.0 -0.5 -1.

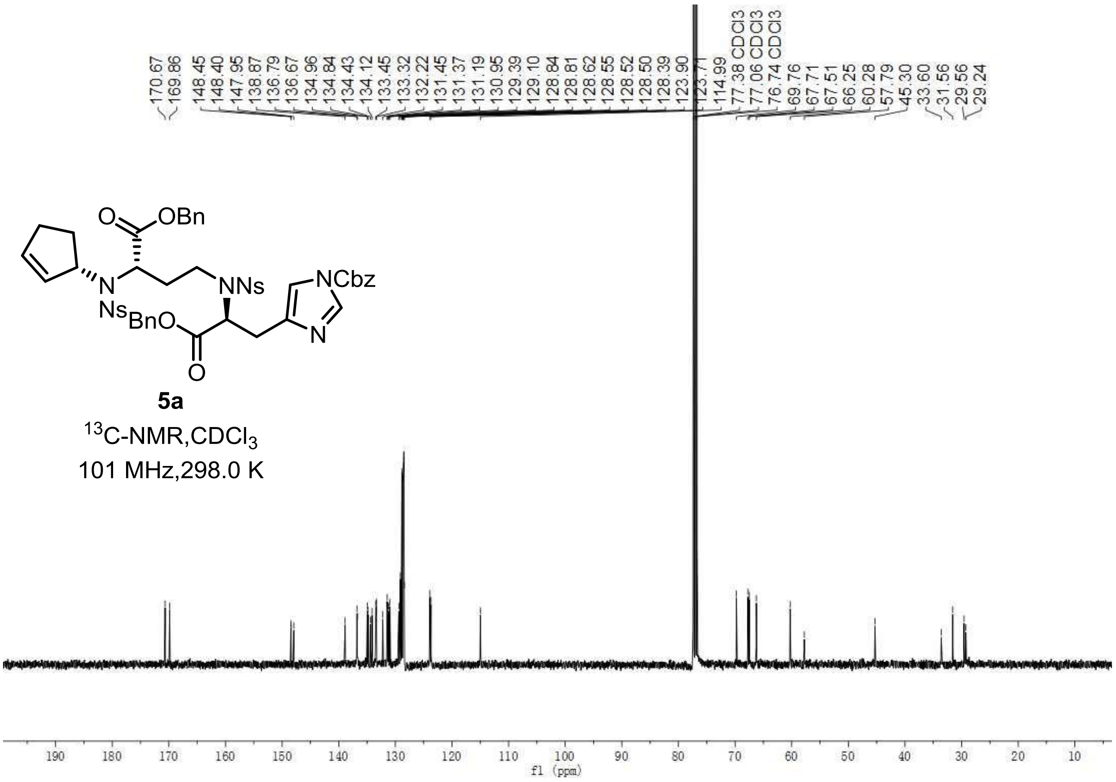
170.67
169.86
148.45
148.40
147.95
138.87
136.79
136.67
134.96
134.84
134.43
134.12
133.45
133.32
132.22
131.45
131.37
131.19
130.95
129.39
129.10
128.84
128.81
128.62
128.55
128.52
128.50
128.39
123.90
123.71
114.99
77.38 CDCl3
77.06 CDCl3
76.74 CDCl3
69.76
67.71
67.51
66.25
60.28
57.79
45.30
33.60
31.56
29.56
29.24
OBn
NNs
NCbz
N
Ns
BnO
N
O
5a
13C-NMR,CDCl3
101 MHz,298.0 K
190 180 170 160 150 140 130 120 110 100 90 80 70 60 50 40 30 20 10
f1 (ppm)

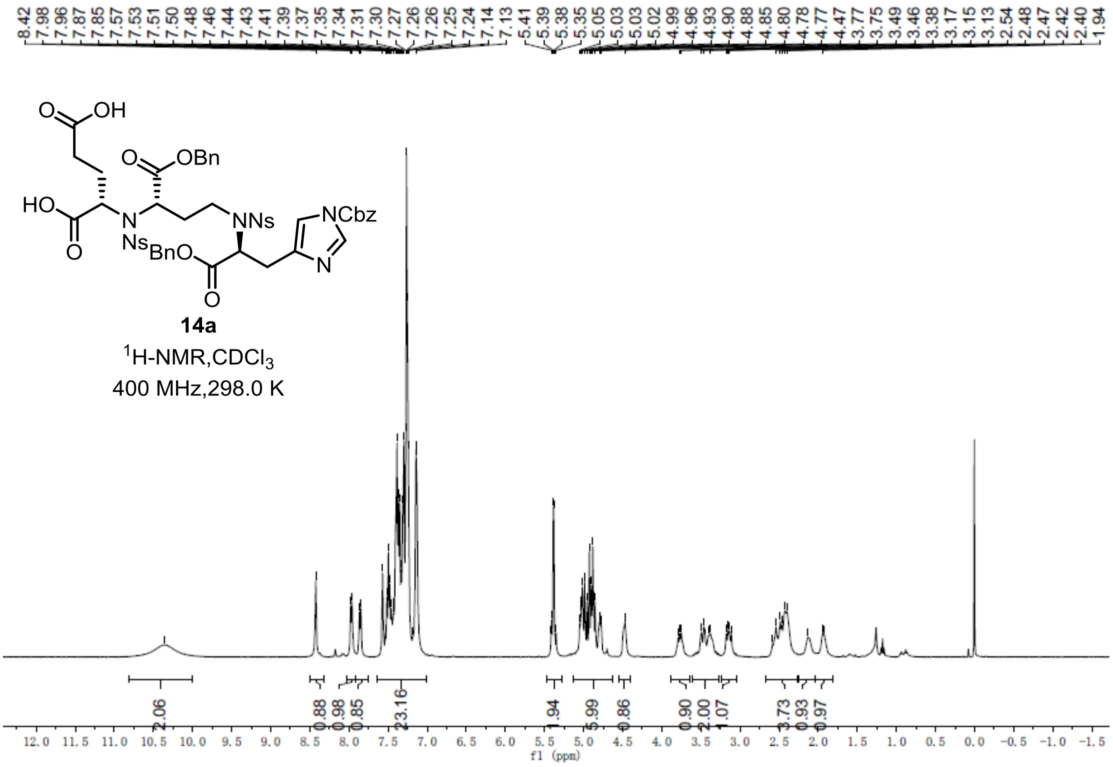

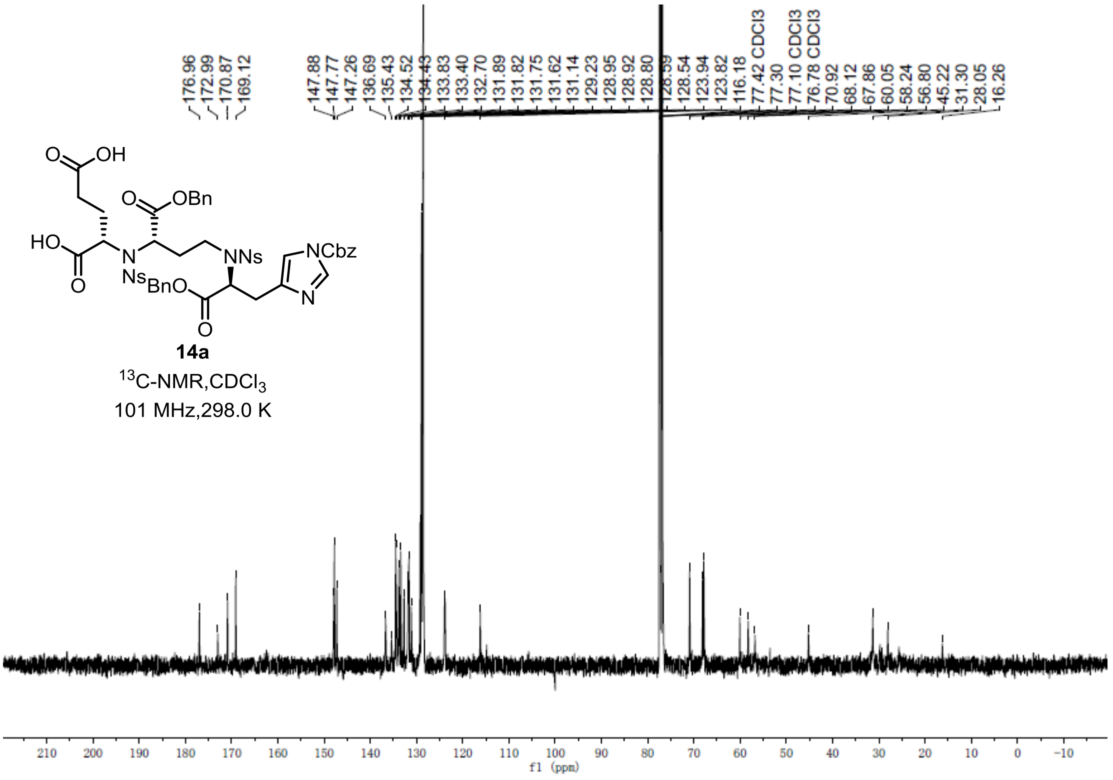

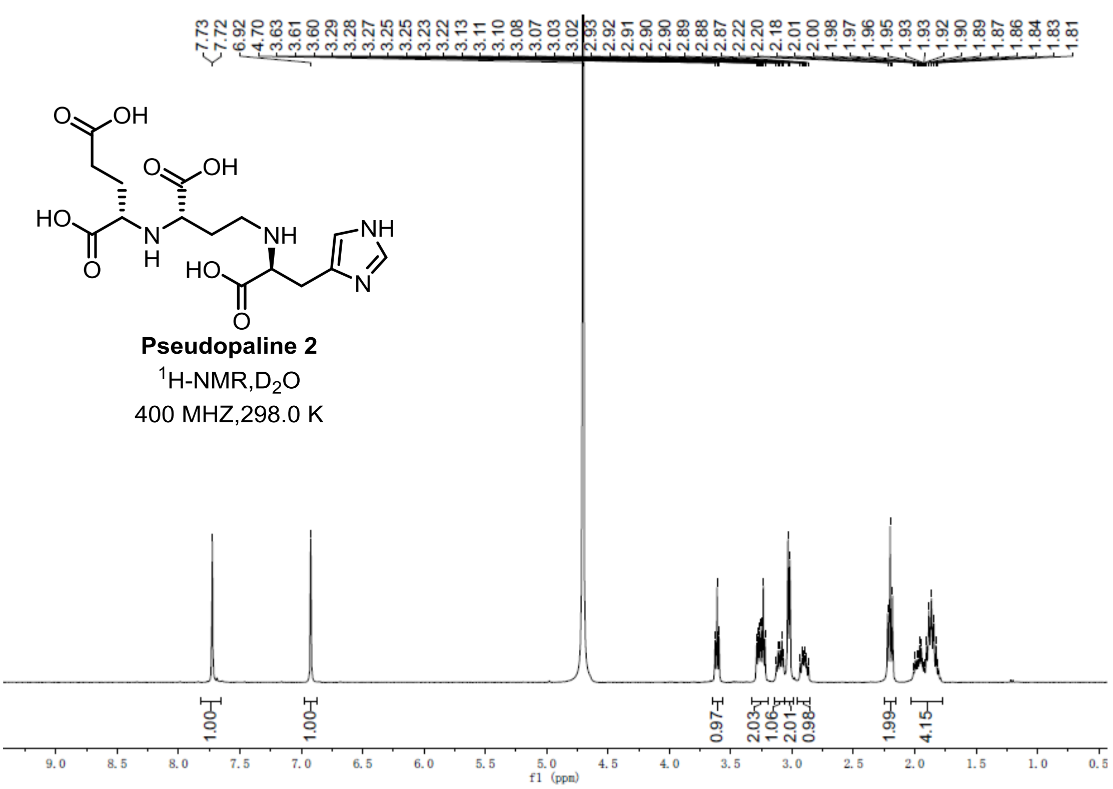
7.73
7.72
6.92
4.70
3.63
3.61
3.60
3.29
3.28
3.27
3.25
3.25
3.23
3.22
3.13
3.11
3.10
3.08
3.07
3.03
3.02
2.93
2.92
2.91
2.90
2.90
2.89
2.88
2.87
2.22
2.20
2.18
2.01
2.00
1.98
1.97
1.96
1.95
1.93
1.93
1.92
1.90
1.89
1.87
1.86
1.84
1.83
1.81
Pseudopaline 2
1H-NMR,D2O
400 MHZ,298.0 K
1.00
1.00
0.97
2.03
1.06
2.01
0.98
1.99
4.15
f1 (ppm)

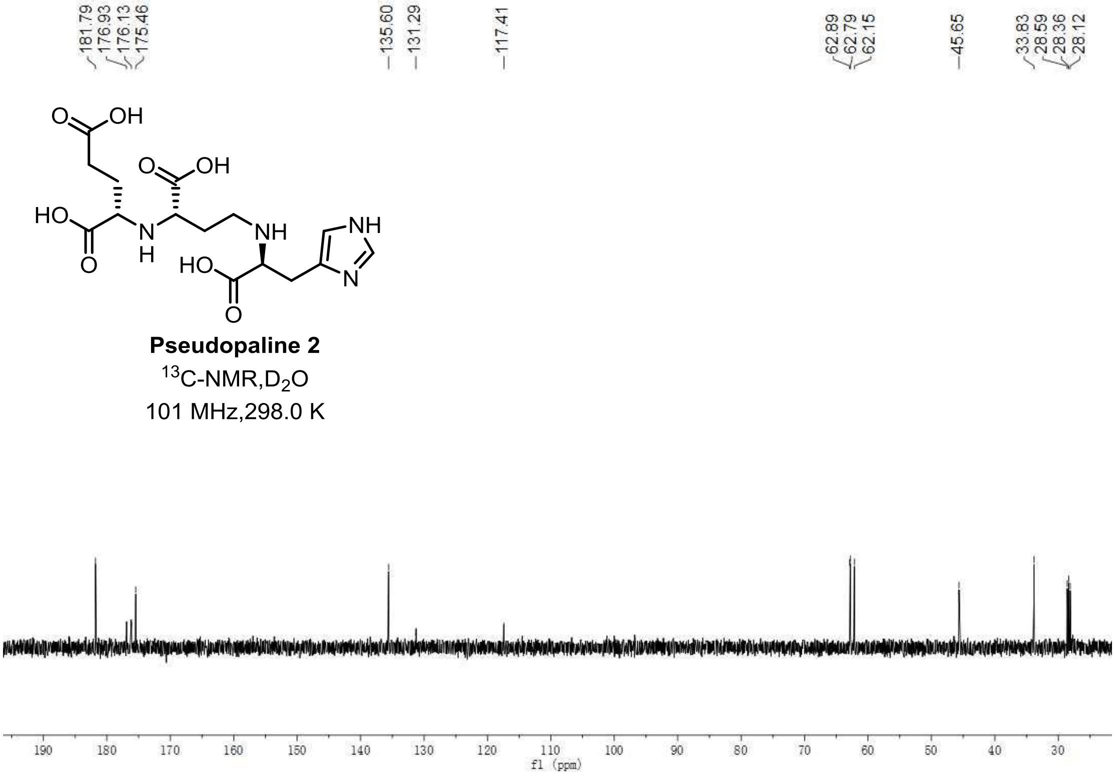
181.79
176.93
176.13
175.46
135.60
131.29
117.41
62.89
62.79
62.15
45.65
33.83
28.59
28.36
28.12
Pseudopaline 2
13C-NMR,D2O
101 MHz,298.0 K
f1 (ppm)

Total synthesis of *epi*-pseudopaline

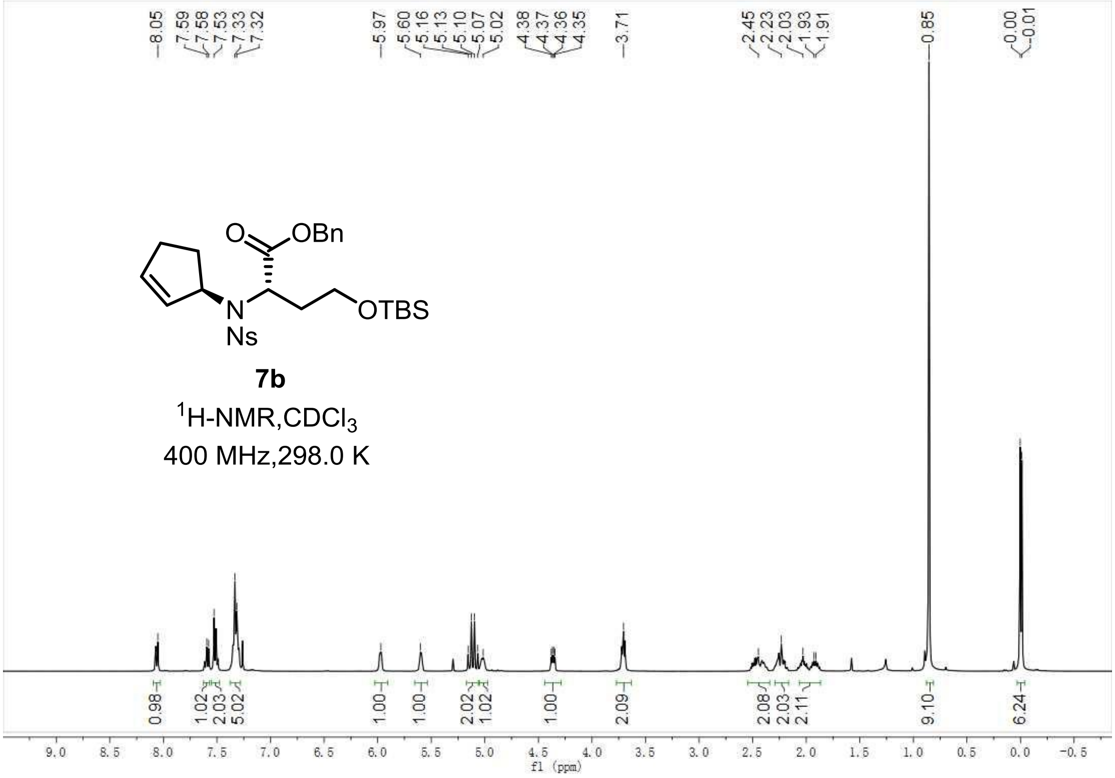

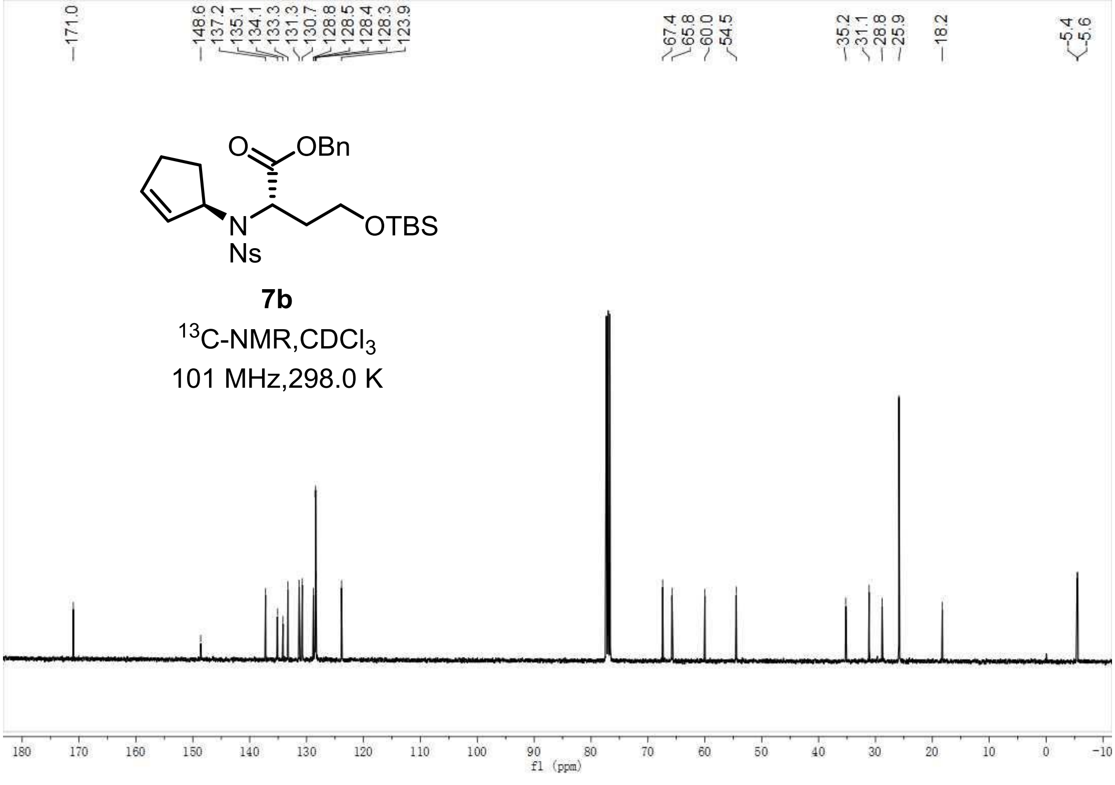

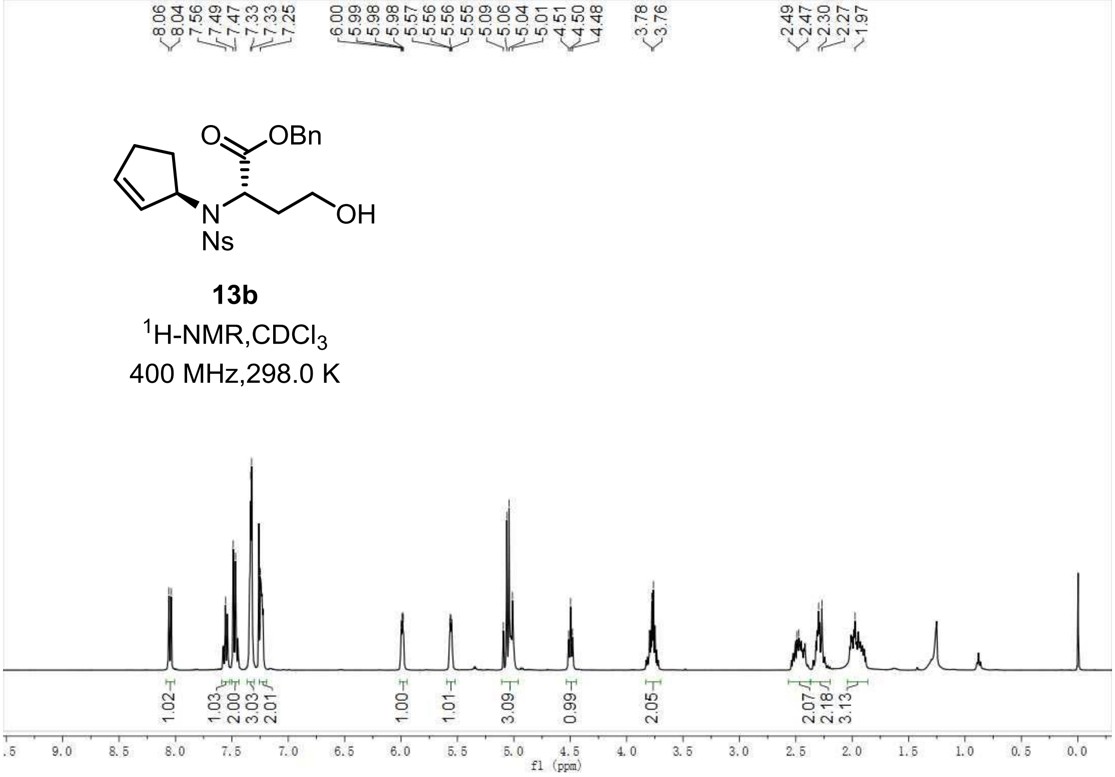

OBn
N
OH
Ns
13b
1H-NMR,CDCl3
400 MHz,298.0 K
f1 (ppm)

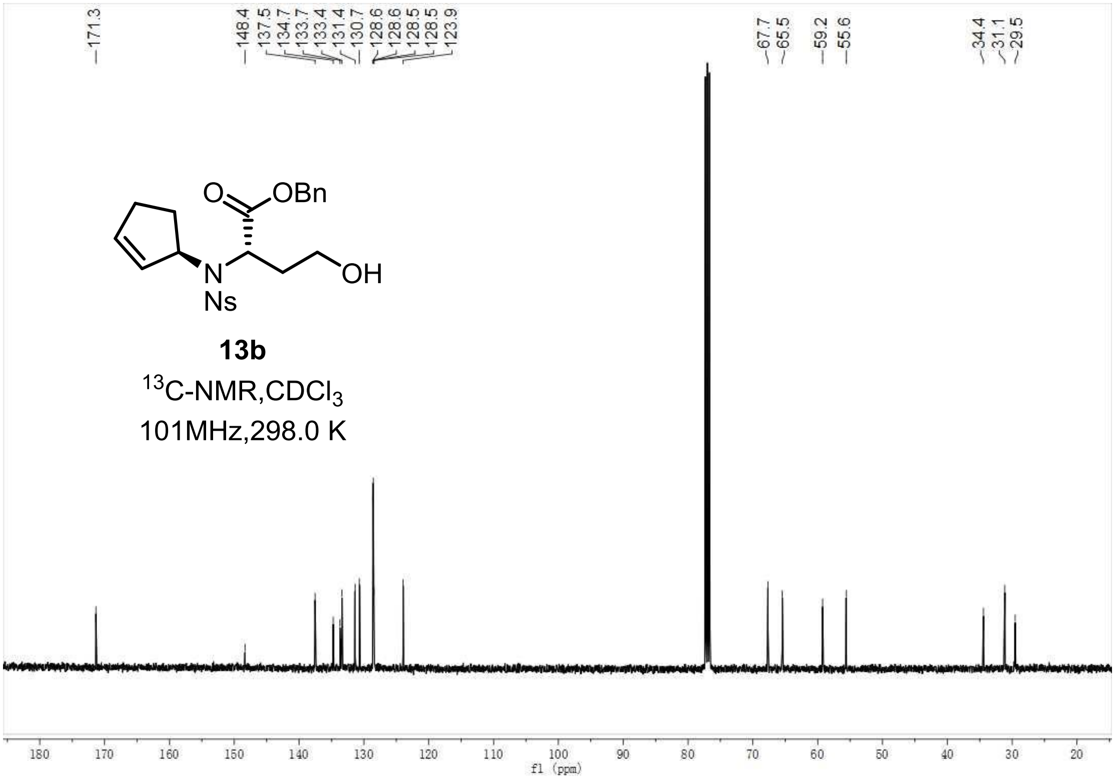

OBn
N
OH
Ns
13b
13C-NMR,CDCl3
101MHz,298.0 K
f1 (ppm)

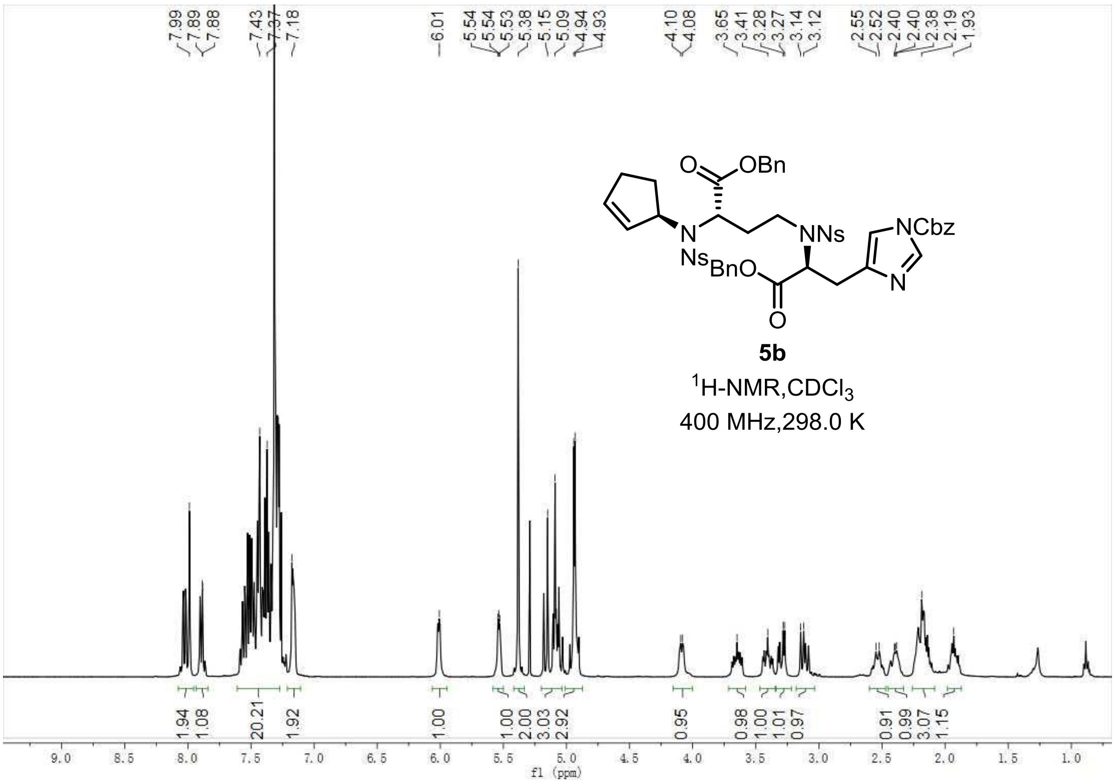

O
OBn
N
Ns
NNs
BnO
O
NCbz
N
5b
1H-NMR,CDCl3
400 MHz,298.0 K
f1 (ppm)

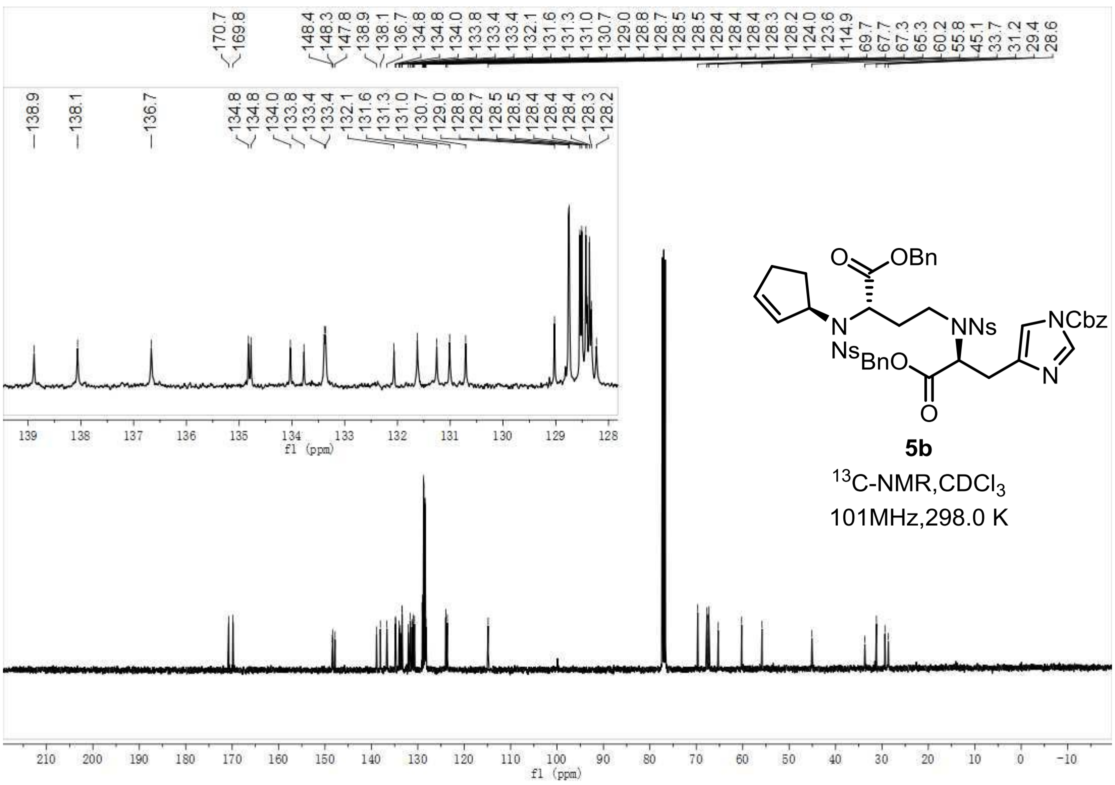

O
OBn
N
Ns
NNs
BnO
O
NCbz
N
5b
13C-NMR,CDCl3
101MHz,298.0 K
f1 (ppm)

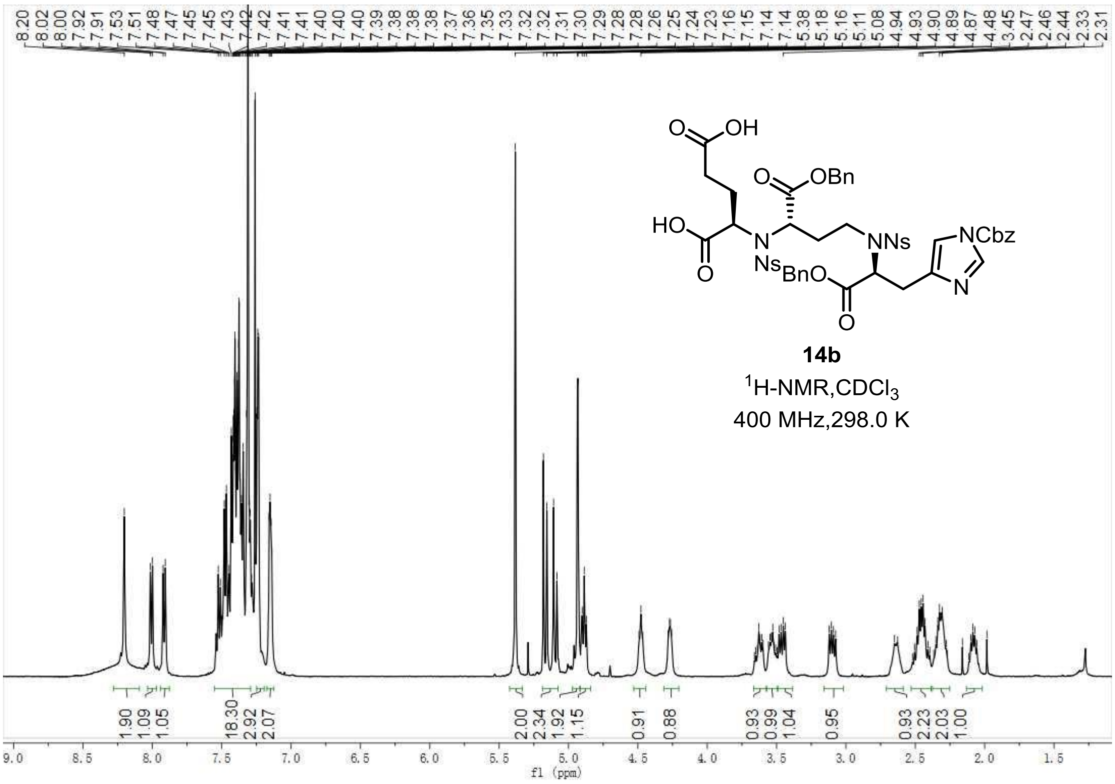
8.20
8.02
8.00
7.92
7.91
7.53
7.51
7.48
7.47
7.45
7.45
7.43
7.42
7.42
7.41
7.41
7.40
7.40
7.40
7.39
7.38
7.38
7.38
7.37
7.36
7.35
7.33
7.32
7.32
7.32
7.31
7.30
7.29
7.28
7.28
7.26
7.25
7.24
7.23
7.16
7.15
7.14
7.14
5.38
5.18
5.16
5.11
5.08
4.94
4.93
4.90
4.89
4.87
4.48
3.45
2.47
2.46
2.44
2.33
2.31
O
OH
O
OBn
HO
N
NNs
NCbz
Ns
BnO
O
N
O
14b
^{1}H-NMR,CDCl$_3$
400 MHz,298.0 K
1.90
1.09
1.05
18.30
2.92
2.07
2.00
2.34
1.92
1.15
0.91
0.88
0.93
0.99
1.04
0.95
0.93
2.23
2.03
1.00
f1 (ppm)

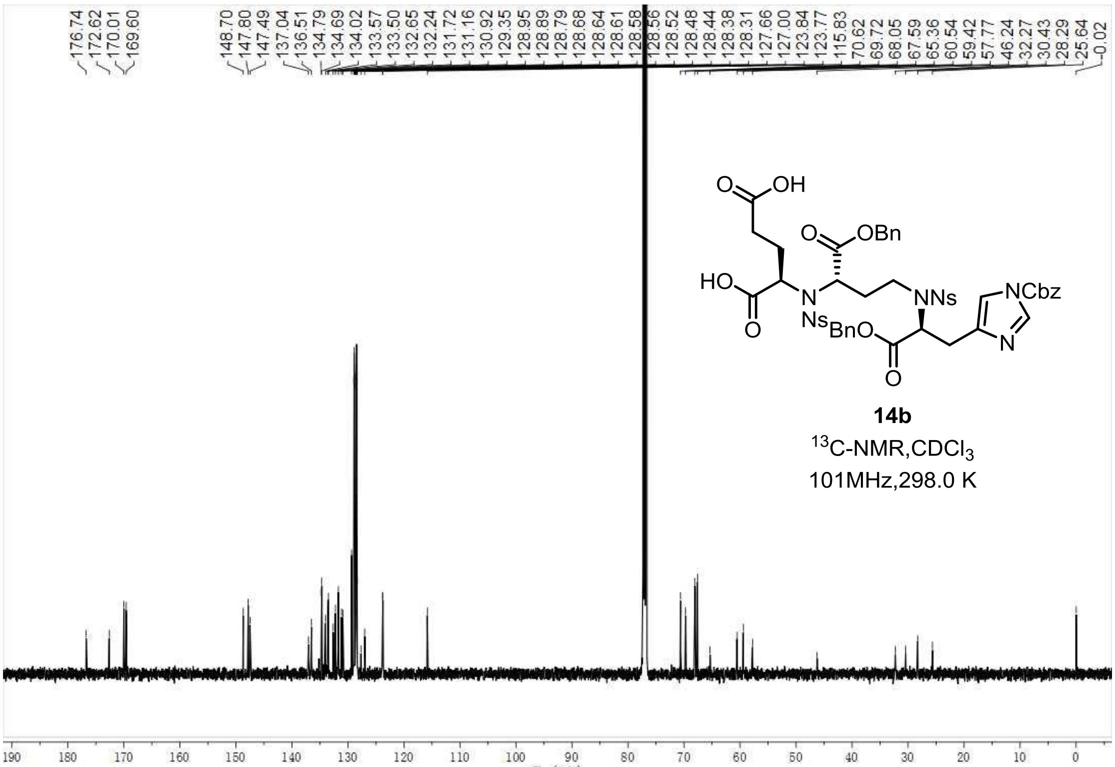
176.74
172.62
170.01
169.60
148.70
147.80
147.49
137.04
136.51
134.79
134.69
134.02
133.57
133.50
132.65
132.24
131.72
131.16
130.92
129.35
128.95
128.89
128.79
128.68
128.64
128.61
128.58
128.56
128.52
128.48
128.44
128.38
128.31
127.66
127.00
123.84
123.77
115.83
70.62
69.72
68.05
67.59
65.36
60.54
59.42
57.77
46.24
32.27
30.43
28.29
25.64
-0.02
O
OH
O
OBn
HO
N
NNs
NCbz
Ns
BnO
O
N
O
14b
^{13}C-NMR,CDCl$_3$
101MHz,298.0 K
f1 (ppm)

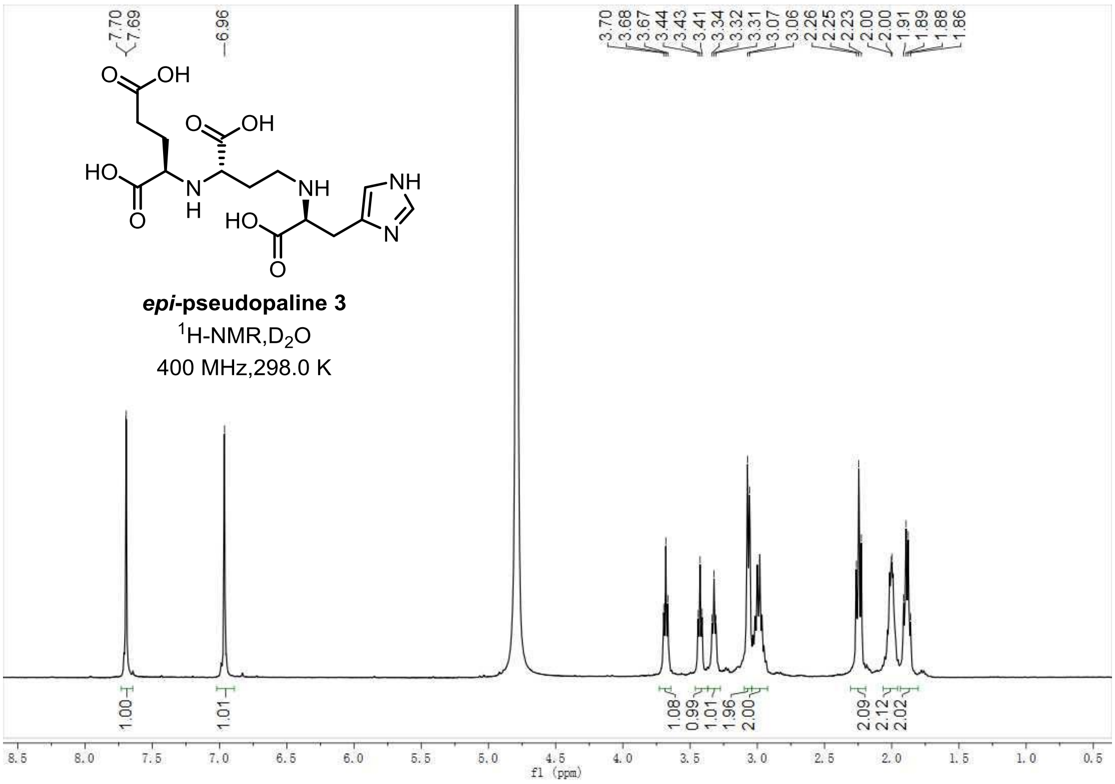
epi-pseudopaline 3
1H-NMR,D2O
400 MHz,298.0 K
f1 (ppm)

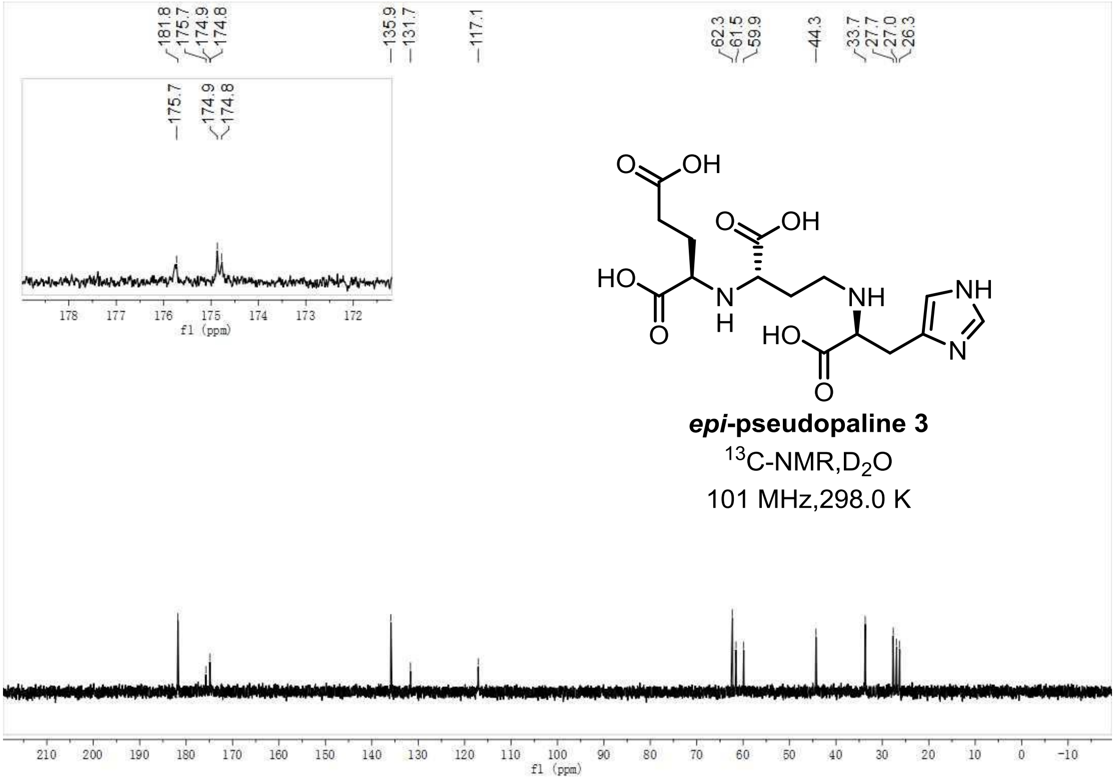
epi-pseudopaline 3
13C-NMR,D2O
101 MHz,298.0 K
f1 (ppm)

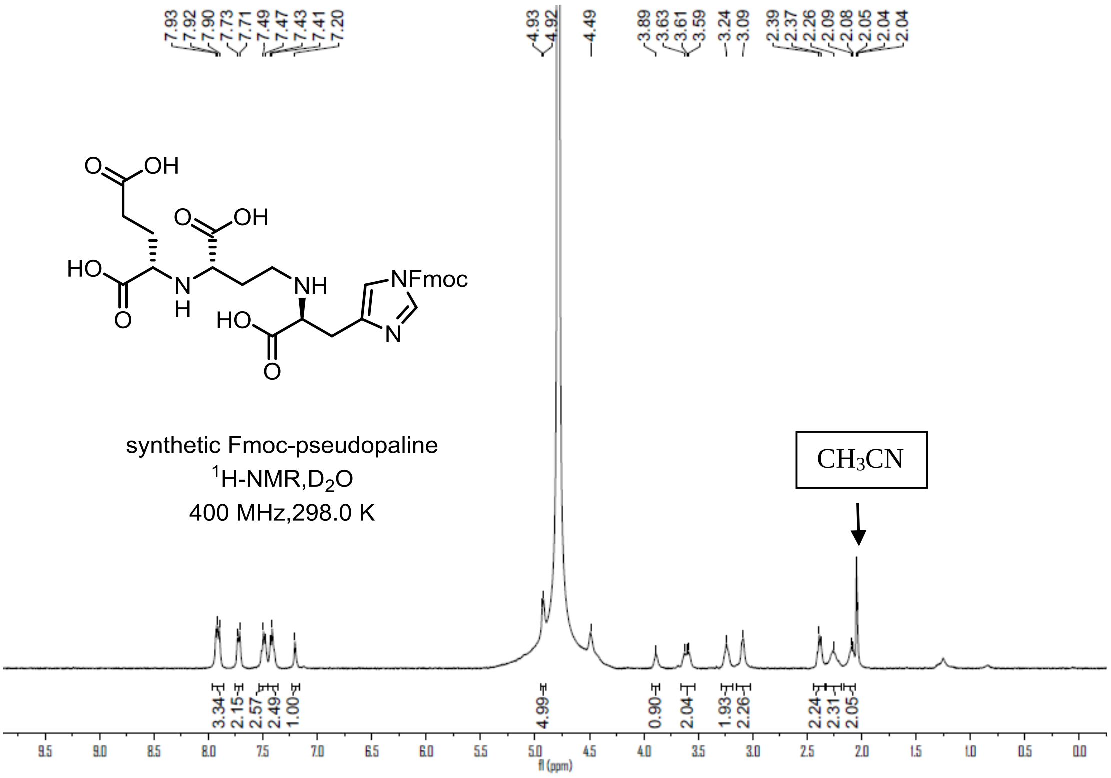
7.93 7.92 7.90 7.73 7.71 7.49 7.47 7.43 7.41 7.20
4.93 4.92 4.49
3.89 3.63 3.61 3.59 3.24 3.09
2.39 2.37 2.26 2.09 2.08 2.05 2.04 2.04
OH
O
HO
N
H
NH
NFmoc
N
synthetic Fmoc-pseudopaline
1H-NMR,D2O
400 MHz,298.0 K
CH3CN
3.34 2.15 2.57 2.49 1.00
4.99
0.90 2.04 1.93 2.26
2.24 2.31 2.05
f1 (ppm)

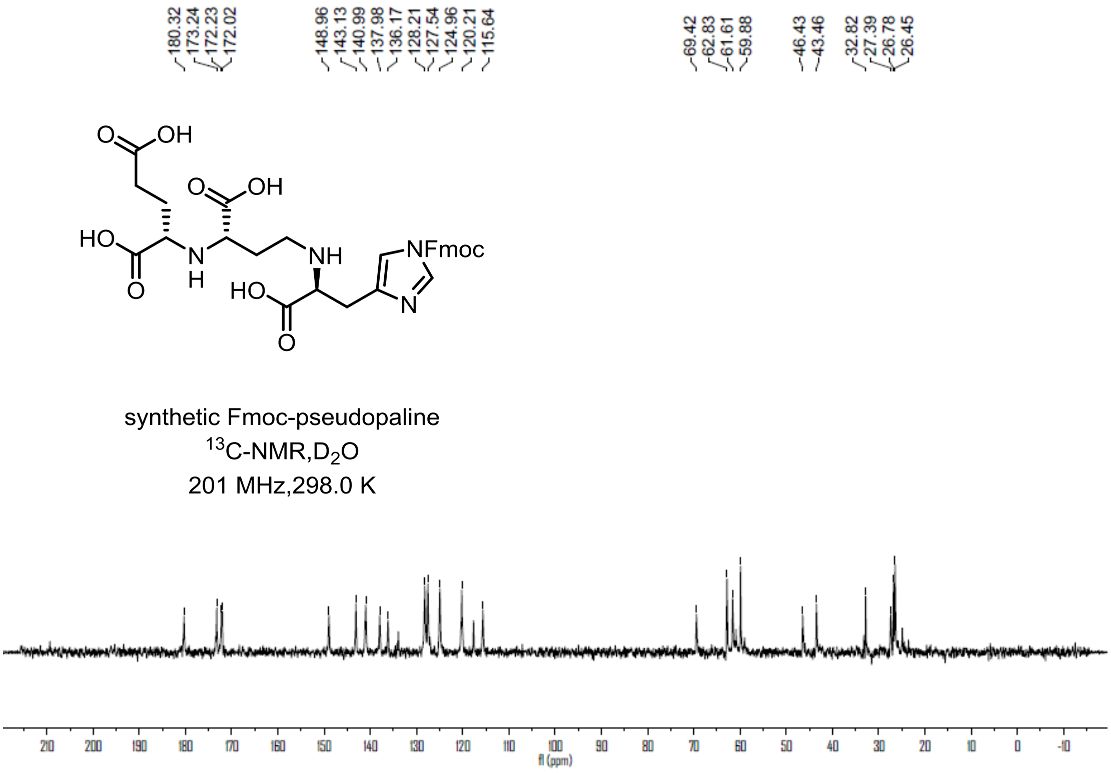
180.32 173.24 172.23 172.02
148.96 143.13 140.99 137.98 136.17 128.21 127.54 124.96 120.21 115.64
69.42 62.83 61.61 59.88
46.43 43.46
32.82 27.39 26.78 26.45
OH
O
HO
N
H
NH
NFmoc
N
synthetic Fmoc-pseudopaline
13C-NMR,D2O
201 MHz,298.0 K
f1 (ppm)

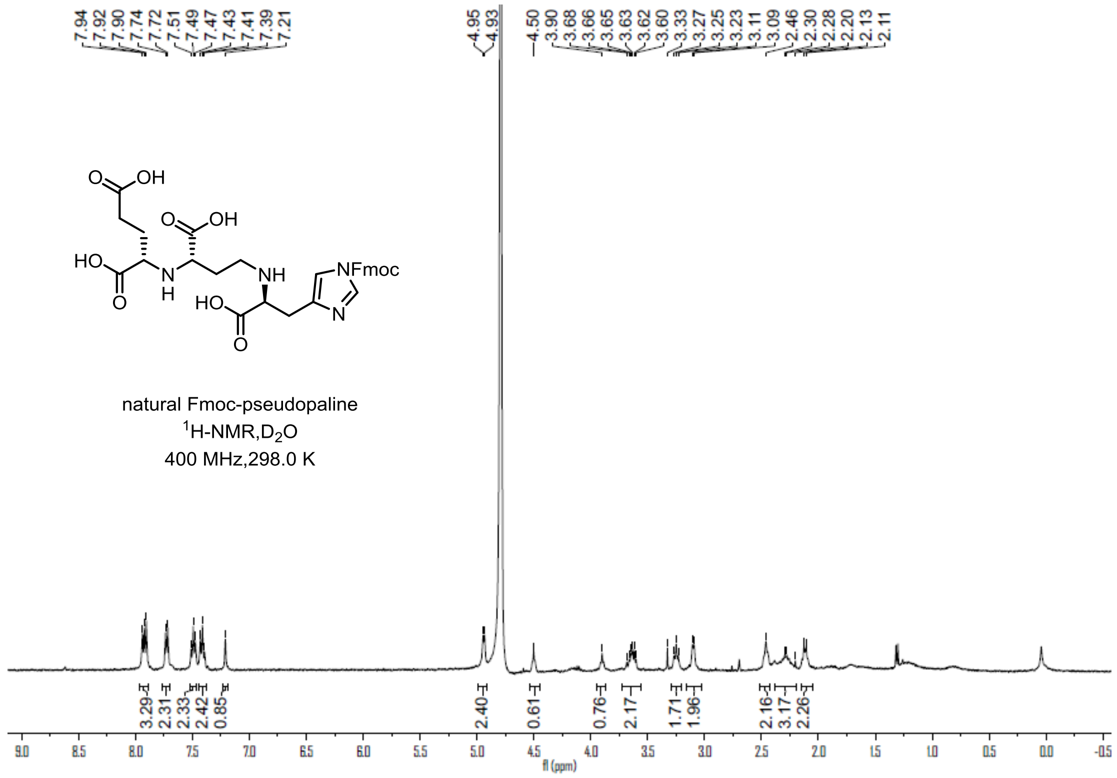
7.94 7.92 7.90 7.74 7.72 7.51 7.49 7.47 7.43 7.41 7.39 7.21
4.95 4.93
4.50 3.90 3.68 3.66 3.65 3.63 3.62 3.60 3.33 3.27 3.25 3.23 3.11 3.09 2.46 2.30 2.28 2.20 2.13 2.11
natural Fmoc-pseudopaline
1H-NMR,D2O
400 MHz,298.0 K
3.29 2.31 2.33 2.42 0.85 2.40 0.61 0.76 2.17 1.71 1.96 2.16 3.17 2.26
f1 (ppm)

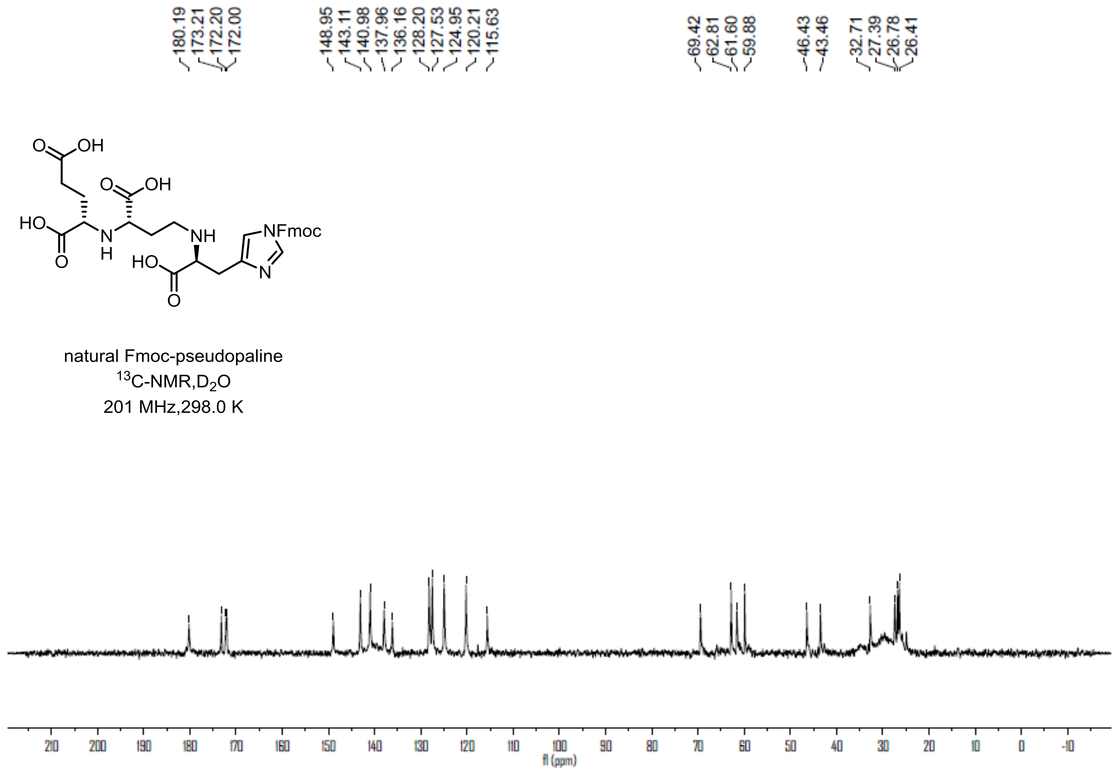
180.19 173.21 172.20 172.00
148.95 143.11 140.98 137.96 136.16 128.20 127.53 124.95 120.21 115.63
69.42 62.81 61.60 59.88
46.43 43.46
32.71 27.39 26.78 26.41
natural Fmoc-pseudopaline
13C-NMR,D2O
201 MHz,298.0 K
f1 (ppm)

Comparison of synthetic and natural Fmoc-pseudopaline

^{1}H NMR comparison of synthetic and natural Fmoc-pseudopaline

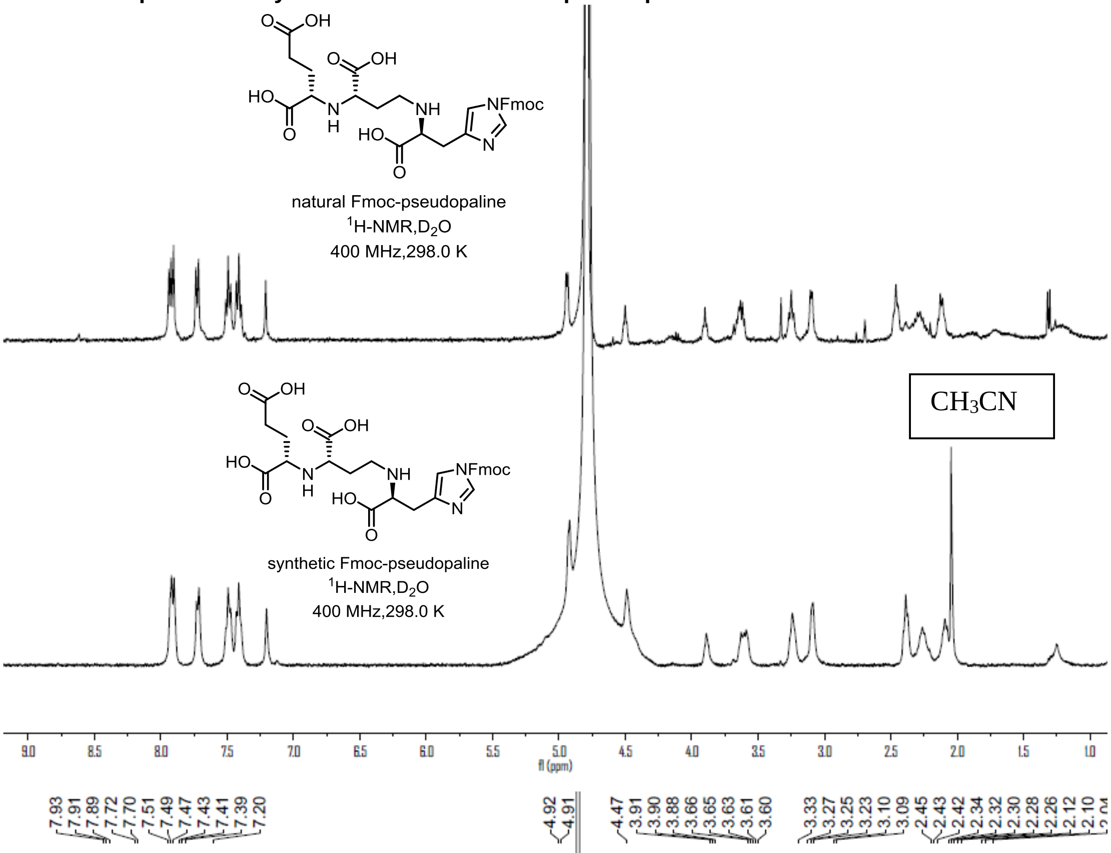

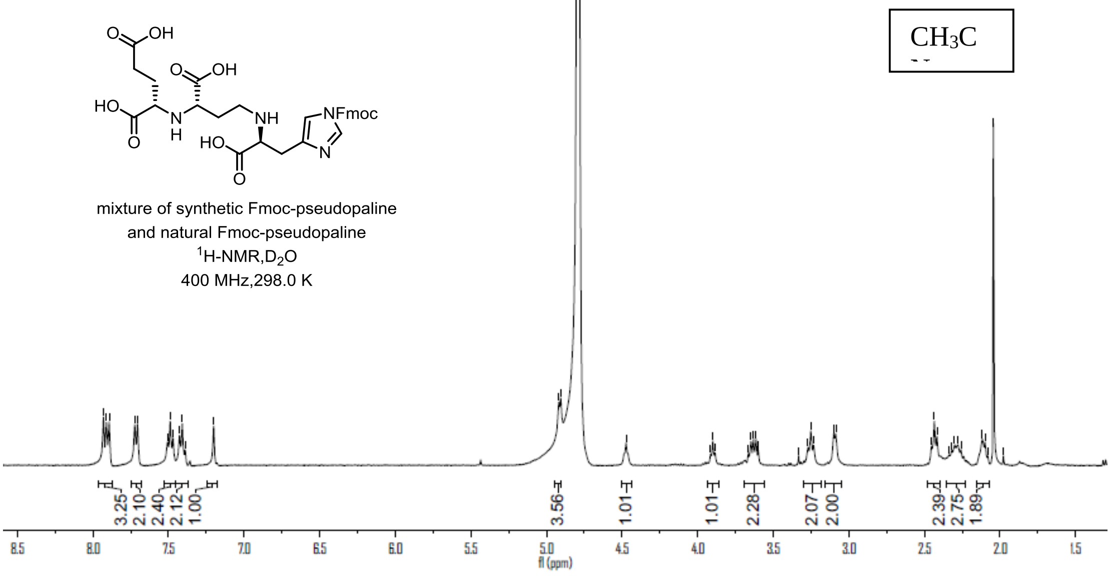

^{13}C NMR comparison of synthetic and natural Fmoc-pseudopaline

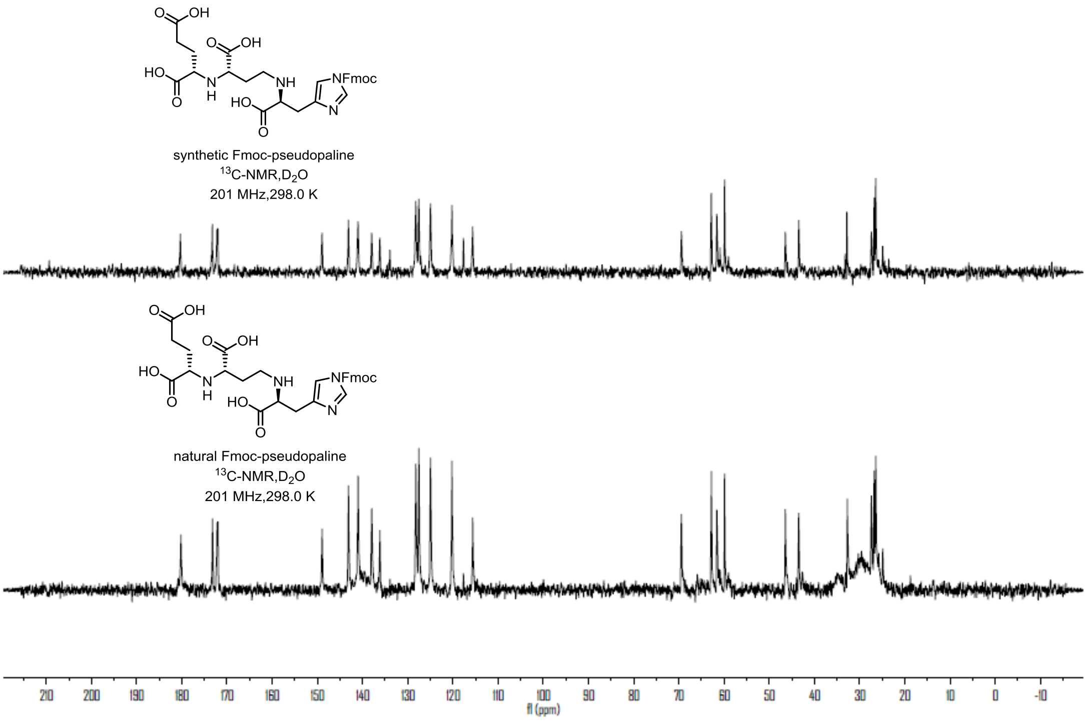

HRMS comparison of synthetic and natural Fmoc-pseudopaline

$[M+H]^+$

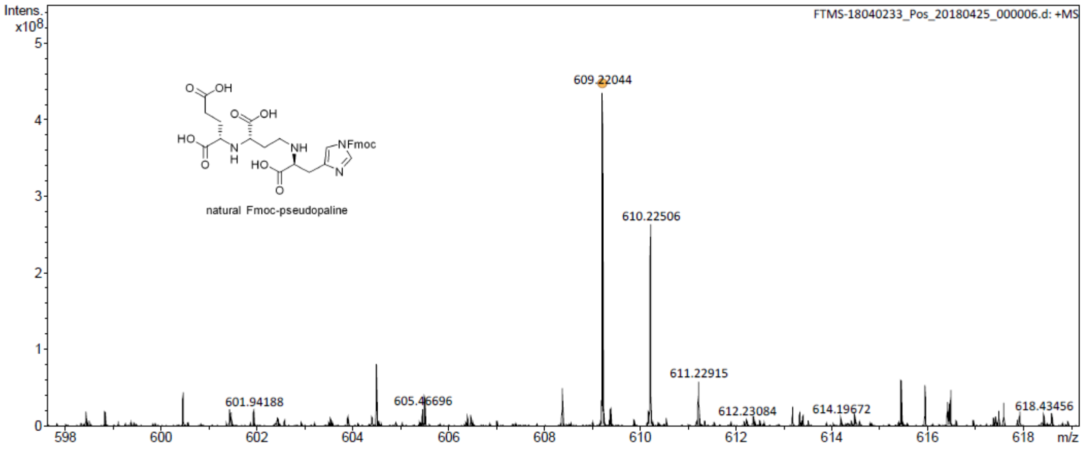

Meas. m/z	#	Ion Formula	Score	m/z	err [ppm]	Mean err [ppm]	mSigma	rdb	e⁻ Conf	N-Rule
609.220445	1	C30H33N4O10	100.00	609.219120	-2.2	-4.1	128.7	17.0	even	ok

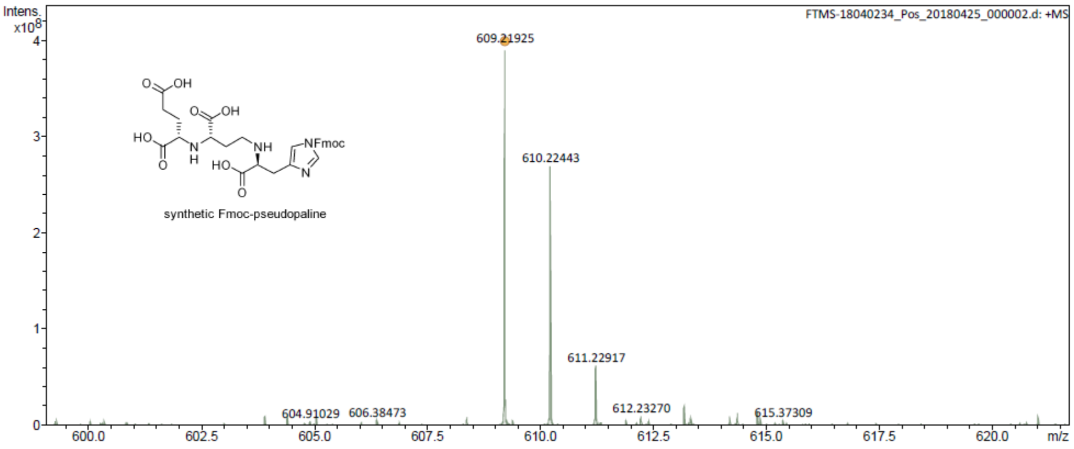

Meas. m/z	#	Ion Formula	Score	m/z	err [ppm]	Mean err [ppm]	mSigma	rdb	e⁻ Conf	N-Rule
609.219247	1	C30H33N4O10	100.00	609.219120	-0.2	-3.5	172.9	17.0	even	ok

[M-H]$^-$

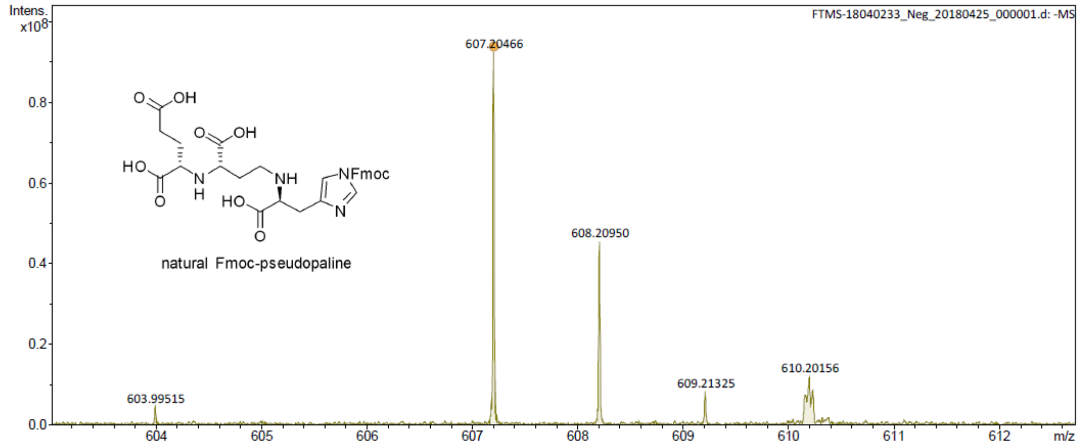

Meas. m/z	#	Ion Formula	Score	m/z	err [ppm]	Mean err [ppm]	mSigma	rdb	e⁻ Conf	N-Rule
607.204664	1	C30H31N4O10	100.00	607.204567	-0.2	-1.6	74.0	17.0	even	ok

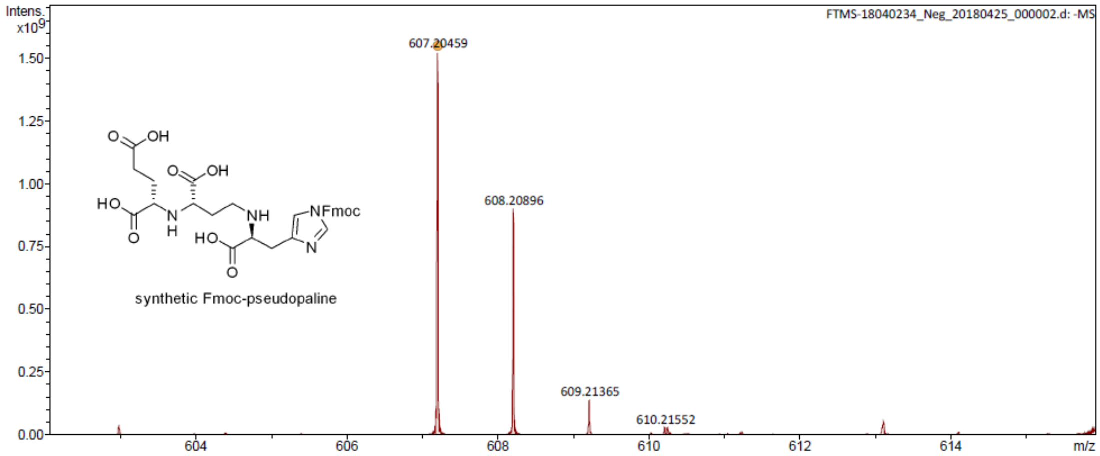

Meas. m/z	#	Ion Formula	Score	m/z	err [ppm]	Mean err [ppm]	mSigma	rdb	e⁻ Conf	N-Rule
607.204588	1	C30H31N4O10	100.00	607.204567	-0.0	-2.2	122.0	17.0	even	ok

^{1}H and ^{13}C NMR spectra of analogs 15a, 15b, and 15c

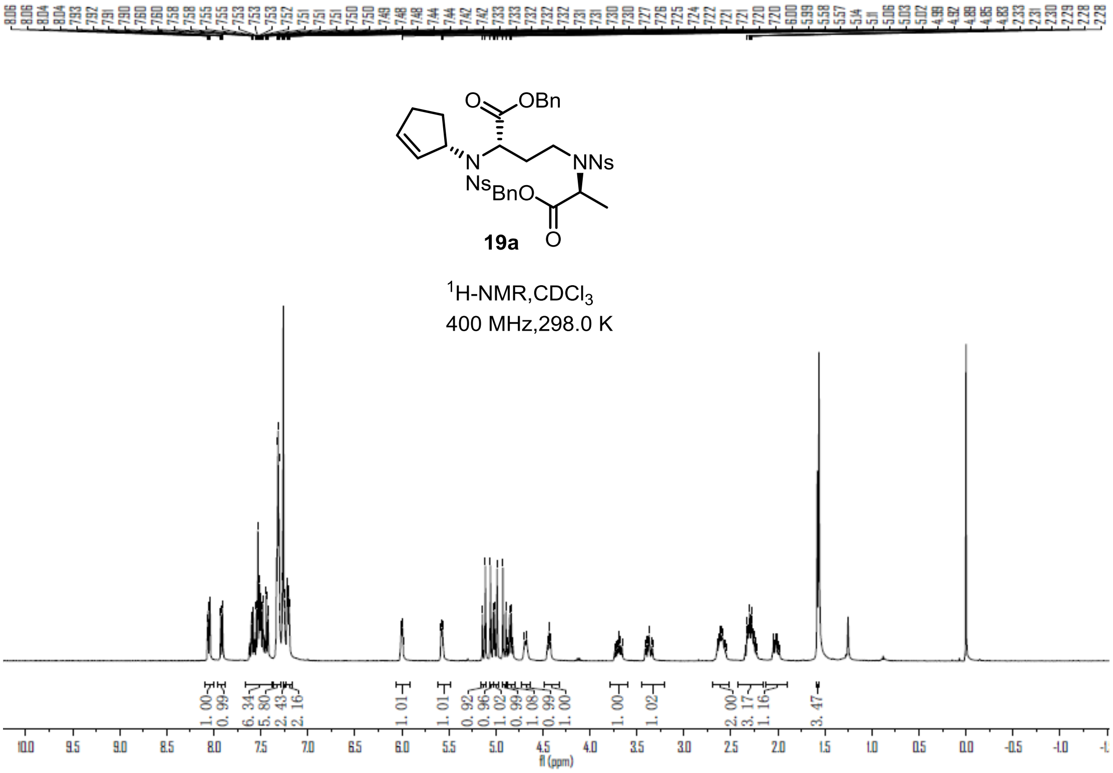

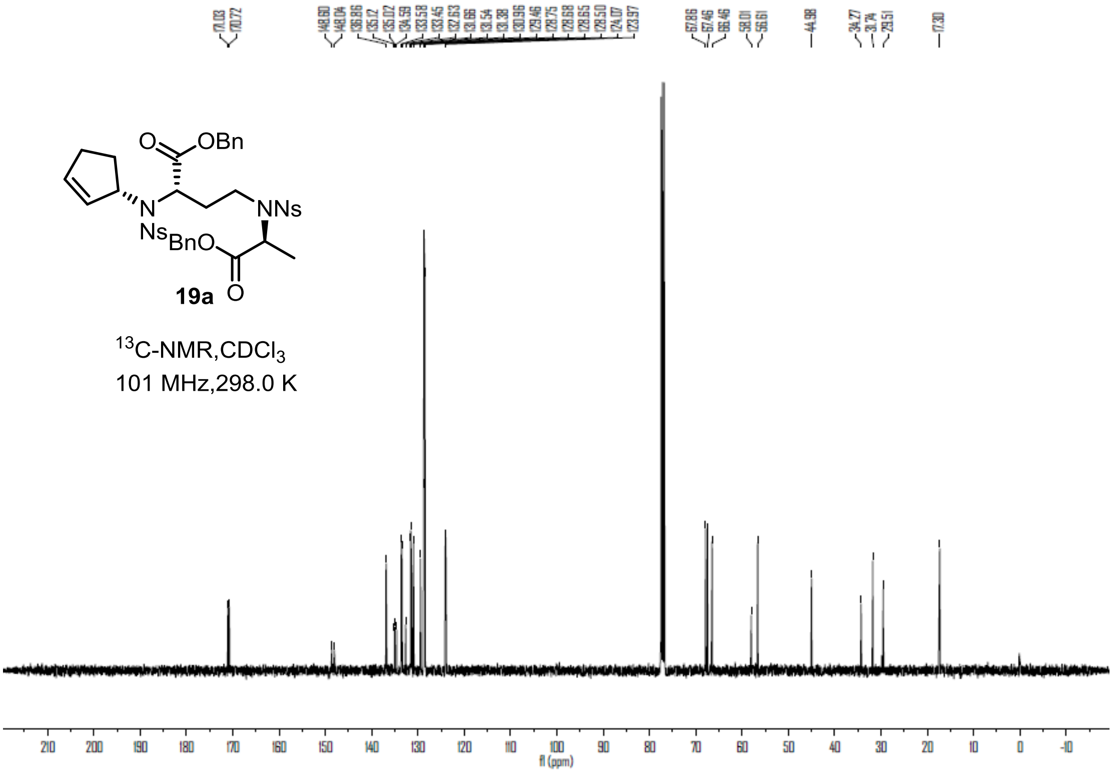

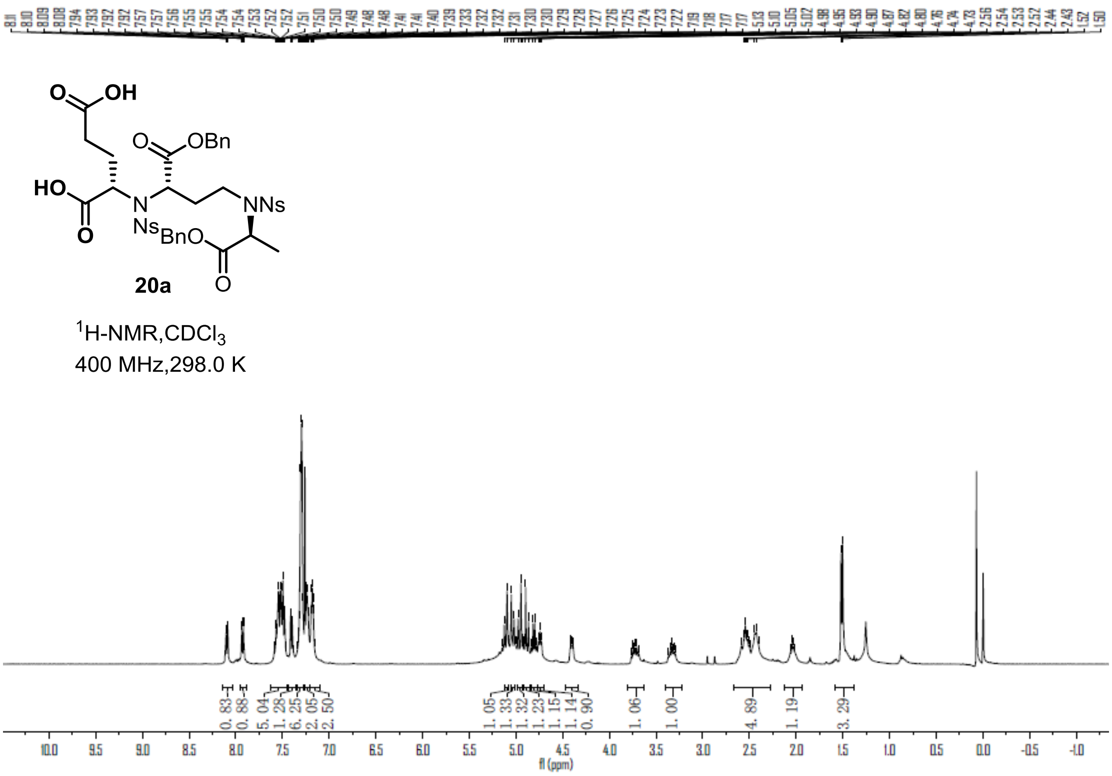

20a

^{1}H-NMR,CDCl$_3$
400 MHz,298.0 K

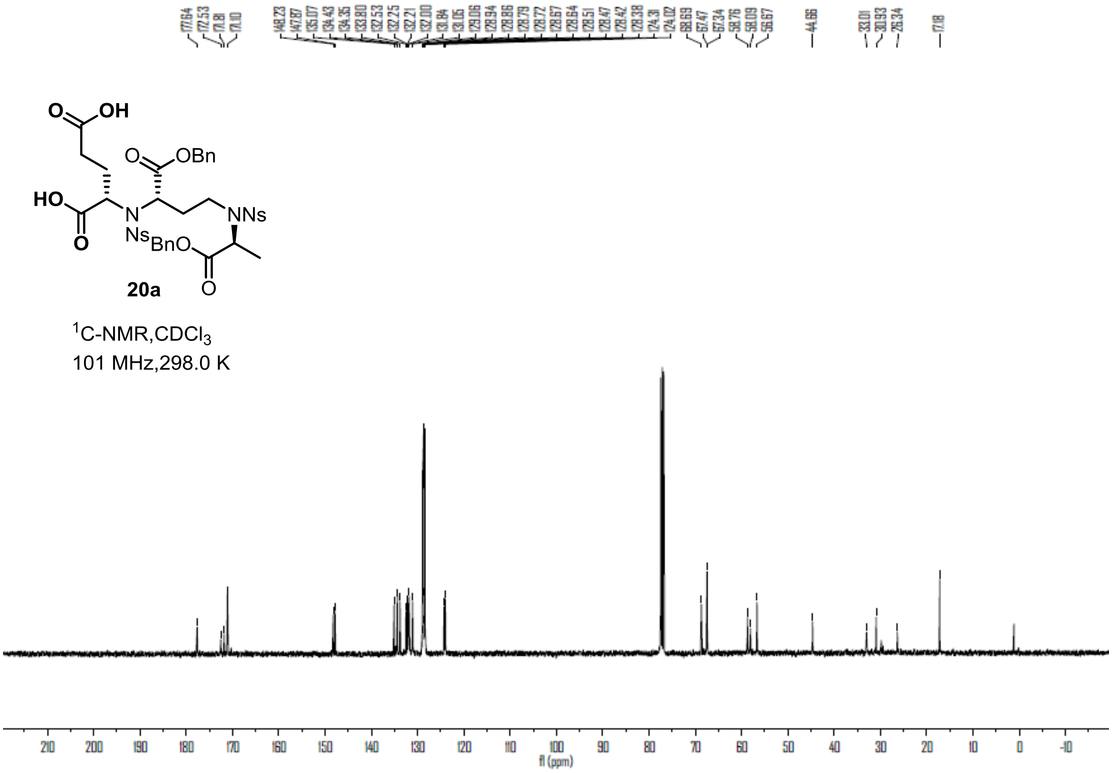

20a

^{1}C-NMR,CDCl$_3$
101 MHz,298.0 K

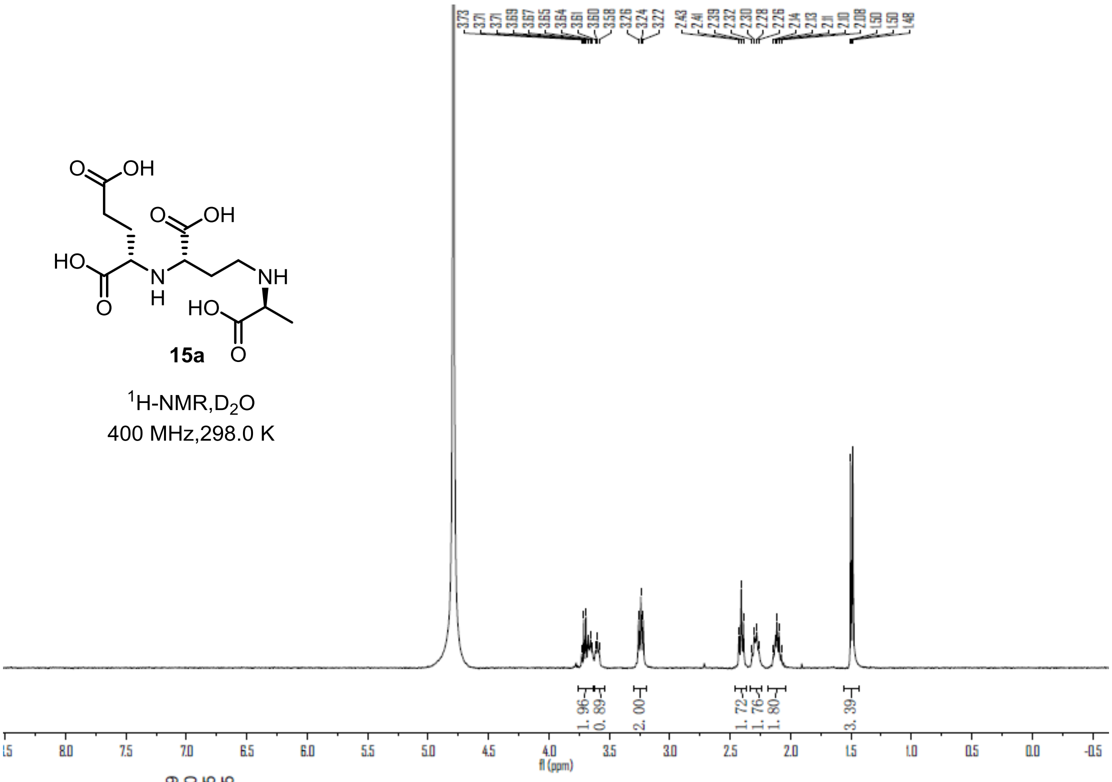

15a
^{1}H-NMR,D_2O
400 MHz,298.0 K
1.96
0.89
2.00
1.72
1.76
1.80
3.39
fl (ppm)

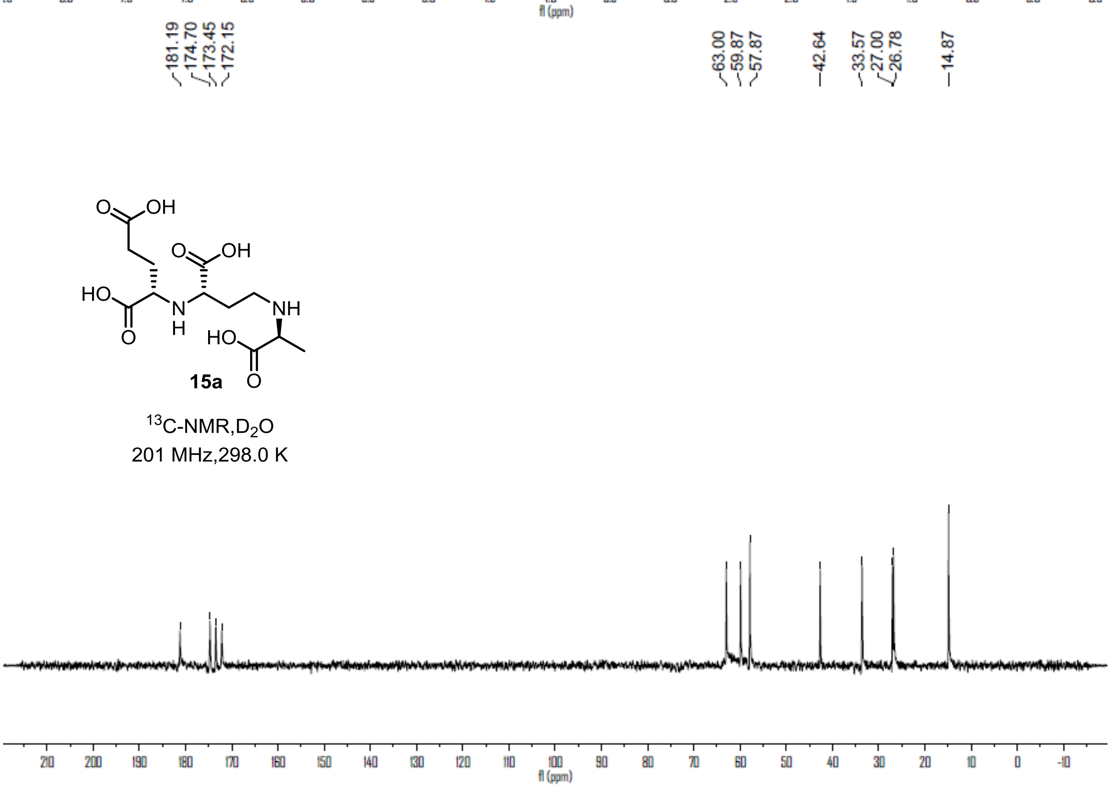

181.19
174.70
173.45
172.15
63.00
59.87
57.87
42.64
33.57
27.00
26.78
14.87
15a
^{13}C-NMR,D_2O
201 MHz,298.0 K
fl (ppm)

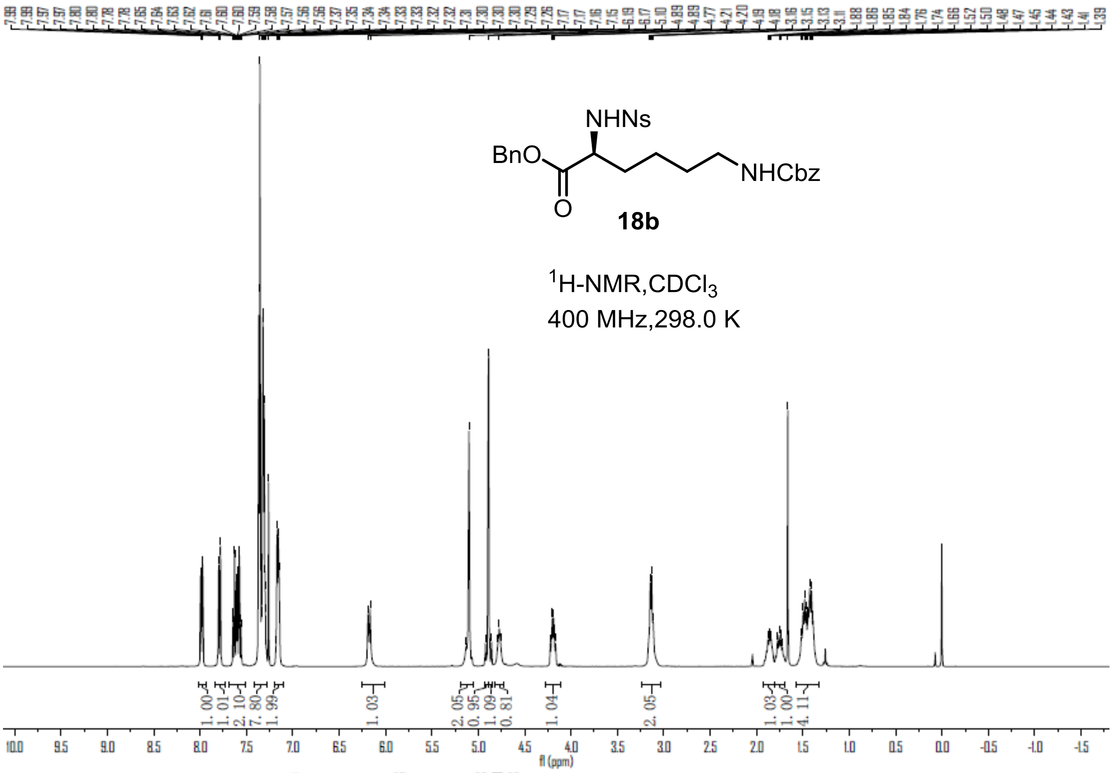
NHNs
BnO
NHCbz
O
18b
1H-NMR,CDCl3
400 MHz,298.0 K
f1 (ppm)

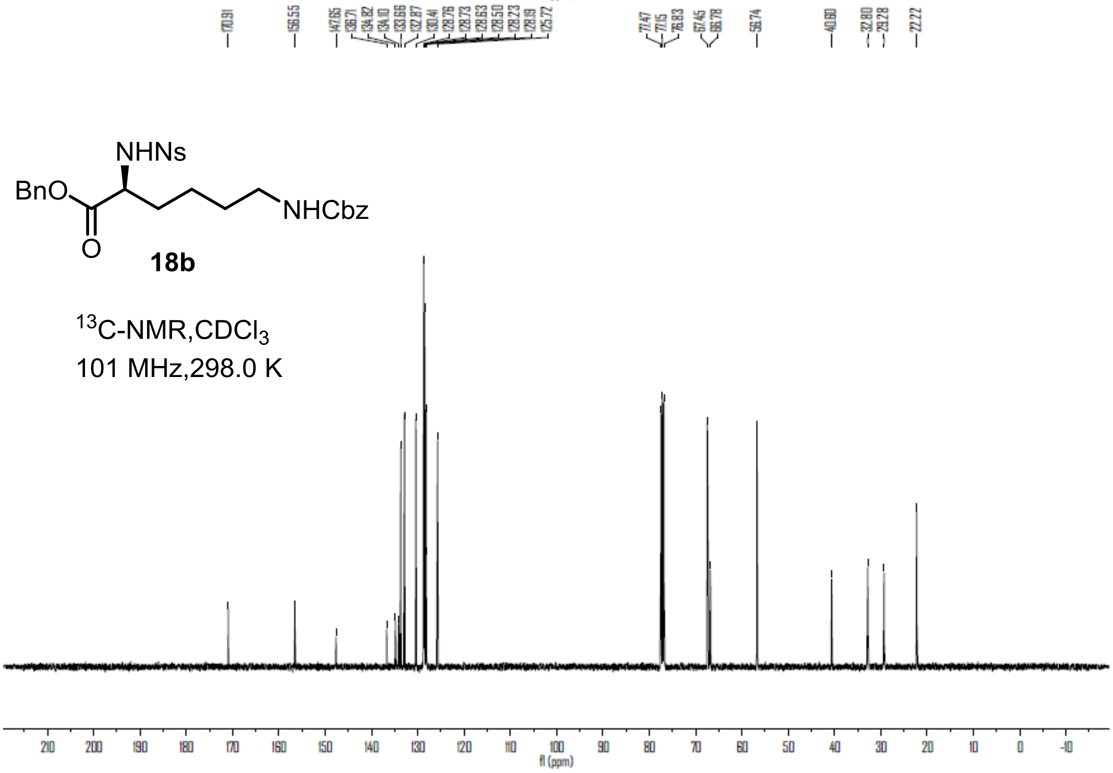
NHNs
BnO
NHCbz
O
18b
13C-NMR,CDCl3
101 MHz,298.0 K
f1 (ppm)

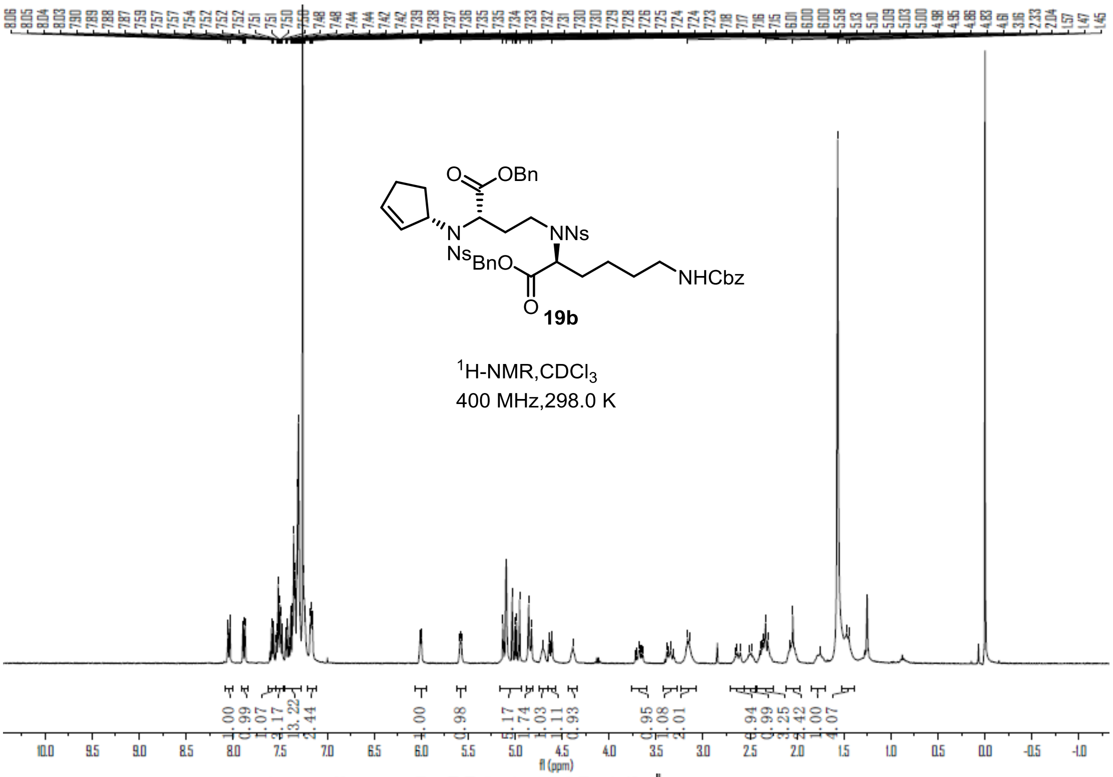
OBn
NNs
Ns
BnO
NHCbz
19b
1H-NMR,CDCl3
400 MHz,298.0 K
f1 (ppm)

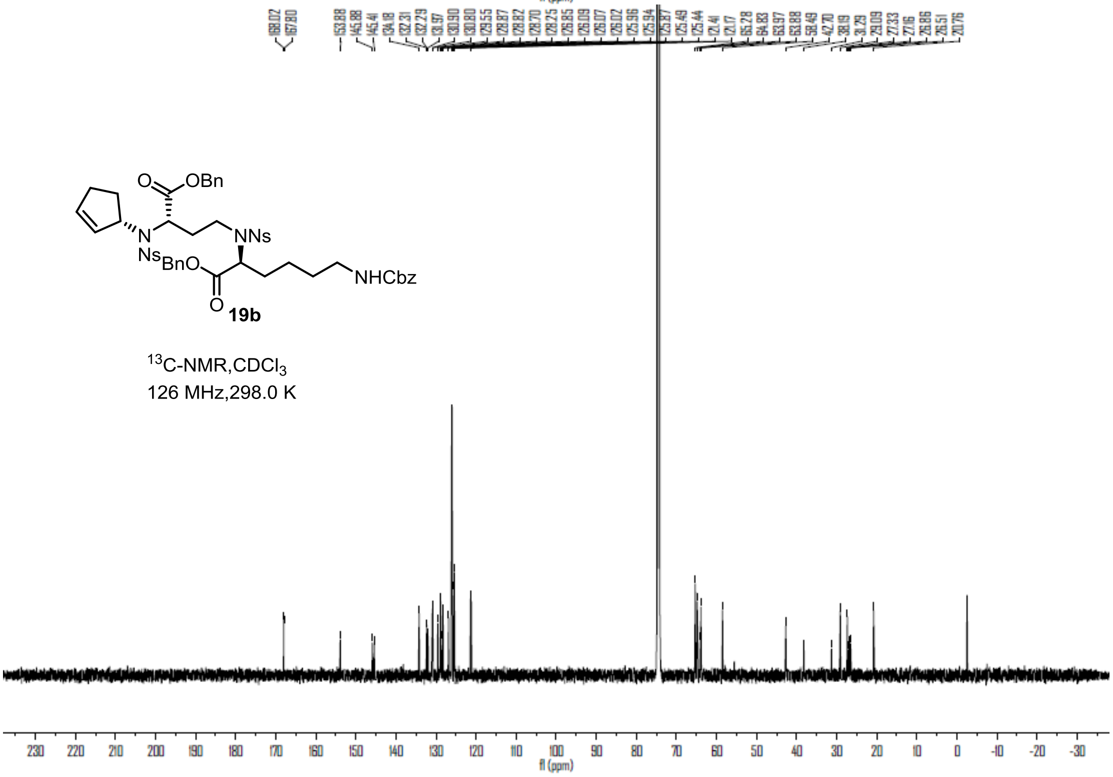
OBn
NNs
Ns
BnO
NHCbz
19b
13C-NMR,CDCl3
126 MHz,298.0 K
f1 (ppm)

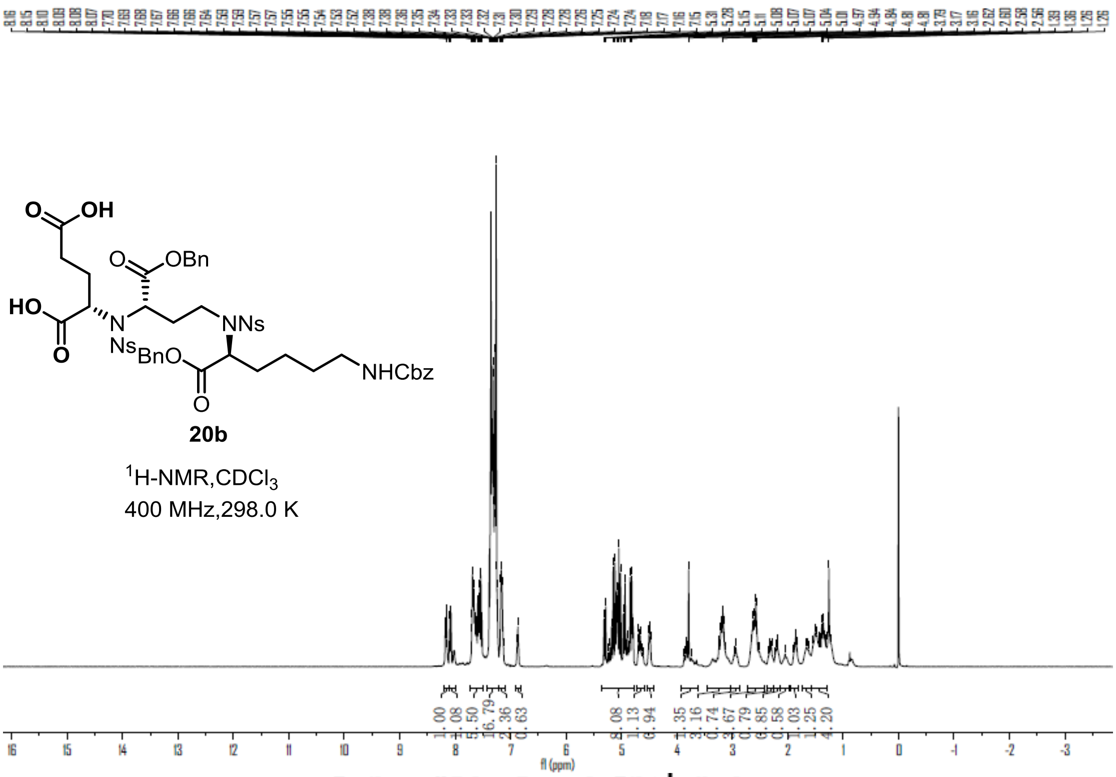
20b
^{1}H-NMR,CDCl$_3$
400 MHz,298.0 K
f1 (ppm)

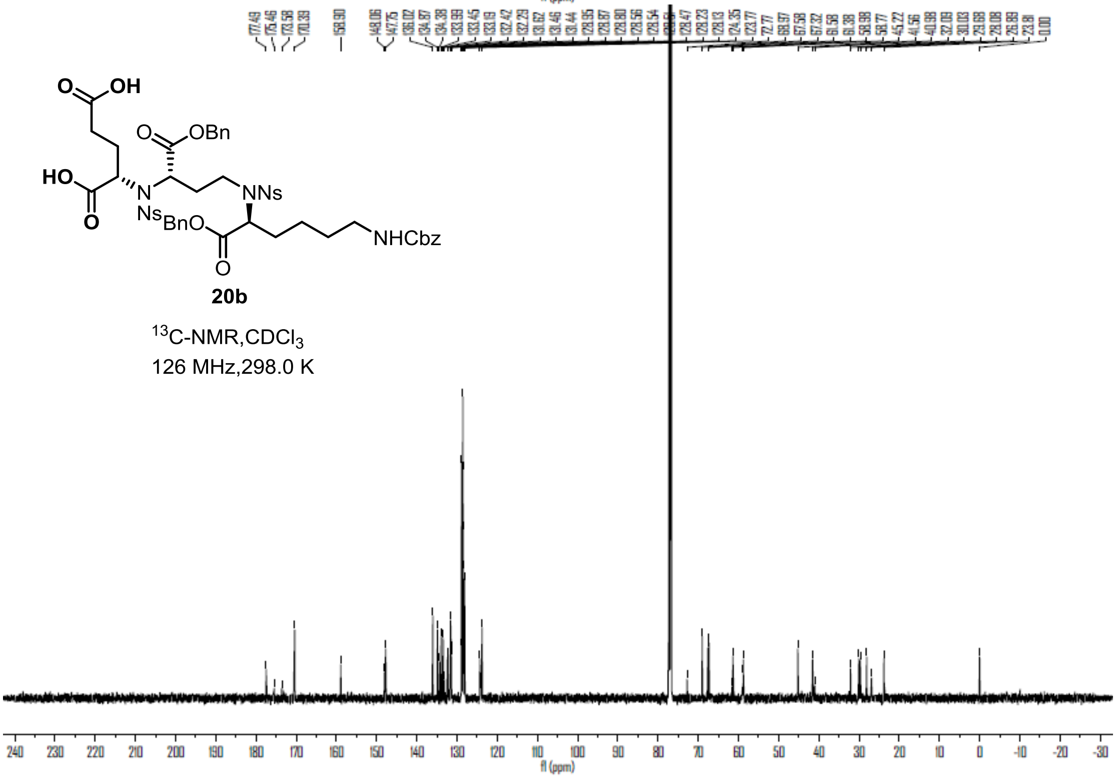
20b
^{13}C-NMR,CDCl$_3$
126 MHz,298.0 K
f1 (ppm)

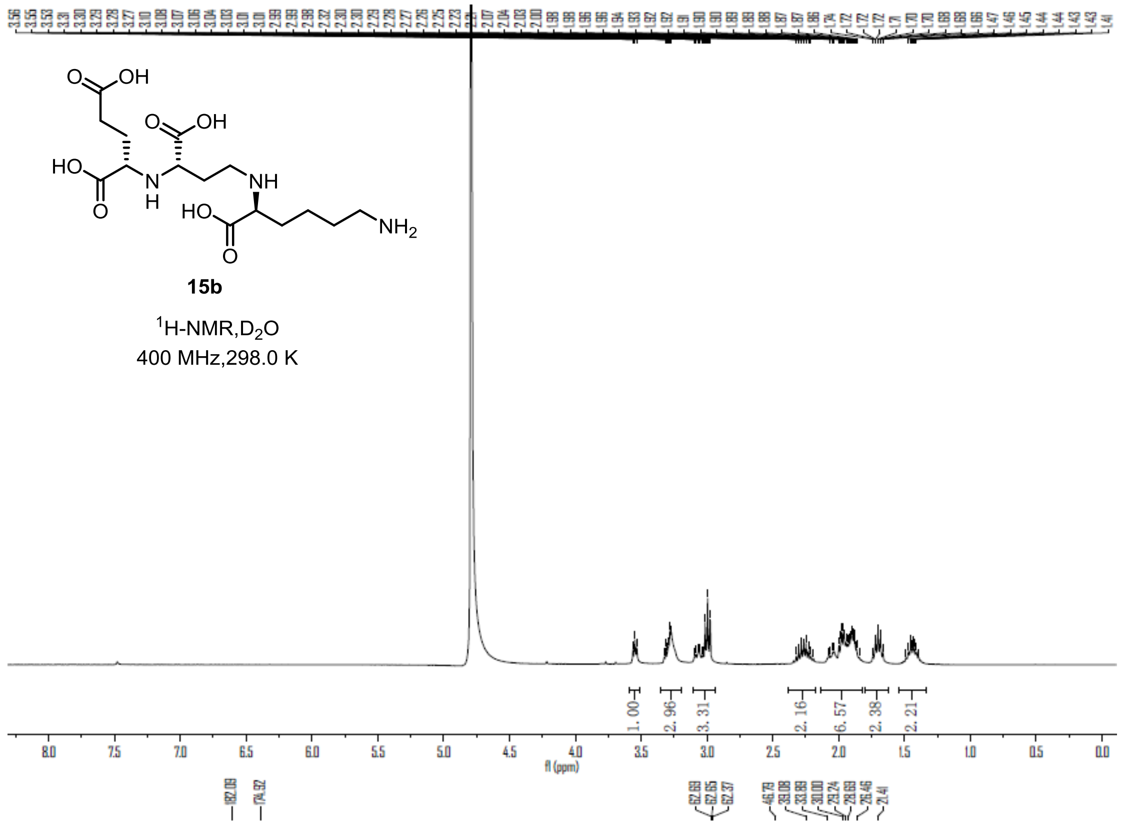
15b
^{1}H-NMR,D_2O
400 MHz,298.0 K
f1 (ppm)

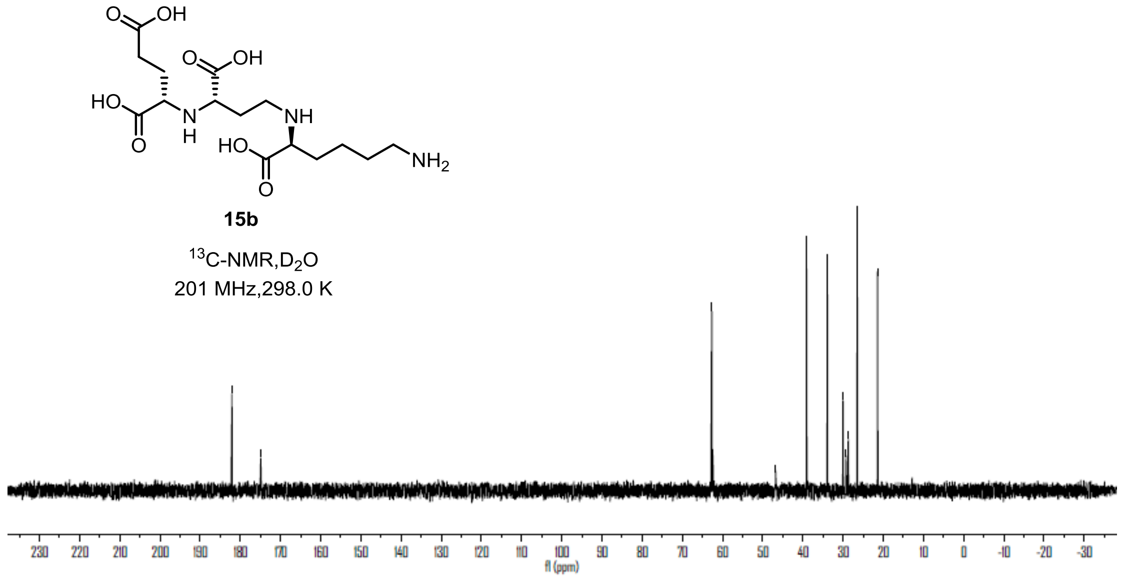
15b
^{13}C-NMR,D_2O
201 MHz,298.0 K
f1 (ppm)

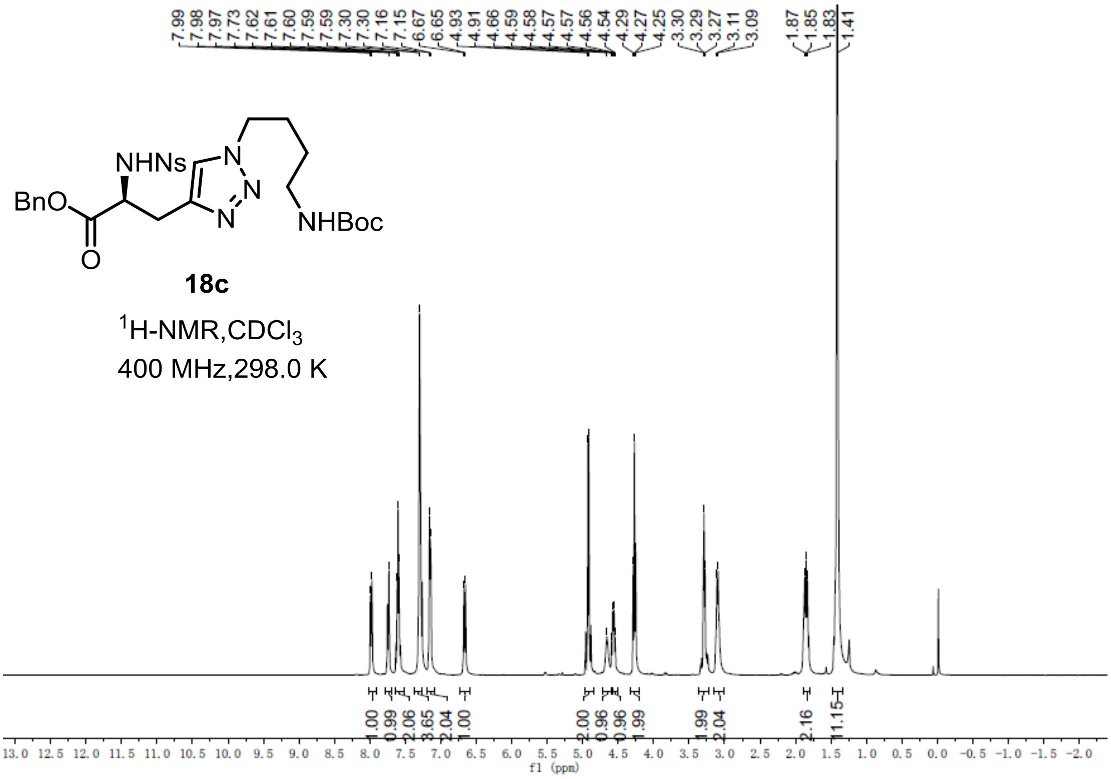
NHNs
BnO
NHBoc
18c
^{1}H-NMR,CDCl$_3$
400 MHz,298.0 K
f1 (ppm)

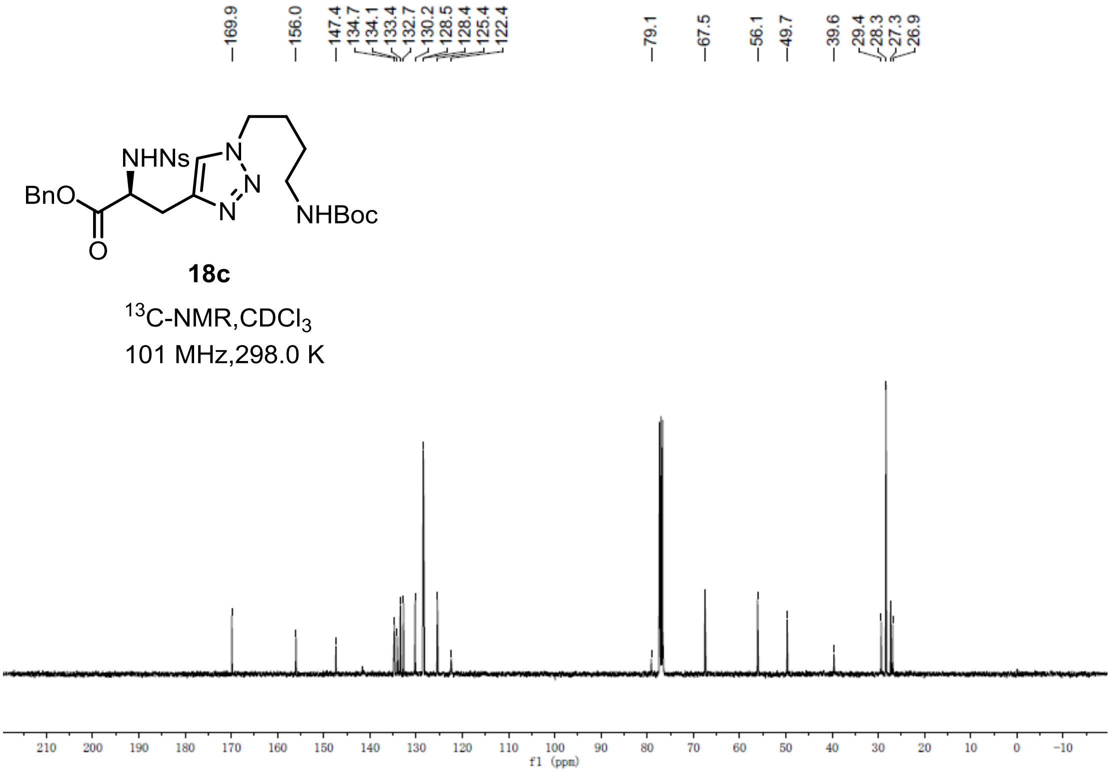
NHNs
BnO
NHBoc
18c
^{13}C-NMR,CDCl$_3$
101 MHz,298.0 K
f1 (ppm)

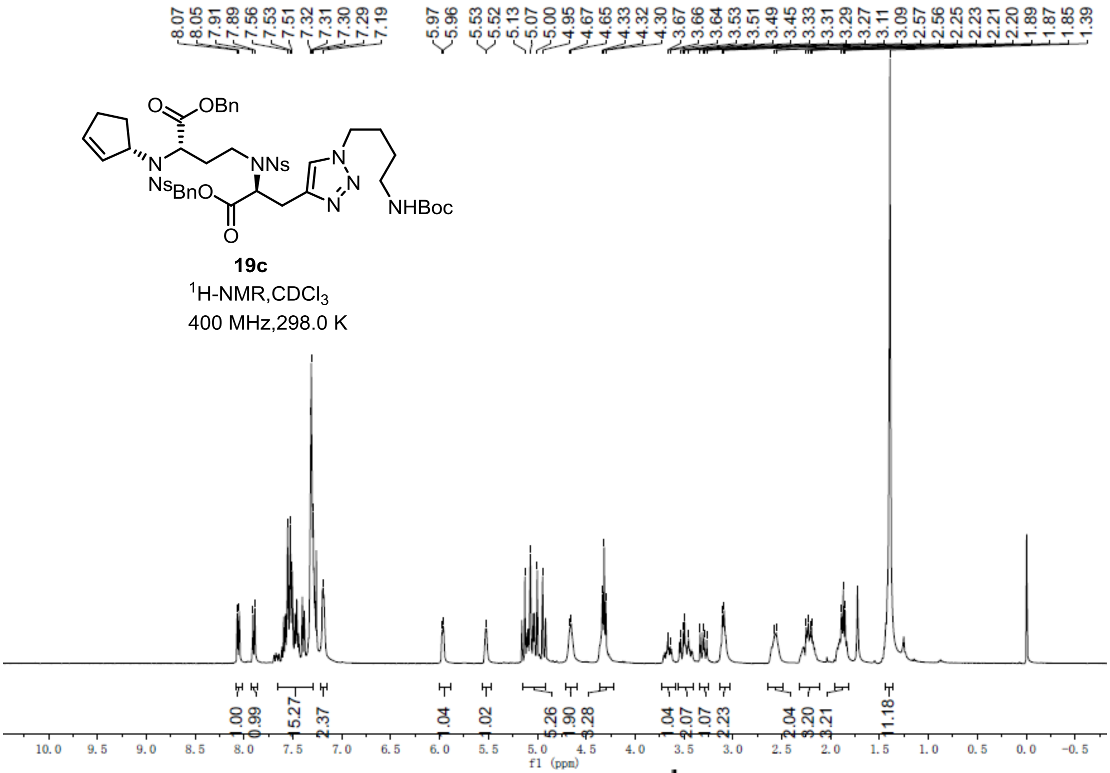
19c
^{1}H-NMR,CDCl$_3$
400 MHz,298.0 K
f1 (ppm)

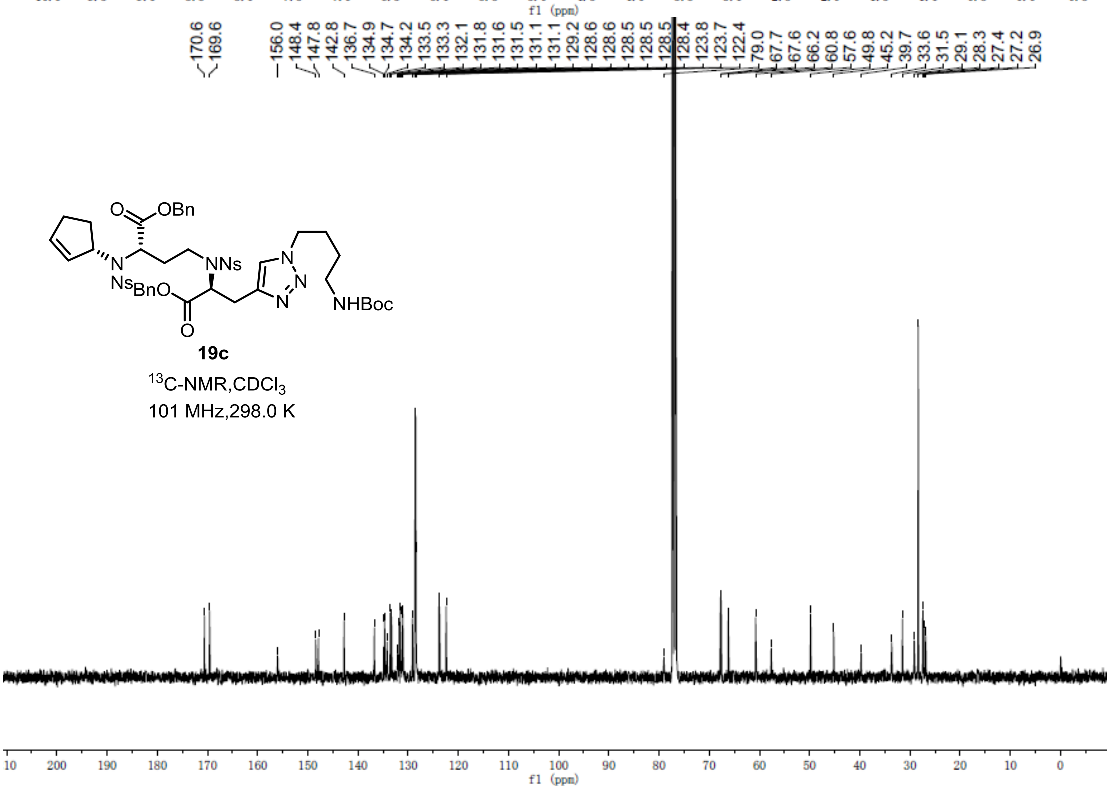
19c
^{13}C-NMR,CDCl$_3$
101 MHz,298.0 K
f1 (ppm)

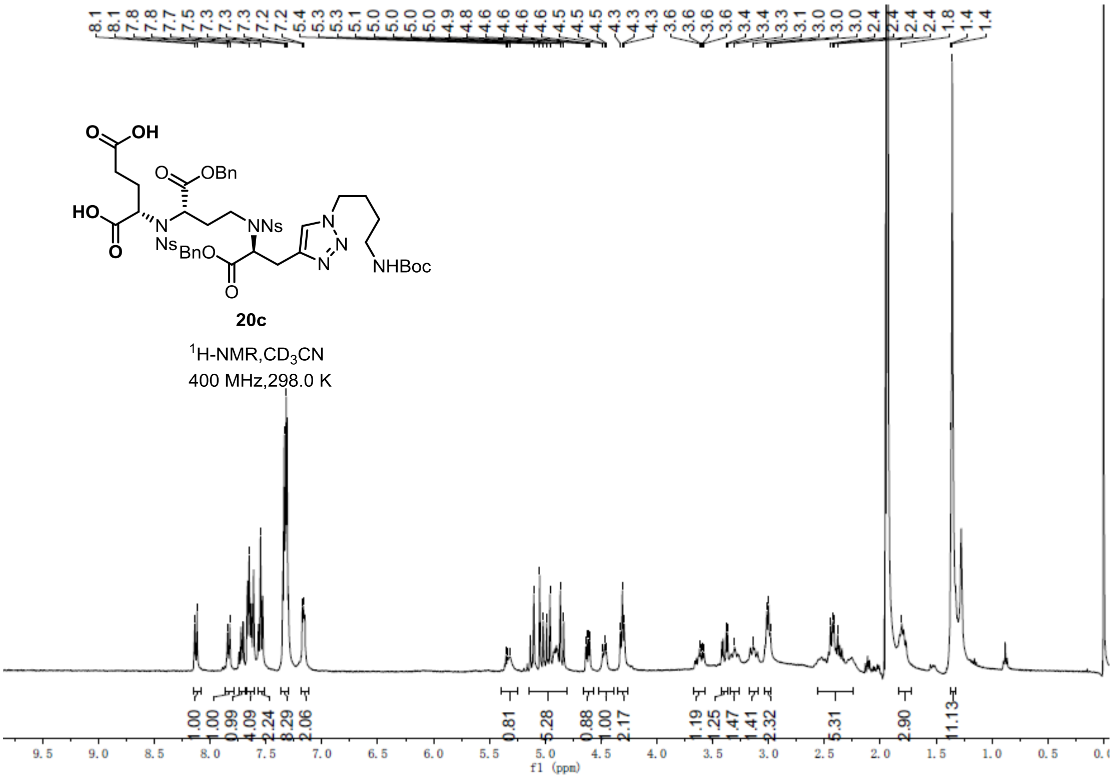
O OH
O OBn
HO
N
NNs
Ns BnO
N
N
N
NHBoc
O
O
20c
1H-NMR,CD3CN
400 MHz,298.0 K
f1 (ppm)

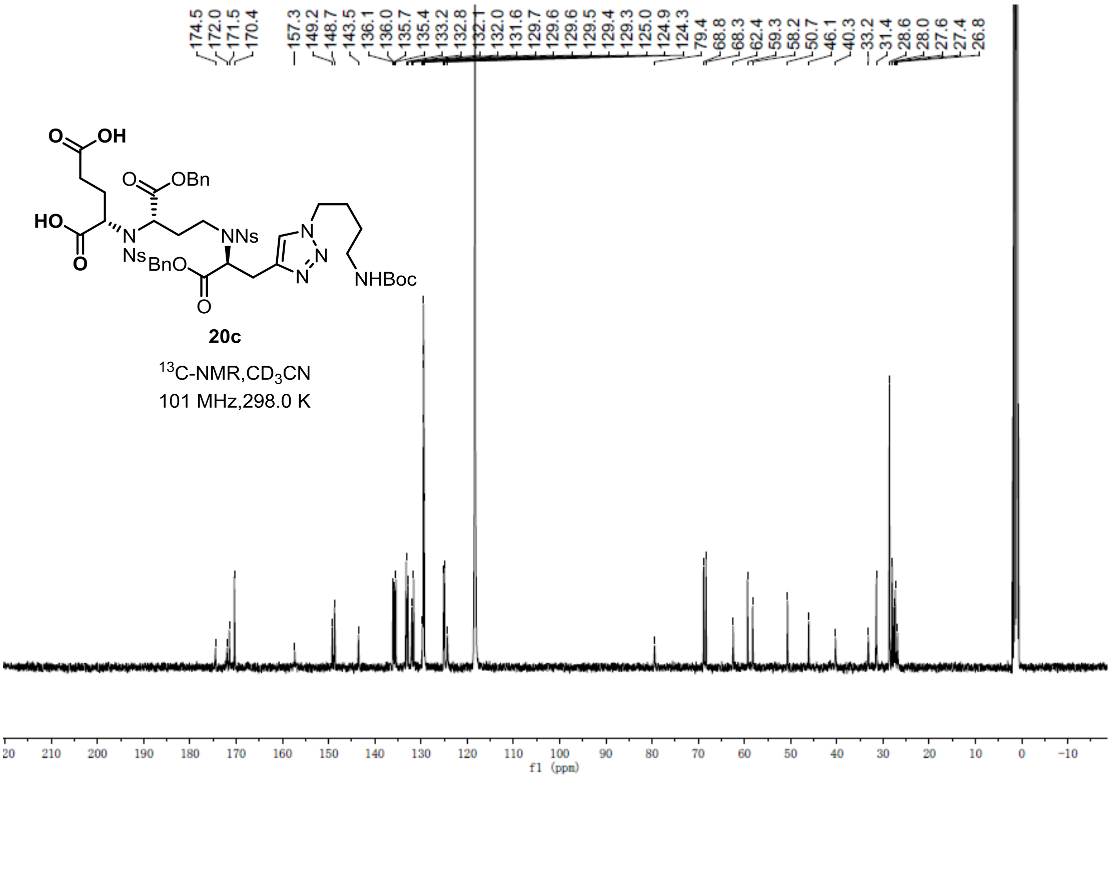
174.5
172.0
171.5
170.4
157.3
149.2
148.7
143.5
136.1
136.0
135.7
135.4
133.2
132.8
132.1
132.0
131.6
129.7
129.6
129.5
129.4
129.3
125.0
124.9
124.3
79.4
68.8
68.3
62.4
59.3
58.2
50.7
46.1
40.3
33.2
31.4
28.6
28.0
27.6
27.4
26.8
20c
13C-NMR,CD3CN
101 MHz,298.0 K
f1 (ppm)

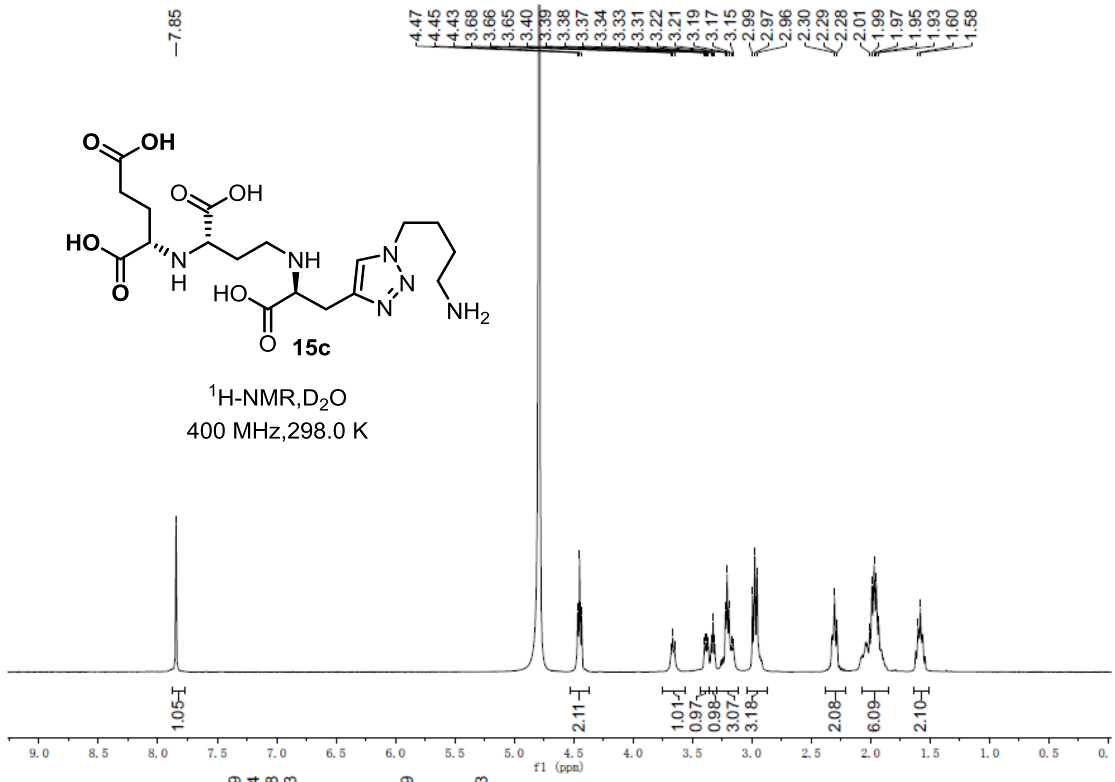
7.85
4.47 4.45 4.43 3.68 3.66 3.65 3.40 3.39 3.38 3.37 3.34 3.33 3.31 3.22 3.21 3.19 3.17 3.15 2.99 2.97 2.96 2.30 2.29 2.28 2.01 1.99 1.97 1.95 1.93 1.60 1.58
15c
^{1}H-NMR,D_2O
400 MHz,298.0 K
1.05 2.11 1.01 0.97 0.98 3.07 3.18 2.08 6.09 2.10
f1 (ppm)

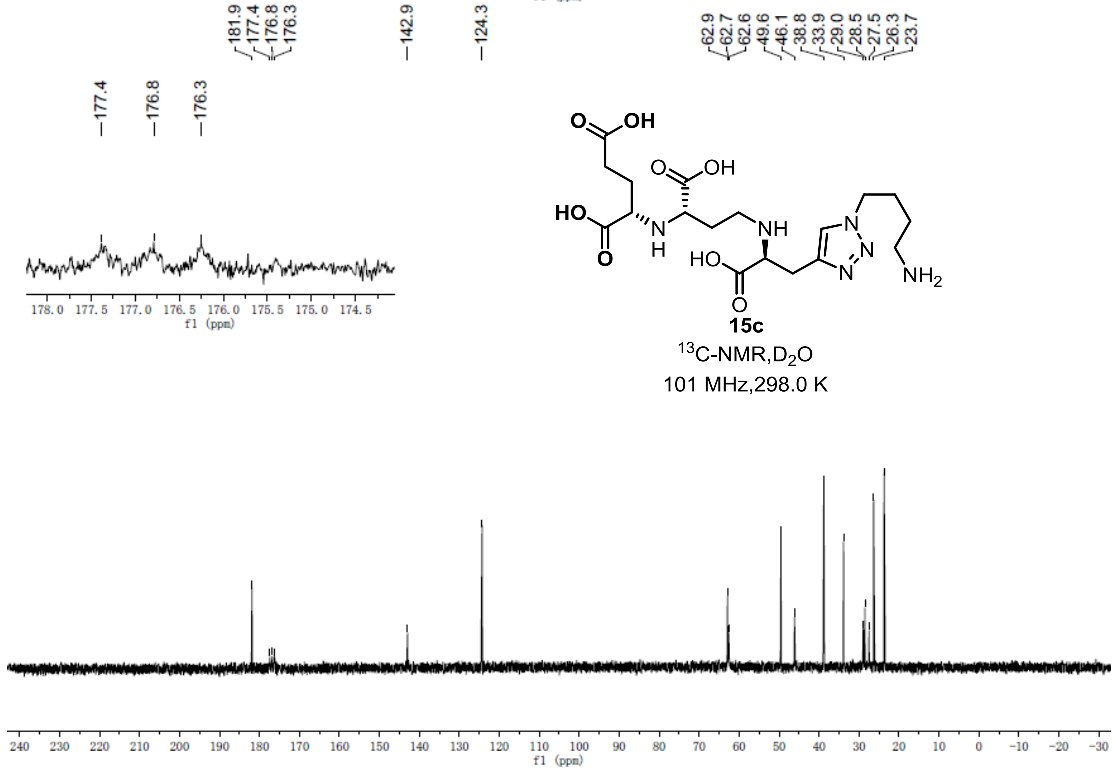
181.9 177.4 176.8 176.3
142.9
124.3
62.9 62.7 62.6 49.6 46.1 38.8 33.9 29.0 28.5 27.5 26.3 23.7
177.4
176.8
176.3
f1 (ppm)
15c
^{13}C-NMR,D_2O
101 MHz,298.0 K
f1 (ppm)

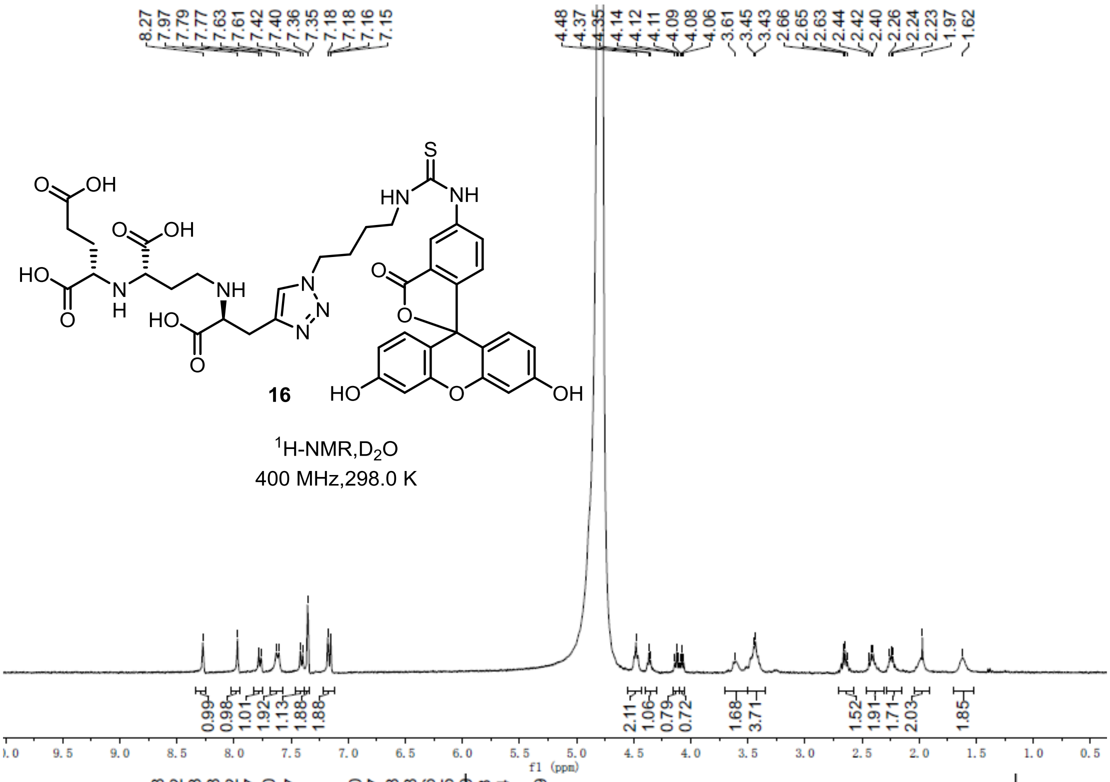
8.27 7.97 7.79 7.77 7.63 7.61 7.42 7.40 7.36 7.35 7.18 7.16 7.15
4.48 4.37 4.35 4.14 4.12 4.11 4.09 4.08 4.06 3.61 3.45 3.43 2.66 2.65 2.63 2.44 2.42 2.40 2.26 2.24 2.23 1.97 1.62
16
1H-NMR,D2O
400 MHz,298.0 K
0.99 0.98 1.01 1.92 1.13 1.88 1.88 2.11 1.06 0.79 0.72 1.68 3.71 1.52 1.91 1.71 2.03 1.85
f1 (ppm)

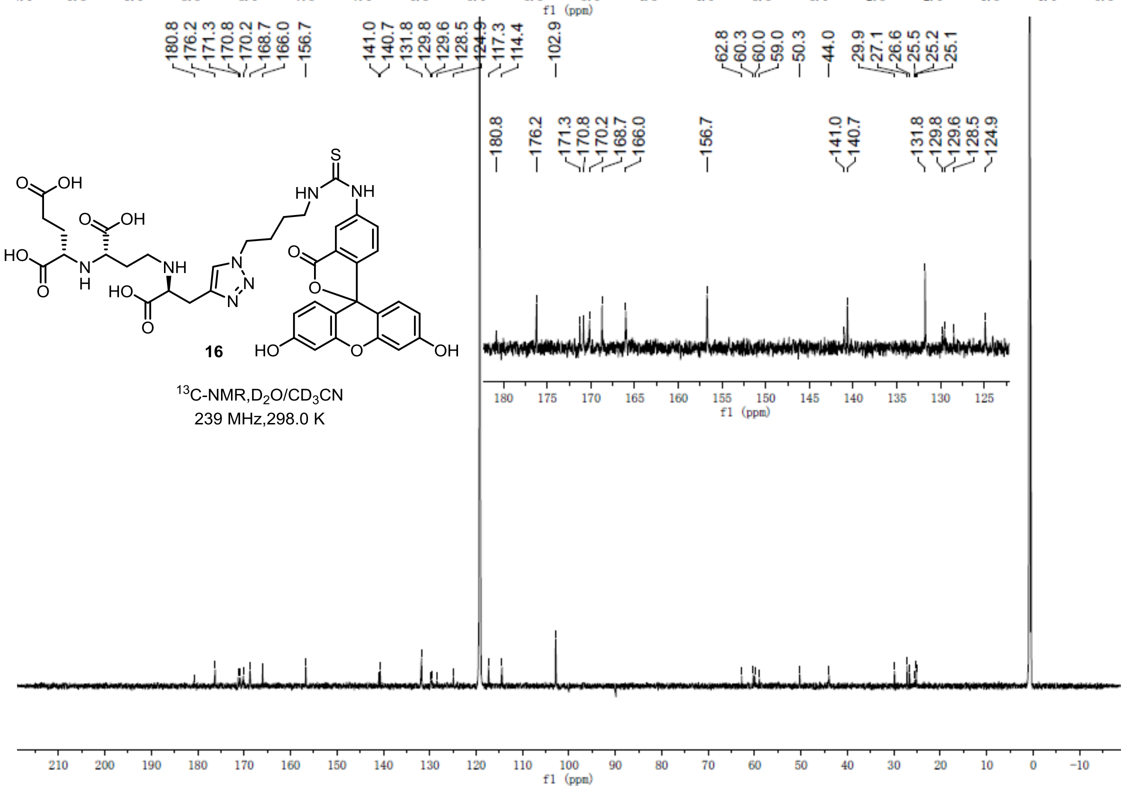
180.8 176.2 171.3 170.8 170.2 168.7 166.0 156.7 141.0 140.7 131.8 129.8 129.6 128.5 124.9 117.3 114.4 102.9 62.8 60.3 60.0 59.0 50.3 44.0 29.9 27.1 26.6 25.5 25.2 25.1
16
13C-NMR,D2O/CD3CN
239 MHz,298.0 K
f1 (ppm)

VII) HRMS Data for Chelation Experiments

1) Pseudopaline-Metal complexes and Staphylophine complexes

$[M+H]^+$ of [M-Sta] was calculated as $C_{13}H_{19}N_4O_6M$
$[M+H]^+$ of [M-Pseu] was calculated as $C_{15}H_{21}N_4O_8M$

1.1 Pseudopaline-Cu Complexes and Staphylophine-Cu Complexes

Peking University Mass Spectrometry Sample Analysis Report

Analysis Info

Analysis Name	FTMS-18080006_Pos_20180809_000003.d	Acquisition Date	8/9/2018 4:19:31 PM
Sample	wss-0495-Cu	Instrument	Bruker Solarix XR FTMS
Comment		Operator	Peking University

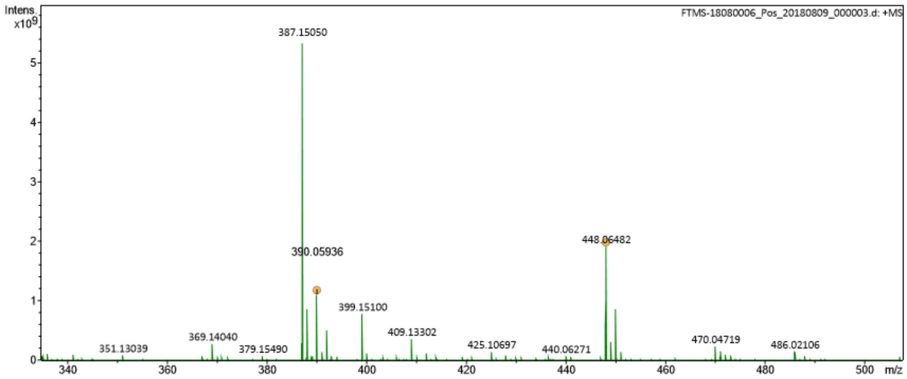

Meas. m/z	#	Ion Formula	Score	m/z	err [ppm]	rdb	e⁻ Conf	N-Rule	I
390.059360	1	C13H19CuN4O6	100.00	390.059508	0.4	6.5	odd	ok	1110598144
448.064816	1	C15H21CuN4O8	100.00	448.064988	0.4	7.5	odd	ok	1915392128

Peking University Mass Spectrometry Sample Analysis Report

Analysis Info

Analysis Name	FTMS-18080006_Pos_20180809_000004.d	Acquisition Date	8/9/2018 4:19:43 PM
Sample	wss-0495-Cu	Instrument	Bruker Solarix XR FTMS
Comment		Operator	Peking University

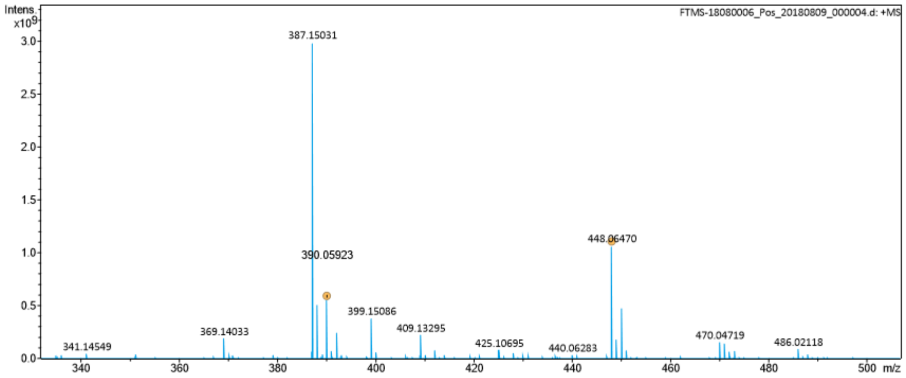

Meas. m/z	#	Ion Formula	Score	m/z	err [ppm]	rdb	e⁻ Conf	N-Rule	I
390.059230	1	C13H19CuN4O6	100.00	390.059508	0.7	6.5	odd	ok	556110336
448.064699	1	C15H21CuN4O8	100.00	448.064988	0.6	7.5	odd	ok	1071163008

1.2 Pseudopaline-Ni Complexes and Staphylophine-Ni Complexes

Peking University Mass Spectrometry Sample Analysis Report

Analysis Info

Analysis Name	FTMS-18080009_Pos_20180809_000002.d	Acquisition Date	8/9/2018 4:43:11 PM
Sample	wss-0495-Ni	Instrument	Bruker Solarix XR FTMS
Comment		Operator	Peking University

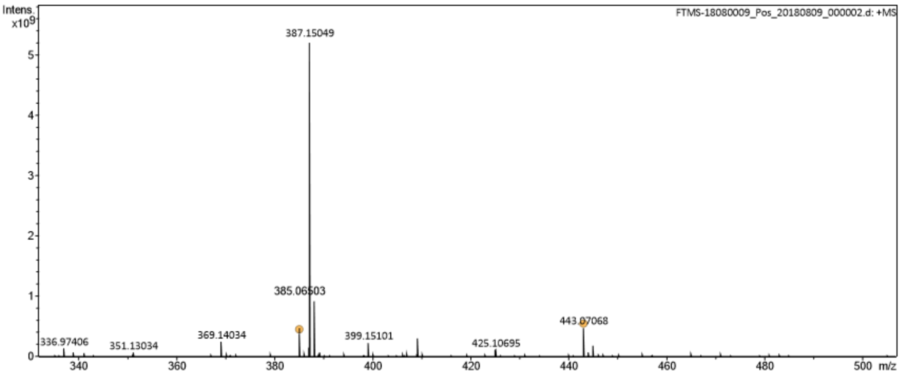

Meas. m/z	#	Ion Formula	Score	m/z	err [ppm]	rdb	e⁻ Conf	N-Rule	I
385.065032	1	C13H19N4NiO6	100.00	385.065254	0.6	7.0	even	ok	388434112
443.070675	1	C15H21N4NiO8	100.00	443.070733	0.1	8.0	even	ok	482600544

Peking University Mass Spectrometry Sample Analysis Report

Analysis Info

Analysis Name	FTMS-18080009_Pos_20180809_000001.d	Acquisition Date	8/9/2018 4:43:00 PM
Sample	wss-0495-Ni	Instrument	Bruker Solarix XR FTMS
Comment		Operator	Peking University

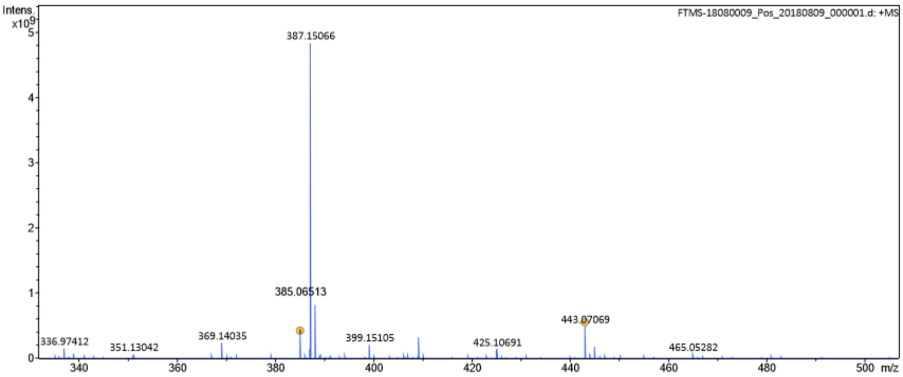

Meas. m/z	#	Ion Formula	Score	m/z	err [ppm]	rdb	e⁻ Conf	N-Rule	I
385.065129	1	C13H19N4NiO6	100.00	385.065254	0.3	7.0	even	ok	363059936
443.070691	1	C15H21N4NiO8	100.00	443.070733	0.1	8.0	even	ok	493273888

1.3 Pseudopaline-Co Complexes and Staphylophine-Co Complexes

Peking University Mass Spectrometry Sample Analysis Report

Analysis Info

Analysis Name	FTMS-18080008_Pos_20180809_000002.d	Acquisition Date	8/9/2018 4:37:22 PM
Sample	wss-0495-Co	Instrument	Bruker Solarix XR FTMS
Comment		Operator	Peking University

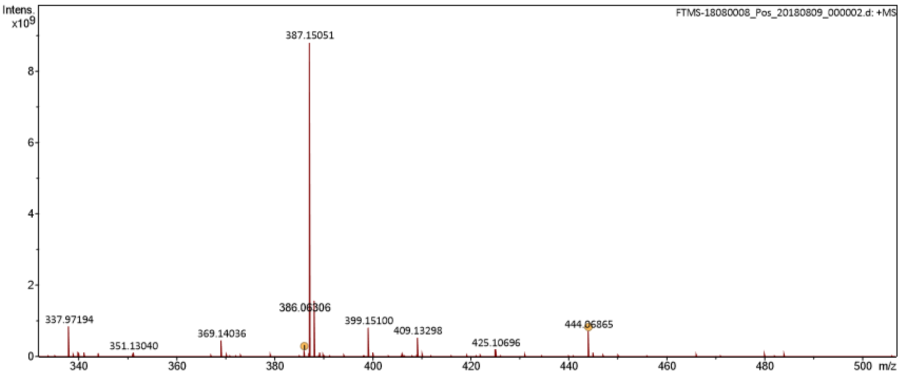

Meas. m/z	#	Ion Formula	Score	m/z	err [ppm]	rdb	e⁻ Conf	N-Rule	I
386.063058	1	C13H19CoN4O6	100.00	386.063106	0.1	7.0	even	-	179817424
444.068649	1	C15H21CoN4O8	100.00	444.068585	-0.1	8.0	even	-	721422208

Peking University Mass Spectrometry Sample Analysis Report

Analysis Info

Analysis Name	FTMS-18080008_Pos_20180809_000001.d	Acquisition Date	8/9/2018 4:37:09 PM
Sample	wss-0495-Co	Instrument	Bruker Solarix XR FTMS
Comment		Operator	Peking University

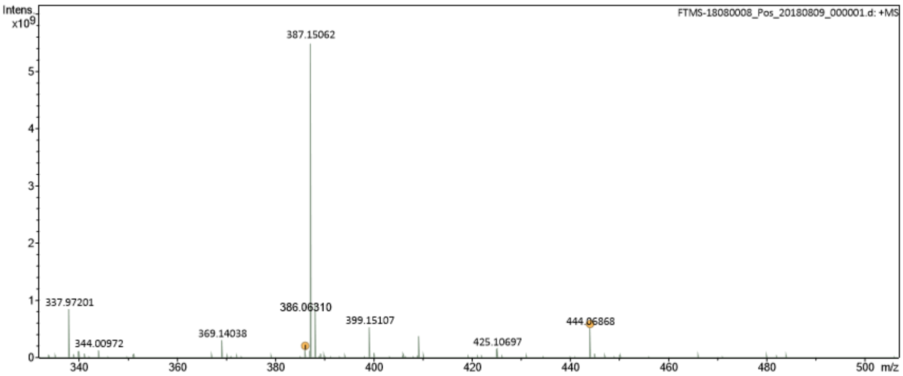

Meas. m/z	#	Ion Formula	Score	m/z	err [ppm]	rdb	e⁻ Conf	N-Rule	I
386.063096	1	C13H19CoN4O6	100.00	386.063106	0.0	7.0	even	-	138679552
444.068682	1	C15H21CoN4O8	100.00	444.068585	-0.2	8.0	even	-	523823584

1.4 Pseudopaline-Zn Complexes and Staphylophine-Zn Complexes

Peking University Mass Spectrometry Sample Analysis Report

Analysis Info

Analysis Name	FTMS-18080007_Pos_20180809_000002.d	Acquisition Date	8/9/2018 4:31:38 PM
Sample	wss-0495-Zn	Instrument	Bruker Solarix XR FTMS
Comment		Operator	Peking University

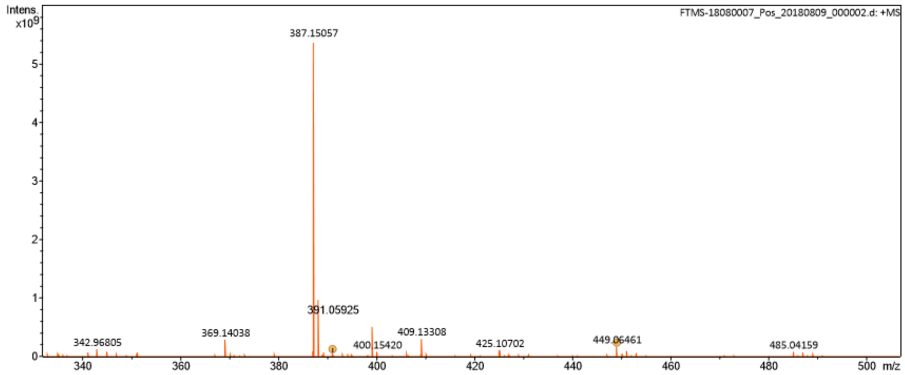

Meas. m/z	#	Ion Formula	Score	m/z	err [ppm]	rdb	e⁻ Conf	N-Rule	I
391.059253	1	C13H19N4O6Zn	100.00	391.059053	-0.5	7.0	even	ok	58516136
449.064608	1	C15H21N4O8Zn	100.00	449.064532	-0.2	8.0	even	ok	175408096

Peking University Mass Spectrometry Sample Analysis Report

Analysis Info

Analysis Name	FTMS-18080007_Pos_20180809_000001.d	Acquisition Date	8/9/2018 4:31:27 PM
Sample	wss-0495-Zn	Instrument	Bruker Solarix XR FTMS
Comment		Operator	Peking University

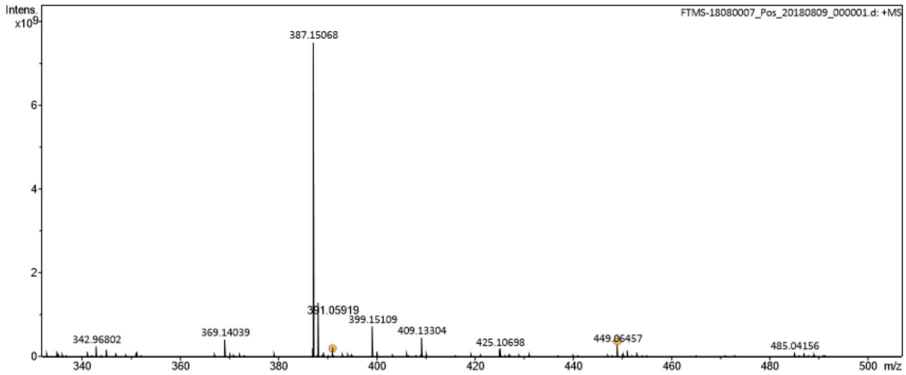

Meas. m/z	#	Ion Formula	Score	m/z	err [ppm]	rdb	e⁻ Conf	N-Rule	I
391.059193	1	C13H19N4O6Zn	100.00	391.059053	-0.4	7.0	even	ok	99059112
449.064573	1	C15H21N4O8Zn	100.00	449.064532	-0.1	8.0	even	ok	276485280

1.5 Pseudopaline-Fe Complexes and Staphylophine-Fe Complexes

Peking University Mass Spectrometry Sample Analysis Report

Analysis Info

Analysis Name	FTMS-18080087_Pos_20180820_000002.d	Acquisition Date	8/20/2018 2:21:44 PM
Sample	wss-0496-SP-Fe	Instrument	Bruker Solarix XR FTMS
Comment		Operator	Peking University

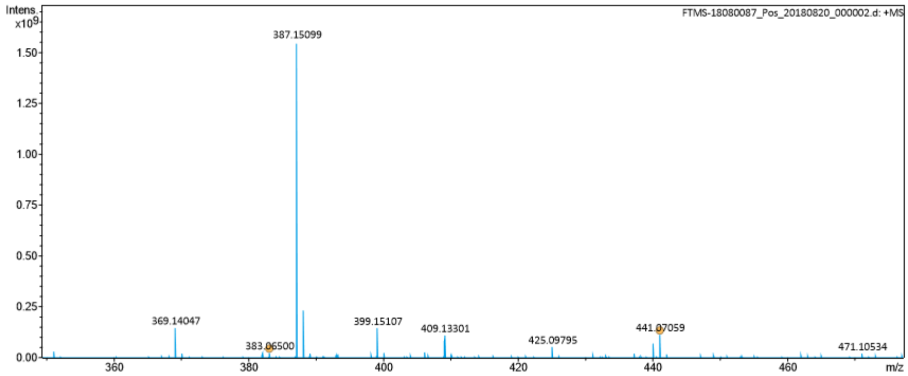

Meas. m/z	#	Ion Formula	Score	m/z	err [ppm]	rdb	e⁻ Conf	N-Rule	I
383.065003	1	C13H19FeN4O6	100.00	383.064848	-0.4	7.0	even	ok	26370426
441.070591	1	C15H21FeN4O8	100.00	441.070328	-0.6	8.0	even	ok	114791312

Peking University Mass Spectrometry Sample Analysis Report

Analysis Info

Analysis Name	FTMS-18080087_Pos_20180820_000001.d	Acquisition Date	8/20/2018 2:21:34 PM
Sample	wss-0496-SP-Fe	Instrument	Bruker Solarix XR FTMS
Comment		Operator	Peking University

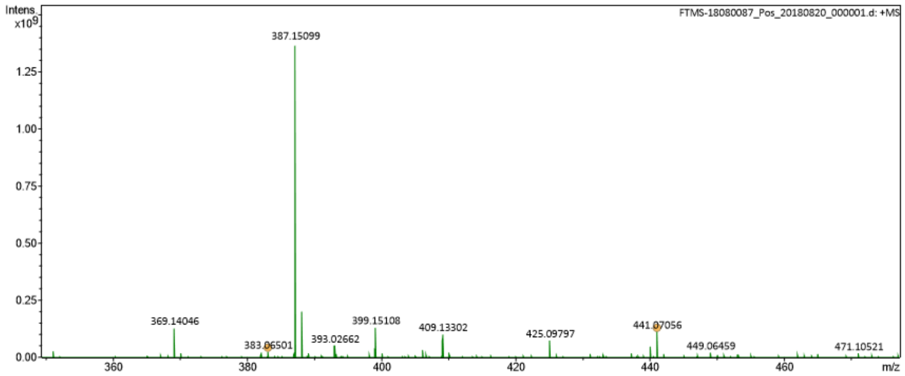

Meas. m/z	#	Ion Formula	Score	m/z	err [ppm]	rdb	e⁻ Conf	N-Rule	I
383.065012	1	C13H19FeN4O6	100.00	383.064848	-0.4	7.0	even	ok	26169726
441.070558	1	C15H21FeN4O8	100.00	441.070328	-0.5	8.0	even	ok	112785584

1.6 Pseudopaline-Mn Complexes and Staphylophine-Mn Complexes

Peking University Mass Spectrometry Sample Analysis Report

Analysis Info

Analysis Name	FTMS-18080010_Pos_20180809_000002.d	Acquisition Date	8/9/2018 4:48:08 PM
Sample	wss-0495-Mn	Instrument	Bruker Solarix XR FTMS
Comment		Operator	Peking University

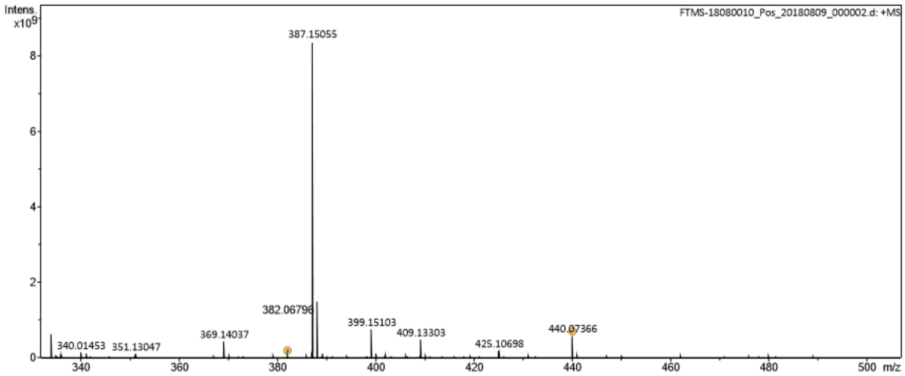

Meas. m/z	#	Ion Formula	Score	m/z	err [ppm]	rdb	e⁻ Conf	N-Rule	I
382.067960	1	C13H19MnN4O6	100.00	382.067956	-0.0	7.0	even	-	88226352
440.073660	1	C15H21MnN4O8	100.00	440.073435	-0.5	8.0	even	-	590819776

Peking University Mass Spectrometry Sample Analysis Report

Analysis Info

Analysis Name	FTMS-18080010_Pos_20180809_000001.d	Acquisition Date	8/9/2018 4:47:57 PM
Sample	wss-0495-Mn	Instrument	Bruker Solarix XR FTMS
Comment		Operator	Peking University

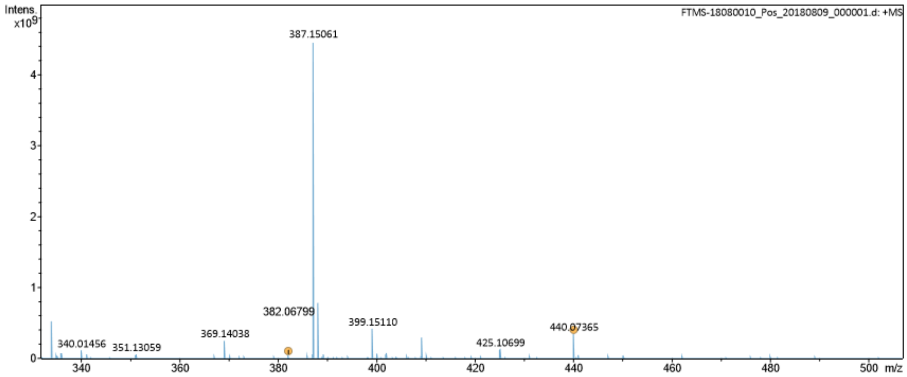

Meas. m/z	#	Ion Formula	Score	m/z	err [ppm]	rdb	e⁻ Conf	N-Rule	I
382.067988	1	C13H19MnN4O6	100.00	382.067956	-0.1	7.0	even	-	50985168
440.073647	1	C15H21MnN4O8	100.00	440.073435	-0.5	8.0	even	-	352160512

2) Pseudopaline-Metal complexes and Staphylophine complexes

$[M+H]^+$ of [M-Sta] was calculated as $C_{13}H_{19}N_4O_6M$

$[M+H]^+$ of [M-*epi*-Pseu] was calculated as $C_{15}H_{21}N_4O_8M$

2.1 *epi*-Pseudopaline-Cu Complexes and Staphylophine-Cu Complexes

Peking University Mass Spectrometry Sample Analysis Report

Analysis Info

Analysis Name	FTMS-18080066_Pos_20180820_000001.d	Acquisition Date	8/20/2018 11:07:51 AM
Sample	wss-0496-SE-Cu	Instrument	Bruker Solarix XR FTMS
Comment		Operator	Peking University

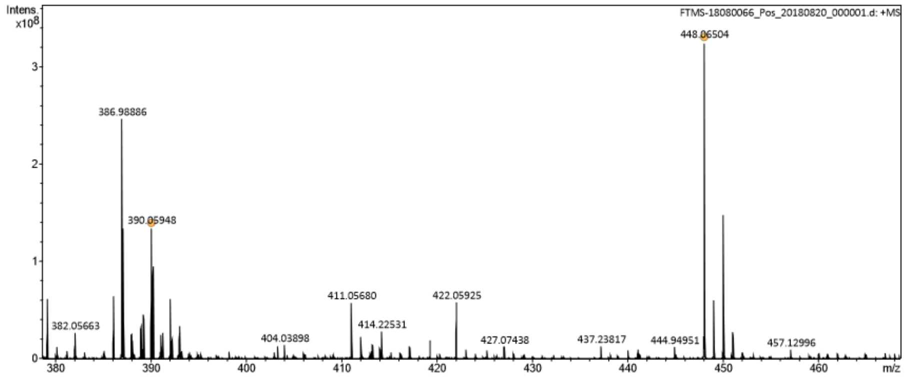

Meas. m/z	#	Ion Formula	Score	m/z	err [ppm]	rdb	e⁻ Conf	N-Rule	I
390.059482	1	C13H19CuN4O6	100.00	390.059508	0.1	6.5	odd	ok	135590480
448.065039	1	C15H21CuN4O8	100.00	448.064988	-0.1	7.5	odd	ok	326262176

Peking University Mass Spectrometry Sample Analysis Report

Analysis Info

Analysis Name	FTMS-18080066_Pos_20180820_000002.d	Acquisition Date	8/20/2018 11:08:29 AM
Sample	wss-0496-SE-Cu	Instrument	Bruker Solarix XR FTMS
Comment		Operator	Peking University

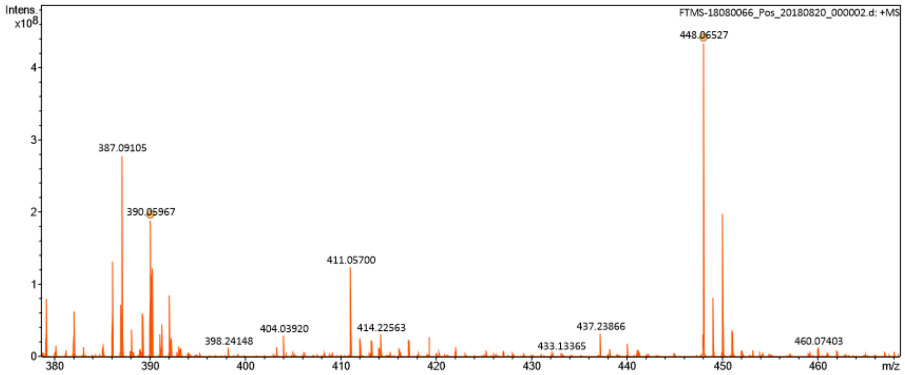

Meas. m/z	#	Ion Formula	Score	m/z	err [ppm]	rdb	e⁻ Conf	N-Rule	I
390.059673	1	C13H19CuN4O6	100.00	390.059508	-0.4	6.5	odd	ok	191046624
448.065266	1	C15H21CuN4O8	100.00	448.064988	-0.6	7.5	odd	ok	435927424

2.2 *epi*-Pseudopaline-Ni Complexes and Staphylophine-Ni Complexes

Peking University Mass Spectrometry Sample Analysis Report

Analysis Info

Analysis Name	FTMS-18080069_Pos_20180820_000002.d	Acquisition Date	8/20/2018 11:28:09 AM
Sample	wss-0496-SE-Ni	Instrument	Bruker Solarix XR FTMS
Comment		Operator	Peking University

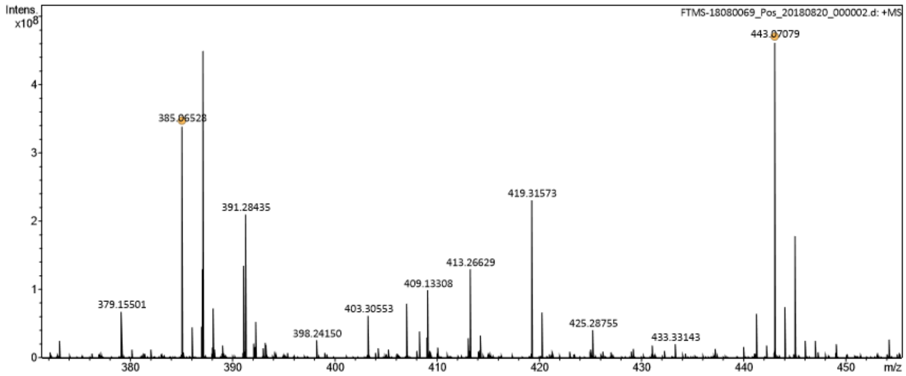

Meas. m/z	#	Ion Formula	Score	m/z	err [ppm]	rdb	e⁻ Conf	N-Rule	I
385.065284	1	C13H19N4NiO6	100.00	385.065254	-0.1	7.0	even	ok	341180128
443.070794	1	C15H21N4NiO8	100.00	443.070733	-0.1	8.0	even	ok	464360320

Peking University Mass Spectrometry Sample Analysis Report

Analysis Info

Analysis Name	FTMS-18080069_Pos_20180820_000001.d	Acquisition Date	8/20/2018 11:28:00 AM
Sample	wss-0496-SE-Ni	Instrument	Bruker Solarix XR FTMS
Comment		Operator	Peking University

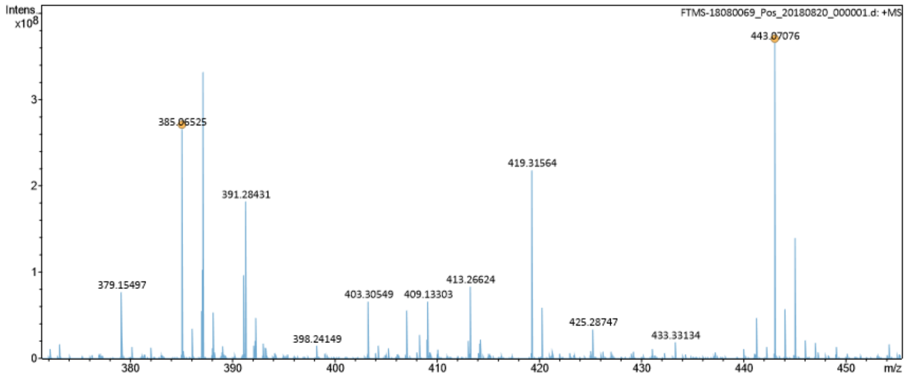

Meas. m/z	#	Ion Formula	Score	m/z	err [ppm]	rdb	e⁻ Conf	N-Rule	I
385.065250	1	C13H19N4NiO6	100.00	385.065254	0.0	7.0	even	ok	266114688
443.070758	1	C15H21N4NiO8	100.00	443.070733	-0.1	8.0	even	ok	365610880

2.3 *epi*-Pseudopaline-Zn Complexes and Staphylophine-Zn Complexes

Peking University Mass Spectrometry Sample Analysis Report

Analysis Info

Analysis Name	FTMS-18080067_Pos_20180820_000001.d	Acquisition Date	8/20/2018 11:14:14 AM
Sample	wss-0496-SE-Zn	Instrument	Bruker Solarix XR FTMS
Comment		Operator	Peking University

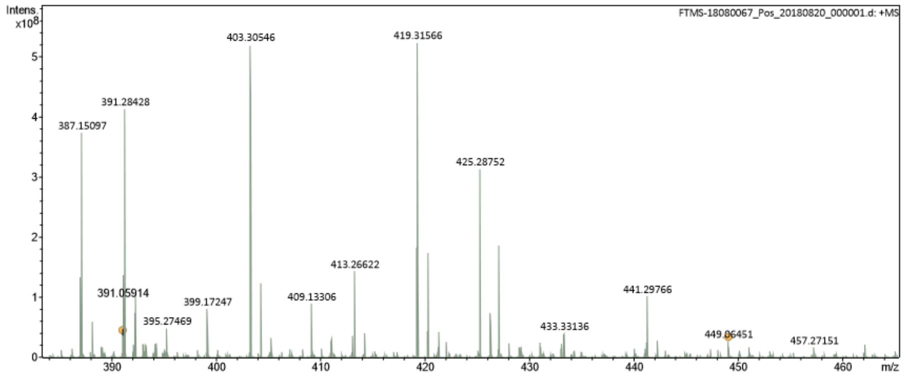

Meas. m/z	#	Ion Formula	Score	m/z	err [ppm]	rdb	e⁻ Conf	N-Rule	I
391.059137	1	C13H19N4O6Zn	100.00	391.059053	-0.2	7.0	even	ok	39112548
449.064512	1	C15H21N4O8Zn	100.00	449.064532	0.0	8.0	even	ok	27795290

Peking University Mass Spectrometry Sample Analysis Report

Analysis Info

Analysis Name	FTMS-18080067_Pos_20180820_000002.d	Acquisition Date	8/20/2018 11:14:40 AM
Sample	wss-0496-SE-Zn	Instrument	Bruker Solarix XR FTMS
Comment		Operator	Peking University

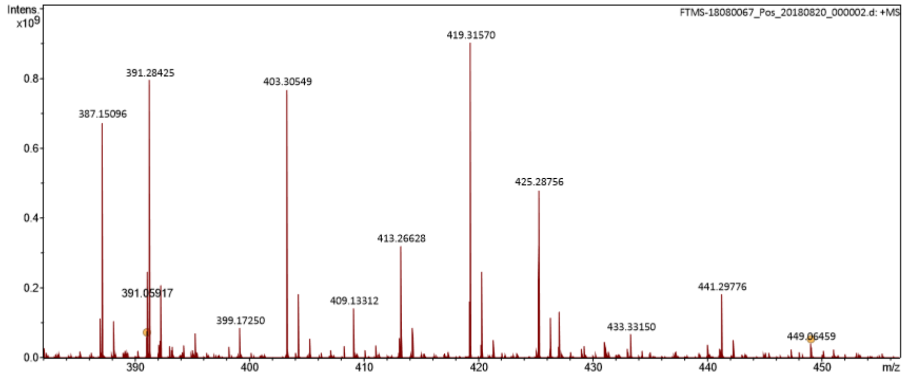

Meas. m/z	#	Ion Formula	Score	m/z	err [ppm]	rdb	e⁻ Conf	N-Rule	I
391.059169	1	C13H19N4O6Zn	100.00	391.059053	-0.3	7.0	even	ok	61467232
449.064593	1	C15H21N4O8Zn	100.00	449.064532	-0.1	8.0	even	ok	42003280

2.4 *epi*-Pseudopaline-Co Complexes and Staphylophine-Co Complexes

Peking University Mass Spectrometry Sample Analysis Report

Analysis Info

Analysis Name	FTMS-18080068_Pos_20180820_000002.d	Acquisition Date	8/20/2018 11:22:35 AM
Sample	wss-0496-SE-Co	Instrument	Bruker Solarix XR FTMS
Comment		Operator	Peking University

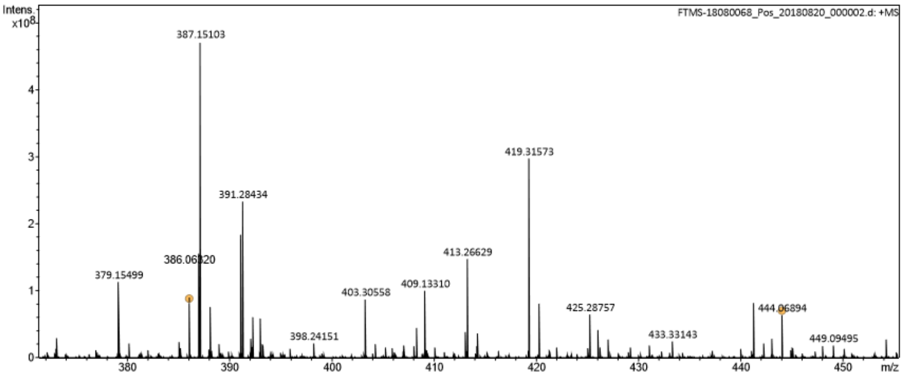

Meas. m/z	#	Ion Formula	Score	m/z	err [ppm]	rdb	e⁻ Conf	N-Rule	I
386.063195	1	C13H19CoN4O6	100.00	386.063106	-0.2	7.0	even	-	82440144
444.068936	1	C15H21CoN4O8	100.00	444.068585	-0.8	8.0	even	-	64471776

Peking University Mass Spectrometry Sample Analysis Report

Analysis Info

Analysis Name	FTMS-18080068_Pos_20180820_000001.d	Acquisition Date	8/20/2018 11:22:24 AM
Sample	wss-0496-SE-Co	Instrument	Bruker Solarix XR FTMS
Comment		Operator	Peking University

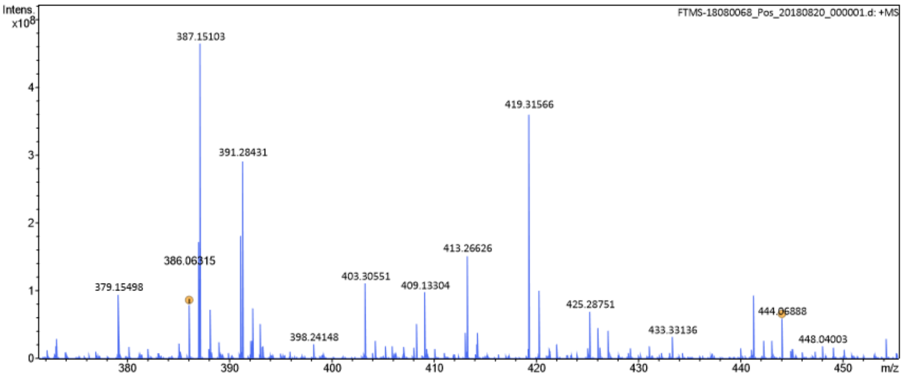

Meas. m/z	#	Ion Formula	Score	m/z	err [ppm]	rdb	e⁻ Conf	N-Rule	I
386.063146	1	C13H19CoN4O6	100.00	386.063106	-0.1	7.0	even	-	80554424
444.068881	1	C15H21CoN4O8	100.00	444.068585	-0.7	8.0	even	-	60373152

2.5 *epi*-Pseudopaline-Fe Complexes and Staphylophine-Fe Complexes

Peking University Mass Spectrometry Sample Analysis Report

Analysis Info

Analysis Name	FTMS-18080088_Pos_20180820_000002.d	Acquisition Date	8/20/2018 2:28:36 PM
Sample	wss-0496-SE-Fe	Instrument	Bruker Solarix XR FTMS
Comment		Operator	Peking University

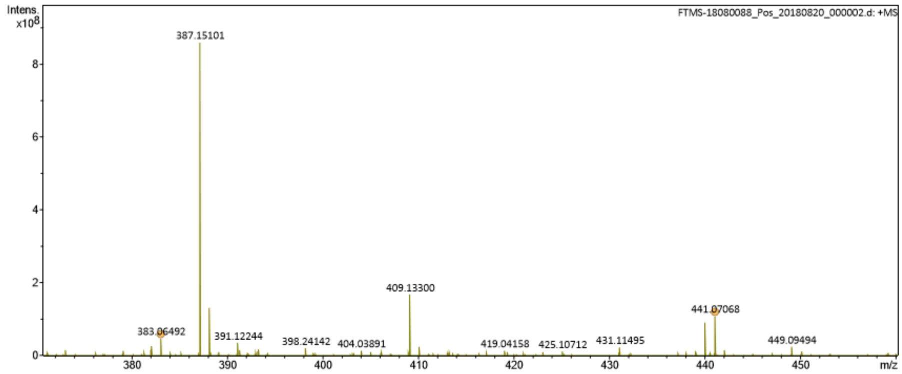

Meas. m/z	#	Ion Formula	Score	m/z	err [ppm]	rdb	e⁻ Conf	N-Rule	I
383.064923	1	C13H19FeN4O6	100.00	383.064848	-0.2	7.0	even	ok	48432136
441.070682	1	C15H21FeN4O8	100.00	441.070328	-0.8	8.0	even	ok	108706456

Peking University Mass Spectrometry Sample Analysis Report

Analysis Info

Analysis Name	FTMS-18080088_Pos_20180820_000001.d	Acquisition Date	8/20/2018 2:28:25 PM
Sample	wss-0496-SE-Fe	Instrument	Bruker Solarix XR FTMS
Comment		Operator	Peking University

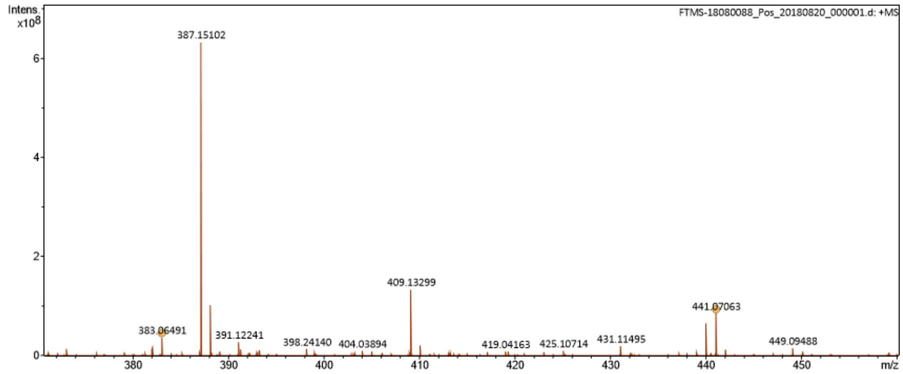

Meas. m/z	#	Ion Formula	Score	m/z	err [ppm]	rdb	e⁻ Conf	N-Rule	I
383.064907	1	C13H19FeN4O6	100.00	383.064848	-0.2	7.0	even	ok	37494300
441.070632	1	C15H21FeN4O8	100.00	441.070328	-0.7	8.0	even	ok	86002280

2.6 *epi*-Pseudopaline-Mn Complexes and Staphylophine-Mn Complexes

Peking University Mass Spectrometry Sample Analysis Report

Analysis Info

Analysis Name	FTMS-18080070_Pos_20180820_000002.d	Acquisition Date	8/20/2018 11:34:12 AM
Sample	wss-0496-SE-Mn	Instrument	Bruker Solarix XR FTMS
Comment		Operator	Peking University

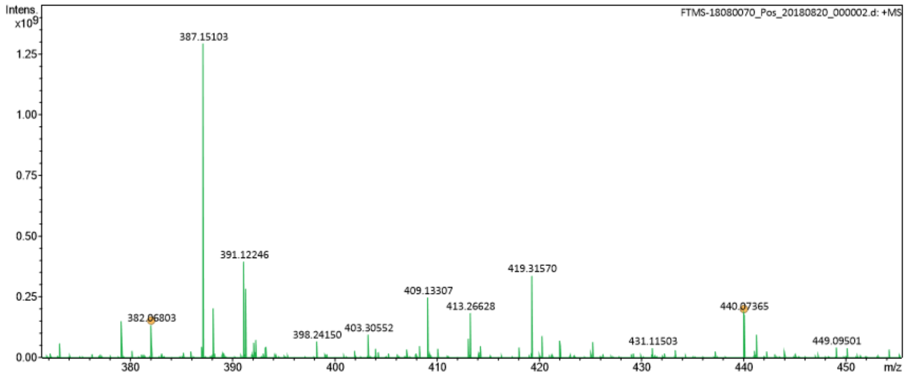

Meas. m/z	#	Ion Formula	Score	m/z	err [ppm]	rdb	e⁻ Conf	N-Rule	I
382.068028	1	C13H19MnN4O6	100.00	382.067956	-0.2	7.0	even	-	136608752
440.073650	1	C15H21MnN4O8	100.00	440.073435	-0.5	8.0	even	-	184520368

Peking University Mass Spectrometry Sample Analysis Report

Analysis Info

Analysis Name	FTMS-18080070_Pos_20180820_000001.d	Acquisition Date	8/20/2018 11:33:58 AM
Sample	wss-0496-SE-Mn	Instrument	Bruker Solarix XR FTMS
Comment		Operator	Peking University

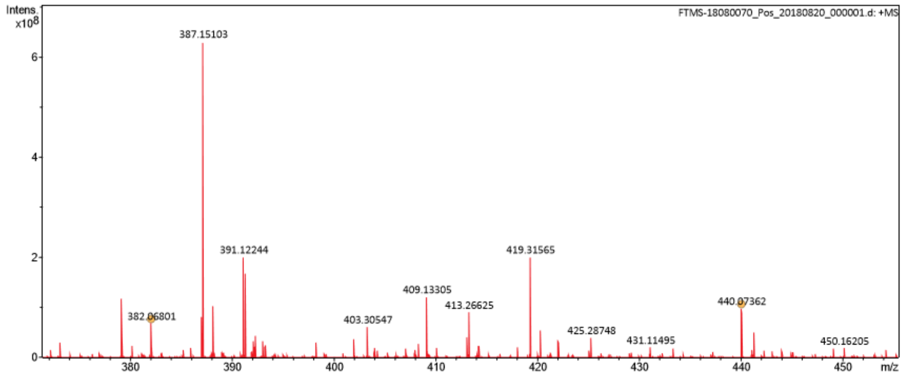

Meas. m/z	#	Ion Formula	Score	m/z	err [ppm]	rdb	e⁻ Conf	N-Rule	I
382.068013	1	C13H19MnN4O6	100.00	382.067956	-0.1	7.0	even	-	69142664
440.073618	1	C15H21MnN4O8	100.00	440.073435	-0.4	8.0	even	-	98724816

3) 15c-Metal complexes and Staphylophine complexes

[M+H]+ of [M-Sta] was calculated as $C_{13}H_{19}N_4O_6M$

[M+H]+ of [M-Pseu-linker] was calculated as $C_{15}H_{21}N_4O_8M$

3.1 15c-Cu Complexes and Staphylophine-Cu Complexes

Peking University Mass Spectrometry Sample Analysis Report

Analysis Info

Analysis Name	FTMS-18080071_Pos_20180820_000002.d	Acquisition Date	8/20/2018 11:42:10 AM
Sample	wss-0496-SZ-Cu	Instrument	Bruker Solarix XR FTMS
Comment		Operator	Peking University

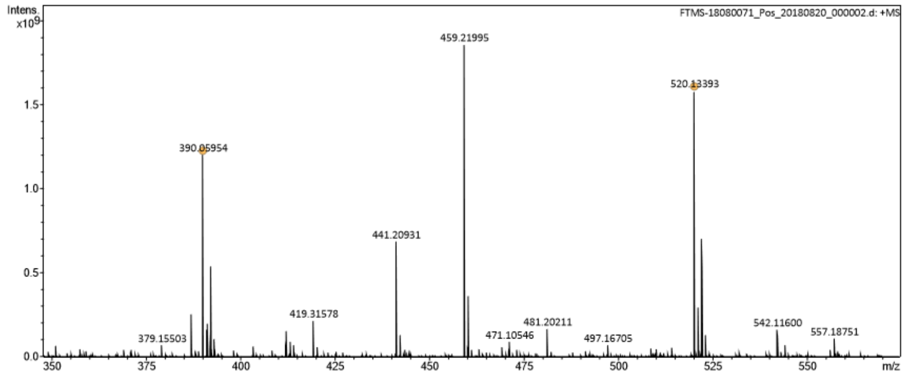

Meas. m/z	#	Ion Formula	Score	m/z	err [ppm]	rdb	e¯ Conf	N-Rule	I
390.059537	1	C13H19CuN4O6	100.00	390.059508	-0.1	6.5	odd	ok	1202426368
520.133933	1	C18H29CuN6O8	100.00	520.133736	-0.4	7.5	odd	ok	1584576896

Peking University Mass Spectrometry Sample Analysis Report

Analysis Info

Analysis Name	FTMS-18080071_Pos_20180820_000001.d	Acquisition Date	8/20/2018 11:41:58 AM
Sample	wss-0496-SZ-Cu	Instrument	Bruker Solarix XR FTMS
Comment		Operator	Peking University

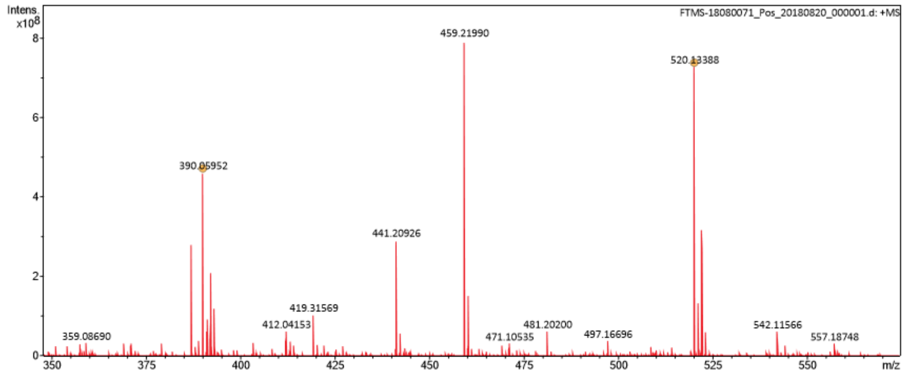

Meas. m/z	#	Ion Formula	Score	m/z	err [ppm]	rdb	e¯ Conf	N-Rule	I
390.059518	1	C13H19CuN4O6	100.00	390.059508	-0.0	6.5	odd	ok	461976064
520.133877	1	C18H29CuN6O8	100.00	520.133736	-0.3	7.5	odd	ok	727656832

3.2 15c-Ni Complexes and Staphylophine-Ni Complexes

Peking University Mass Spectrometry Sample Analysis Report

Analysis Info

Analysis Name	FTMS-18080074_Pos_20180820_000002.d	Acquisition Date	8/20/2018 11:54:18 AM
Sample	wss-0496-SZ-Ni	Instrument	Bruker Solarix XR FTMS
Comment		Operator	Peking University

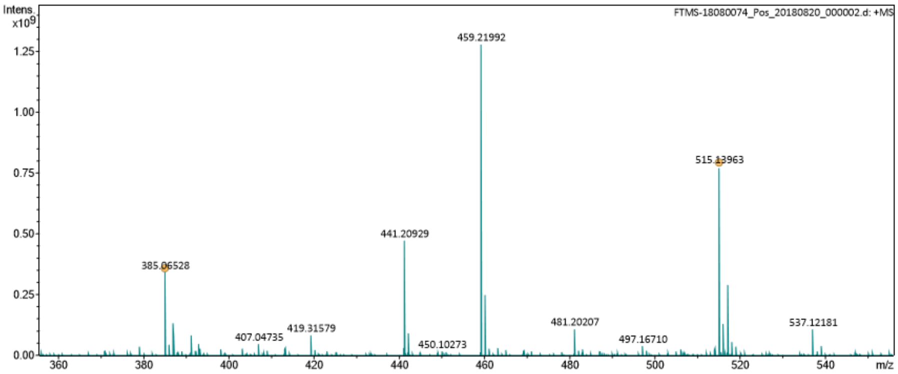

Meas. m/z	#	Ion Formula	Score	m/z	err [ppm]	rdb	e⁻ Conf	N-Rule	I
385.065276	1	C13H19N4NiO6	100.00	385.065254	-0.1	7.0	even	ok	343434368
515.139633	1	C18H29N6NiO8	100.00	515.139481	-0.3	8.0	even	ok	777142208

Peking University Mass Spectrometry Sample Analysis Report

Analysis Info

Analysis Name	FTMS-18080074_Pos_20180820_000001.d	Acquisition Date	8/20/2018 11:54:06 AM
Sample	wss-0496-SZ-Ni	Instrument	Bruker Solarix XR FTMS
Comment		Operator	Peking University

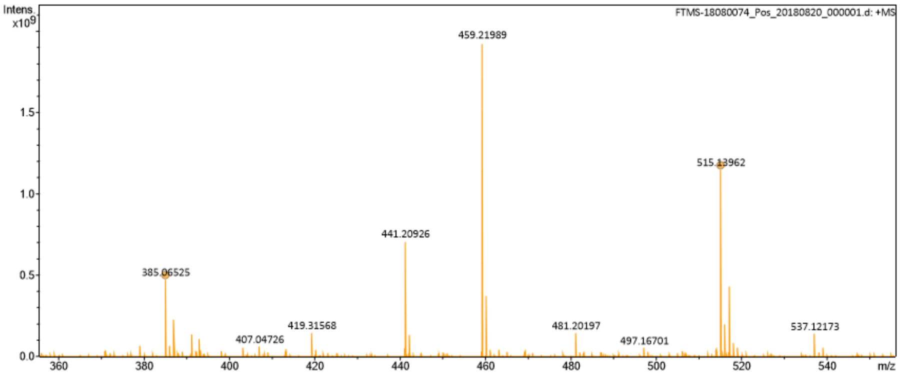

Meas. m/z	#	Ion Formula	Score	m/z	err [ppm]	rdb	e⁻ Conf	N-Rule	I
385.065255	1	C13H19N4NiO6	100.00	385.065254	-0.0	7.0	even	ok	477076704
515.139624	1	C18H29N6NiO8	100.00	515.139481	-0.3	8.0	even	ok	1150678272

3.3 15c-Zn Complexes and Staphylophine-Zn Complexes

Peking University Mass Spectrometry Sample Analysis Report

Analysis Info

Analysis Name	FTMS-18080076_Pos_20180820_000002.d	Acquisition Date	8/20/2018 12:06:41 PM
Sample	wss-0496-SZ-Zn	Instrument	Bruker Solarix XR FTMS
Comment		Operator	Peking University

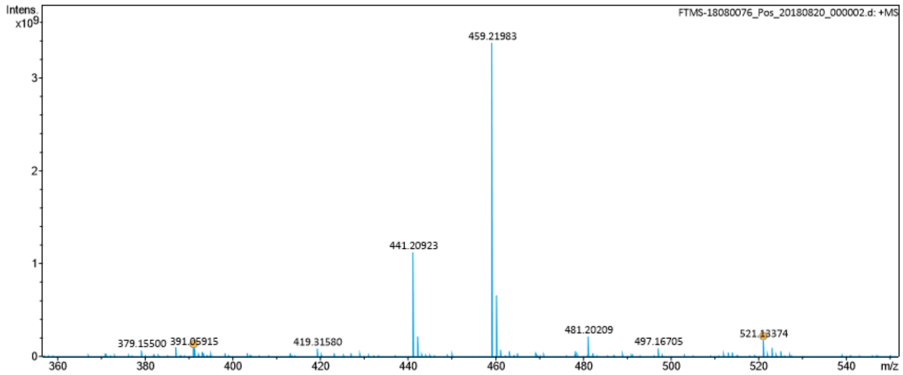

Meas. m/z	#	Ion Formula	Score	m/z	err [ppm]	rdb	e⁻ Conf	N-Rule	I
391.059154	1	C13H19N4O6Zn	100.00	391.059053	-0.3	7.0	even	ok	90750464
521.133743	1	C18H29N6O8Zn	100.00	521.133281	-0.9	8.0	even	ok	179796496

Peking University Mass Spectrometry Sample Analysis Report

Analysis Info

Analysis Name	FTMS-18080076_Pos_20180820_000001.d	Acquisition Date	8/20/2018 12:06:28 PM
Sample	wss-0496-SZ-Zn	Instrument	Bruker Solarix XR FTMS
Comment		Operator	Peking University

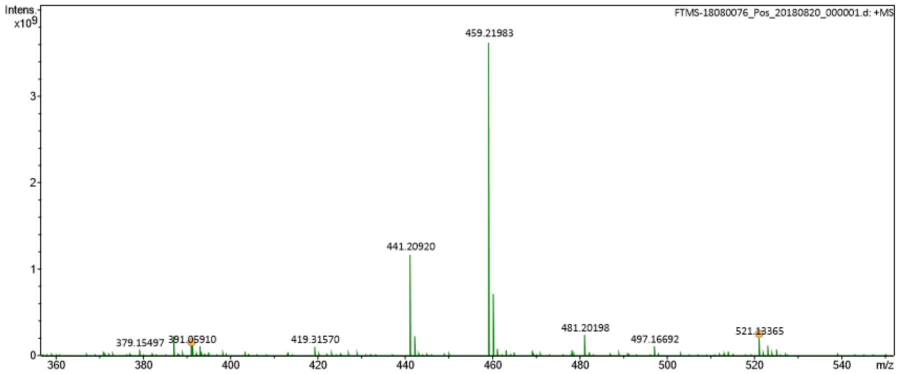

Meas. m/z	#	Ion Formula	Score	m/z	err [ppm]	rdb	e⁻ Conf	N-Rule	I
391.059102	1	C13H19N4O6Zn	100.00	391.059053	-0.1	7.0	even	ok	110415616
521.133649	1	C18H29N6O8Zn	100.00	521.133281	-0.7	8.0	even	ok	208558512

3.4 15c-Co Complexes and Staphylophine-Co Complexes

Peking University Mass Spectrometry Sample Analysis Report

Analysis Info

Analysis Name	FTMS-18080073_Pos_20180820_000001.d	Acquisition Date	8/20/2018 11:48:04 AM
Sample	wss-0496-SZ-Co	Instrument	Bruker Solarix XR FTMS
Comment		Operator	Peking University

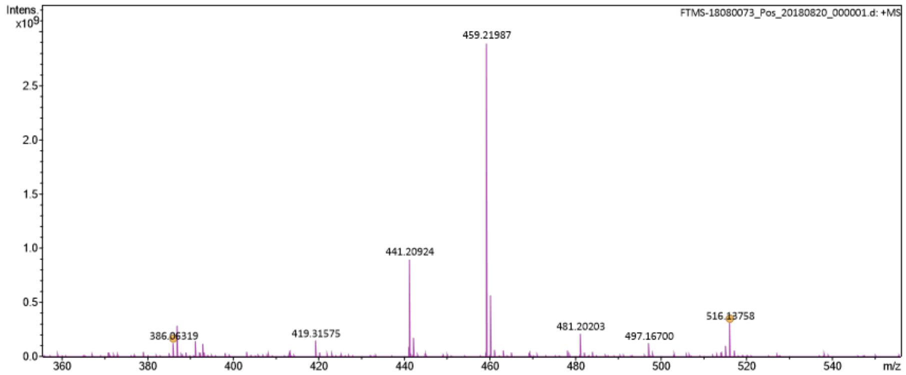

Meas. m/z	#	Ion Formula	Score	m/z	err [ppm]	rdb	e⁻ Conf	N-Rule	I
386.063192	1	C13H19CoN4O6	100.00	386.063106	-0.2	7.0	even	-	130852272
516.137579	1	C18H29CoN6O8	100.00	516.137333	-0.5	8.0	even	-	314113408

Peking University Mass Spectrometry Sample Analysis Report

Analysis Info

Analysis Name	FTMS-18080073_Pos_20180820_000002.d	Acquisition Date	8/20/2018 11:48:15 AM
Sample	wss-0496-SZ-Co	Instrument	Bruker Solarix XR FTMS
Comment		Operator	Peking University

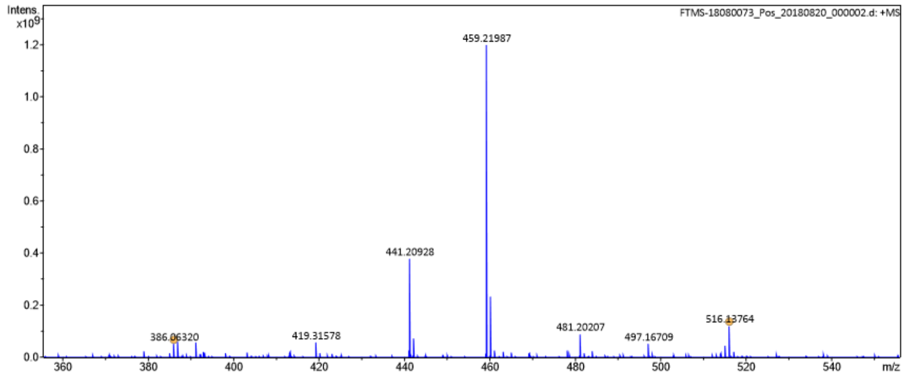

Meas. m/z	#	Ion Formula	Score	m/z	err [ppm]	rdb	e⁻ Conf	N-Rule	I
386.063202	1	C13H19CoN4O6	100.00	386.063106	-0.2	7.0	even	-	52798276
516.137640	1	C18H29CoN6O8	100.00	516.137333	-0.6	8.0	even	-	122784616

3.5 15c-Fe Complexes and Staphylophine-Fe Complexes

Peking University Mass Spectrometry Sample Analysis Report

Analysis Info

Analysis Name	FTMS-18080089_Pos_20180820_000002.d	Acquisition Date	8/20/2018 2:33:21 PM
Sample	wss-0496-SZ-Fe	Instrument	Bruker Solarix XR FTMS
Comment		Operator	Peking University

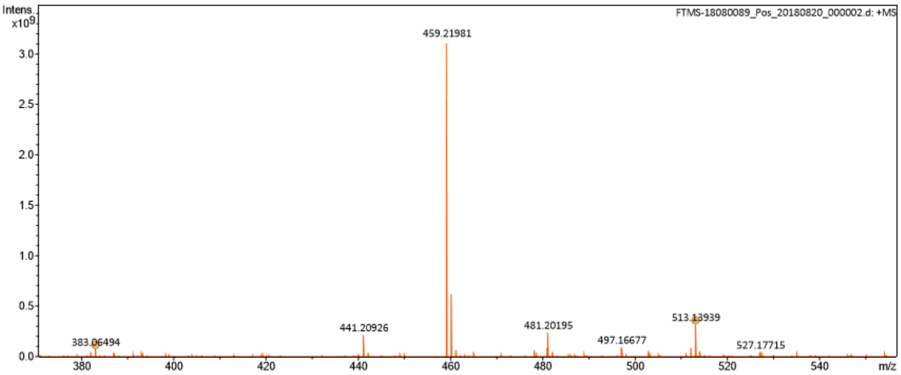

Meas. m/z	#	Ion Formula	Score	m/z	err [ppm]	rdb	e⁻ Conf	N-Rule	I
383.064942	1	C13H19FeN4O6	100.00	383.064848	-0.2	7.0	even	ok	77252280
513.139385	1	C18H29FeN6O8	100.00	513.139076	-0.6	8.0	even	ok	322008096

Peking University Mass Spectrometry Sample Analysis Report

Analysis Info

Analysis Name	FTMS-18080089_Pos_20180820_000001.d	Acquisition Date	8/20/2018 2:33:11 PM
Sample	wss-0496-SZ-Fe	Instrument	Bruker Solarix XR FTMS
Comment		Operator	Peking University

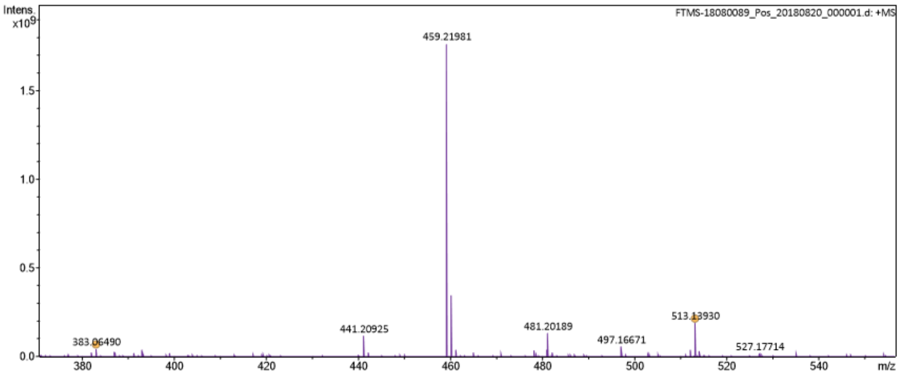

Meas. m/z	#	Ion Formula	Score	m/z	err [ppm]	rdb	e⁻ Conf	N-Rule	I
383.064900	1	C13H19FeN4O6	100.00	383.064848	-0.1	7.0	even	ok	45801936
513.139301	1	C18H29FeN6O8	100.00	513.139076	-0.4	8.0	even	ok	192920560

3.6 15c-Mn Complexes and Staphylophine-Mn Complexes

Peking University Mass Spectrometry Sample Analysis Report

Analysis Info

Analysis Name	FTMS-18080075_Pos_20180820_000002.d	Acquisition Date	8/20/2018 11:59:17 AM
Sample	wss-0496-SZ-Mn	Instrument	Bruker Solarix XR FTMS
Comment		Operator	Peking University

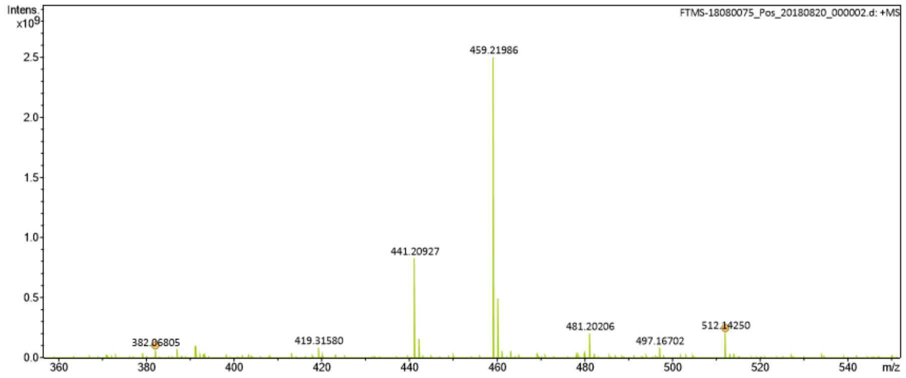

Meas. m/z	#	Ion Formula	Score	m/z	err [ppm]	rdb	e⁻ Conf	N-Rule	I
382.068048	1	C13H19MnN4O6	100.00	382.067956	-0.2	7.0	even	-	70222208
512.142500	1	C18H29MnN6O8	100.00	512.142183	-0.6	8.0	even	-	215342480

Peking University Mass Spectrometry Sample Analysis Report

Analysis Info

Analysis Name	FTMS-18080075_Pos_20180820_000001.d	Acquisition Date	8/20/2018 11:59:06 AM
Sample	wss-0496-SZ-Mn	Instrument	Bruker Solarix XR FTMS
Comment		Operator	Peking University

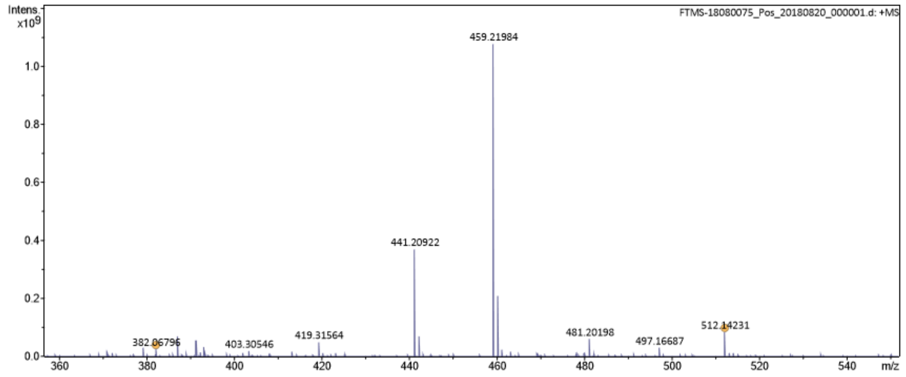

Meas. m/z	#	Ion Formula	Score	m/z	err [ppm]	rdb	e⁻ Conf	N-Rule	I
382.067961	1	C13H19MnN4O6	100.00	382.067956	-0.0	7.0	even	-	25787570
512.142307	1	C18H29MnN6O8	100.00	512.142183	-0.2	8.0	even	-	84966288

VIII) X-ray crystal structures

1. Crystal data and X-ray structure refinement for 14c (CCDC 1850196):

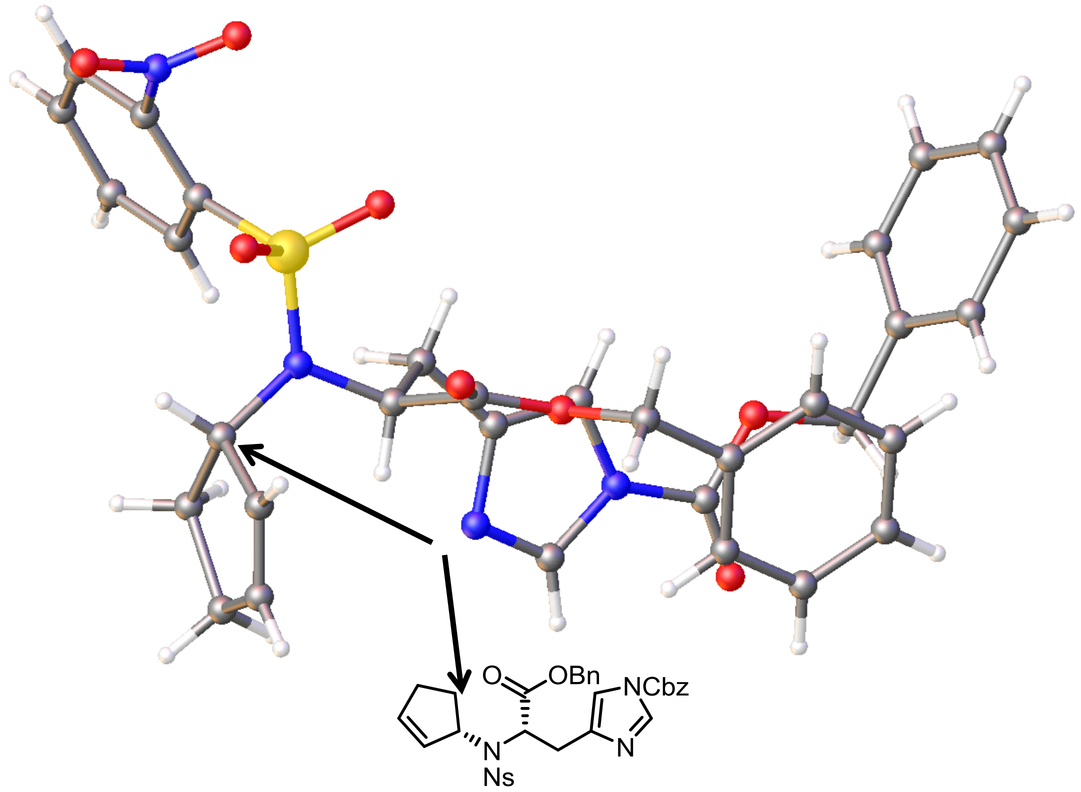

7c

Crystal data and X-ray structure refinement for **7c**.

Identification code	Compound **7c**
Empirical formula	$C_{32}H_{30}N_4O_8S$
Formula weight	630.66
Temperature/K	179.99(11)
Crystal system	orthorhombic
Space group	$P2_12_12_1$
a/Å	7.7277(2)
b/Å	11.2221(3)
c/Å	35.0781(8)
α/°	90
β/°	90
γ/°	90
Volume/Å^3	3042.01(13)
Z	4
ρ_{calc}g/cm^3	1.377
μ/mm^{-1}	0.165
F(000)	1320.0
Crystal size/mm^3	0.3 × 0.08 × 0.04
Radiation	MoKα (λ = 0.71073)
2Θ range for data collection/°	3.81 to 54.968
Index ranges	-10 ≤ h ≤ 10, -14 ≤ k ≤ 14, -45 ≤ l ≤ 45
Reflections collected	68493
Independent reflections	6961 [R_{int} = 0.0496, R_{sigma} = 0.0269]
Data/restraints/parameters	6961/0/406
Goodness-of-fit on F^2	1.017
Final R indexes [I>=2σ (I)]	R_1 = 0.0344, wR_2 = 0.0881
Final R indexes [all data]	R_1 = 0.0391, wR_2 = 0.0907
Largest diff. peak/hole / e Å^{-3}	0.25/-0.28

Flack parameter	-0.03(2)

2. Crystal data and X-ray structure refinement for 20b (CCDC 1863986):

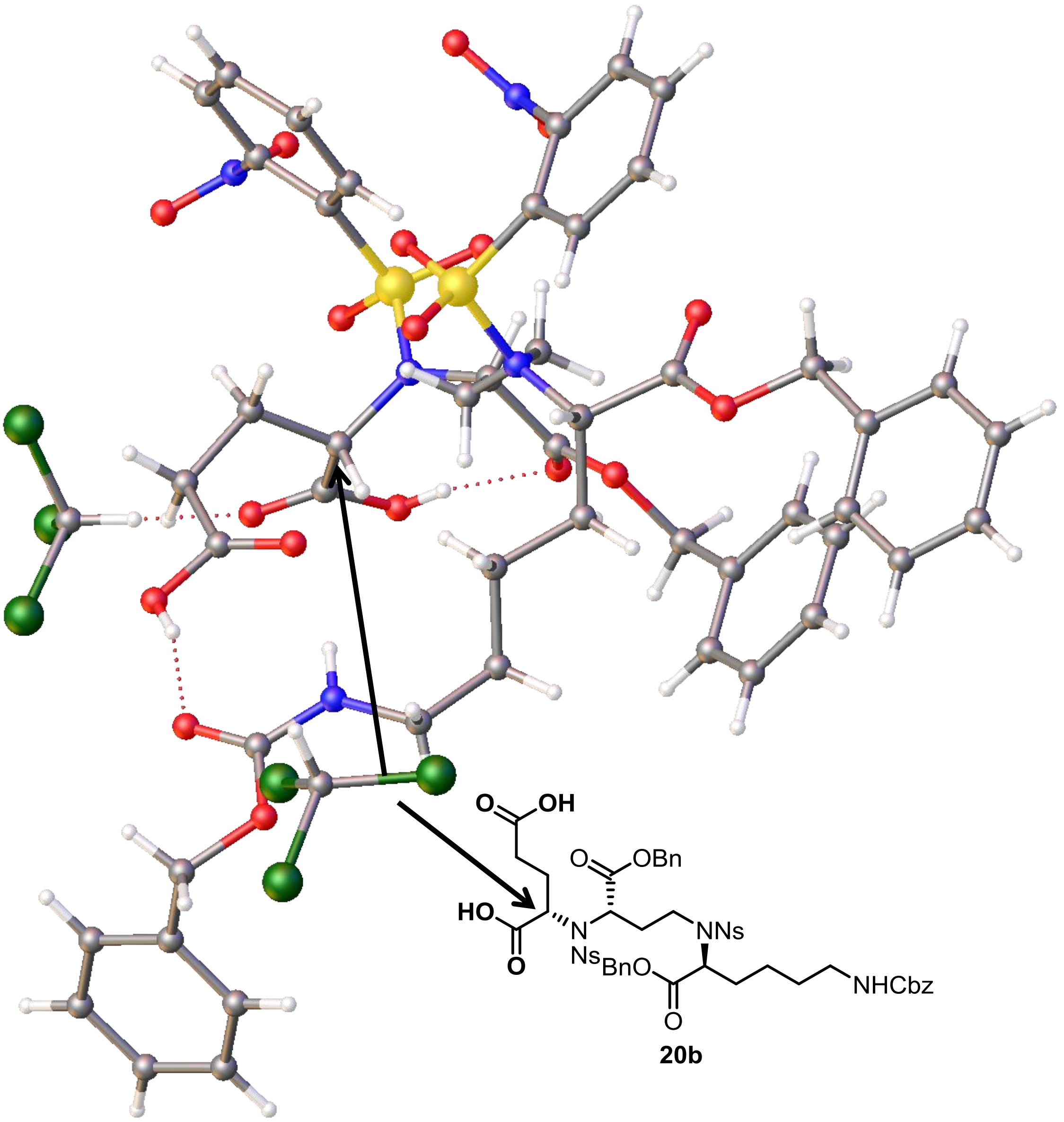

20b

Crystal data and structure refinement for 20b.

Identification code	Compound **20b**
Empirical formula	$C_{51}H_{53}Cl_6N_5O_{18}S_2$
Formula weight	1300.80
Temperature/K	180.00(10)
Crystal system	monoclinic
Space group	$P2_1$
a/Å	14.5442(2)
b/Å	14.2345(2)
c/Å	14.7460(2)
α/°	90
β/°	106.978(2)
γ/°	90
Volume/Å^3	2919.80(8)
Z	2
ρ_{calc}g/cm^3	1.480
μ/mm^{-1}	0.441
F(000)	1344.0
Crystal size/mm^3	0.25 × 0.12 × 0.08

Radiation	Mo Kα (λ = 0.71073)
2Θ range for data collection/°	4.066 to 54.97
Index ranges	$-18 \le h \le 18$, $-18 \le k \le 18$, $-19 \le l \le 19$
Reflections collected	68502
Independent reflections	13403 [R_{int} = 0.0329, R_{sigma} = 0.0287]
Data/restraints/parameters	13403/2/738
Goodness-of-fit on F^2	1.039
Final R indexes [I>=2σ (I)]	R_1 = 0.0552, wR_2 = 0.1421
Final R indexes [all data]	R_1 = 0.0611, wR_2 = 0.1458
Largest diff. peak/hole / e $Å^{-3}$	0.83/-0.75
Flack parameter	0.002(11)

IX) References

(1) G. Ghssein, C. Brutesco, L. Ouerdane, C. Fojcik, A. Izaute, S. Wang, C. Hajjar, R. Lobinski, D. Lemaire, P. Richaud, R. Voulhoux, A. Espaillat, F. Cava, D. Pignol, E. B. Durant, P. Arnoux, Science 2016, 352, 1105−1109.

(2) M. C. Mastropasqua, M. D'Orazio, M. Cerasi, F. Pacello, A. Gismondi, A. Canini, L. Canuti, A. Consalvo, D. Ciavardelli, B. Chirullo, P. Pasquali, A. Battistoni, *Mol. Microbiol.* **2017**, *106*, 543-561.

(3) T.T. Hoang, R. R. Karkhoff-Schweizer, A. J. Kutchma, H. P. Schweizer, *Gene.* **1998***, 212*, 77-86.

(4) S. Noël, L. Guillon, I. J. Schalk, G. t. L. Mislin, *Org. Lett.* **2011**, *13*, 844−847.

(5) J. Zhang, S. Wang, Y. Bai, Q. Guo, J. Zhou, X. Lei, *J. Org. Chem.* **2017**, *82*, 13643-13648.
